# A General Method to Access Underexplored Ylideneamino Sulfates as Interrupted Beckmann-Type Rearrangement Intermediates

**DOI:** 10.3390/molecules29071667

**Published:** 2024-04-08

**Authors:** Yifei Zhou, Alan M. Jones

**Affiliations:** School of Pharmacy, University of Birmingham, Edgbaston, Birmingham B15 2TT, UK

**Keywords:** Beckmann, rearrangement, sulfation, oxime, interrupted, ylideneamino sulfate

## Abstract

The Beckmann rearrangement of ketoximes to their corresponding amides, using a Brønsted acid-mediated fragmentation and migration sequence, has found wide-spread industrial application. We postulated that the development of a methodology to access ylideneamino sulfates using tributylsulfoammonium betaine (TBSAB) would afford isolable Beckmann-type intermediates and competent partners for subsequent rearrangement cascades. The ylideneamino sulfates generated, isolated as their tributylammonium salts, are sufficiently activated to undergo Beckmann rearrangement without additional reagent activation. The generation of sulfuric acid in situ from the ylideneamino sulfate giving rise to a routine Beckmann rearrangement and additional amide bond cleavage to the corresponding aniline was detrimental to reaction success. The screening of bases revealed inexpensive sodium bicarbonate to be an effective additive to prevent classic Brønsted acid-mediated fragmentation and achieve optimal conversions of up to 99%.

## 1. Introduction

**Figure 1:**
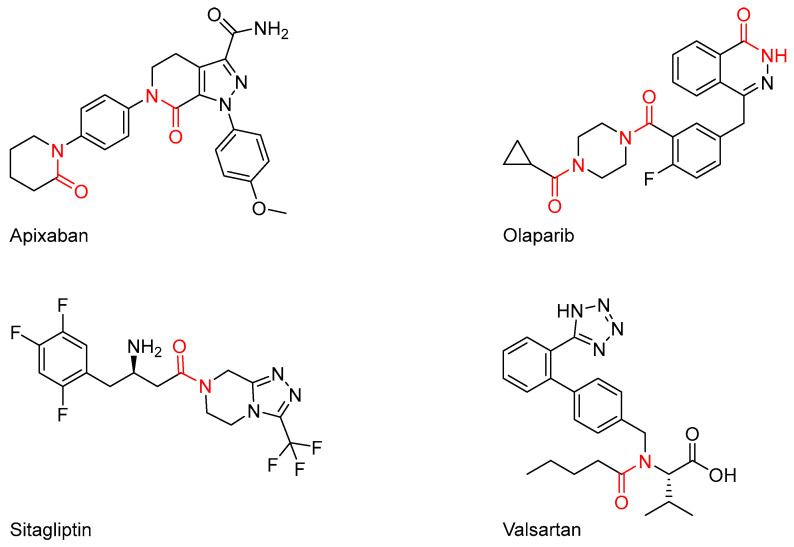
Amide bonds found in commonly encountered medicines (highlighted in red).

The amide functional group is one of the most important and widely encountered structural units in organic chemistry and biochemistry. The amide bond is commonly found in bioactive drug molecules including valsartan, an angiotensin receptor blocker (ARB) [1]; sitagliptin, an anti-diabetic medicine used to treat type-2 diabetes [2]; olaparib, a PARP inhibitor used to treat BRCA-mutated advanced ovarian cancer [3]; and apixaban, an anti-coagulant used to prevent stroke in nonvalvular atrial fibrillation via the inhibition of Factor Xa (Figure 1) [4].

The Beckmann rearrangement, which involves the conversion of an oxime to an amide [5,6], represents an attractive method for the synthesis of amides due to its simple operation, high selectivity, and atom economy. The Beckmann rearrangement is typically carried out by treating a ketoxime with a strong acid, such as sulfuric acid or phosphoric acid, at an elevated temperature (130 °C). The Beckmann rearrangement has been modified and updated using catalysts [7,8], acidic ionic liquids [9,10,11], radicals [12], photochemistry [13], reagent improvements [14,15], and recently, an example of an interrupted Beckmann rearrangement [16]. In turn, the amides generated are important substrates for electrosynthesis applications [17,18,19,20].

**Scheme 1:**
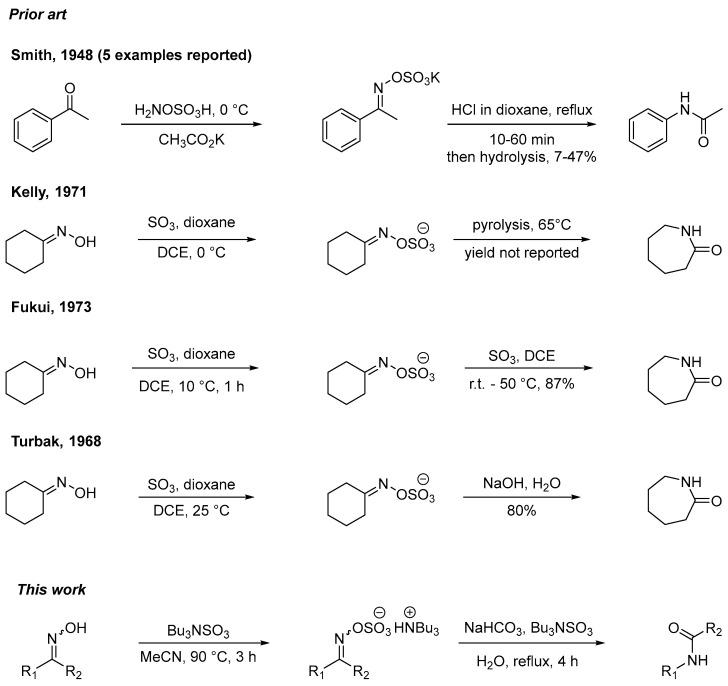
Prior art and this work. R_1_ and R_2_ can be H, alkyl, aryl, or acyl. Dioxane cations omitted for clarity [21,22,23,24].

There are limited known methods for the use of ylideneamino sulfates as a Beckmann intermediate (Scheme 1). The rearrangement of potassium salts of ketoxime *O*-sulfates prepared via the reaction of the respective ketone with hydroxylamine-*O*-sulfonic acid gave amide products in very limited examples (5 exemplars, 7–47% yield) with multiple by-products formed using anhydrous hydrochloric acid mediated rearrangement conditions [21].

The use of reactive liquid sulfur trioxide in dioxane resulted in the isolation of the *O*-sulfate of cyclohexanone oxime [22,23,24]. Kelly observed that cyclohexanone oxime sulfate undergoes exothermic rearrangement under mild heating [22]. This contrasts with the work of Fukui who did not observe this phenomenon but instead explored a range of Lewis bases (alkylamines, arylamines), Lewis acids (sulfur trioxide, zinc chloride, tin tetrachloride), hydrochloric acid, water, and methanol-mediated effects on the cyclohexanone oxime sulfate intermediate [23]. It was found that sulfur trioxide was an optimal additive for the rearrangement step to caprolactam in this single example [23]. A series of complexing agents (pyridine, dioxane, triethyl phosphate, DMF) with sulfur trioxide have been explored to prepare caprolactam via the *O*-sulfate and subsequent treatment with aqueous sodium hydroxide [24].

Inspired by these findings in Scheme 1, we initially considered whether tributylsulfoammonium betaine (TBSAB) [25,26,27,28,29,30] would be an effective sulfation reagent for the generation of *O*-sulfated oximes (ylideneamino sulfates), a functional group with a paucity of methods to tractably access [31]. It was anticipated that these ylideneamino sulfates could act as intercepted Beckmann rearrangement intermediates [15].

## 2. Results and Discussion

Attempts to optimize the *O*-oxime sulfation step with TBSAB are reported in Table 1 using the model substrate 1-phenylethan-1-one oxime (**1a**). The optimal conditions identified (Table 1, entry 1) required 2.0 equivalents of TBSAB in acetonitrile at 90 °C for 3 h, consistent with the *O*-sulfation of alcohol functional groups [30].

**Chart 1:**
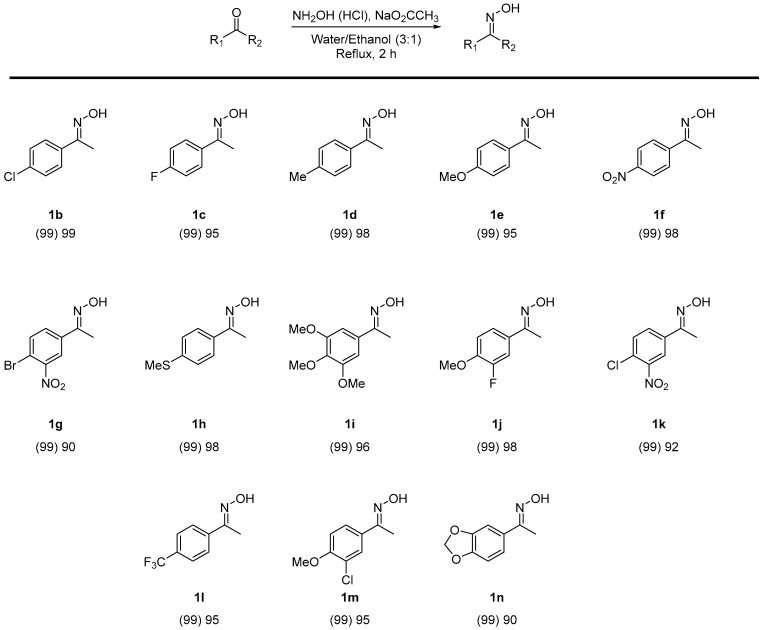
Synthesis of oxime substrates. Percentage conversion is given in parentheses as measured by ^1^H NMR spectroscopy. An isolated yield is given thereafter.

Due to a lack of commercially available oximes, 13 additional oximes were synthesized [32] from their respective ketones with hydroxylamine, in excellent yields of 90–99% (Chart 1).

**Chart 2:**
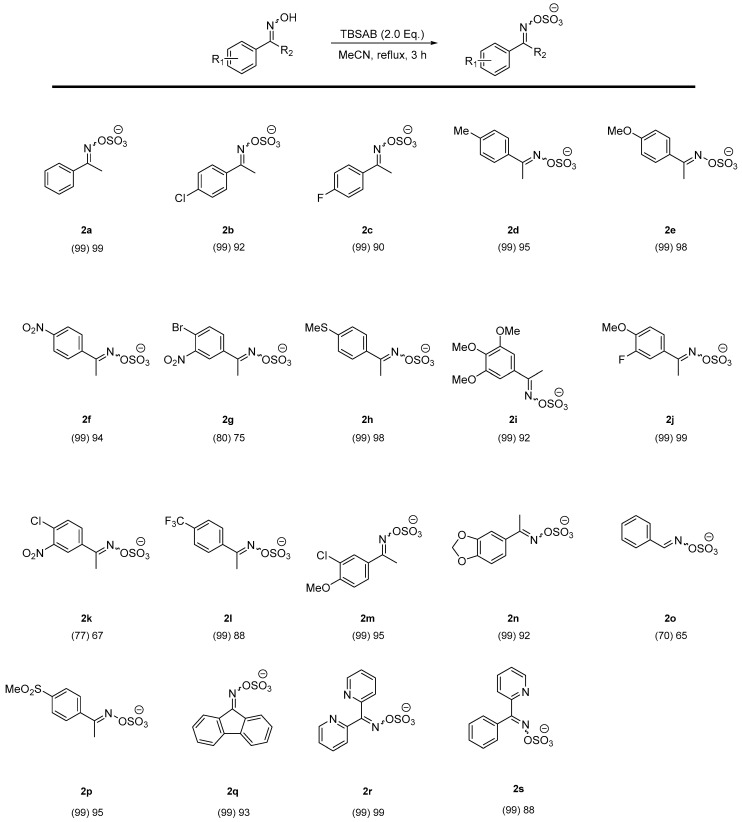
Screening of ylideneamino sulfate formation. *Cation omitted for clarity (^+^NBu_3_H). The percentage conversion is given in parentheses as measured by ^1^H NMR spectroscopy. An isolated yield is given thereafter.

With the optimal conditions in hand (Table 1) and a range of oxime substrates prepared (Chart 1), these were then in turn explored using TBSAB as the *O*-sulfating agent (Chart 2).

The *O*-sulfation of ketoximes (Chart 2) all showed good to excellent conversions as measured by ^1^H NMR spectroscopy and isolated yields (typically 67–98%) after an extractive purification protocol (Supplementary materials). These results also showed that mono-substituted aromatic oximes were not influenced by the attached groups from highly electron-donating methoxy groups (**2e**) to highly electron-withdrawing nitro groups (**2f**). However, compound **2o**, starting from an aldoxime, gave a relatively low yield in comparison to the ketoximes. A potential rationale for this finding was due to the higher reactivity of the aldehyde functional group in comparison to the more hindered, and stabilized, ketone group found in the precursor to **2a** (*c.f.* **1a**). The instability of the aldehyde and resulting imine functionality may be one cause of the lower isolated yield in **2o**. For the poly-substituted examples, compounds **2i**, **2j**, and **2m** gave good yields whilst compounds **2g** and **2k** were affected by the two electron-withdrawing groups, giving a lower rate of conversion but acceptable yield of 67%. This contrasts with both the excellent conversion in **2f** containing a *para*-nitro electron-withdrawing group and a *para*-chloro inductive withdrawing group and resonance electron-donating group found in **2b**. A plausible rationale is both the *meta*-positioning of the nitro group leading to a destabilizing delta-positive effect adjacent to the carbonyl group and reduced solubility of the halogen and nitro containing **2g** and **2k**.

With a range of differentially substituted ylideneamino sulfates isolated, our attention turned to whether these intermediates would undergo a Beckmann-type rearrangement. Optimization studies are shown in Table 2.

Entries 1–3 (Table 2) studied the effects of temperature on the rearrangement and identified that 100 °C gave the highest conversion to **3a** at 54% (entry 2). With increased temperatures, the formation of aniline (**4a**) became more pronounced (20%, entry 3) via presumably the hydrolysis of the amide motif in **3a**.

The analogous thermal conditions in entries 1–3 were compared with an additional equivalent of TBSAB (entries 4–6) and gave no improvement. 

**Scheme 2:**
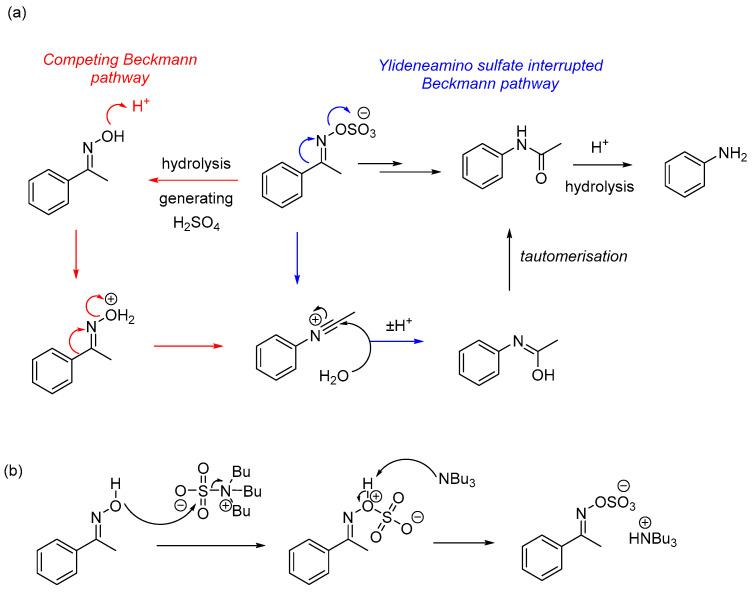
(**a**) Proposed mechanism of this work (blue) versus acid-mediated Beckmann mechanism (red); and (**b**) mechanism of the *O*-oxime sulfation step.

The possibility of the ylideneamino sulfate (**2a**) undergoing hydrolysis (entries 1–6, Table 2) to generate sulfuric acid and thus undergoing Brønsted-acid catalyzed rearrangement and/or further hydrolysis was initially overlooked. However, the low yields and multiple product formations made us consider two competing reaction pathways (Scheme 2). It should also be noted that the group *trans* to the oxime *O*-sulfate should migrate from a molecular orbital perspective [33]. However, under the reaction conditions, the *E*/*Z* conversion of the oxime *O*-sulfate is rapid, such that the following order of migratory capacity is adhered to aryl, alkenyl > tertiary alkyl > secondary alkyl > primary alkyl based on electron density effects.

Entries 7–10 (Table 2) were designed to solve this issue through the addition of different bases to intercept any acid-mediated pathways in operation. Entry 8 (Table 2) was selected as the optimized condition with six equivalents of NaHCO_3_ together with one equivalent of TBSAB at 100 °C for 4 h. Fortuitously, the increased yield and selectivity gained with the addition of sodium bicarbonate enabled a simple work-up to afford the amide products (Supplementary Materials).

The rationale for additional TBSAB being required is to return the formation of any trace oxime by-product (**1a**) to a competent reaction partner (**2a**) and increase the overall conversion to the desired amide product (**3a**) and concomitantly improve the purification protocol. The sulfur trioxide group in TBSAB may also activate the ylideneamino sulfate via coordination to the oxygen or the formation of pyrosulfate via analogy to the reported reactivity of sulfur trioxide-dioxane [23].

**Scheme 3:**
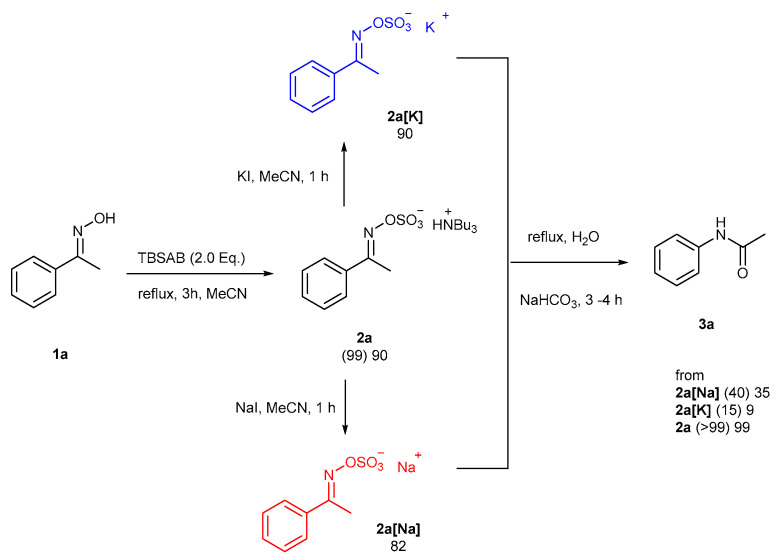
A control experiment to explore alternative sodium and potassium counter ion effects in the Beckmann-type rearrangement.

To consider the possibility of a counter ion effect of the base used, a controlled experiment was designed (Scheme 3). After sulfation of **1a**, the corresponding **2a** (as the HNBu_3_ salt) was converted into the inorganic salts of sodium, **2a[Na]** and potassium **2a[K]**, respectively, in highly isolated yields for comparison. The lower conversions for the Beckmann-type rearrangement (40 and 15%, sodium and potassium salts, respectively) showed that the exchange of HNBu_3_ in situ was unlikely with NaHCO_3_ and the conclusion was that the base acted merely to intercept any hydrolytic acid generated. The lower conversions with the inorganic alkali metal salts may also be due to the tributylammonium cation acting as a phase transfer catalyst for the highly polar sulfate group.

**Chart 3:**
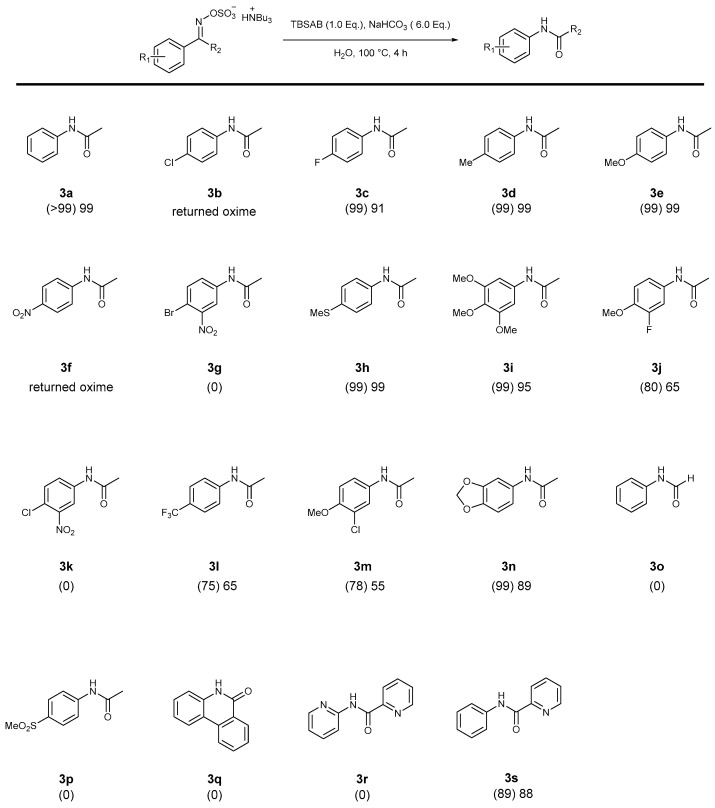
Screening for Beckmann-type rearrangement of the ylideneamino sulfates to amides. Percentage conversion is given in parentheses as measured by ^1^H NMR spectroscopy. An isolated yield is given thereafter.

With the optimized conditions in place, the nineteen ylideneamino sulfates were screened for Beckmann-type rearrangement competency (Chart 3). 

Electron-rich or moderately withdrawing mono-substituted aryl substrates gave excellent results, with 91–99% isolated yields (**3a**, **3c**, **3d**, **3e**, **3h** in Chart 3), and a trifluoromethyl example (**3l** in Chart 3) gave a lower 65% isolated yield under these conditions. More powerful electron withdrawing functional groups gave mixed results: **3p**, **3g**, and **3k** did not react and returned complex mixtures of products, whilst **3b** and **3f** returned the oxime starting material (**1b** and **1f**, respectively). A tentative reason for these unsuccessful examples can be elucidated from the electron withdrawing effect on the migratory capacity of the aryl ring system, which is clear in the starting materials **2p** and **2f**. Indeed, **2b** and **2f** gave clean (>99%) conversions back to **1b** and **1f** via inspection of ^1^H NMR spectra and comparison to authentic samples.

A similar pattern with poly-substituted substrates with electron-rich **3i**, **3j**, **3n**, and **3m** (Chart 3) proceeded in modest to high yields. Electron-withdrawing examples **3g** and **3k** did not participate. A rationale for this effect derives from the electron-rich nature required for the migratory group. Compound **3o**, an example of a sulfated aldoxime, did not participate in the reaction despite the generally increased reactivity of aldoximes in the Beckmann rearrangement [34] or the possibility of benzonitrile formation via dehydration [23]. The degradation of **2o** (precursor to **3o**) was observed, giving credence to the higher reactivity of the ketoxime example.

A cyclic example (**3q**) proved recalcitrant to these conditions. This may be attributed to the steric bulk around the reacting *O*-sulfate oxime and tributylammonium cation effect. This would be especially pronounced in a more rigid cyclic example, as was witnessed.

The deletion of one pyridyl group in **3s** vs. **3r** led to an excellent 88% yield vs. no reaction, respectively. Most likely, the deactivating nature of a bipyridyl example prevents the migratory step, as it is the phenyl group that migrates, not the pyridyl ring in **3s** which is not an available pathway to **3r**.

## 3. Conclusions

In summary, we have developed a general methodology to access nineteen isolable ylideneamino sulfates using tributylsulfoammonium betaine (TBSAB) in an up to 99% isolated yield, which was tolerant of a wide range of substituted and cyclic ket(ald)oximes. In turn, these novel ylideneamino sulfates, isolated as their tributylammonium salts, are sufficiently activated to undergo Beckmann-type rearrangement. The generation of sulfuric acid in situ from the ylideneamino sulfate giving rise to a classic Beckmann rearrangement and subsequent additional amide bond cleavage to the corresponding aniline was detrimental to reaction success. The screening of bases revealed inexpensive sodium bicarbonate to be an effective additive to prevent classic Brønsted acid-mediated fragmentation and achieve optimal conversions up to 99% in 11 examples with steric and electronic factors that influenced the outcome explored. A tentative mechanism is proposed based on the control experiments with alternative alkali metal cation intermediates. Taken together, these findings demonstrate an alternative protocol to activate and rearrange oximes to amides.

## 4. Experimental

All reactions involving moisture-sensitive reagents were carried out using standard Schlenk techniques in a dry reaction vessel under argon. All solvents used under anhydrous conditions were decanted directly from an SPS dispensary or were stored over 4 Å molecular sieves 24 h prior to use. 

Solvents used for workup procedures were of technical grade from Sigma-Aldrich, Honeywell, VWR, or Fisher Scientific. Unless stated otherwise, solvents were removed by rotary evaporation under reduced pressure between 30–50 °C. All chemical reagents were used as received unless stated otherwise. Reactions were monitored by TLC analysis on Merck silica gel 60 F254 using UV light (254 nm) and/or potassium permanganate. 

^1^H and ^13^C NMR spectra were recorded either on a Bruker AVIII operating at 300 MHz for ^1^H and fitted with a 5mm BBFO probe or on a Bruker AVANCE NEO operating at 400 MHz for ^1^H fitted with a 5mm “smart” BBFO probe, respectively. ^1^H-^1^H COSY, DEPT-45, ^1^H-^13^C HSQC, and ^1^H-^13^C HMBC NMR spectra were recorded on a Bruker AVANCE NEO console operating at 400 MHz for ^1^H and fitted with a nitrogen-cooled BBFO probe. Chemical shift data for ^1^H are reported in parts per million (ppm, δ scale) downfield from tetramethylsilane (TMS: δ 0.0) and referenced internally to the residual proton in the solvent. The deuterated solvents used for NMR analysis were chloroform (CDCl_3_: δH 7.26, δC 77.2), methanol (MeOD: δH 3.31, δC 49.2), and dimethyl sulfoxide (DMSO-d_6_: δH 2.50, δC 39.5). Coupling constants (*J*) are given in hertz (Hz). The data are presented as follows: chemical shift, multiplicity (s = singlet, d = doublet, t = triplet, q = quartet, p = pentet, m = multiple, br = broad, app = apparent and combinations thereof), coupling constant, and integration and assignment. 

Mass spectra were recorded on a Waters Xevo G2-XS ToF or Synap G2-S mass spectrometer using Zspray and Electro-spray ionization in negative (ESI-) and positive (ESI+) mode, respectively.

General procedure 1: Synthetic procedure for the preparation of oximes.

A magnetically stirred mixture of the corresponding acetophenone (5.0 mmol), NH_2_OH·HCl (0.520 g, 7.5 mmol), and sodium acetate (1.025 g, 12.5 mmol) in ethanol/water (20 mL of a 1:3 *v*/*v* mixture) was heated under reflux for 2 h. The precipitate was formed upon cooling and the reaction mixture was isolated by filtration and washed with water (3 × 10 mL) to give the desired oximes.

General procedure 2. Synthetic procedure for the preparation of sulfates using tributyl sulfoammonium betaine (Bu_3_NSO_3_, TBSAB).

A flask was charged with the respective oxime (1.0 mmol) and TBSAB (528 mg, 2.0 mmol, 2.0 eq) was added under an argon atmosphere. Anhydrous MeCN was added (giving a concentration of 0.50 Mol dm^−3^ to the limiting oxime reagent). The reaction mixture was heated at 82 °C (reflux) for 3 h and monitored by TLC. After reaction completion, the flask was cooled to room temperature and the solvent was removed under reduced pressure. The reaction was quenched with cold water (10 mL) and filtered. The aqueous solution was extracted with EtOAc (4 × 50 mL). The organic layer was dried (MgSO_4_), filtered, and the filtrate solvent was removed in vacuo to afford the desired compound as a clear oil. 

General procedure 3. Synthetic procedure for the preparation of amides.

A 25 mL round-bottom flask was charged with the respective *O*-sulfate (1.0 mmol) from General procedure 1, TBSAB (256 mg, 1.0 mmol), NaHCO_3_ (504 mg, 6.0 mmol), and water (10 mL). The reaction was heated under reflux (100 °C) for 4 h and monitored by TLC. The crude product was washed with cold water (3 × 10 mL), filtered, and freeze-dried to afford the desired product.

Selected compound characterization 

*N*-phenylacetamide (**3a**)

Following general procedure 3: A round-bottom flask was charged with **2a** (400 mg, 1.0 mmol), TBSAB (256 mg, 1.0 mmol), NaHCO_3_ (504 mg, 6.0 mmol), and water (10 mL). The reaction was heated under reflux for 4 h, monitored by TLC. The crude product was washed with cold water (3 × 10 mL) and then filtered and freeze-dried to give the desired product as a white solid (163 mg, 99%). M.P. 112–114 °C; ^1^H NMR (400 MHz, DMSO-d_6_) δ_H_ 9.90 (s, 1H), 7.56 (d, *J* = 7.8 Hz, 2H), 7.28 (t, *J* = 7.8 Hz, 2H), 7.01 (t, *J* = 7.8 Hz, 1H), 2.03 (s, 3H); ^13^C NMR (101 MHz, DMSO-d_6_) δ_C_ 168.2, 139.8, 128.6, 122.9, 119.0, 23.6; LRMS *m*/*z* (ESI+) 136.07 ([M+H]^+^, 100%); HRMS *m*/*z* (ESI+) C_8_H_10_NO requires 136.0711, found 136.0710 ([M+H]^+^).

*N*-(4-fluorophenyl)acetamide (**3c**)

Following general procedure 3: A round-bottom flask was charged with **2c** (419 mg, 1.0 mmol), TBSAB (256 mg, 1.0 mmol), NaHCO_3_ (504 mg, 6.0 mmol), and water (10 mL). The reaction was heated under reflux for 4 h, monitored by TLC. The crude product was washed with cold water (3 × 10 mL) and then filtered and freeze-dried to give the desired product as a white solid (279 mg, 91%). M.P. 152–153 °C; ^1^H NMR (400 MHz, DMSO-d_6_) δ_H_ 9.96 (1 H, s), 7.61–7.55 (2 H, m), 7.15–7.09 (2 H, m), 2.02 (3 H, s); ^13^C NMR (101 MHz, DMSO-d_6_) δ_C_ 168.6, 159.5 (d, ^1^*J*_C-F_ = 239.4 Hz), 135.0 (d, ^4^*J*_C-F_ = 2.5 Hz), 121.2 (d, ^3^*J*_C-F_ = 7.5 Hz), 115.8 (d, ^2^*J*_C-F_ = 22.3 Hz), 25.0; ^19^F NMR (377 MHz, MeOD) δ −120.75; LRMS *m*/*z* (ESI+) 154.06 ([M+H]^+^, 100%); HRMS *m*/*z* (ESI+) C_8_H_9_FNO requires 154.0598, found 154.0599 ([M+H]^+^).

*N*-(4-methylphenyl)acetamide (**3d**)

Following general procedure 3: A round-bottom flask was charged with **2d** (414 mg, 1.0 mmol), TBSAB (256 mg, 1.0 mmol), NaHCO_3_ (504 mg, 6.0 mmol), and water (10 mL). The reaction was heated under reflux for 4 h, monitored by TLC. The crude product was washed with cold water (3 × 10 mL) and then filtered and freeze-dried to give the desired product as a white solid (148 mg, 99%). M.P. 151–153 °C; ^1^H NMR (400 MHz, DMSO-d_6_) δ_H_ 9.81 (1 H, s), 7.46–7.43 (2 H, m), 7.09–7.06 (2 H, m), 2.23 (3 H, s), 2.01 (3 H, s); ^13^C NMR (101 MHz, DMSO-d_6_) δ_C_ 168.5, 137.3, 132.6, 129.0, 121.2, 24.4, 19.5; LRMS *m*/*z* (ESI+) 150.08 ([M+H]^+^, 100%); HRMS *m*/*z* (ESI+) C_9_H_12_NO requires 150.0804, found 150.0802 ([M+H]^+^).

*N*-(4-methoxyphenyl)acetamide (**3e**)

Following general procedure 3: A round-bottom flask was charged with **2e** (411 mg, 1.0 mmol), TBSAB (256 mg, 1.0 mmol), NaHCO_3_ (504 mg, 6.0 mmol), and water (10 mL). The reaction was heated under reflux for 4 h, monitored by TLC. The crude product was washed with cold water (3 × 10 mL) and then filtered and freeze-dried to give the desired product as a white solid (163 mg, 99%). M.P. 125–128 °C; ^1^H NMR (400 MHz, DMSO-d_6_) δ_H_ 9.77 (s, 1H), 7.50–7.43 (m, 2H), 6.88–6.82 (m, 2H), 3.70 (s, 3H), 2.00 (s, 3H); ^13^C NMR (101 MHz, DMSO-d_6_) δ_C_ 169.7, 154.1, 134.8, 119.7, 115.5, 55.0, 26.0; LRMS *m*/*z* (ESI+) 166.08 ([M+H]^+^, 100%); HRMS *m*/*z* (ESI+) C_9_H_12_NO_2_ requires 166.0839, found 166.0842 ([M+H]^+^).

*N*-(4-methylthiophenyl)acetamide (**3h**)

Following general procedure 3: A 25 mL round-bottom flask was charged with **2h** (480 mg, 1.0 mmol), TBSAB (256 mg, 1.0 mmol), NaHCO_3_ (504 mg, 6.0 mmol), and water (10 mL). The reaction was heated under reflux for 4 h monitored by TLC. The crude product was washed with cold water (3 × 10 mL) and then filtered and freeze-dried to give the desired product as a white solid (179 mg, 99%). M.P. 125–128 °C; ^1^H NMR (400 MHz, DMSO-d_6_) δ_H_ 9.92 (s, 1H), 7.57–7.49 (m, 2H), 7.25–7.17 (m, 2H), 2.43 (s, 3H), 2.02 (s, 3H); ^13^C NMR (101 MHz, DMSO-d_6_) δ_C_ 170.6, 136.1, 131.8, 126.2, 118.4, 25.0, 17.1; LRMS *m*/*z* (ESI+) 182.06 ([M+H]^+^, 100%); HRMS *m*/*z* (ESI+) C_9_H_12_NOS requires 182.0603, found 182.0607 ([M+H]^+^).

*N*-(3,4,5-trimethoxyphenyl)acetamide (**3i**)

Following general procedure 3: A round-bottom flask was charged with **2i** (490 mg, 1.0 mmol), TBSAB (256 mg, 1.0 mmol), NaHCO_3_ (504 mg, 6.0 mmol), and water (10 mL). The reaction was heated under reflux for 4 h, monitored by TLC. The crude product was washed with cold water (3 × 10 mL) and then filtered and freeze-dried to give the desired product as a white solid (214 mg, 95%). M.P. 140–142 °C; ^1^H NMR (400 MHz, DMSO-d_6_) δ_H_ 9.85 (s, 1H), 6.95 (s, 2H), 3.72 (s, 6H), 3.60 (s, 3H), 2.01 (s, 3H); ^13^C NMR (101 MHz, DMSO-d_6_) δ_C_ 169.0, 155.2, 137.7, 134.3, 97.2, 59.5, 55.6, 24.5; LRMS *m*/*z* (ESI+) 226.10 ([M+H]^+^, 100%); HRMS *m*/*z* (ESI+) C_11_H_16_NO_4_ requires 226.1027, found 226.1022 ([M+H]^+^).

*N*-(3-fluoro-4-methoxyphenyl)acetamide (**3j**)

Following general procedure 3: A round-bottom flask was charged with **2j** (468 mg, 1.0 mmol), TBSAB (256 mg, 1.0 mmol), NaHCO_3_ (504 mg, 6.0 mmol), and water (10 mL). The reaction was heated under reflux for 4 h, monitored by TLC. The crude product was washed with cold water (3 × 10 mL) and then filtered and freeze-dried and then purified with (SiO_2_; Hexane/EtOAc, 1:1, R_f_ = 0.2) to give the desired product as a white solid (119 mg, 65%). M.P. 165–168 °C; ^1^H NMR (400 MHz, DMSO-d_6_) δ_H_ 9.93 (s, 1H), 7.56 (dd, *J* = 9.0, 2.5 Hz, 1H), 7.20 (ddd, *J* = 9.0, 2.5, 1.4 Hz, 1H), 7.08 (t, *J* = 9.0, 1.4 Hz, 1H), 3.78 (s, 3H), 2.01 (s, 3H); ^13^C NMR (101 MHz, DMSO-d_6_) δ_C_ 168.6 (d, *J*_C-F_ = 250.2 Hz), 160.1 (d, *J*_C-F_ = 3.2 Hz), 150.1 (d, *J*_C-F_ = 13.3 Hz), 146.6 (d, *J*_C-F_ = 8.4 Hz), 132.1 (d, *J*_C-F_ = 4.2 Hz), 114.9 (d, *J*_C-F_ = 1.5 Hz), 108.0 (d, *J*_C-F_ = 12.6 Hz), 56.6, 24.3; ^19^F NMR (377 MHz, DMSO-d_6_) δ_F_ −134.08; LRMS *m*/*z* (ESI+) 184.07 ([M+H]^+^, 100%); HRMS *m*/*z* (ESI+) C_9_H_11_FNO_2_ requires 184.0695, found 184.0692 ([M+H]^+^).

*N*-(4-trimethylfluorophenyl)acetamide (**3l**)

Following general procedure 3: A round-bottom flask was charged with **2l** (468 mg, 1.0 mmol), TBSAB (256 mg, 1.0 mmol), NaHCO_3_ (504 mg, 6.0 mmol), and water (10 mL). The reaction was heated under reflux for 4 h, monitored by TLC. The crude product was washed with cold water (3 × 10 mL) and then filtered, freeze-dried, and then purified with (SiO_2_; Hexane/EtOAc, 4:6, R_f_ = 0.2) to give the desired product as a white solid (132 mg, 65%). M.P. 103–104 °C; ^1^H NMR (400 MHz, DMSO-d_6_) δ_H_ 10.30 (s, 1H), 7.78 (d, *J* = 8.2 Hz, 2H), 7.65 (d, *J* = 8.2 Hz, 2H), 2.08 (s, 3H); ^13^C NMR (101 MHz, DMSO-d_6_) δ_C_ 169.3, 150.3, 141.9 (d, ^2^*J*_C-F_ = 31 Hz), 127.5 (q, ^4^*J*_C-F_ = 4.8 Hz), 127.3 (q, ^3^*J*_C-F_ = 12.6 Hz), 119.2 (d, ^1^*J*_C-F_ = 281.1 Hz), 24.6; ^19^F NMR (377 MHz, DMSO-d_6_) δ_F_ −59.25; LRMS *m*/*z* (ESI+) 204.06 ([M+H]^+^, 100%); HRMS *m*/*z* (ESI+) C_9_H_9_F_3_NO requires 204.0628, found 204.0627 ([M+H]^+^).

*N*-(3-chloro-4-methoxyphenyl)acetamide (**3m**)

Following general procedure 3: A round-bottom flask was charged with **2m** (465 mg, 1.0 mmol), TBSAB (256 mg, 1.0 mmol), NaHCO_3_ (504 mg, 6.0 mmol), and water (10 mL). The reaction was heated under reflux for 4 h, monitored by TLC. The crude product was washed with cold water (3 × 10 mL) and then filtered, freeze-dried, and then purified with (SiO_2_; Hexane/EtOAc, 1:1, R_f_ = 0.2) to give the desired product as a white solid (110 mg, 55%). M.P. 82–85 °C; ^1^H NMR (400 MHz, DMSO-d_6_) δ_H_ 9.93 (s, 1H), 7.76 (d, *J* = 2.6 Hz, 1H), 7.39 (dd, *J* = 8.9, 2.6 Hz, 1H), 7.08 (d, *J* = 8.9 Hz, 1H), 3.80 (s, 3H), 2.01 (s, 3H); ^13^C NMR (101 MHz, DMSO-d_6_) δ_C_ 168.5, 150.7, 133.5, 121.0, 120.9, 119.2, 113.4, 56.6, 23.9; LRMS *m*/*z* (ESI+) 200.04 ([M^35^Cl+H]^+^, 100%), 202.04 ([M^37^Cl+H]^+^, 60%); HRMS *m*/*z* (ESI+) C_9_H_11_ClNO_2_ requires 200.0441, found 200.0439 ([M^35^Cl+H]^+^).

*N*-(benzo[*d*][1,3]dioxol-5-yl)acetamide (**3n**)

Following general procedure 3: A round-bottom flask was charged with **2n** (414 mg, 1.0 mmol), TBSAB (256 mg, 1.0 mmol), NaHCO_3_ (504 mg, 6.0 mmol), and water (10 mL). The reaction was heated under reflux for 4 h, monitored by TLC. The crude product was washed with cold water (3 × 10 mL) and then filtered and freeze-dried to give the desired product as a white solid (159 mg, 80%). M.P. 135–137 °C; ^1^H NMR (400 MHz, DMSO-d_6_) δ_H_ 9.83 (s, 1H), 7.28 (d, *J* = 2.1 Hz, 1H), 6.92 (dd, *J* = 8.4, 2.1 Hz, 1H), 6.82 (d, *J* = 8.4 Hz, 1H), 5.96 (s, 2H), 1.99 (s, 3H); ^13^C NMR (101 MHz, DMSO-d_6_) δ_C_ 168.1, 146.5, 142.4, 134.7, 114.9, 108.9, 101.7, 101.3, 22.9; LRMS *m*/*z* (ESI+) 180.06 ([M+H]^+^, 100%); HRMS *m*/*z* (ESI+) C_9_H_10_NO_3_ requires 180.0622, found 180.0620 ([M+H]^+^).

*N*-(pyridin-2-yl)benzamide (**3s**)

Following general procedure 3: A round-bottom flask was charged with **2s** (445 mg, 1.0 mmol), TBSAB (256 mg, 1.0 mmol), NaHCO_3_ (504 mg, 6.0 mmol), and water (10 mL). The reaction was heated under reflux for 4 h while being monitored by TLC. The crude product was washed with cold water (3 × 10 mL) and then filtered and freeze-dried to give the desired product as a white solid (160 mg, 89%). M.P. 78–79 °C; ^1^H NMR (400 MHz, DMSO-d_6_) δ_H_ 10.62 (s, 1H), 8.74 (ddd, *J* = 4.8, 1.7, 1.1 Hz, 1H), 8.17 (dt, *J* = 7.9, 1.1 Hz, 1H), 8.07 (td, *J* = 7.9, 1.7 Hz, 1H), 7.93–7.89 (m, 2H), 7.68 (ddd, *J* = 7.9, 4.8, 1.1 Hz, 1H), 7.39–7.34 (m, 2H), 7.15–7.10 (m, 1H); ^13^C NMR (101 MHz, DMSO-d_6_) δ_C_ 162.9, 150.4, 148.9, 138.8, 138.6, 131.1, 129.2, 128.5, 127.4, 124.4, 122.8, 120.7; LRMS *m*/*z* (ESI+) 199.08 ([M+H]^+^, 100%); HRMS *m*/*z* (ESI+) C_12_H_11_N_2_O requires 199.0809, found 199.0813 ([M+H]^+^.

## Figures and Tables

**Table 1:** Identification of optimal *O*-oxime sulfation conditions on **1a**.

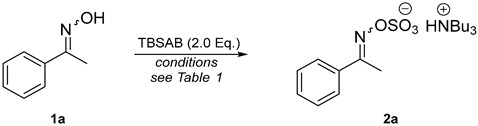				
**Entry**	**Solvent**	**Temperature [°C]**	**Time [h]**	**Conv. [%]**
1	MeCN	90	3	>99
2	MeCN	60	3	90
3	MeCN	30	>24	<10

**Table 2:** Optimization of the Beckmann rearrangement from **2a** to **3a** (and/or **1a** and **4a**).

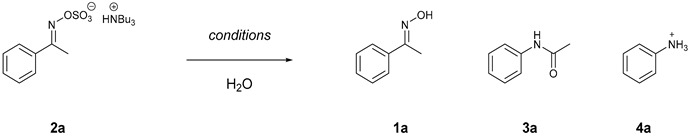							
**Entry**	**T (°C)**	**Time (h)**	**TBSAB (1.0 Equiv.)**	**Base [6.0 Equiv.]**	**Conv. [%] ^a^ 1a**	**Conv. [%] ^a^ 3a**	**Conv. [%] ^a^ 4a**
1	80	8	-	-	9	26	trace
2	100	8	-	-	6	54	14
3	120	8	-	-	7	37	20
4	80	8	Yes	-	5	40	trace
5	100	8	Yes	-	10	57	10
6	120	8	Yes	-	9	37	25
7 ^b^	100	4	-	NaHCO_3_	trace	>99	0
8 ^b^	100	4	Yes	NaHCO_3_	0	>99	0
9 ^b^	100	4	Yes	KHCO_3_	0	90	0
10 ^b^	100	4	Yes	pyridine	0	60	0

^a^ Conversions were determined by ^1^H NMR with internal standard. ^b^ Reaction time for entries 7–10 was reduced to 4 h.

## Data Availability

The original contributions presented in the study are included in the article/supplementary material, further inquiries can be directed to the corresponding author.

## Supplementary Materials

The following supporting information can be downloaded at: https://www.mdpi.com/article/10.3390/molecules29071667/s1, General procedures, compound characterization, and copies of ^1^H, ^13^C, and ^19^F NMR spectra; References are cited in [35,36,37,38,39,40,41,42,43,44,45].
