# Supplementary material for: A General Method to Access Underexplored Ylideneamino Sulfates as Interrupted Beckmann-Type Rearrangement Intermediates
Source: Molecules. 2024 Apr 8;29(7):1667. doi: 10.3390/molecules29071667 (PMC11013155; doi:10.3390/molecules29071667)
Supplement: Supplementary file 1 [file molecules-29-01667-s001.zip › molecules-2946379-supplementary.pdf]

SUPPORTING INFORMATION

A general method to access underexplored ylideneamino sulfates as interrupted Beckmann-type rearrangement intermediates

Yifei Zhou[1] and Alan M. Jones[1*]

[1]School of Pharmacy, University of Birmingham, Edgbaston, Birmingham, B15 2TT, United Kingdom

* corresponding author: (A.M.J.) a.m.jones.2@bham.ac.uk; +44(0)121-414-7288.

CONTENTS

GENERAL CONSIDERATIONS	Page 2
GENERAL PROCEDURES	Page 3-4
COMPOUND CHARACTERISATION	Page 5-31
Copies of ^{1}H, ^{13}C, ^{19}F NMR spectra	Page 32-130
REFERENCES	Page 131

GENERAL CONSIDERATIONS

All reactions involving moisture sensitive reagents were carried out using standard Schlenk techniques, in a dry reaction vessel under argon. All solvents used under anhydrous conditions were decanted directly from an SPS dispensary or were stored over 4 Å molecular sieves 24 h prior to use.

Solvents used for workup procedures were of technical grade from Sigma-Aldrich, Honeywell, VWR or Fisher Scientific. Unless stated otherwise, solvents were removed by rotary evaporation under reduced pressure between 30-50 °C. All chemical reagents were used as received unless stated otherwise. Reactions were monitored by TLC analysis on Merck silica gel 60 F254 using UV light (254 nm) and/or potassium permanganate.

^{1}H and ^{13}C NMR spectra were recorded either on a Bruker AVIII operating at 300 MHz for ^{1}H and fitted with a 5mm BBFO probe or on a Bruker AVANCE NEO operating at 400 MHz for ^{1}H fitted with a 5mm "smart" BBFO probe, respectively. ^{1}H-^{1}H COSY, DEPT-45, ^{1}H-^{13}C HSQC, and ^{1}H-^{13}C HMBC NMR spectra were recorded on a Bruker AVANCE NEO console operating at 400 MHz for 1H and fitted with a nitrogen-cooled BBFO probe. Chemical shift data for ^{1}H are reported in parts per million (ppm, δ scale) downfield from tetramethylsilane (TMS: δ 0.0) and referenced internally to the residual proton in the solvent. The deuterated solvents used for NMR analysis were chloroform ($CDCl_3$:δH 7.26, δC 77.2), methanol (MeOD: δH 3.31, δC 49.2) and dimethyl sulfoxide (DMSO-d_6: δH 2.50, δC 39.5). Coupling constants (*J*) are given in hertz (Hz). The data are presented as follows: chemical shift, multiplicity (s = singlet, d = doublet, t = triplet, q = quartet, p = pentet, m = multiple, br = broad, app = apparent and combinations thereof), coupling constant and integration and assignment.

Mass spectra were recorded on a Waters Xevo G2-XS ToF or Synap G2-S mass spectrometer using Zspray, Electro-spray ionization in negative (ESI^-) and positive (ESI^+) mode, respectively.

GENERAL PROCEDURES

General procedure 1: Synthetic procedure for the preparation of oximes.

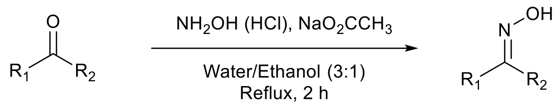

A magnetically stirred mixture of the corresponding acetophenone (5.0 mmol), $NH_2OH{\cdot}HCl$ (0.520 g, 7.5 mmol) and sodium acetate (1.025 g, 12.5 mmol) in ethanol/water (20 mL of a 1:3 v/v mixture) was heated under reflux for 2 h. The precipitate was formed upon cooling and the reaction mixture was isolated by filtration, washed by water (3 × 10 mL) to give the desired oximes.

General procedure 2. Synthetic procedure for the preparation of sulfates using tributyl sulfoammonium betaine (Bu_3NSO_3, TBSAB).

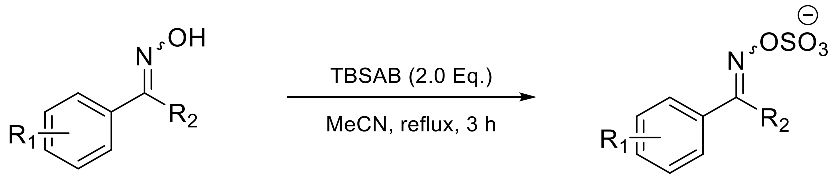

A flask was charged with the respective oxime (1.0 mmol) and TBSAB (528 mg, 2.0 mmol, 2.0 eq) was added under an argon atmosphere. Anhydrous MeCN was added (giving a concentration of 0.50 Mol dm^{-3} to the limiting oxime reagent). The reaction mixture was heated at 82°C (reflux) for 3 h and monitored by TLC. After reaction completion, the flask was cooled to room temperature and the solvent removed under reduced pressure. The reaction was quenched with cold water (10 mL) and filtered. The aqueous solution was extracted with EtOAc (4 x 50 mL). The organic layer was dried ($MgSO_4$), filtered, and the filtrate solvent was removed *in vacuo* to afford the desired compound as a clear oil.

General procedure 3. Synthetic procedure for the preparation of amides.

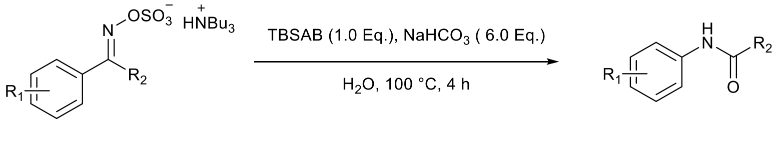

A 25 mL round bottom flask was charged with the respective *O*-sulfate (1.0 mmol) from **General procedure 1**, TBSAB (256 mg, 1.0 mmol), $NaHCO_3$ (504 mg, 6.0 mmol) and water (10 mL). The reaction was heated under reflux (100 °C) for 4 h and monitored by TLC. The

crude product was washed with cold water (3 × 10 mL), filtered, and freeze-dried to afford the desired product.

COMPOUND CHARACTERISATION

1-(4-Chlorophenyl)ethan-1-one oxime (**1b**)[S1]

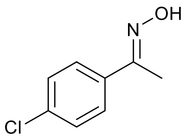

Following **general procedure 1**: A magnetically stirred mixture of 4-chloroacetophenone (0.65 mL, 5 mmol), $NH_2OH{\cdot}HCl$ (0.520 g, 7.5 mmol) and sodium acetate (1.025 g, 12.5 mmol) in ethanol/water (20 mL of a 1:3 v/v mixture) was heated under reflux for 2 h. The precipitate was formed upon cooling and the reaction mixture was isolated by filtration, washed by water (3 × 10 mL) to give the desired ketoxime as a white solid. [839 mg, (99) 99%].

M.P. 97-98 °C (lit. 97.8-97.9 °C)[S1]

^{1}H NMR (400 MHz, DMSO-d_6) δ_H 11.34 (s, 1H), 7.68 – 7.64 (m, 2H), 7.45 – 7.42 (m, 2H), 2.14 (s, 3H).

^{13}C NMR (101 MHz, DMSO-d_6) δ_C 152.5, 136.2, 133.7, 128.8, 127.7, 11.8.

LRMS m/z (ESI-) 168.03 ([M^{35}Cl-H]$^-$, 100%), 170.03 ([M^{37}Cl-H]$^-$, 50%)

HRMS m/z (ESI-) C_8H_7ClNO requires 168.0222, found 168.0301 ([M^{35}Cl -H]$^-$)

1-(4-Fluorophenyl)ethan-1-one oxime (**1c**)[S1]

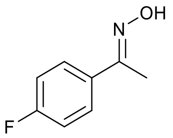

Following **general procedure 1**: A magnetically stirred mixture of 4-fluoroacetophenone (0.60 mL, 5 mmol), $NH_2OH{\cdot}HCl$ (0.520 g, 7.5 mmol) and sodium acetate (1.025 g, 12.5 mmol) in ethanol/water (20 mL of a 1:3 v/v mixture) was heated under reflux for 2 h. The precipitate was formed upon cooling and the reaction mixture was isolated by filtration, washed by water (3 × 10 mL) to give the desired ketoxime as a white solid. [728 mg, (99) 95%].

M.P. 59-60 °C (lit. 58.2-60.1 °C)[S1]

^{1}H NMR (400 MHz, DMSO-d_6) δ_H 11.35 (s, 1H), 7.70 – 7.66 (m, 2H), 7.23 – 7.18 (m, 2H), 2.13 (s, 3H).

^{13}**C NMR** (101 MHz, DMSO-d$_6$) δ_C 163.3 (d, $^1J_{C\text{-}F}$ = 244.5 Hz), 161.6, 150.8 (d, $^4J_{C\text{-}F}$ = 3.1 Hz) , 133.4 (d, $^3J_{C\text{-}F}$ = 7.7 Hz), 124.2 (d, $^2J_{C\text{-}F}$ = 22.0 Hz), 12.0.

^{19}**F NMR** (377 MHz, MeOD) δ -112.18

LRMS m/z (ESI-) 152.06 ([M-H]$^-$, 100%)

HRMS m/z (ESI-) C_8H_7FNO requires 152.0517, found 152.0622 ([M-H]$^-$)

1-(4-Tolyl)ethan-1-one oxime (**1d**)[S2]

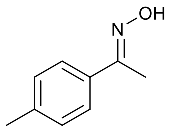

Following **general procedure 1**: A magnetically stirred mixture of 4-methylacetophenone (0.65 mL, 5 mmol), $NH_2OH\cdot HCl$ (0.520 g, 7.5 mmol) and sodium acetate (1.025 g, 12.5 mmol) in ethanol/water (20 mL of a 1:3 v/v mixture) was heated under reflux for 2 h. The precipitate was formed upon cooling and the reaction mixture was isolated by filtration, washed by water (3 × 10 mL) to give the desired ketoxime as a white solid. [739 mg, (99) 98%].

M.P. 82-85 °C (lit. 85-86 °C)[S2]

^{1}**H NMR** (400 MHz, DMSO-d$_6$) δ_H 11.08 (s, 1H), 7.60 – 7.47 (m, 2H), 7.26 – 7.13 (m, 2H), 2.30 (s, 3H), 2.12 (s, 3H).

^{13}**C NMR** (101 MHz, DMSO-d$_6$) δ_C 153.2, 138.5, 134.7, 129.4, 125.9, 21.2, 12.0

LRMS m/z (ESI-) 148.08 ([M-H]$^-$, 100%)

HRMS m/z (ESI-) $C_9H_{10}NO$ requires 148.0768, found 148.0844 ([M-H]$^-$)

1-(4-Methoxyphenyl)ethan-1-one oxime (**1e**)[S1]

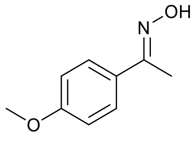

Following **general procedure 1**: A magnetically stirred mixture of 4-methoxylacetophenone (0.65 mL, 5 mmol), $NH_2OH\cdot HCl$ (0.520 g, 7.5 mmol) and sodium acetate (1.025 g, 12.5 mmol) in ethanol/water (20 mL of a 1:3 v/v mixture) was heated under reflux for 2 h. The precipitate was formed upon cooling and the reaction mixture was isolated by filtration, washed by water (3 × 10 mL) to give the desired ketoxime as a white solid. [784 mg, (99) 95%].

M.P. 92-93 °C (lit. 90.7-91.5 °C)[S1]

^{1}H NMR (400 MHz, DMSO-d_6) δ_H 10.96 (s, 1H), 7.60 – 7.56 (m, 2H), 6.95 – 6.91 (m, 2H), 3.77 (s, 3H), 2.11 (s, 3H).

^{13}C NMR (101 MHz, DMSO-d_6) δ_C 160.1, 152.8, 129.9, 127.3, 114.2, 55.6, 11.9.

LRMS m/z (ESI-) 164.08 ([M-H]$^-$, 100%)

HRMS m/z (ESI-) $C_9H_{10}NO_2$ requires 164.0712, found 164.0829 ([M-H]$^-$)

1-(4-Nitrophenyl)ethan-1-one oxime (**1f**)[S1]

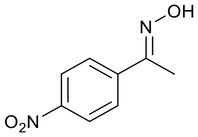

Following **general procedure 1**: A magnetically stirred mixture of 4-nitroacetophenone (825.8 mg, 5 mmol), $NH_2OH·HCl$ (0.520 g, 7.5 mmol) and sodium acetate (1.025 g, 12.5 mmol) in ethanol/water (20 mL of a 1:3 v/v mixture) was heated under reflux for 2 h. The precipitate was formed upon cooling and the reaction mixture was isolated by filtration, washed by water (3 × 10 mL) to give the desired ketoxime as a white solid. [856 mg, (99) 95%].

M.P. 168-170 °C (lit. 170.5-172.5 °C)[S1]

^{1}H NMR (400 MHz, DMSO-d_6) δ_H 11.84 (s, 1H), 8.21 (d, *J* = 8.9 Hz, 2H), 7.90 (d, *J* = 8.9 Hz, 2H), 2.20 (s, 3H).

^{13}C NMR (101 MHz, DMSO-d_6) δ_C 152.9, 147.7, 143.6, 128.8, 124.8, 13.0.

LRMS m/z (ESI-) 179.05 ([M-H]$^-$, 100%)

HRMS m/z (ESI-) $C_8H_7N_2O_3$ requires 179.0457, found 179.0493 ([M-H]$^-$)

1-(4-Bromo-3-nitrophenyl)ethan-1-one oxime (**1g**)

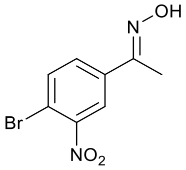

Following **general procedure 1**: A magnetically stirred mixture of 4-bromo-3-nitroacetophenone (1101 mg, 5 mmol), $NH_2OH·HCl$ (0.520 g, 7.5 mmol) and sodium acetate

(1.025 g, 12.5 mmol) in ethanol/water (20 mL of a 1:3 v/v mixture) was heated under reflux for 2 h. The precipitate was formed upon cooling and the reaction mixture was isolated by filtration, washed by water (3 × 10 mL) to give the desired ketoxime as a yellow solid. [1166 mg, (99) 90%].

M.P. 156-158 °C

^{1}H NMR (400 MHz, DMSO-d$_6$) δ_H 11.82 (s, 1H), 8.20 (d, *J* = 2.1 Hz, 1H), 7.91 (d, *J* = 8.4 Hz, 1H), 7.85 (dd, *J* = 2.1 Hz, *J* = 8.4 Hz, 1H), 2.17 (s, 3H).

^{13}C NMR (101 MHz, DMSO-d$_6$) δ_C 151.3, 150.3, 138.3, 135.2, 130.7, 122.5, 113.2, 11.6.

LRMS m/z (ESI-) 256.96 ([M-H]$^-$, 100%)

HRMS m/z (ESI-) $C_8H_5BrN_2O_3$ requires 256.9611, found 256.9610 ([M-H]$^-$)

1-(4-(methylthio)phenyl)ethan-1-one oxime (**1h**)

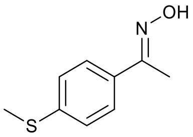

Following **general procedure 1**: A magnetically stirred mixture of 4-methylthioacetophenone (831.2 mg, 5 mmol), $NH_2OH \cdot HCl$ (0.520 g, 7.5 mmol) and sodium acetate (1.025 g, 12.5 mmol) in ethanol/water (20 mL of a 1:3 v/v mixture) was heated under reflux for 2 h. The precipitate was formed upon cooling and the reaction mixture was isolated by filtration, washed by water (3 × 10 mL) to give the desired ketoxime as a white solid. [888.1 mg, (99) 98%].

M.P. 120-122 °C

^{1}H NMR (400 MHz, DMSO-d$_6$) δ_H 11.19 (1 H, s), 7.66 – 7.55 (2 H, m), 7.35 – 7.18 (2 H, m), 2.14 (3 H, s).

^{13}C NMR (101 MHz, DMSO-d$_6$) δ_C 152.8, 139.3, 133.9, 126.4, 126.0, 14.9, 11.8.

LRMS m/z (ESI-) 180.06 ([M-H]$^-$, 100%)

HRMS m/z (ESI-) $C_9H_{10}NOS$ requires 180.0599, found 180.0597 ([M-H]$^-$)

1-(3,4,5-Trimethoxyphenyl)ethan-1-one oxime (**1i**)[S3]

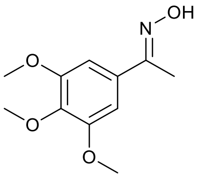

Following **general procedure 1**: A magnetically stirred mixture of 3,4,5-trimethoxylacetophenone (1052 mg, 5 mmol), $NH_2OH{\cdot}HCl$ (0.520 g, 7.5 mmol) and sodium acetate (1.025 g, 12.5 mmol) in ethanol/water (20 mL of a 1:3 v/v mixture) was heated under reflux for 2 h. The precipitate was formed upon cooling and the reaction mixture was isolated by filtration, washed by water (3 × 10 mL) to give the desired ketoxime as a white solid. [1081 mg, (99) 96%].

M.P. 104-105 °C (lit. 102 °C)[S3]

^{1}H NMR (400 MHz, DMSO-d_6) δ_H 11.15 (1 H, s), 6.92 (2 H, s), 3.80 (6 H, s), 3.67 (3 H, s), 2.14 (3 H, s).

^{13}C NMR (101 MHz, DMSO-d_6) δ_C 153.2, 138.6, 133.1, 103.5, 60.5, 56.3, 12.1.

LRMS m/z (ESI-) 224.10 ([M-H]$^-$, 100%)

HRMS m/z (ESI-) $C_{11}H_{14}NO_4$ requires 224.1001, found 224.1005 ([M-H]$^-$)

1-(3-Fluoro-4-methoxylphenyl)ethan-1-one oxime (**1j**)

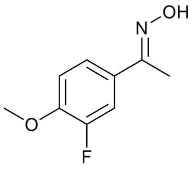

Following **general procedure 1**: A magnetically stirred mixture of 3-fluoro-4-methoxylacetophenone (840.9 mg, 5 mmol), $NH_2OH{\cdot}HCl$ (0.520 g, 7.5 mmol) and sodium acetate (1.025 g, 12.5 mmol) in ethanol/water (20 mL of a 1:3 v/v mixture) was heated under reflux for 2 h. The precipitate was formed upon cooling and the reaction mixture was isolated by filtration, washed by water (3 × 10 mL) to give the desired ketoxime as a white solid. [824 mg, (99) 98%].

M.P. 150-151 °C

^{1}H NMR (400 MHz, DMSO-d_6) δ_H 11.18 (s, 1H), 7.50 – 7.38 (m, 2H), 7.16 (t, J = 8.8 Hz, 1H), 3.85 (s, 3H), 2.11 (s, 3H).

^{13}C NMR (101 MHz, DMSO-d$_6$) δ_C 152.9 (d, J_{C-F} = 243.3 Hz), 152.1 (d, J_{C-F} = 2.6 Hz), 148.0 (d, J_{C-F} = 10.9 Hz), 130.5 (d, J_{C-F} = 6.6 Hz), 122.5 (d, J_{C-F} = 3.3 Hz), 114.0 (d, J_{C-F} = 2.2 Hz), 113.2 (d, J_{C-F} = 19.5 Hz), 56.5, 11.8.

^{19}F NMR (377 MHz, DMSO-d$_6$) δ -135.39

LRMS m/z (ESI-) 182.07 ([M-H]$^-$, 100%)

HRMS m/z (ESI-) $C_9H_9FNO_2$ requires 182.0754, found 182.0750 ([M-H]$^-$)

1-(4-Chloro-3-nitrophenyl)ethan-1-one oxime (**1k**)

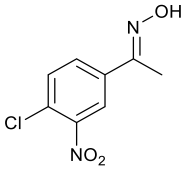

Following **general procedure 1**: A magnetically stirred mixture of 4-chloro-3-nitroacetophenone (998 mg, 5 mmol), $NH_2OH{\cdot}HCl$ (0.520 g, 7.5 mmol) and sodium acetate (1.025 g, 12.5 mmol) in ethanol/water (20 mL of a 1:3 v/v mixture) was heated under reflux for 2 h. The precipitate was formed upon cooling and the reaction mixture was isolated by filtration, washed by water (3 × 10 mL) to give the desired ketoxime as a yellow solid. [1060 mg, (99) 98%].

M.P. 160-162 °C

^{1}H NMR (400 MHz, DMSO-d$_6$) δ_H 11.80 (s, 1H), 8.25 (d, *J* = 2.2 Hz, 1H), 7.95 (dd, *J* = 8.5 Hz, *J* = 2.2 Hz, 1H), 7.78 (d, *J* = 8.5 Hz, 1H), 2.17 (s, 3H).

^{13}C NMR (101 MHz, DMSO-d$_6$) δ_C 151.3, 148.2, 137.8, 132.1, 130.8, 125.1, 122.6, 11.7.

LRMS m/z (ESI-) 213.01 ([M^{35}Cl-H]$^-$, 100%), 215.01 ([M^{37}Cl-H]$^-$, 50%)

HRMS m/z (ESI-) $C_8H_6ClN_2O_3$ requires 213.0139, found 213.0144 ([M^{35}Cl -H]$^-$)

1-(4-(Trifluoromethyl)phenyl)ethan-1-one oxime (**1l**)[S1]

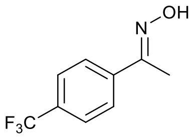

Following **general procedure 1**: A magnetically stirred mixture of 4-trifluoromethylacetophenone (940.8 mg, 5 mmol), $NH_2OH{\cdot}HCl$ (0.520 g, 7.5 mmol) and sodium acetate (1.025 g, 12.5 mmol) in ethanol/water (20 mL of a 1:3 v/v mixture) was heated under reflux for 2 h. The precipitate was formed upon cooling and the reaction mixture was isolated by filtration, washed by water (3 × 10 mL) to give the desired ketoxime as a white solid. [965 mg, (99) 95%].

M.P. 104-106 °C (lit. 104.6-104.7 °C)[S1]

^{1}H NMR (400 MHz, DMSO-d$_6$) δ_H 11.55 (s, 1H), 7.93 – 7.89 (m, 2H), 7.84 – 7.72 (m, 2H), 2.19 (s, 3H).

^{13}C NMR (101 MHz, DMSO-d$_6$) δ_C 152.5, 141.3, 129.4 (d, $^2J_{C\text{-}F}$ = 32 Hz), 126.7 (q, $^4J_{C\text{-}F}$ = 3.8 Hz), 125.7 (q, $^3J_{C\text{-}F}$ = 11.5 Hz), 123.4 (d, $^1J_{C\text{-}F}$ = 271.6 Hz), 11.9.

^{19}F NMR (377 MHz, DMSO-d$_6$) δ -61.10

LRMS m/z (ESI-) 202.06 ([M-H]$^-$, 100%)

HRMS m/z (ESI-) $C_9H_7F_3NO$ requires 202.0596, found 202.0594([M-H]$^-$)

1-(3-Chloro-4-methoxylphenyl)ethan-1-one oxime (**1m**)

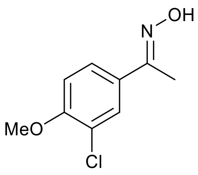

Following **general procedure 1**: A magnetically stirred mixture of 3-chloro-4-methoxylacetophenone (825.1 mg, 5 mmol), $NH_2OH{\cdot}HCl$ (0.520 g, 7.5 mmol) and sodium acetate (1.025 g, 12.5 mmol) in ethanol/water (20 mL of a 1:3 v/v mixture) was heated under reflux for 2 h. The precipitate was formed upon cooling and the reaction mixture was isolated by filtration, washed by water (3 × 10 mL) to give the desired ketoxime as a solid. [789 mg, (99) 95%].

M.P. 160-162 °C

^{1}**H NMR** (400 MHz, DMSO-d$_6$) δ_H δ 11.26 (s, 1H), 7.67 (d, *J* = 2.2 Hz, 1H), 7.58 (dd, *J* = 8.7, 2.2 Hz, 1H), 7.15 (d, *J* = 8.7 Hz, 1H), 3.87 (s, 3H), 2.11 (s, 3H).

^{13}**C NMR** (101 MHz, DMSO-d$_6$) δ_C 155.2, 151.9, 130.9, 127.1, 126.1, 121.5, 113.0, 56.7, 11.8.

LRMS m/z (ESI-) 198.04 ([M^{35}Cl-H]$^-$, 100%), 200.04 ([M^{37}Cl-H]$^-$, 50%)

HRMS m/z (ESI-) $C_9H_6ClNO_2$ requires 198.0441, found 198.0443 ([M^{35}Cl-H]$^-$)

1-(Benzo[*d*][1,3]dioxol-5-yl)ethan-1-one oxime (**1n**)

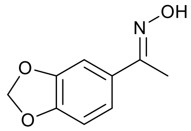

Following **general procedure 1**: A magnetically stirred mixture of 3,4-(methylenedioxy)acetophenone (783.2 mg, 5 mmol), $NH_2OH{\cdot}HCl$ (0.520 g, 7.5 mmol) and sodium acetate (1.025 g, 12.5 mmol) in ethanol/water (20 mL of a 1:3 v/v mixture) was heated under reflux for 2 h. The precipitate was formed upon cooling and the reaction mixture was isolated by filtration, washed by water (3 × 10 mL) to give the desired ketoxime as a white solid. [965.0 mg, (99) 90%].

M.P. 126-128 °C

^{1}**H NMR** (400 MHz, DMSO-d$_6$) δ_H 11.07 (s, 1H), 7.20 (d, *J* = 1.8 Hz, 1H), 7.12 (dd, *J* = 8.2, 1.8 Hz, 1H), 6.91 (d, *J* = 8.2 Hz, 1H), 6.03 (s, 2H), 2.10 (s, 3H).

^{13}**C NMR** (101 MHz, DMSO-d$_6$) δ_C 152.9, 148.1, 148.0, 131.7, 120.2, 108.4, 105.8, 101.6, 12.1

LRMS m/z (ESI-) 178.06 ([M-H]$^-$, 100%)

HRMS m/z (ESI-) $C_9H_8NO_3$ requires 178.0622, found 178.0620 ([M-H]$^-$)

Tributylammonium (1-phenylethylidene)amino sulfate (**2a**)

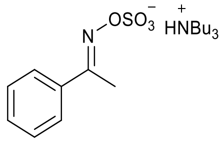

Following **general procedure 2**: 1-phenylethan-1-one oxime (**1a**) (135mg, 1.0 mmol) and TBSAB (531 mg, 2.0 mmol) were dissolved in anhydrous MeCN (2.0 mL) and heated under

reflux for 3 h. The flask was cooled, and the solvent removed *in vacuo*. The flask was charged with H_2O (20 mL) and the aqueous mixture extracted with EtOAc (4 × 30 mL). The organic extracts were pooled, dried ($MgSO_4$), filtered and the filtrate solvent was removed *in vacuo* to afford the desired product as a clear oil as a clear oil (398 mg, 99%).

^{1}H NMR (400 MHz, DMSO-d$_6$) δ_H 8.95 (s, 1H), 7.74 – 7.62 (m, 2H), 7.49 – 7.36 (m, 3H), 3.05 – 2.99 (m, 6H), 2.18 (s, 3H), 1.61 – 1.54 (m, 6H), 1.30 (q, *J* = 7.4 Hz, 7H), 0.90 (t, *J* = 7.4 Hz, 9H)

^{13}C NMR (101 MHz, DMSO-d$_6$) δ_C 156.6, 136.7, 130.7, 128.3, 124.6, 52.4, 25.4, 19.3, 14.0, 12.0.

LRMS m/z (ESI-) 214.02 ([M-Bu_3NH]$^-$, 100%)

HRMS m/z (ESI-) $C_8H_8NO_4S$ requires 214.0222, found 214.0219 ([M - Bu_3NH]$^-$)

Sodium (1-phenylethylidene)amino sulfate (**2a[Na]**)

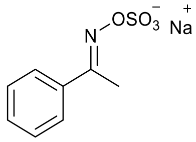

1-Phenylethan-1-one oxime **2a** (135 mg, 1.0 mmol) and TBSAB (531.0 mg, 2.0 mmol) were dissolved in dry MeCN (2.0 mL) and heated under reflux for 3 h. The reaction was cooled, and Sodium iodide (750 mg, 5 mmol) was added, followed by MeCN (25 mL). The reaction was stirred for an hour and then washed with MeCN (3 x 10 mL), filtered to give the desired sodium sulfate (194.4 mg, 82%).

M.P. >350 °C (dec.)

^{1}H NMR (400 MHz, D_2O) δ_H 7.68 – 7.63 (m, 2H), 7.47 – 7.38 (m, 3H), 2.29 (s, 3H).

^{13}C NMR (101 MHz, D_2O) δ_C 164.6, 134.5, 130.8, 128.8, 126.9, 12.7.

LRMS m/z (ESI+) 260.00 ([M+Na]$^+$, 100%)

HRMS m/z (ESI+) $C_8H_8NO_4Na_2S$ requires 260.0005, found 260.0003([M+Na]$^+$)

Potassium (1-phenylethylidene)amino sulfate (**2a[K]**)

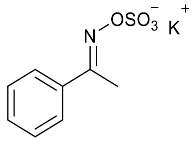

1-Phenylethan-1-one oxime **2a** (135 mg, 1.0 mmol) and TBSAB (531.0 mg, 2.0 mmol) were dissolved in dry MeCN (2.0 mL) and heated under reflux for 3 h. The reaction was cooled and potassium iodide (864 mg, 5 mmol) was added, followed by MeCN (25 mL). The reaction was stirred for an hour and then washed with MeCN (3 x 10 mL), filtered to give the desired sodium sulfate (228 mg, 90%).

M.P. 225 - 230 °C

^{1}H NMR (400 MHz, D_2O) δ_H 7.68 – 7.61 (m, 2H), 7.48 – 7.39 (m, 3H), 2.28 (s, 3H).

^{13}C NMR (101 MHz, D_2O) δ_C 166.7, 134.4, 130.8, 128.8, 126.9, 13.9.

LRMS m/z (ESI+) 291.94 ($[M+K]^+$, 100%)

HRMS m/z (ESI+) $C_8H_8NO_4K_2S$ requires 291.9398, found 291.9399 ($[M+K]^+$)

Tributylammonium (1-(4-chlorophenyl)ethylidene)amino sulfate (**2b**)

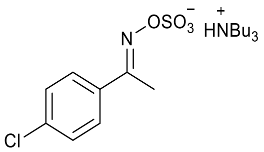

Following **general procedure 2**: 1-(4-chlorophenyl)ethan-1-one oxime (**1b**) (170 mg, 1.0 mmol) and TBSAB (531 mg, 2.0 mmol) were dissolved in anhydrous MeCN (2.0 mL) and heated under reflux for 3 h. The flask was cooled, and the solvent removed *in vacuo*. The flask was charged with H_2O (20 mL) and the aqueous mixture extracted with EtOAc (4 × 30 mL). The organic extracts were pooled, dried ($MgSO_4$), filtered and the filtrate solvent was removed *in vacuo* to afford the desired product as a clear oil as a clear oil (400 mg, 92%).

^{1}H NMR (400 MHz, DMSO-d_6) δ_H 8.94 (s, 1H), 7.71 (d, *J* = 7.2 Hz, 2H), 7.50 (d, *J* = 7.2 Hz, 2H), 3.03 – 3.00 (m, 6H), 2.17 (s, 3H), 1.60 – 1.56 (m, 6H), 1.32 (h, *J* = 7.4 Hz, 6H), 0.91 (t, *J* = 7.4 Hz, 9H).

^{13}C NMR (101 MHz, DMSO-d_6) δ_C 154.3, 135.3, 134.1, 128.9, 128.5, 50.9, 25.1, 19.8, 14.5, 11.3.

LRMS m/z (ESI-) 247.98 ($[M^{35}Cl - Bu_3NH]^-$, 100%), 249.98 ($[M^{37}Cl - Bu_3NH]^-$, 40%)

HRMS m/z (ESI-) $C_8H_7ClNO_4S$ requires 247.9802, found 247.9799 ($[M^{35}Cl - Bu_3NH]^-$)

Tributylammonium (1-(4-fluorophenyl)ethylidene)amino sulfate (**2c**)

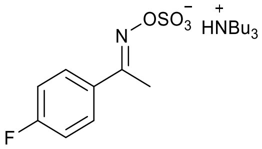

Following **general procedure 2**: 1-(4-fluorophenyl)ethan-1-one oxime (**1c**) (153 mg, 1.0 mmol) and TBSAB (531 mg, 2.0 mmol) were dissolved in anhydrous MeCN (2.0 mL) and heated under reflux for 3 h. The flask was cooled, and the solvent removed *in vacuo*. The flask was charged with H_2O (20 mL) and the aqueous mixture extracted with EtOAc (4 × 30 mL). The organic extracts were pooled, dried ($MgSO_4$), filtered and the filtrate solvent was removed *in vacuo* to afford the desired product as a clear oil as a clear oil (376 mg, 90%).

^{1}H NMR (400 MHz, DMSO-d$_6$) δ_H 9.01 (s, 1H), 7.74 (d, *J* = 7.3 Hz, 2H), 7.26 (d, *J* = 7.3 Hz, 2H), 3.03-3.00 (m, 6H), 2.17 (s, 3H), 1.60 – 1.56 (m, 6H), 1.31 (h, *J* = 7.4 Hz, 6H), 0.91 (t, *J* = 7.4 Hz, 9H).

^{13}C NMR (101 MHz, DMSO-d$_6$) δ_C 161.8 (d, $^1J_{C\text{-}F}$ = 242.4 Hz), 153.2, 130.3 (d, $^4J_{C\text{-}F}$ = 3.0 Hz), 126.4 (d, $^3J_{C\text{-}F}$ = 8.5 Hz), 113.5 (d, $^2J_{C\text{-}F}$ = 21.6 Hz), 50.1, 25.6, 17.1, 11.9, 11.2.

^{19}F NMR (377 MHz, MeOD) δ -112.1

LRMS m/z (ESI-) 232.01 ([M-Bu_3NH]$^-$, 100%),

HRMS m/z (ESI-) $C_8H_7FNO_4S$ requires 232.0137, found 232.0134 ([M-Bu_3NH]$^-$)

Tributylammonium (1-(4-methylphenyl)ethylidene)amino sulfate (**2d**)

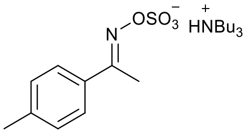

Following **general procedure 2**: 1-(4-methylphenyl)ethan-1-one oxime (**1d**) (149 mg, 1.0 mmol) and TBSAB (531 mg, 2.0 mmol) were dissolved in anhydrous MeCN (2.0 mL) and heated under reflux for 3 h. The flask was cooled, and the solvent removed *in vacuo*. The flask was charged with H_2O (20 mL) and the aqueous mixture extracted with EtOAc (4 × 30 mL). The organic extracts were pooled, dried ($MgSO_4$), filtered and the filtrate solvent was removed *in vacuo* to afford the desired product as a clear oil as a clear oil (393 mg, 95%).

^{1}H NMR (400 MHz, DMSO-d$_6$) δ_H 8.94 (s, 1H), 7.58 (d, *J* = 8.0 Hz, 2H), 7.23 (d, *J* = 8.0 Hz, 2H), 3.09 – 2.88 (m, 6H), 2.33 (s, 3H), 2.15 (s, 3H), 1.61 – 1.59 (m, 6H), 1.31 (h, *J* = 7.4 Hz, 6H), 0.90 (t, *J* = 7.4 Hz, 9H).

^{13}C NMR (101 MHz, DMSO-d$_6$) δ_C 156.1, 138.1, 131.9, 128.3, 124.5, 51.4, 25.0, 19.6, 16.4, 11.9, 10.7.

LRMS m/z (ESI-) 228.03 ([M-Bu$_3$NH]$^-$, 100%),

HRMS m/z (ESI-) $C_9H_{10}NO_4S$ requires 228.0341, found 228.0338 ([M-Bu$_3$NH]$^-$)

Tributylammonium (1-(4-methoxyphenyl)ethylidene)amino sulfate (**2e**)

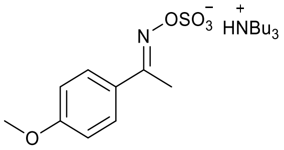

Following **general procedure 2**: 1-(4-methoxyphenyl)ethan-1-one oxime (**1e**) (149 mg, 1.0 mmol) and TBSAB (531 mg, 2.0 mmol) were dissolved in anhydrous MeCN (2.0 mL) and heated under reflux for 3 h. The flask was cooled, and the solvent removed *in vacuo*. The flask was charged with H_2O (20 mL) and the aqueous mixture extracted with EtOAc (4 × 30 mL). The organic extracts were pooled, dried ($MgSO_4$), filtered and the filtrate solvent was removed *in vacuo* to afford the desired product as a clear oil as a clear oil (402 mg, 98%).

^{1}H NMR (400 MHz, DMSO-d$_6$) δ_H 8.85 (s, 1H), 7.64 (d, *J* = 8.7 Hz, 2H), 6.98 (d, *J* = 8.7 Hz, 2H), 3.79 (s, 3H), 3.06 – 3.00 (m, 6H), 2.14 (s, 3H), 1.59 – 1.55 (m, 6H), 1.31 (h, *J* = 7.4 Hz, 6H), 0.91 (t, *J* = 7.4 Hz, 9H).

^{13}C NMR (101 MHz, DMSO-d$_6$) δ_C 160.7, 156.4, 128.7, 128.1, 114.2, 55.7, 52.3, 25.5, 19.1, 14.0, 12.8.

LRMS m/z (ESI-) 244.03 ([M-Bu$_3$NH]$^-$, 100%),

HRMS m/z (ESI-) $C_9H_{10}NO_5S$ requires 244.0319, found 244.0322 ([M-Bu$_3$NH]$^-$)

Tributylammonium (1-(4-nitrophenyl)ethylidene)amino sulfate (**2f**)

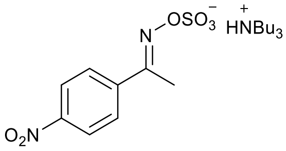

Following **general procedure 2**: 1-(4-nitrophenyl)ethan-1-one oxime (**1f**) (180 mg, 1.0 mmol) and TBSAB (531 mg, 2.0 mmol) were dissolved in anhydrous MeCN (2.0 mL) and heated under reflux for 3 h. The flask was cooled, and the solvent removed *in vacuo*. The flask was charged with H_2O (20 mL) and the aqueous mixture extracted with EtOAc (4 × 30 mL). The

organic extracts were pooled, dried ($MgSO_4$), filtered and the filtrate solvent was removed *in vacuo* to afford the desired product as a clear oil as a clear oil (419 mg, 94%).

^{1}H NMR (400 MHz, DMSO-d$_6$) δ_H 8.85 (s, 1H), 8.28 (d, *J* = 9.2 Hz, 2H), 7.97 (d, *J* = 9.2 Hz, 2H), 3.03 – 3.00 (m, 6H), 2.23 (s, 3H), 1.62 – 1.52 (m, 6H), 1.31 (h, *J* = 7.4 Hz, 6H), 0.91 (t, *J* = 7.4 Hz, 9H).

^{13}C NMR (101 MHz, DMSO-d$_6$) δ_C 153.0, 150.0, 142.1, 128.4, 124.1, 52.9, 25.5, 20.8, 14.0, 12.6.

LRMS m/z (ESI-) 259.00 ([M-$Bu_3NH]^-$, 100%),

HRMS m/z (ESI-) $C_8H_7N_2O_6S$ requires 259.0025, found 259.0030 ([M-$Bu_3NH]^-$

Tributylammonium (1-(4-bromo-3-nitrophenyl)ethylidene)amino sulfate (**2g**)

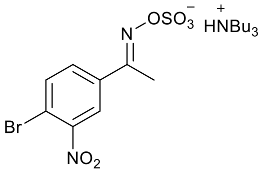

Following **general procedure 2**: 1-(4-bromo-3-nitrophenyl) ethan-1-one oxime (259 mg, 1.0 mmol) and TBSAB (531 mg, 2.0 mmol) were dissolved in anhydrous Dioxane (2.0 mL) and heated under reflux for 3 h. The flask was cooled, and the solvent removed *in vacuo*. The crude product was purified with (SiO_2; Hexane/EtoAc, 1:1, R_f = 0.2) to afford the desired product as a clear oil as a clear oil (393 mg, 75%).

^{1}H NMR (400 MHz, DMSO-d$_6$) δ_H 9.03 (s, 1H), 8.25 (d, *J* = 2.1 Hz, 1H), 7.99 (d, *J* = 8.5 Hz, 1H), 7.90 (dd, *J* = 8.5, 2.1 Hz, 1H), 3.06 – 2.84 (m, 6H), 2.20 (s, 3H), 1.60-1.55 (m, 6H), 1.32 (h, *J* = 7.4 Hz, 6H), 0.91 (t, *J* = 7.4 Hz, 9H).

^{13}C NMR (101 MHz, DMSO-d$_6$) δ_C 153.9, 137.3, 135.4, 131.5, 123.2, 118.2, 114.3, 52.2, 25.5, 19.8, 14.0, 12.9.

LRMS m/z (ESI-) 338.91 ([M-$Bu_3NH]^-$, 100%)

HRMS m/z (ESI-) $C_8H_6BrN_2O_6$ requires 338.9097, found 338.9101 ([M-$Bu_3NH]^-$)

Tributylammonium (1-(4-(methylthio)phenyl)ethylidene)amino sulfate (**2h**)

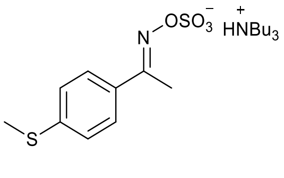

Following **general procedure 2**: 1-(4-(methylthio)phenyl)ethan-1-one oxime (**1h**) (181 mg, 1.0 mmol) and TBSAB (531 mg, 2.0 mmol) were dissolved in anhydrous MeCN (2.0 mL) and heated under reflux for 3 h. The flask was cooled, and the solvent removed *in vacuo*. The flask was charged with H_2O (20 mL) and the aqueous mixture extracted with EtOAc (4 × 30 mL). The organic extracts were pooled, dried ($MgSO_4$), filtered and the filtrate solvent was removed *in vacuo* to afford the desired product as a clear oil as a clear oil (437 mg, 98%).

^{1}H NMR (400 MHz, DMSO-d_6) δ_H 9.00 (s, 1H), 7.74-7.69 (m, 2H), 7.35-7.25 (m, 2H), 3.06 – 2.98 (m, 6H), 2.50 (s, 3H), 2.17 (s, 3H), 1.62 – 1.56 (m, 6H), 1.31 (h, *J* = 7.4 Hz, 6H), 0.91 (t, *J* = 7.4 Hz, 9H).

^{13}C NMR (101 MHz, DMSO-d_6) δ_C 156.3, 141.6, 133.4, 127.1, 125.3, 54.2, 25.9, 19.8, 15.5, 14.0, 11.9.

LRMS m/z (ESI-) 260.01 ($[M\text{-}Bu_3NH]^-$, 100%),

HRMS m/z (ESI-) $C_9H_{10}NO_4S_2$ requires 260.0057, found 260.0113 ($[M\text{-}Bu_3NH]^-$)

Tributylammonium (1-(3,4,5-trimethoxyphenyl)ethylidene)amino sulfate (**2i**)

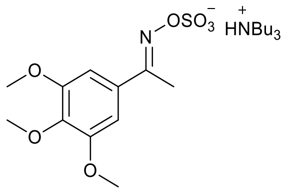

Following **general procedure 2**: 1-(3,4,5-trimethoxyphenyl)ethan-1-one oxime (**1i**) (225 mg, 1.0 mmol) and TBSAB (531 mg, 2.0 mmol) were dissolved in anhydrous MeCN (2.0 mL) and heated under reflux for 3 h. The flask was cooled, and the solvent removed *in vacuo*. The flask was charged with H_2O (20 mL) and the aqueous mixture extracted with EtOAc (4 × 30 mL). The organic extracts were pooled, dried ($MgSO_4$), filtered and the filtrate solvent was removed *in vacuo* to afford the desired product as a clear oil as a clear oil (480 mg, 92%).

^{1}H NMR (400 MHz, DMSO-d_6) δ_H 8.90 (s, 1H), 6.94 (s, 2H), 3.82 (s, 6H), 3.69 (s, 3H), 3.06 – 2.98 (m, 6H), 2.17 (s, 3H), 1.62 – 1.56 (m, 6H), 1.31 (h, *J* = 7.4 Hz, 6H), 0.91 (t, *J* = 7.4 Hz, 9H)

^{13}C NMR (101 MHz, DMSO-d_6) δ_C 159.1, 154.0, 141.0, 132.9, 103.3, 60.6, 57.1, 52.9, 26.4, 20.6, 14.0, 13.7.

LRMS m/z (ESI-) 304.05 ($[M\text{-}Bu_3NH]^-$, 100%)

HRMS m/z (ESI-) $C_{11}H_{14}NO_7S$ requires 304.0543, found 304.0539 ($[M-Bu_3NH]^-$)

Tributylammonium (*E*)-(1-(3-fluoro-4-methoxyphenyl)ethylidene)amino sulfate (**2j**)

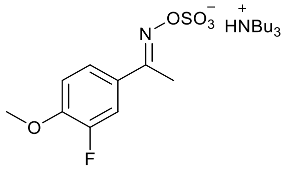

Following **General procedure 2**: 1-(3-fluoro-4-methoxyphenyl) ethan-1-one oxime (183 mg, 1.0 mmol) and TBSAB (531.0 mg, 2.0 mmol) were dissolved in dry MeCN (2.0 mL) and heated under reflux for 3 h. The flask was cooled, and the solvent removed *in vacuo*. The flask was charged with H_2O (20 mL) and the aqueous mixture extracted with EtOAc (4 × 30 mL). The organic extracts were pooled, dried ($MgSO_4$), filtered and the filtrate solvent was removed *in vacuo* to afford the desired product as a clear oil (444 mg, 99%)

^{1}H NMR (400 MHz, DMSO-d$_6$) δ_H 8.98 (s, 1H), 7.52 (d, *J* = 2.1 Hz, 1H), 7.48 (dd, *J* = 8.5, 2.1 Hz, 1H), 7.21 (d, *J* = 8.5 Hz, 1H), 3.88 (s, 3H), 3.04 – 2.92 (m, 6H), 2.13 (s, 3H), 1.57 (m, 6H), 1.31 (h, *J* = 7.4 Hz, 6H), 0.91 (t, *J* = 7.4 Hz, 9H).

^{13}C NMR (101 MHz, DMSO-d$_6$) δ_C 154.6 (d, $J_{C\text{-}F}$ = 256.4 Hz), 152.5 (d, $J_{C\text{-}F}$ = 2.5 Hz), 150.2 (d, $J_{C\text{-}F}$ = 11.2 Hz), 148.2 (d, $J_{C\text{-}F}$ = 7.2 Hz), 127.2 (d, $J_{C\text{-}F}$ = 3.4 Hz), 122.1 (d, $J_{C\text{-}F}$ = 1.8 Hz), 115.6 (d, $J_{C\text{-}F}$ = 12.3 Hz), 56.1, 51.8, 25.1, 19.4, 13.5, 12.6

^{19}F NMR (377 MHz, DMSO-d$_6$) δ_F -135.47

LRMS m/z (ESI-) 262.02 ($[M-Bu_3NH]^-$, 100%)

HRMS m/z (ESI-) $C_9H_9FNO_5S$ requires 262.0195, found 262.0200 ($[M-Bu_3NH]^-$)

Tributylammonium (1-(4-chloro-3-nitrophenyl)ethylidene)amino sulfate (**2k**)

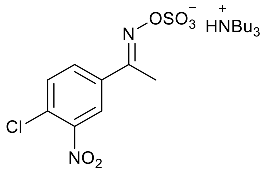

Following **General procedure 2**: 1-(4-chloro-3-nitrophenyl) ethan-1-one oxime (215 mg, 1.0 mmol) and TBSAB (531 mg, 2.0 mmol) were dissolved in dry Dioxane (2.0 mL) and heated

under reflux for 3 h. The flask was cooled, and the solvent removed *in vacuo*. The crude product was purified with (SiO_2; Hexane/EtoAc, 1:1, R_f = 0.2) to afford the title compound as a clear oil (321 mg, 67%).

^{1}H NMR (400 MHz, DMSO-d$_6$) δ_H 8.85 (s, 1H), 8.30 (d, *J* = 2.1 Hz, 1H), 8.00 (d, *J* = 8.5 Hz, 1H), 7.85 (dd, *J* = 8.5, 2.1 Hz, 1H), 3.06 – 3.00 (m, 6H), 2.21 (s, 3H), 1.60-1.54 (m, 6H), 1.32 (h, *J* = 7.4 Hz, 6H), 0.91 (t, *J* = 7.4 Hz, 9H).

^{13}C NMR (101 MHz, DMSO-d$_6$) δ_C 153.8, 148.2, 136.8, 132.3, 131.7, 126.1, 123.4, 52.3, 25.5, 19.8, 14.0, 13.0.

LRMS m/z (ESI-) 292.96 ([M-Bu$_3$NH]$^-$, 100%)

HRMS m/z (ESI-) $C_8H_6ClN_2O_6S$ requires 292.9619, found 293.292.9624 ([M-Bu$_3$NH]$^-$)

Tributylammonium (1-(4-trifluoromethylphenyl)ethylidene)amino sulfate (**2l**)

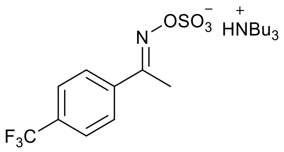

Following **General procedure 2**: 1-(4-(trifluoromethyl) phenyl)ethan-1-one oxime (203 mg, 1.0 mmol) and TBSAB (531.0 mg, 2.0 mmol) were dissolved in dry MeCN (2.0 mL) and heated under reflux for 3 h. The flask was cooled, and the solvent removed *in vacuo*. The flask was charged with H_2O (20 mL) and the aqueous mixture extracted with EtOAc (4 × 30 mL). The organic extracts were pooled, dried ($MgSO_4$), filtered and the filtrate solvent was removed *in vacuo* to afford the desired product as a clear oil (412 mg, 88%)

^{1}H NMR (400 MHz, DMSO-d$_6$) δ_H 8.98 (s, 1H), 7.98- 7.89 (m, 2H), 7.82-7.73 (m, 2H),3.04 – 2.92 (m, 6H), 2.13 (s, 3H), 2.01 (s, 3H), 1.57-1.49 (m, 6H), 1.31 (h, *J* = 7.4 Hz, 6H), 0.91 (t, *J* = 7.4 Hz, 9H).

^{13}C NMR (101 MHz, DMSO-d$_6$) δ_C 173.0, 150.5, 140.5 (d, $^2J_{C\text{-}F}$ = 30 Hz), 127.5 (q, $^4J_{C\text{-}F}$ = 4.2 Hz), 125.8 (q, $^3J_{C\text{-}F}$ = 12.2 Hz), 116.8 (d, $^1J_{C\text{-}F}$ = 280.3 Hz), 52.2, 25.5, 19.8, 14.0, 13.2

^{19}F NMR (377 MHz, DMSO-d$_6$) δ_F -61.21

LRMS m/z (ESI-) 282.00 ([M-Bu$_3$NH]$^-$, 100%)

HRMS m/z (ESI-) $C_9H_7F_3NO_4S$ requires 282.0001, found 282.0003 ([M-Bu$_3$NH]$^-$)

Tributylammonium (1-(3-chloro-4-methoxyphenyl)ethylidene)amino sulfate (**2m**)

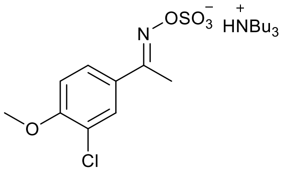

Following **General procedure 2**.: 1-(3-chloro-4-methoxyphenyl) ethan-1-one oxime (200 mg, 1.0 mmol) and TBSAB (531.0 mg, 2.0 mmol) were dissolved in dry MeCN (2.0 mL) and heated under reflux for 3 h. The flask was cooled, and the solvent removed *in vacuo*. The flask was charged with H_2O (20 mL) and the aqueous mixture extracted with EtOAc (4 × 30 mL). The organic extracts were pooled, dried ($MgSO_4$), filtered and the filtrate solvent was removed *in vacuo* to afford the desired product as a clear oil (442 mg, 95%)

^{1}H NMR (400 MHz, DMSO-d_6) δ_H 8.98 (s, 1H), 7.52 (d, *J* = 2.1 Hz, 1H), 7.48 (dd, *J* = 8.5, 2.1 Hz, 1H), 7.21 (d, *J* = 8.5 Hz, 1H), 3.88 (s, 3H), 3.04 – 2.92 (m, 6H), 2.13 (s, 3H), 1.57 (m, 6H), 1.31 (h, *J* = 7.4 Hz, 6H), 0.91 (t, *J* = 7.4 Hz, 9H).

^{13}C NMR (101 MHz, DMSO-d_6) δ_C 155.5, 129.2, 127.3, 126.5, 121.1, 116.9, 112.1, 56.3, 51.8, 25.0, 19.4, 13.5, 12.5.

LRMS m/z (ESI-) 277.99 ([M-Bu_3NH]$^-$, 100%)

HRMS m/z (ESI-) $C_9H_9ClNO_5S$ requires 277.9896, found 277.9901 ([M-Bu_3NH]$^-$)

Tributylammonium (1-(benzo[*d*][1,3]dioxol-5-yl)ethylidene)amino sulfate (**2n**)

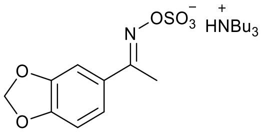

Following **General procedure 2**: (*E*)-1-(benzo[d][1,3]dioxol-5-yl)ethan-1-one oxime (**1n**) (179 mg, 1.0 mmol) and TBSAB (531 mg, 2.0 mmol) were dissolved in dry MeCN (2.0 mL) and heated under reflux for 3 h. The flask was cooled, and the solvent removed *in vacuo*. The flask was charged with H_2O (20 mL) and the aqueous mixture extracted from cold water (10 mL) with EtOAc (4 × 50 mL) and washed with brine (10 mL). The organic layer was dried ($MgSO_4$), filtered, and evaporated *in vacuo* to afford the title compound as a clear oil (409 mg, 92%).

^{1}H NMR (400 MHz, DMSO-d_6) δ_H 8.99 (s, 1H), 7.23 (d, *J* = 1.7 Hz, 1H), 7.19 (dd, *J* = 8.2, 1.7 Hz, 1H), 6.95 (d, *J* = 8.2 Hz, 1H), 6.06 (s, 2H), 3.06 – 2.98 (m, 6H), 2.12 (s, 3H), 1.64 – 1.52 (m, 6H), 1.31 (h, *J* = 7.4 Hz, 6H), 0.91 (t, *J* = 7.4 Hz, 9H).

^{13}C NMR (101 MHz, DMSO-d_6) δ_C 156.3, 149.8, 134.3, 131.5, 121.2, 108.9, 106.4, 101.0, 53.8, 29.4, 19.8, 14.6, 13.3.

LRMS m/z (ESI-) 258.01 ([M-Bu_3NH]$^-$, 100%)

HRMS m/z (ESI-) $C_9H_8NO_6S$ requires 258.0129, found 258.0132 ([M-Bu_3NH]$^-$

Tributylammonium benzylideneamino sulfate (**2o**)

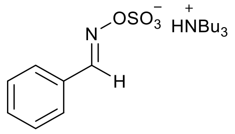

Following **general procedure 2**: Benzaldehyde oxime (181 mg, 1.0 mmol) and TBSAB (531 mg, 2.0 mmol) were dissolved in anhydrous MeCN (2.0 mL) and heated under reflux for 3 h. The flask was cooled, and the solvent removed *in vacuo*. The crude product was purified with (SiO_2; Hexane/EtoAc, 1:1, R_f = 0.2) to afford the desired product as a clear oil as a clear oil (257 mg, 65%).

^{1}H NMR (400 MHz, DMSO-d_6) δ_H 8.21 (1 H, s), 7.67 – 7.63 (2 H, m), 7.46 – 7.42 (3 H, m), 5.75 (1 H, s), 3.09 – 2.88 (m, 6H), 1.62 – 1.58 (m, 6H), 1.32 (h, *J* = 7.4 Hz, 6H), 0.91 (t, *J* = 7.4 Hz, 9H).

^{13}C NMR (101 MHz, DMSO-d_6) δ_C 152.7, 132.3, 130.7, 129.6, 127.7, 52.3, 27.5, 20.4, 15.1.

LRMS m/z (ESI-) 200.00 ([M-Bu_3NH]$^-$, 100%),

HRMS m/z (ESI-) $C_7H_6NO_4S$ requires 200.0045, found 200.0044 ([M-Bu_3NH]$^-$)

Tributylammonium (*E*)-(1-(4-(methylsulfonyl)phenyl)ethylidene)amino sulfate (**2p**)

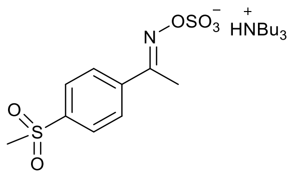

Following **General procedure 2**: 1-(4-(methylsulfonyl) phenyl)ethan-1-one oxime (213 mg, 1.0 mmol) and TBSAB (531 mg, 2.0 mmol) were dissolved in dry MeCN (2.0 mL) and heated under reflux for 3 h. The flask was cooled, and the solvent removed *in vacuo*. The flask was charged with H_2O (20 mL) and the aqueous mixture extracted with EtOAc (4 × 30 mL). The

organic extracts were pooled, dried ($MgSO_4$), filtered and the filtrate solvent was removed *in vacuo* to afford the desired product as a clear oil (442 mg, 95%).

^{1}H NMR (400 MHz, DMSO-d$_6$) δ_H 8.99 (s, 1H), 7.99 – 7.92 (m, 4H), 3.24 (s, 3H), 3.02 – 2.88 (m, 6H), 2.22 (s, 3H), 1.58 – 1.44 (m, 6H), 1.32 (h, *J* = 7.4 Hz, 6H), 0.91 (t, *J* = 7.4 Hz, 9H).

^{13}C NMR (101 MHz, DMSO-d$_6$) δ_C 155.0, 141.7, 141.2, 127.6, 126.2, 52.3, 43.9, 25.5, 19.8, 14.0, 13.2.

LRMS m/z (ESI-) 291.99 ([M-$Bu_3NH]^-$, 100%)

HRMS m/z (ESI-) $C_9H_{10}NO_6S_2$ requires 291.9925, found 291.9924 ([M-$Bu_3NH]^-$)

Tributylammonium (9*H*-fluoren-9-ylidene)amino sulfate (**2q**)

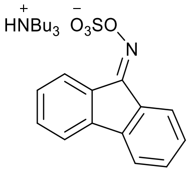

Following **General procedure 2**: 9*H*-fluoren-9-one oxime (192 mg, 1.0 mmol) and TBSAB (531 mg, 2.0 mmol) were dissolved in dry MeCN (2.0 mL) and heated under reflux for 3 h. The flask was cooled, and the solvent removed *in vacuo*. The flask was charged with H_2O (20 mL) and the aqueous mixture extracted from cold water (10 mL) with EtOAc (4 × 50 mL) and washed with brine (10 mL). The organic layer was dried ($MgSO_4$), filtered, and evaporated *in vacuo* to afford the title compound as a clear oil (428 mg, 93%).

^{1}H NMR (400 MHz, DMSO-d$_6$) δ_H 8.92 (s, 1H), 8.26 (dd, *J* = 7.6, 1.0 Hz, 1H), 7.87-7.80 (m, 2H), 7.72 (dd, *J* = 7.6, 1.0 Hz, 1H), 7.57 – 7.45 (m, 2H), 7.39-7.30 (m, 2H), 3.07 – 2.98 (m, 6H), 1.64 – 1.51 (m, 6H), 1.31 (h, *J* = 7.4 Hz, 6H), 0.90 (t, *J* = 7.4 Hz, 9H).

^{13}C NMR (101 MHz, DMSO-d$_6$) δ_C 152.9, 141.3, 140.4, 135.0, 132.3, 131.1, 130.0, 129.6, 129.0, 128.8, 122.1, 121.1, 121.0, 52.3, 25.5, 19.8, 14.0.

LRMS m/z (ESI-) 274.02 ([M-$Bu_3NH]^-$, 100%)

HRMS m/z (ESI-) $C_{13}H_8NO_4S$ requires 274.0192, found 274.0195 ([M-$Bu_3NH]^-$)

Tributylammonium (di(pyridin-2-yl)methylene)amino sulfate (**2r**)

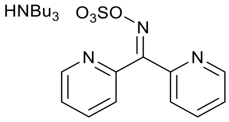

Following **General procedure 2**: di-2-pyridyl ketone oxime (199 mg, 1.0 mmol) and TBSAB (531 mg, 2.0 mmol) were dissolved in dry MeCN (2.0 mL) and heated under reflux for 3 h. The flask was cooled, and the solvent removed *in vacuo*. The flask was charged with H_2O (20 mL) and the aqueous mixture extracted from cold water (10 mL) with EtOAc (4 × 50 mL) and washed with brine (10 mL). The organic layer was dried ($MgSO_4$), filtered, and evaporated *in vacuo* to afford the title compound as a clear oil (460 mg, 99%)

^{1}H NMR (400 MHz, DMSO-d_6) δ_H 8.99 (s, 1H), 8.59 (ddd, *J* = 4.8, 1.8, 1.0 Hz, 1H), 8.46 (ddd, *J* = 4.8, 1.8, 1.0 Hz, 1H), 7.96 – 7.84 (m, 3H), 7.49 (dt, *J* = 7.8, 1.0 Hz, 1H), 7.44 – 7.38 (m, 2H), 3.06 – 2.97 (m, 6H), 1.64 – 1.52 (m, 6H), 1.31 (h, *J* = 7.4 Hz, 6H), 0.91 (t, *J* = 7.4 Hz, 9H).

^{13}C NMR (101 MHz, DMSO-d_6) δ_C 157.5, 154.8, 152.2, 149.5, 149.4, 137.9, 136.5, 125.7, 124.7, 124.0, 53.1, 26.9, 20.6, 15.2.

LRMS m/z (ESI-) 278.02([M-$Bu_3NH]^-$, 100%)

HRMS m/z (ESI-) $C_{11}H_8N_3O_4S$ requires 278.0244, found 278.0240 ([M-$Bu_3NH]^-$)

Tributylammonium (phenyl(pyridin-2-yl)methylene)amino sulfate (**2s**)

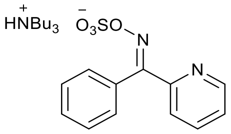

Following **General procedure 2**: phenyl(pyridin-2-yl)methanone oxime (198 mg, 1.0 mmol) and TBSAB (531 mg, 2.0 mmol) were dissolved in dry MeCN (2.0 mL) and heated under reflux for 3 h. The flask was cooled, and the solvent removed *in vacuo*. The flask was charged with H_2O (20 mL) and the aqueous mixture extracted from cold water (10 mL) with EtOAc (4 × 50 mL) and washed with brine (10 mL). The organic layer was dried ($MgSO_4$), filtered, and evaporated *in vacuo* to afford the title compound as a clear oil (394 mg, 88%).

^{1}H NMR (400 MHz, DMSO-d_6) δ_H 8.94 (s, 1H), 8.67 (ddd, *J* = 4.9, 1.8, 1.0 Hz, 1H), 7.95 (td, *J* = 7.7, 1.8 Hz, 1H), 7.52 – 7.35 (m, 7H), 3.07 – 2.98 (m, 6H), 1.62 – 1.58 (m, 6H), 1.31 (h, *J* = 7.4 Hz, 6H), 0.91 (t, *J* = 7.4 Hz, 9H).

^{13}C NMR (101 MHz, DMSO-d_6) δ_C 192.7, 157.1, 152.1, 149.9, 137.0, 135.3, 130.2, 128.8, 128.2, 127.9, 125.6, 124.3, 52.2, 25.5, 19.9, 13.9.

LRMS m/z (ESI-) 277.03([M-$Bu_3NH]^-$, 100%)

HRMS m/z (ESI-) $C_{12}H_9N_2O_4S$ requires 277.0325, found 277.0324 ([M-$Bu_3NH]^-$)

N-phenylacetamide (**3a**)[S4]

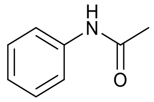

Following **general procedure 3**: A round bottom flask was charged with **2a** (400 mg, 1.0 mmol), TBSAB (256 mg, 1.0 mmol), $NaHCO_3$ (504 mg, 6.0 mmol) and Water (10 mL). The reaction was heated under reflux for 4 h monitored by TLC. The crude product was washed with cold water (3 × 10 mL) and then filtered, freeze-dried to give the desired product as a white solid (163 mg, 99%).

M.P. 112-114 °C (lit. 112-114 °C)[S4]

^{1}H NMR (400 MHz, DMSO-d$_6$) δ_H 9.90 (s, 1H), 7.56 (d, *J* = 7.8 Hz, 2H), 7.28 (t, *J* = 7.8 Hz, 2H), 7.01 (t, *J* = 7.8 Hz, 1H), 2.03 (s, 3H).

^{13}C NMR (101 MHz, DMSO-d$_6$) δ_C 168.2, 139.8, 128.6, 122.9, 119.0, 23.6.

LRMS m/z (ESI+) 136.07 ($[M+H]^+$, 100%)

HRMS m/z (ESI+) $C_8H_{10}NO$ requires 136.0711, found 136.0710 ($[M+H]^+$)

N-(4-fluorophenyl)acetamide (**3c**)[S5]

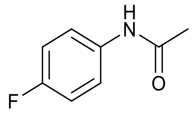

Following **general procedure 3**: A round bottom flask was charged with **2c** (419 mg, 1.0 mmol), TBSAB (256 mg, 1.0 mmol), $NaHCO_3$ (504 mg, 6.0 mmol) and Water (10 mL). The reaction was heated under reflux for 4 h monitored by TLC. The crude product was washed with cold water (3 × 10 mL) and then filtered, freeze-dried to give the desired product as a white solid (279 mg, 91%).

M.P. 152-153 °C (lit. 152.3-153.5 °C)[S5]

^{1}H NMR (400 MHz, DMSO-d$_6$) δ_H 9.96 (1 H, s), 7.61 – 7.55 (2 H, m), 7.15 – 7.09 (2 H, m), 2.02 (3 H, s).

^{13}C NMR (101 MHz, DMSO-d$_6$) δ_C 168.6, 159.5 (d, $^1J_{C\text{-}F}$ = 239.4 Hz), 135.0 (d, $^4J_{C\text{-}F}$ = 2.5 Hz), 121.2 (d, $^3J_{C\text{-}F}$ = 7.5 Hz), 115.8 (d, $^2J_{C\text{-}F}$ = 22.3 Hz), 25.0

^{19}F NMR (377 MHz, MeOD) δ -120.75

LRMS m/z (ESI+) 154.06 ($[M+H]^+$, 100%)

HRMS m/z (ESI+) C_8H_9FNO requires 154.0598, found 154.0599 ($[M+H]^+$)

N-(4-methylphenyl)acetamide (**3d**)[S6]

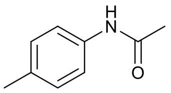

Following **general procedure 3**: A round bottom flask was charged with **2d** (414 mg, 1.0 mmol), TBSAB (256 mg, 1.0 mmol), $NaHCO_3$ (504 mg, 6.0 mmol) and Water (10 mL). The reaction was heated under reflux for 4 h monitored by TLC. The crude product was washed with cold water (3 × 10 mL) and then filtered, freeze-dried to give the desired product as a white solid (148 mg, 99%).

M.P. 151-153 °C (lit. 150-151 °C)[S6]

^{1}H NMR (400 MHz, DMSO-d_6) δ_H 9.81 (1 H, s), 7.46 – 7.43 (2 H, m), 7.09 – 7.06 (2 H, m), 2.23 (3 H, s), 2.01 (3 H, s).

^{13}C NMR (101 MHz, DMSO-d_6) δ_C 168.5, 137.3, 132.6, 129.0, 121.2, 24.4, 19.5.

LRMS m/z (ESI+) 150.08 ($[M+H]^+$, 100%)

HRMS m/z (ESI+) $C_9H_{12}NO$ requires 150.0804, found 150.0802 ($[M+H]^+$)

N-(4-methoxyphenyl)acetamide (**3e**)[S7]

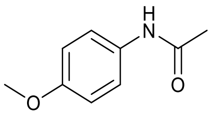

Following **general procedure 3**: A round bottom flask was charged with **2e** (411 mg, 1.0 mmol), TBSAB (256 mg, 1.0 mmol), $NaHCO_3$ (504 mg, 6.0 mmol) and Water (10 mL). The reaction was heated under reflux for 4 h monitored by TLC. The crude product was washed with cold water (3 × 10 mL) and then filtered, freeze-dried to give the desired product as a white solid (163 mg, 99%).

M.P. 125-128 °C (lit. 130-132 °C)[S7]

^{1}**H NMR** (400 MHz, DMSO-d$_6$) δ_H 9.77 (s, 1H), 7.50 – 7.43 (m, 2H), 6.88 – 6.82 (m, 2H), 3.70 (s, 3H), 2.00 (s, 3H).

^{13}**C NMR** (101 MHz, DMSO-d$_6$) δ_C 169.7, 154.1, 134.8, 119.7, 115.5, 55.0, 26.0.

LRMS m/z (ESI+) 166.08 ([M+H]$^+$, 100%)

HRMS m/z (ESI+) $C_9H_{12}NO_2$ requires 166.0839, found 166.0842 ([M+H]$^+$)

N-(4-methylthiophenyl)acetamide (**3h**)[S8]

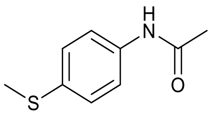

Following **general procedure 3**: A 25 mL round bottom flask was charged with **2h** (480 mg, 1.0 mmol), TBSAB (256 mg, 1.0 mmol), $NaHCO_3$ (504 mg, 6.0 mmol) and Water (10 mL). The reaction was heated under reflux for 4 h monitored by TLC. The crude product was washed with cold water (3 × 10 mL) and then filtered, freeze-dried to give the desired product as a white solid (179 mg, 99%).

M.P. 125-128 °C (lit. 124.8-126.5 °C)[S8]

^{1}**H NMR** (400 MHz, DMSO-d$_6$) δ_H 9.92 (s, 1H), 7.57 – 7.49 (m, 2H), 7.25 – 7.17 (m, 2H), 2.43 (s, 3H), 2.02 (s, 3H).

^{13}**C NMR** (101 MHz, DMSO-d$_6$) δ_C 170.6, 136.1, 131.8, 126.2, 118.4, 25.0, 17.1

LRMS m/z (ESI+) 182.06 ([M+H]$^+$, 100%)

HRMS m/z (ESI+) $C_9H_{12}NOS$ requires 182.0603, found 182.0607 ([M+H]$^+$)

N-(3,4,5-trimethoxyphenyl)acetamide (**3i**)[S4]

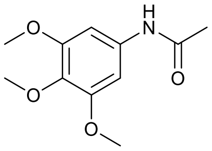

Following **general procedure 3**: A round bottom flask was charged with **2i** (490 mg, 1.0 mmol), TBSAB (256 mg, 1.0 mmol), $NaHCO_3$ (504 mg, 6.0 mmol) and Water (10 mL). The reaction was heated under reflux for 4 h monitored by TLC. The crude product was washed

with cold water (3 × 10 mL) and then filtered, freeze-dried to give the desired product as a white solid (214 mg, 95%).

M.P. 140-142 °C (lit. 140-142 °C)[S4]

^{1}H NMR (400 MHz, DMSO-d$_6$) δ_H 9.85 (s, 1H), 6.95 (s, 2H), 3.72 (s, 6H), 3.60 (s, 3H), 2.01 (s, 3H).

^{13}C NMR (101 MHz, DMSO-d$_6$) δ_C 169.0, 155.2, 137.7, 134.3, 97.2, 59.5, 55.6, 24.5

LRMS m/z (ESI+) 226.10 ([M+H]$^+$, 100%)

HRMS m/z (ESI+) $C_{11}H_{16}NO_4$ requires 226.1027, found 226.1022 ([M+H]$^+$)

N-(3-fluoro-4-methoxyphenyl)acetamide (**3j**)[S9]

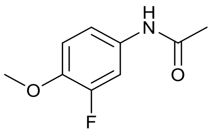

Following **general procedure 3**: A round bottom flask was charged with **2j** (468 mg, 1.0 mmol), TBSAB (256 mg, 1.0 mmol), $NaHCO_3$ (504 mg, 6.0 mmol) and Water (10 mL). The reaction was heated under reflux for 4 h monitored by TLC. The crude product was washed with cold water (3 × 10 mL) and then filtered, freeze-dried then purified with (SiO_2; Hexane/EtOAc, 1:1, R_f = 0.2) to give the desired product as a white solid (119 mg, 65%).

M.P. 165-168 °C (lit. 165-168 °C)[S9]

^{1}H NMR (400 MHz, DMSO-d$_6$) δ_H 9.93 (s, 1H), 7.56 (dd, *J* = 9.0, 2.5 Hz, 1H), 7.20 (ddd, *J* = 9.0, 2.5, 1.4 Hz, 1H), 7.08 (t, *J* = 9.0, 1.4 Hz, 1H), 3.78 (s, 3H), 2.01 (s, 3H).

^{13}C NMR (101 MHz, DMSO-d$_6$) δ_C 168.6 (d, $J_{C\text{-}F}$ = 250.2 Hz), 160.1 (d, $J_{C\text{-}F}$ = 3.2 Hz), 150.1 (d, $J_{C\text{-}F}$ = 13.3 Hz), 146.6 (d, $J_{C\text{-}F}$ = 8.4 Hz), 132.1 (d, $J_{C\text{-}F}$ = 4.2 Hz), 114.9 (d, $J_{C\text{-}F}$ = 1.5 Hz), 108.0 (d, $J_{C\text{-}F}$ = 12.6 Hz), 56.6, 24.3.

^{19}F NMR (377 MHz, DMSO-d$_6$) δ_F -134.08

LRMS m/z (ESI+) 184.07 ([M+H]$^+$, 100%)

HRMS m/z (ESI+) $C_9H_{11}FNO_2$ requires 184.0695, found 184.0692 ([M+H]$^+$)

N-(4-trimethylfluorophenyl)acetamide (**3l**)[S10]

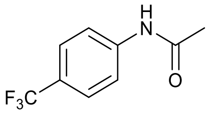

Following **general procedure 3**: A round bottom flask was charged with **2l** (468 mg, 1.0 mmol), TBSAB (256 mg, 1.0 mmol), $NaHCO_3$ (504 mg, 6.0 mmol) and Water (10 mL). The reaction was heated under reflux for 4 h monitored by TLC. The crude product was washed with cold water (3 × 10 mL) and then filtered, freeze-dried then purified with (SiO_2; Hexane/EtOAc, 4:6, R_f = 0.2) to give the desired product as a white solid (132 mg, 65%).

M.P. 103 - 104 °C (lit. 105.5-106.4 °C)[S10]

^{1}H NMR (400 MHz, DMSO-d$_6$) δ_H 10.30 (s, 1H), 7.78 (d, J = 8.2 Hz, 2H), 7.65 (d, J = 8.2 Hz, 2H), 2.08 (s, 3H).

^{13}C NMR (101 MHz, DMSO-d$_6$) δ_C 169.3, 150.3, 141.9 (d, $^2J_{C\text{-}F}$ = 31 Hz), 127.5 (q, $^4J_{C\text{-}F}$ = 4.8 Hz), 127.3 (q, $^3J_{C\text{-}F}$ = 12.6 Hz), 119.2 (d, $^1J_{C\text{-}F}$ = 281.1 Hz), 24.6

^{19}F NMR (377 MHz, DMSO-d$_6$) δ_F -59.25

LRMS m/z (ESI+) 204.06 ([M+H]$^+$, 100%)

HRMS m/z (ESI+) $C_9H_9F_3NO$ requires 204.0628, found 204.0627 ([M+H]$^+$)

N-(3-chloro-4-methoxyphenyl)acetamide (**3m**)[S11]

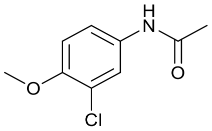

Following **general procedure 3**: A round bottom flask was charged with **2m** (465 mg, 1.0 mmol), TBSAB (256 mg, 1.0 mmol), $NaHCO_3$ (504 mg, 6.0 mmol) and Water (10 mL). The reaction was heated under reflux for 4 h monitored by TLC. The crude product was washed with cold water (3 × 10 mL) and then filtered, freeze-dried then purified with (SiO_2; Hexane/EtOAc, 1:1, R_f = 0.2) to give the desired product as a white solid (110 mg, 55%).

M.P. 82-85 °C (lit. 92-93 °C)[S11]

^{1}H NMR (400 MHz, DMSO-d$_6$) δ_H 9.93 (s, 1H), 7.76 (d, J = 2.6 Hz, 1H), 7.39 (dd, J = 8.9, 2.6 Hz, 1H), 7.08 (d, J = 8.9 Hz, 1H), 3.80 (s, 3H), 2.01 (s, 3H).

^{13}C NMR (101 MHz, DMSO-d$_6$) δ_C 168.5, 150.7, 133.5, 121.0, 120.9, 119.2, 113.4, 56.6, 23.9

LRMS m/z (ESI+) 200.04 ([M^{35}Cl+H]$^+$, 100%), 202.04 ([M^{37}Cl+H]$^+$, 60%)

HRMS m/z (ESI+) $C_9H_{11}ClNO_2$ requires 200.0441, found 200.0439 ([M^{35}Cl+H]$^+$)

N-(benzo[*d*][1,3]dioxol-5-yl)acetamide (**3n**)[S12]

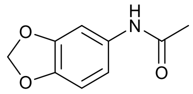

Following **general procedure 3**: A round bottom flask was charged with **2n** (414 mg, 1.0 mmol), TBSAB (256 mg, 1.0 mmol), $NaHCO_3$ (504 mg, 6.0 mmol) and Water (10 mL). The reaction was heated under reflux for 4 h monitored by TLC. The crude product was washed with cold water (3 × 10 mL) and then filtered, freeze-dried to give the desired product as a white solid (159 mg, 80%).

M.P. 135 - 137 °C (lit. 133-134 °C)[S12]

^{1}H NMR (400 MHz, DMSO-d$_6$) δ_H 9.83 (s, 1H), 7.28 (d, *J* = 2.1 Hz, 1H), 6.92 (dd, *J* = 8.4, 2.1 Hz, 1H), 6.82 (d, *J* = 8.4 Hz, 1H), 5.96 (s, 2H), 1.99 (s, 3H).

^{13}C NMR (101 MHz, DMSO-d$_6$) δ_C 168.1, 146.5, 142.4, 134.7, 114.9, 108.9, 101.7, 101.3, 22.9.

LRMS m/z (ESI+) 180.06 ([M+H]$^+$, 100%)

HRMS m/z (ESI+) $C_9H_{10}NO_3$ requires 180.0622, found 180.0620 ([M+H]$^+$)

N-(pyridin-2-yl)benzamide (**3s**)[S13]

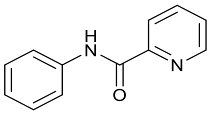

Following **general procedure 3**: A round bottom flask was charged with **2s** (445 mg, 1.0 mmol), TBSAB (256 mg, 1.0 mmol), $NaHCO_3$ (504 mg, 6.0 mmol) and Water (10 mL). The reaction was heated under reflux for 4 h monitored by TLC. The crude product was washed with cold water (3 × 10 mL) and then filtered, freeze-dried to give the desired product as a white solid (160 mg, 89%).

M.P. 78-79 °C (lit. 78.2-78.6 °C)[S13]

^{1}H NMR (400 MHz, DMSO-d_6) δ_H 10.62 (s, 1H), 8.74 (ddd, *J* = 4.8, 1.7, 1.1 Hz, 1H), 8.17 (dt, *J* = 7.9, 1.1 Hz, 1H), 8.07 (td, *J* = 7.9, 1.7 Hz, 1H), 7.93 – 7.89 (m, 2H), 7.68 (ddd, *J* = 7.9, 4.8, 1.1 Hz, 1H), 7.39 – 7.34 (m, 2H), 7.15 – 7.10 (m, 1H).

^{13}C NMR (101 MHz, DMSO-d_6) δ_C 162.9, 150.4, 148.9, 138.8, 138.6, 131.1, 129.2, 128.5, 127.4, 124.4, 122.8, 120.7

LRMS m/z (ESI+) 199.08 ($[M+H]^+$, 100%)

HRMS m/z (ESI+) $C_{12}H_{11}N_2O$ requires 199.0809, found 199.0813 ($[M+H]^+$

Copies of ^{1}H, ^{13}C, ^{19}F NMR spectra

^{1}H NMR spectrum of **1b** (400 MHz, DMSO-d_6)

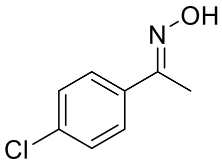

1b

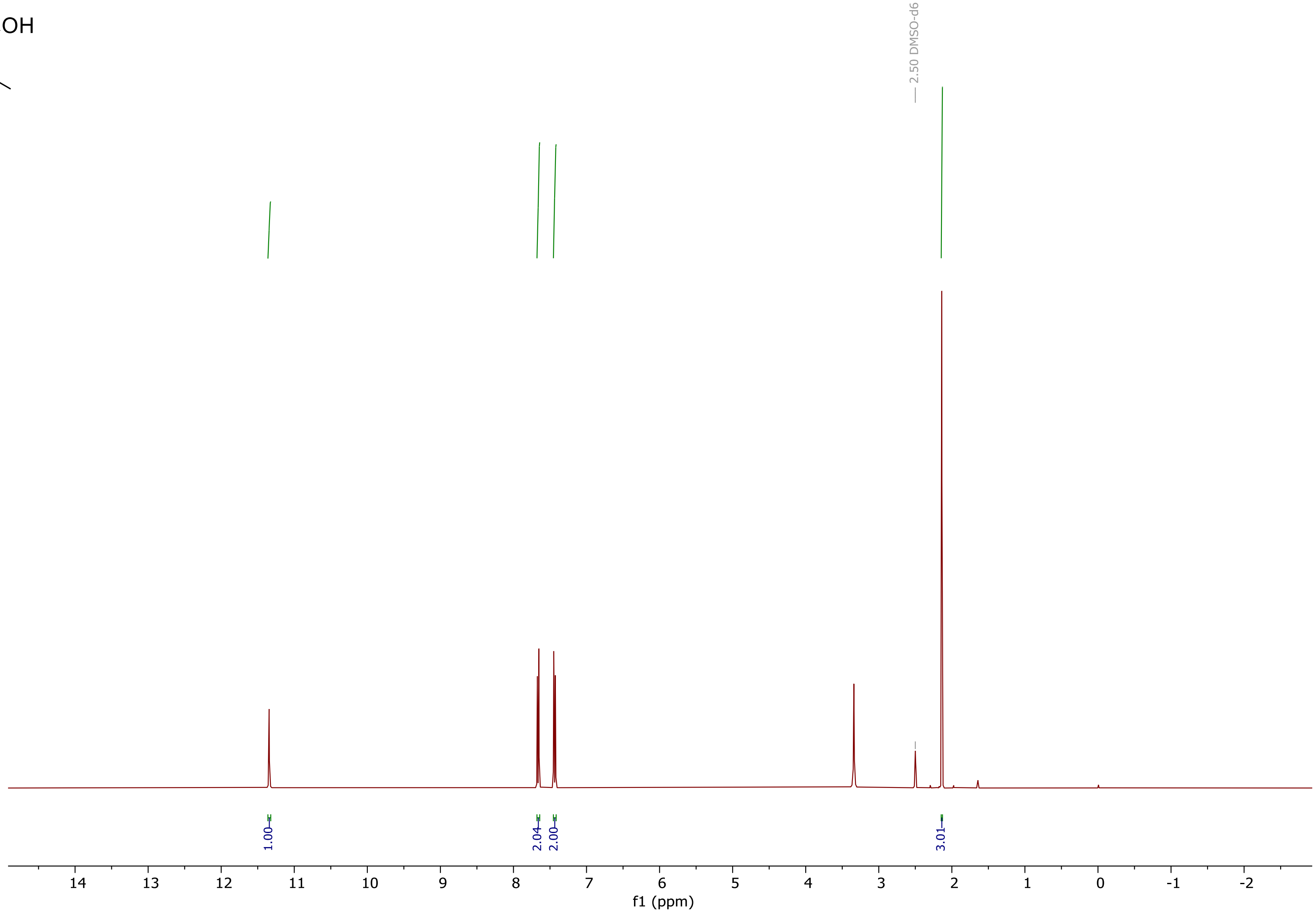

^{13}C NMR spectrum of **1b** (101 MHz, DMSO-d$_6$)

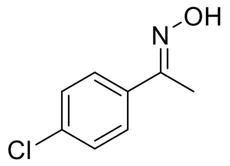

1b

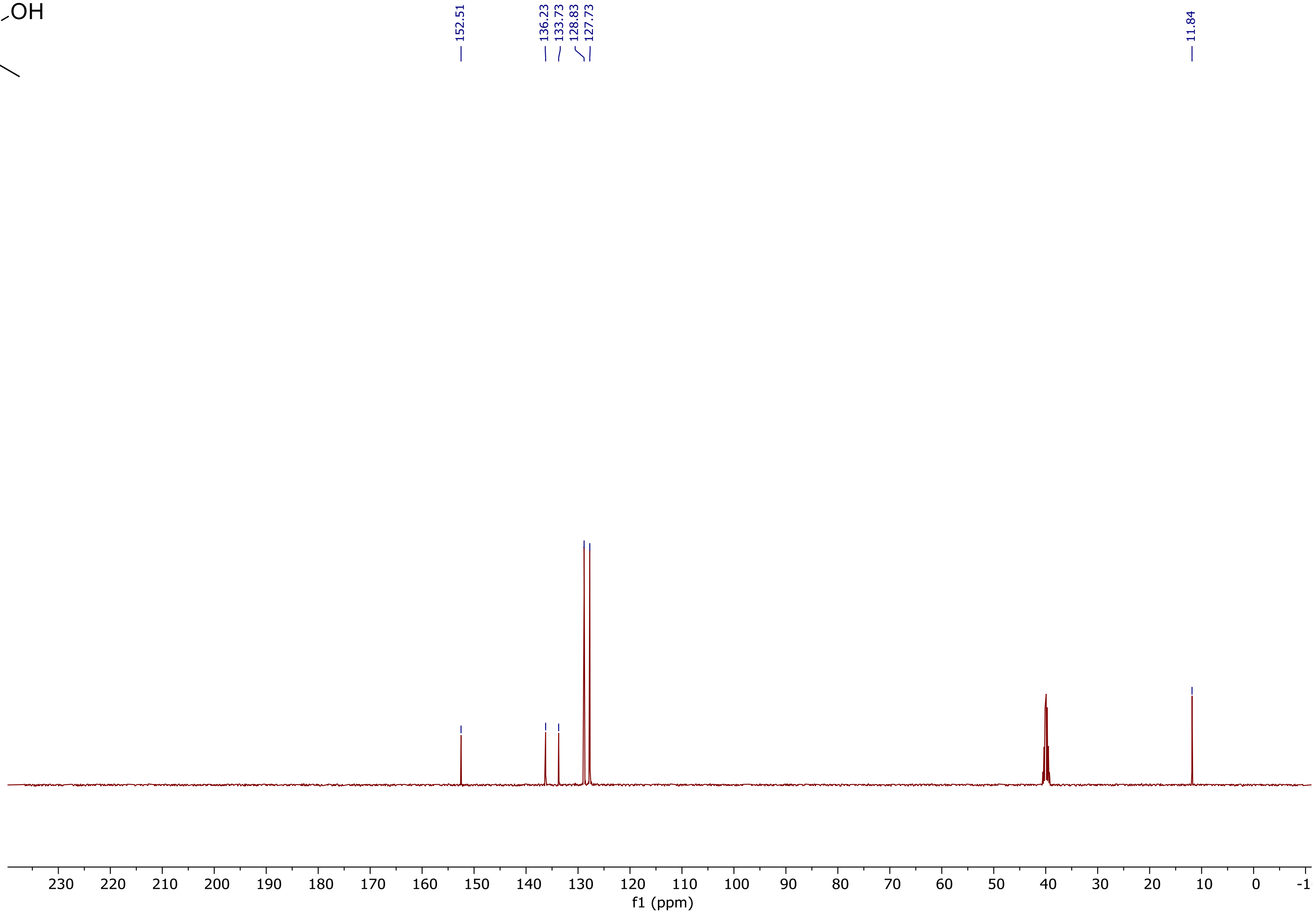

^{1}H NMR spectrum of **1c** (400 MHz, DMSO-d6)

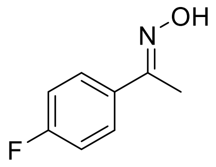

1c

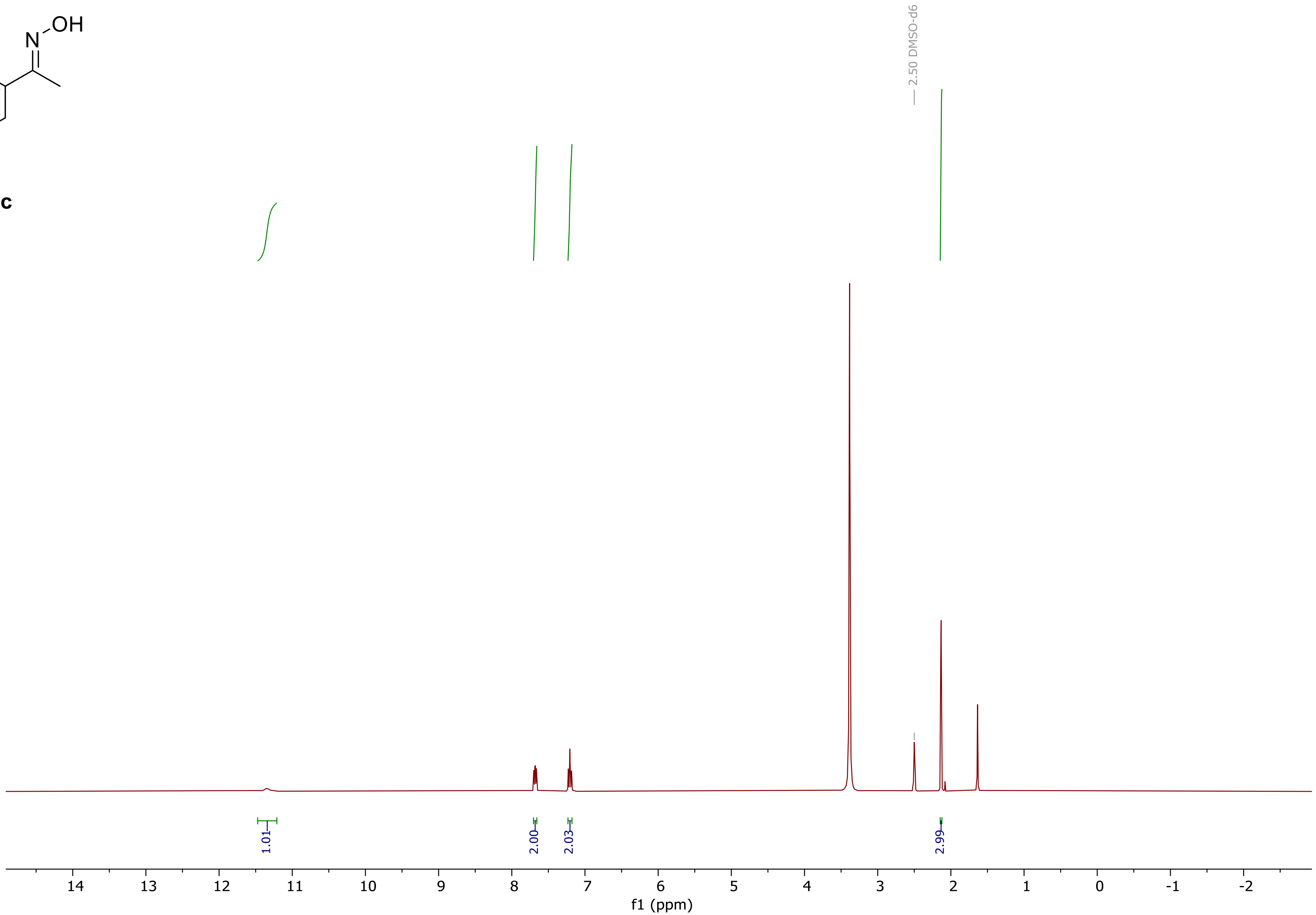

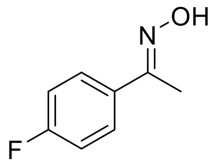

1c

^{13}C NMR spectrum of **1c** (101 MHz, DMSO-d$_6$)

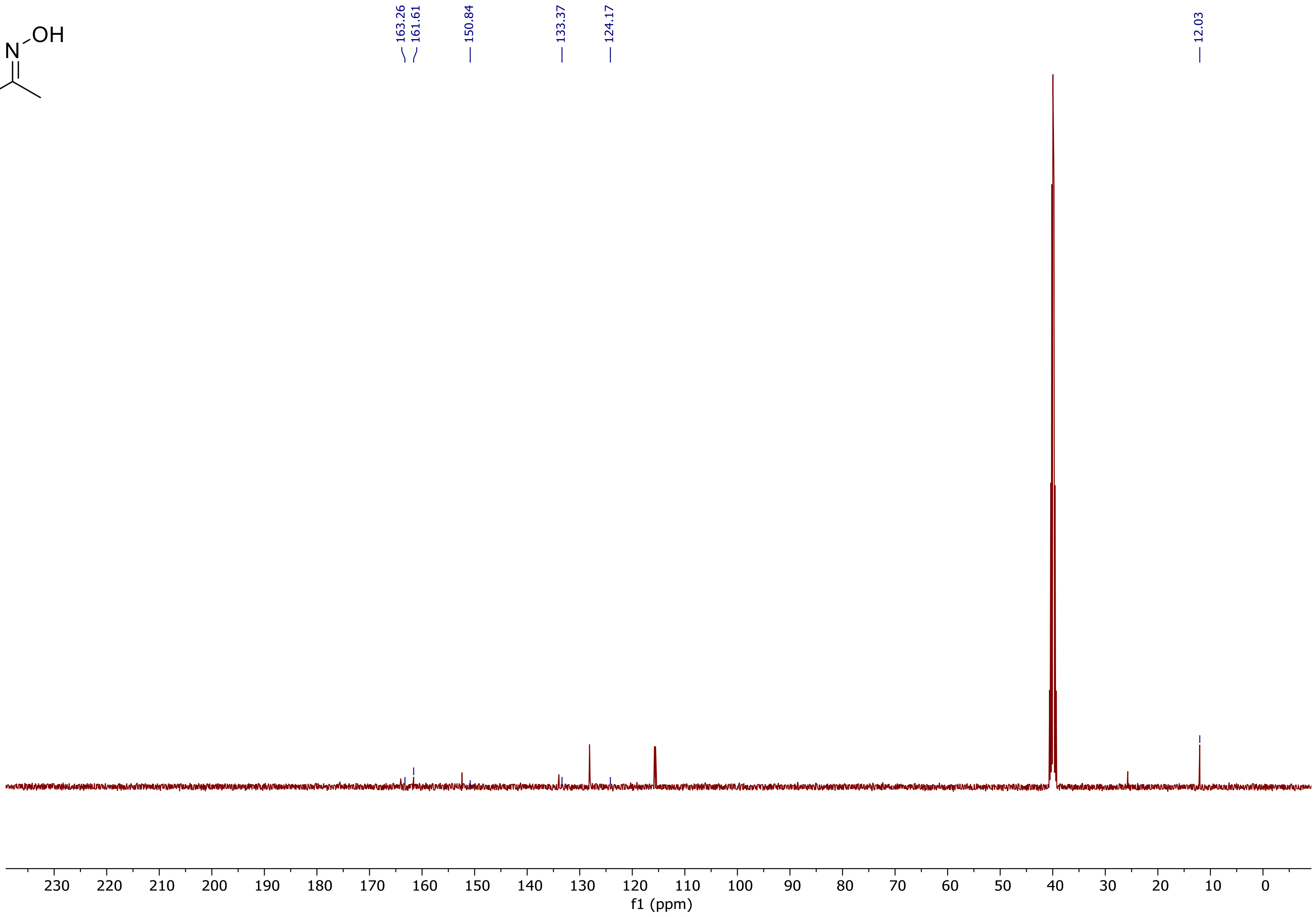

^{19}F NMR spectrum of **1c** (377 MHz, DMSO-d_6)

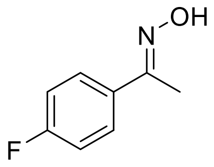

1c

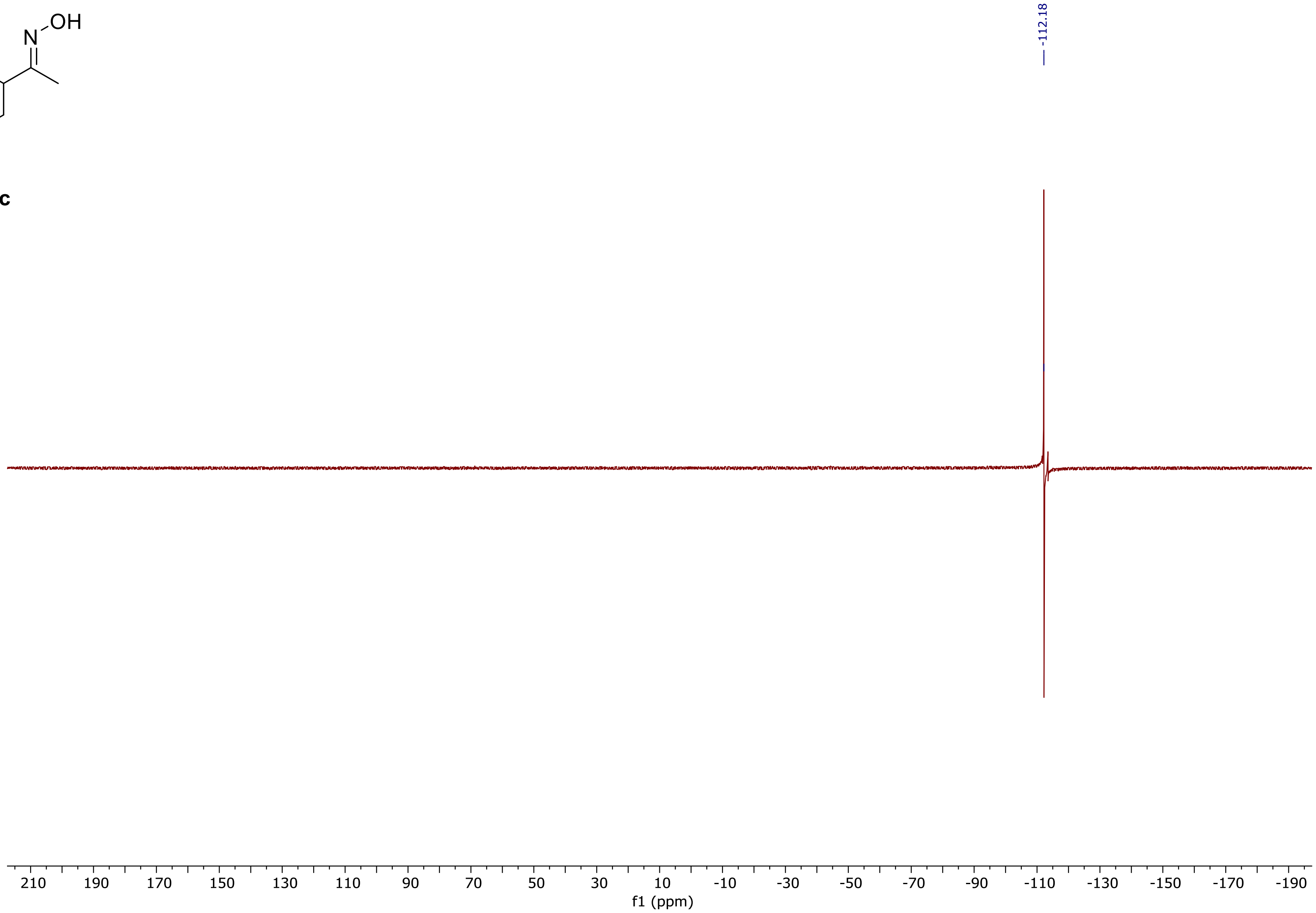

^{1}H NMR spectrum of **1d** (400 MHz, DMSO-d$_6$)

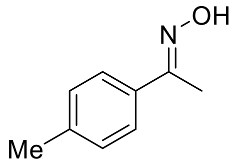

1d

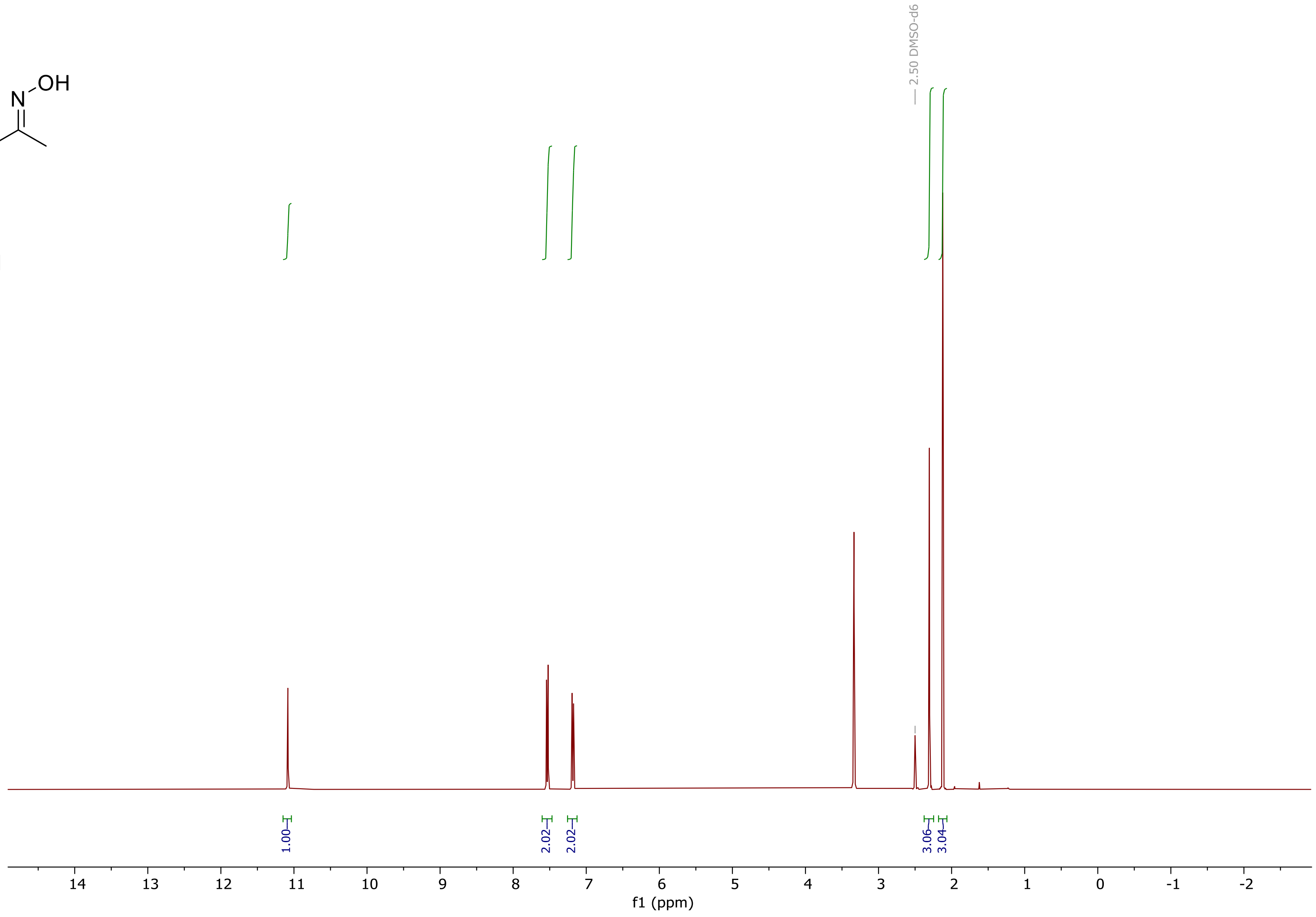

^{13}C NMR spectrum of **1d** (101 MHz, DMSO-d$_6$)

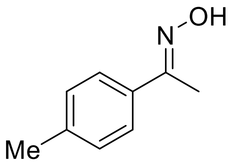

1d

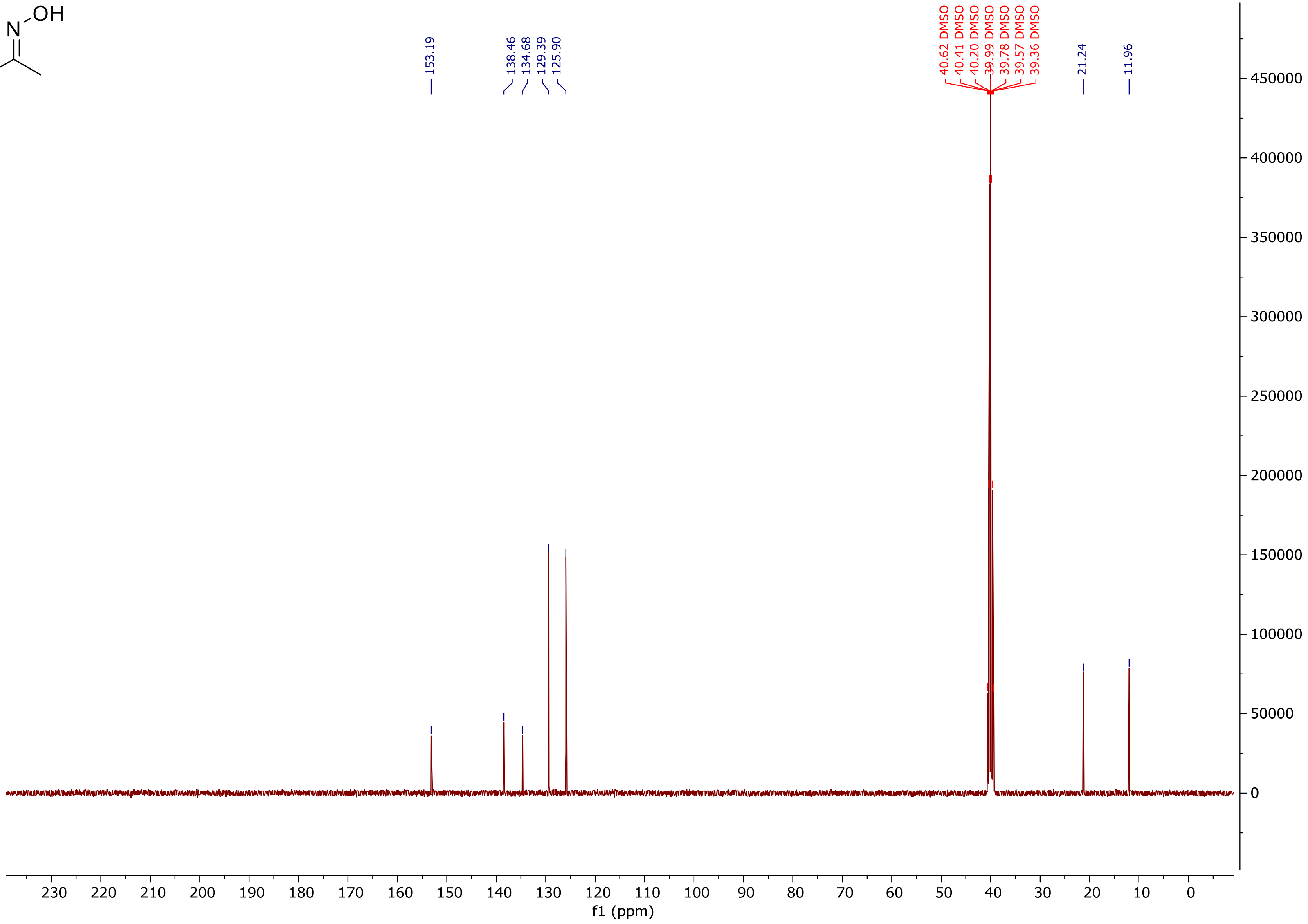

^{1}H NMR spectrum of **1e** (400 MHz, DMSO-d$_6$)

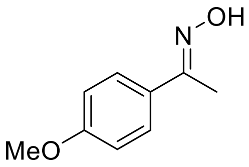

1e

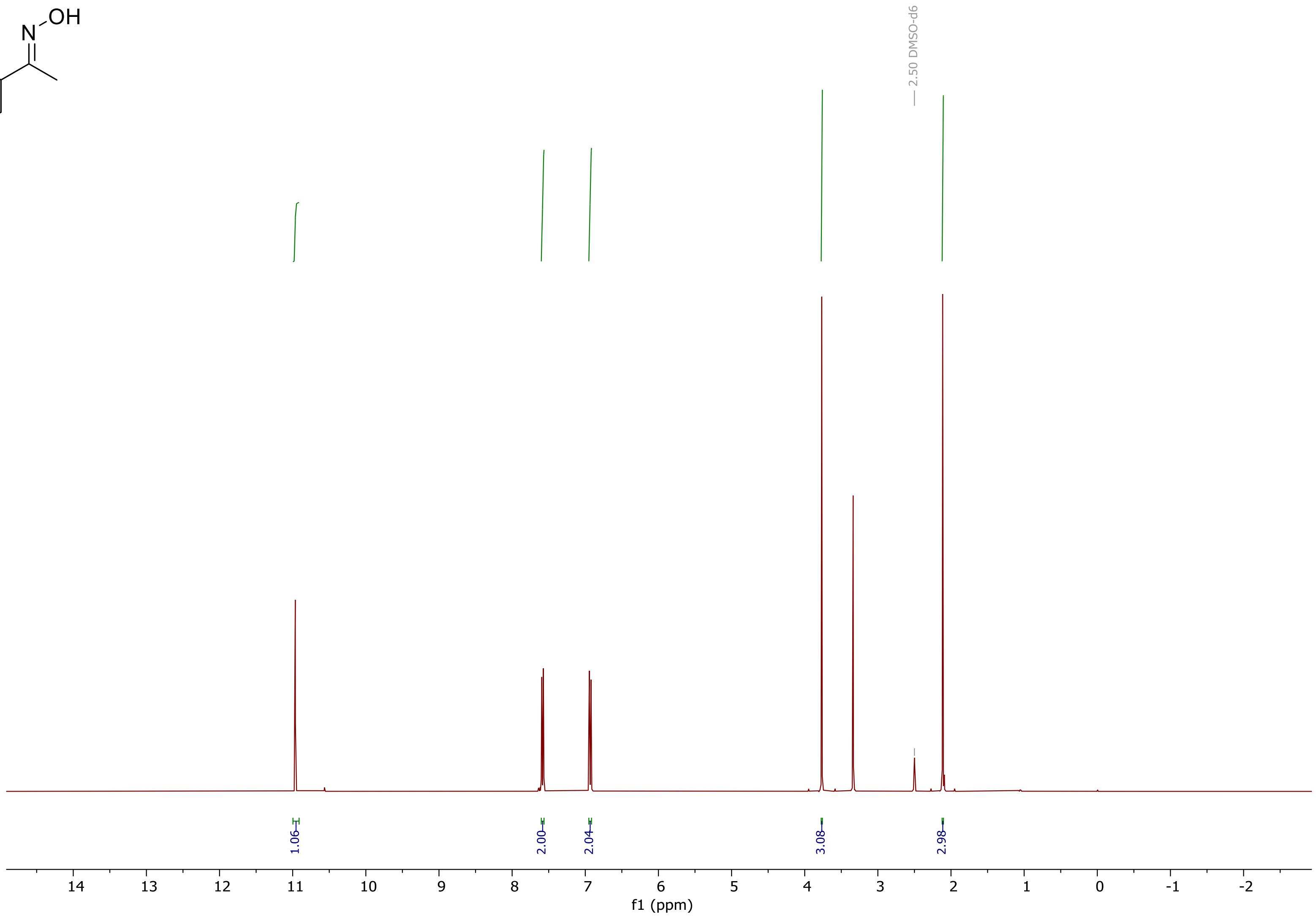

^{13}C NMR spectrum of **1e** (101 MHz, DMSO-d_6)

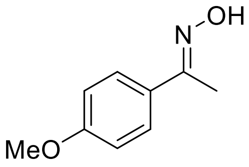

1e

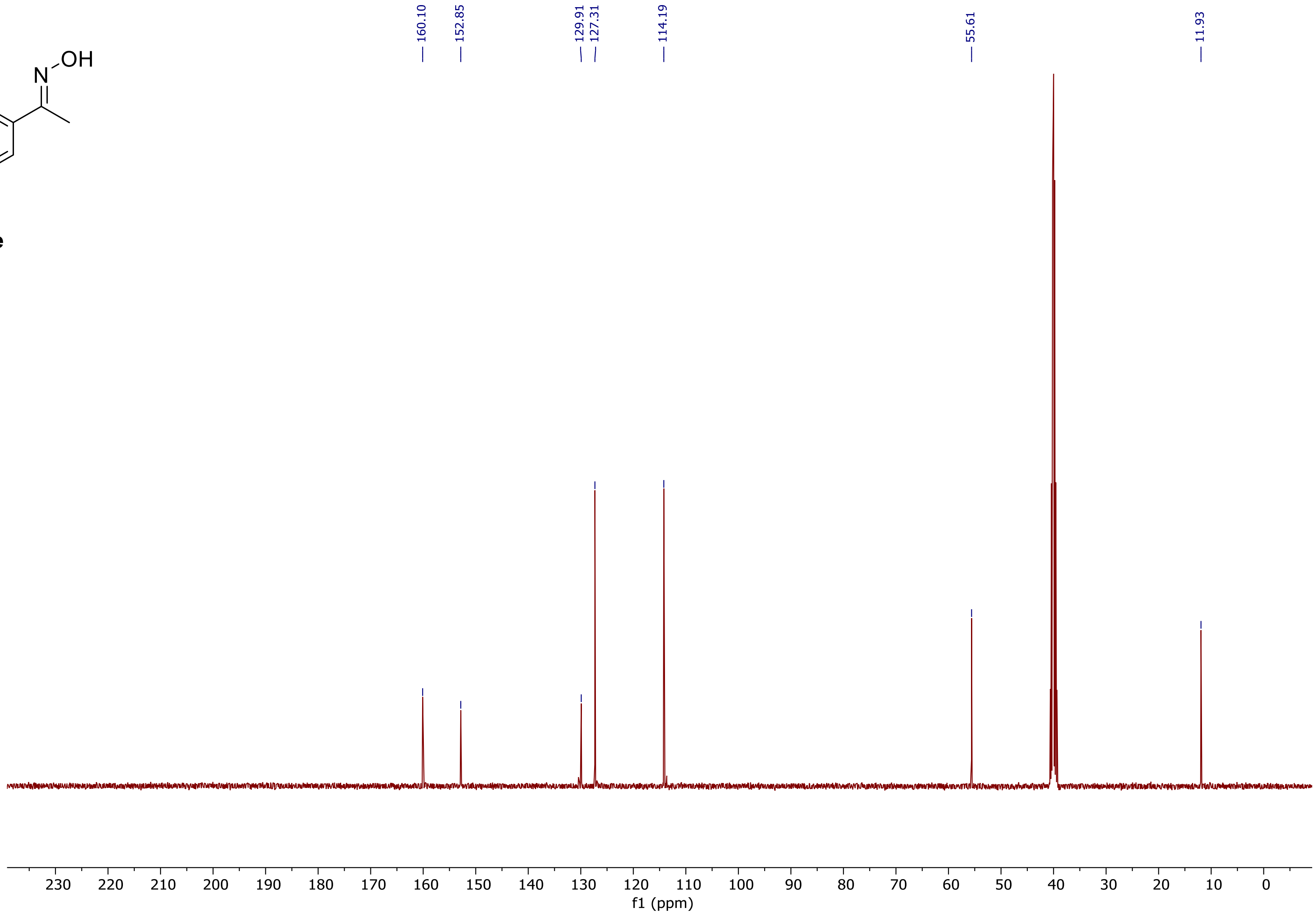

^{1}H NMR spectrum of **1f** (400 MHz, DMSO-d_6)

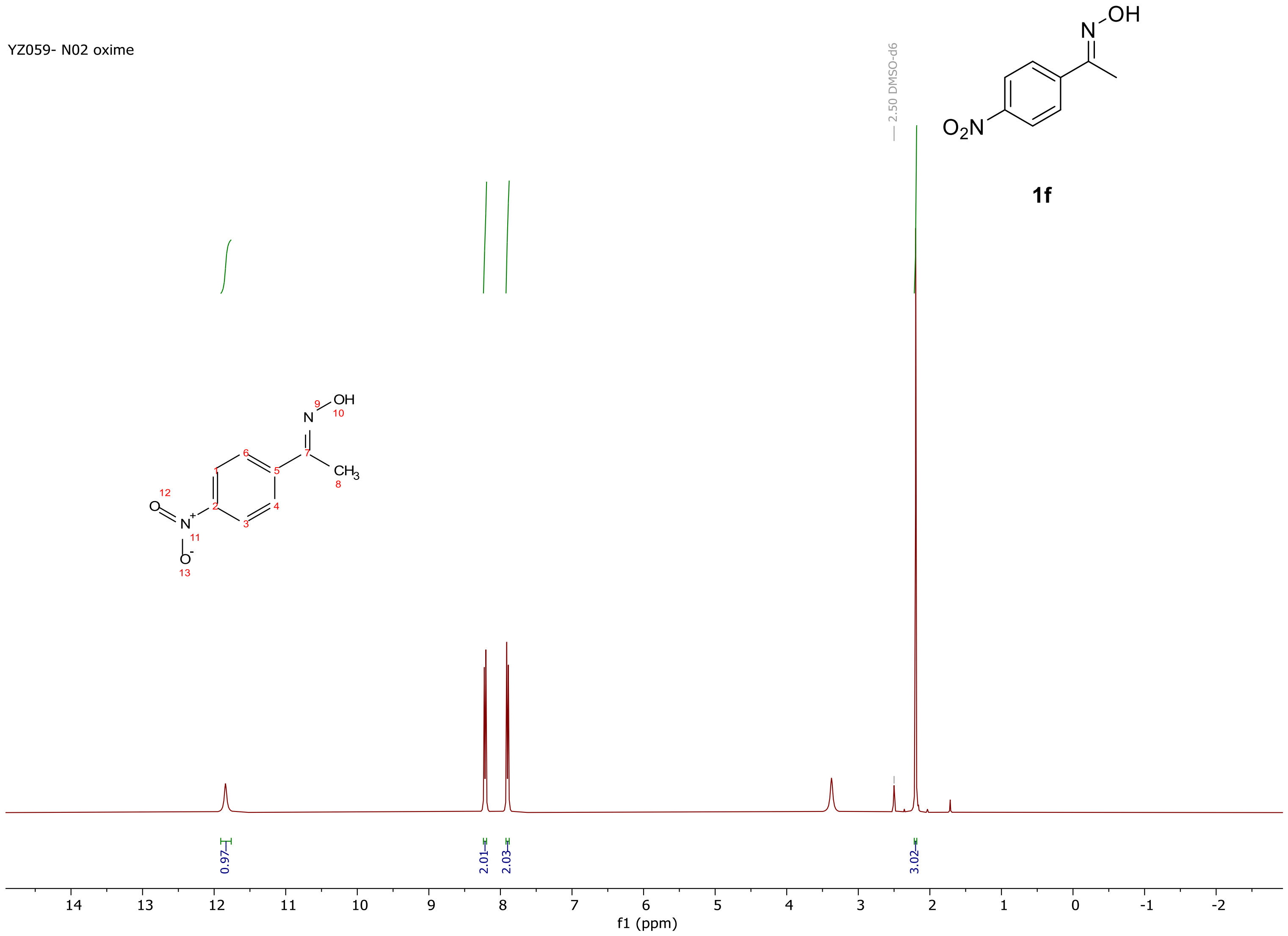

1f

^{13}C NMR spectrum of **1f** (101 MHz, DMSO-d_6)

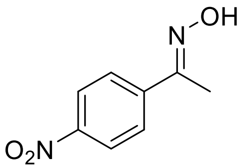

1f

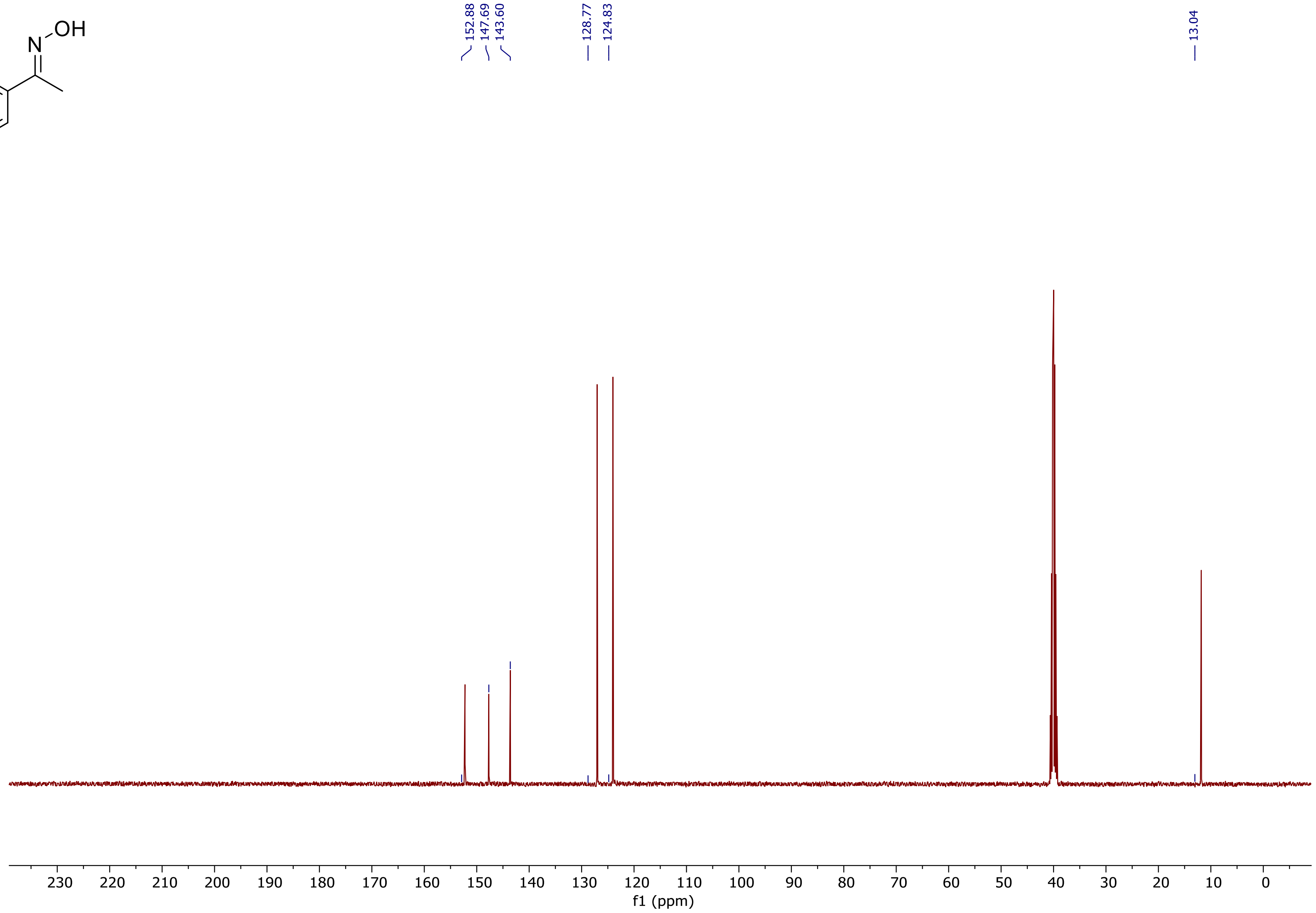

^1H NMR spectrum of **1g** (400 MHz, DMSO-d_6)

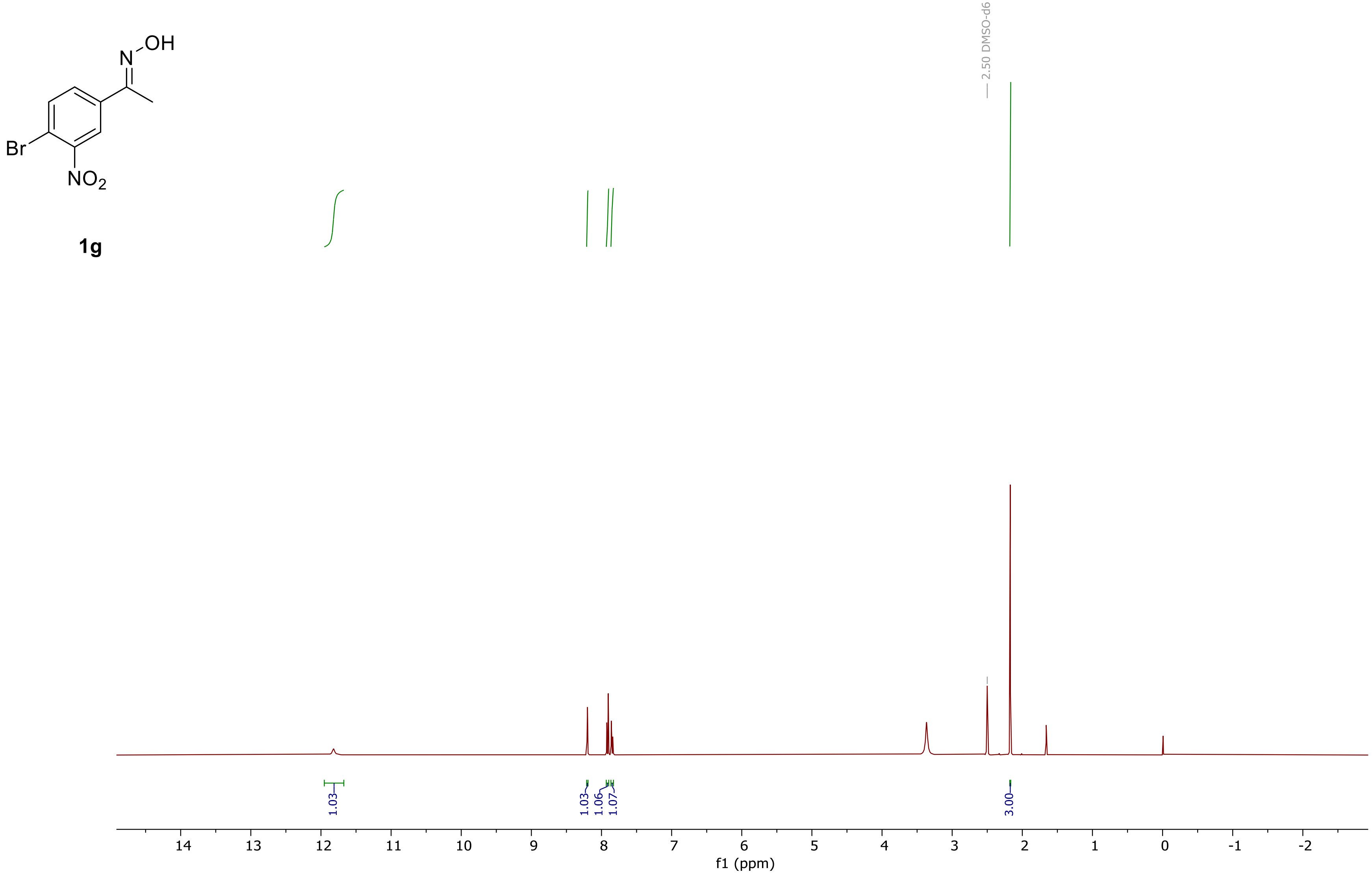

^{13}C NMR spectrum of **1g** (101 MHz, DMSO-d$_6$)

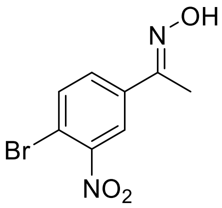

1g

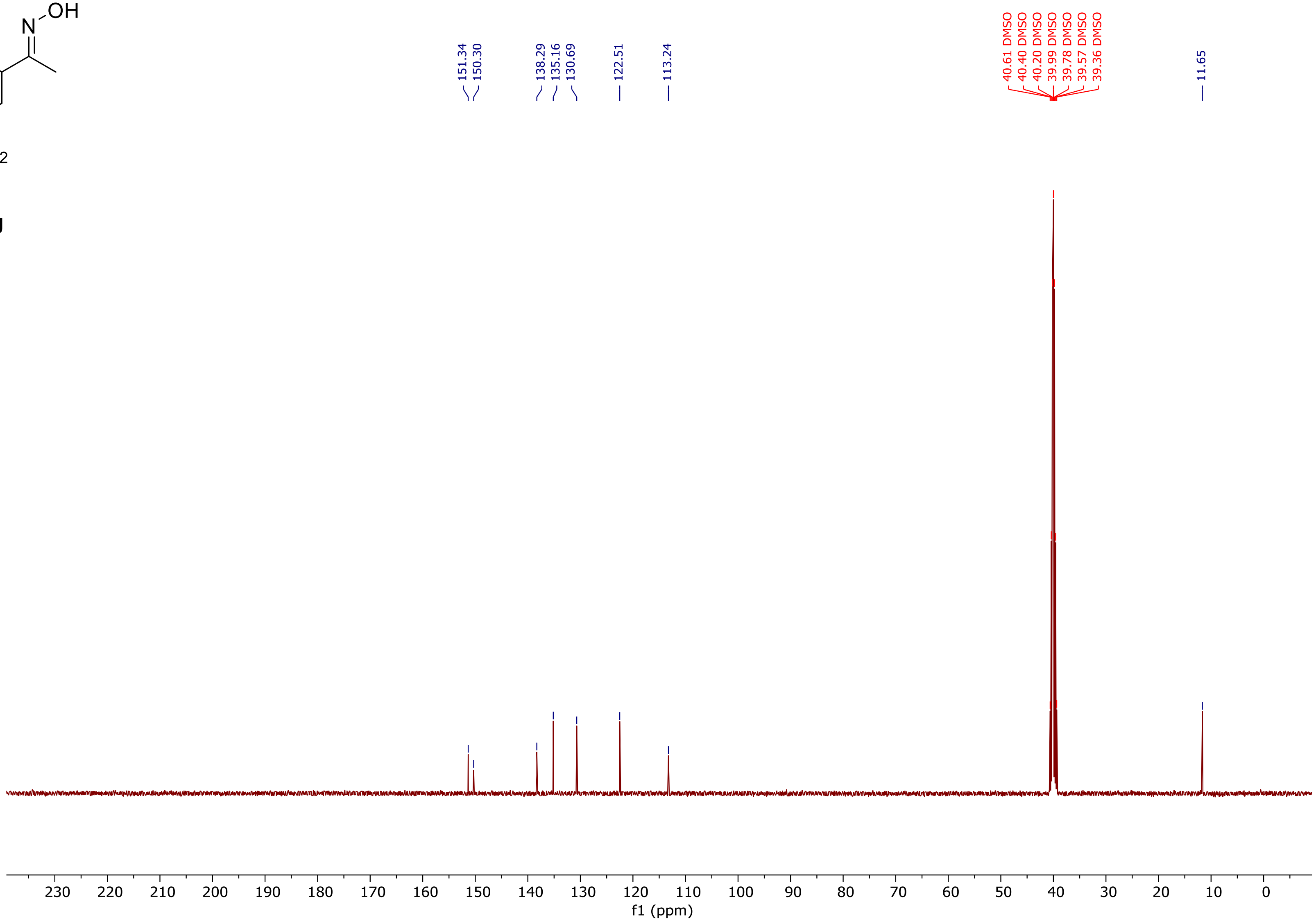

^{1}H NMR spectrum of **1h** (400 MHz, DMSO-d_6)

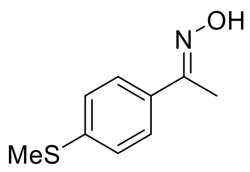

1h

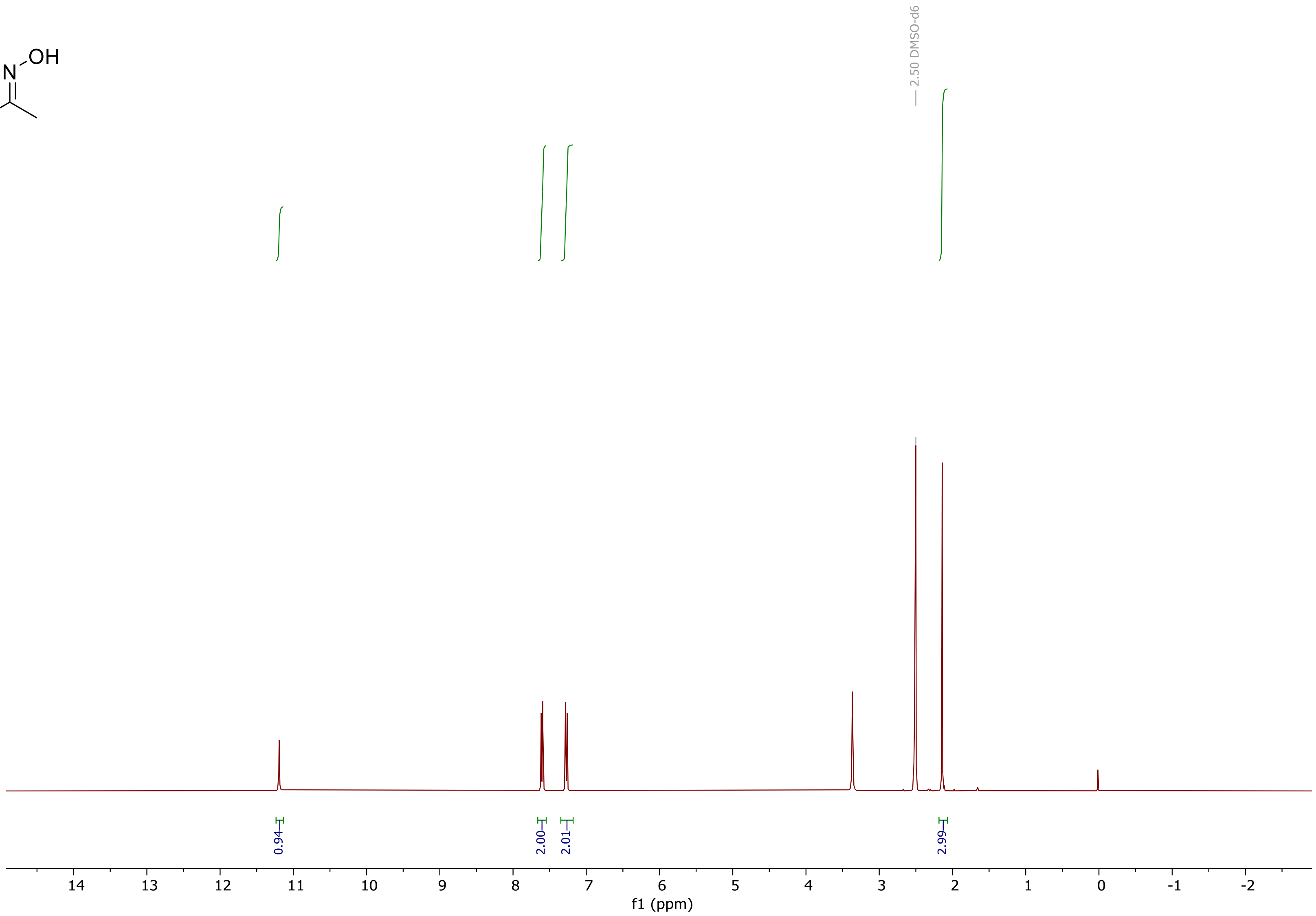

^{13}C NMR spectrum of **1h** (101 MHz, DMSO-d_6)

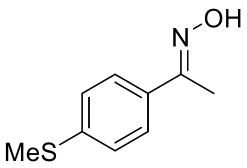

1h

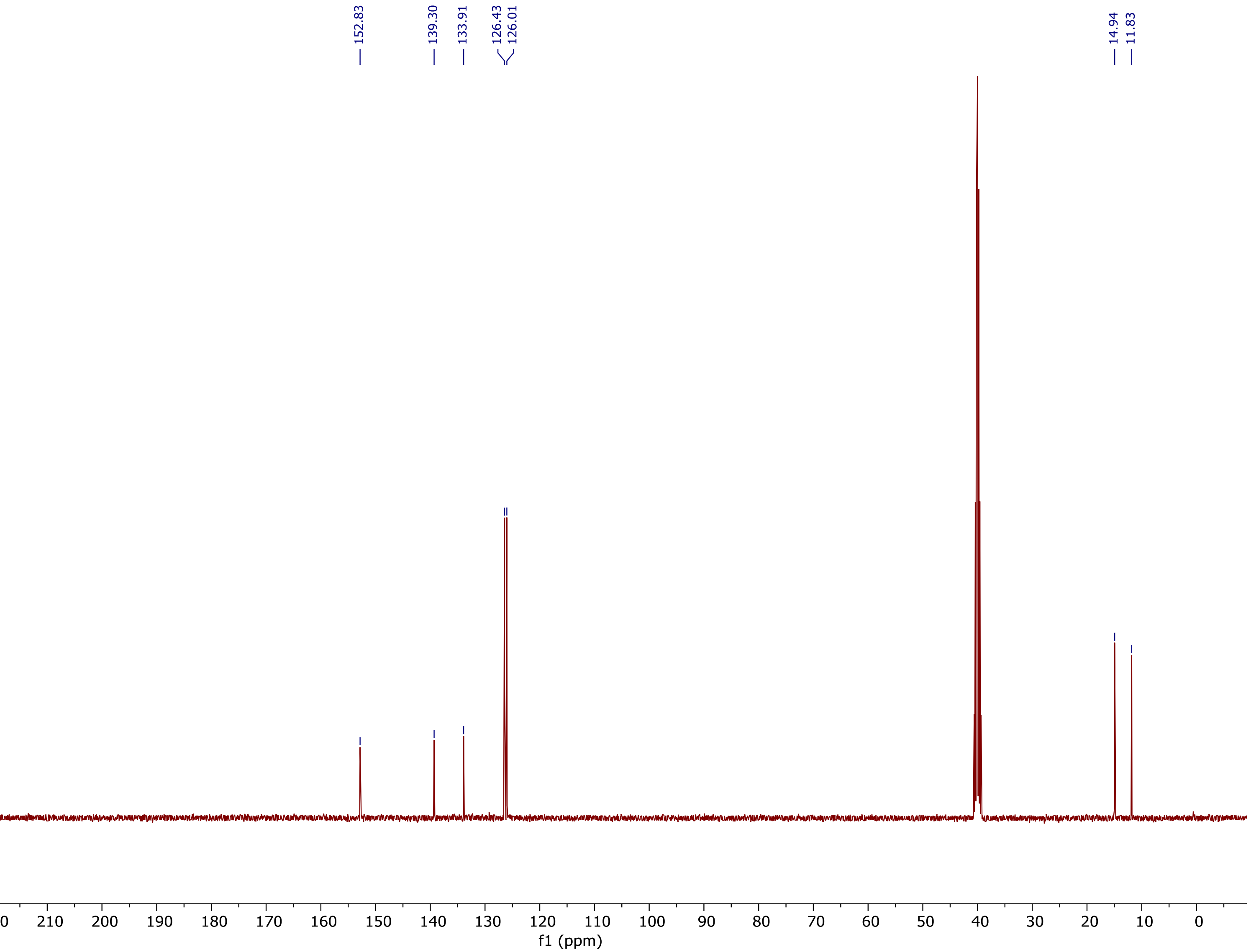

^{1}H NMR spectrum of **1i** (400 MHz, DMSO-d$_6$)

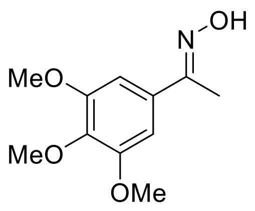

1i

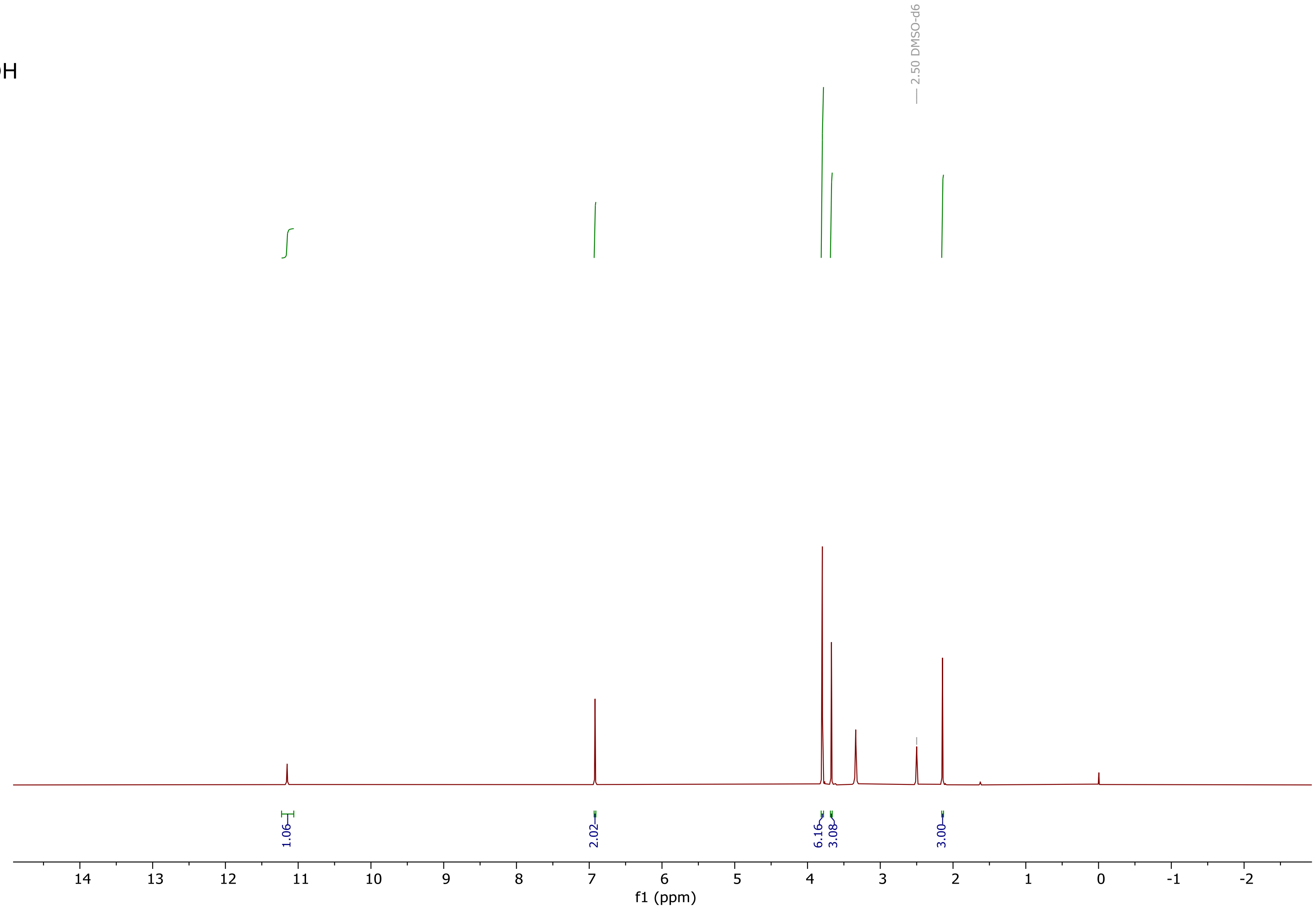

^{13}C NMR spectrum of **1i** (101 MHz, DMSO-d_6)

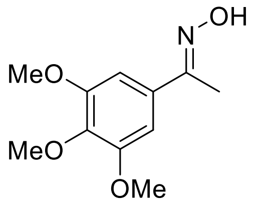

1i

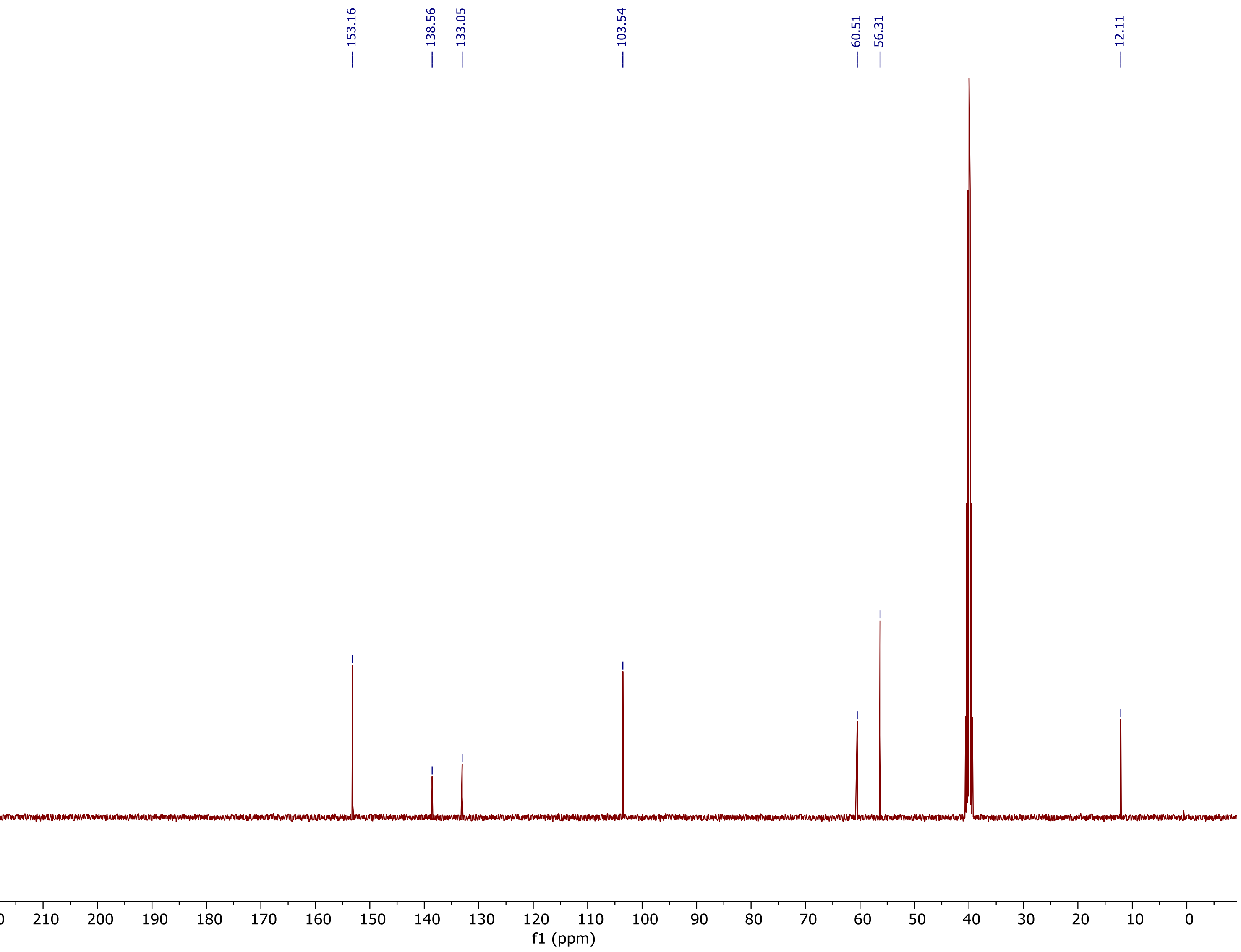

^1H NMR spectrum of **1j** (400 MHz, DMSO-d$_6$)

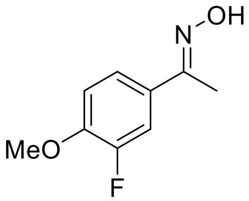

1j

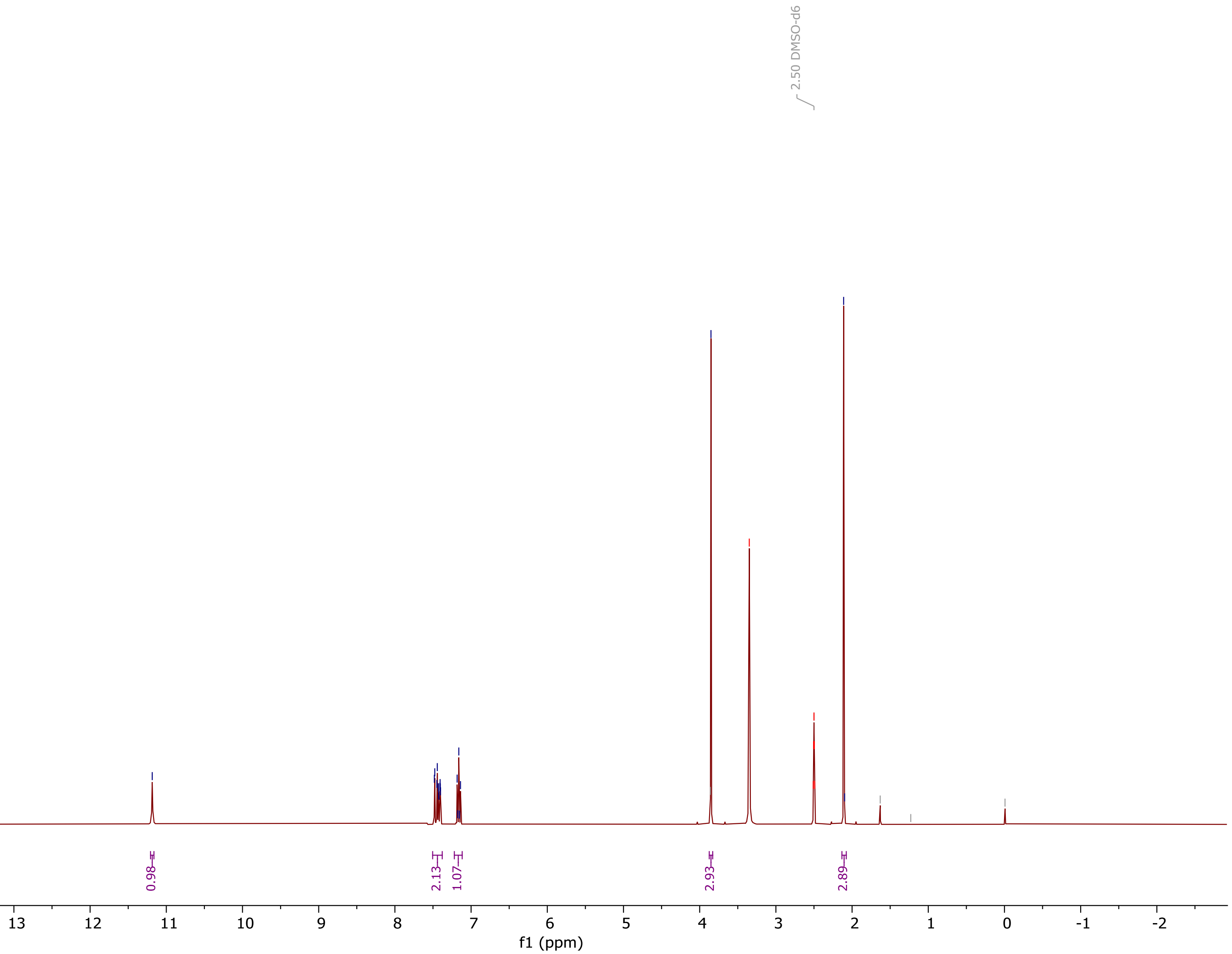

^{13}C NMR spectrum of **1j** (101 MHz, DMSO-d$_6$)

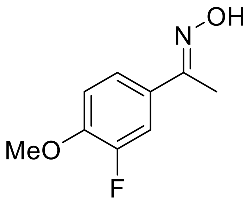

1j

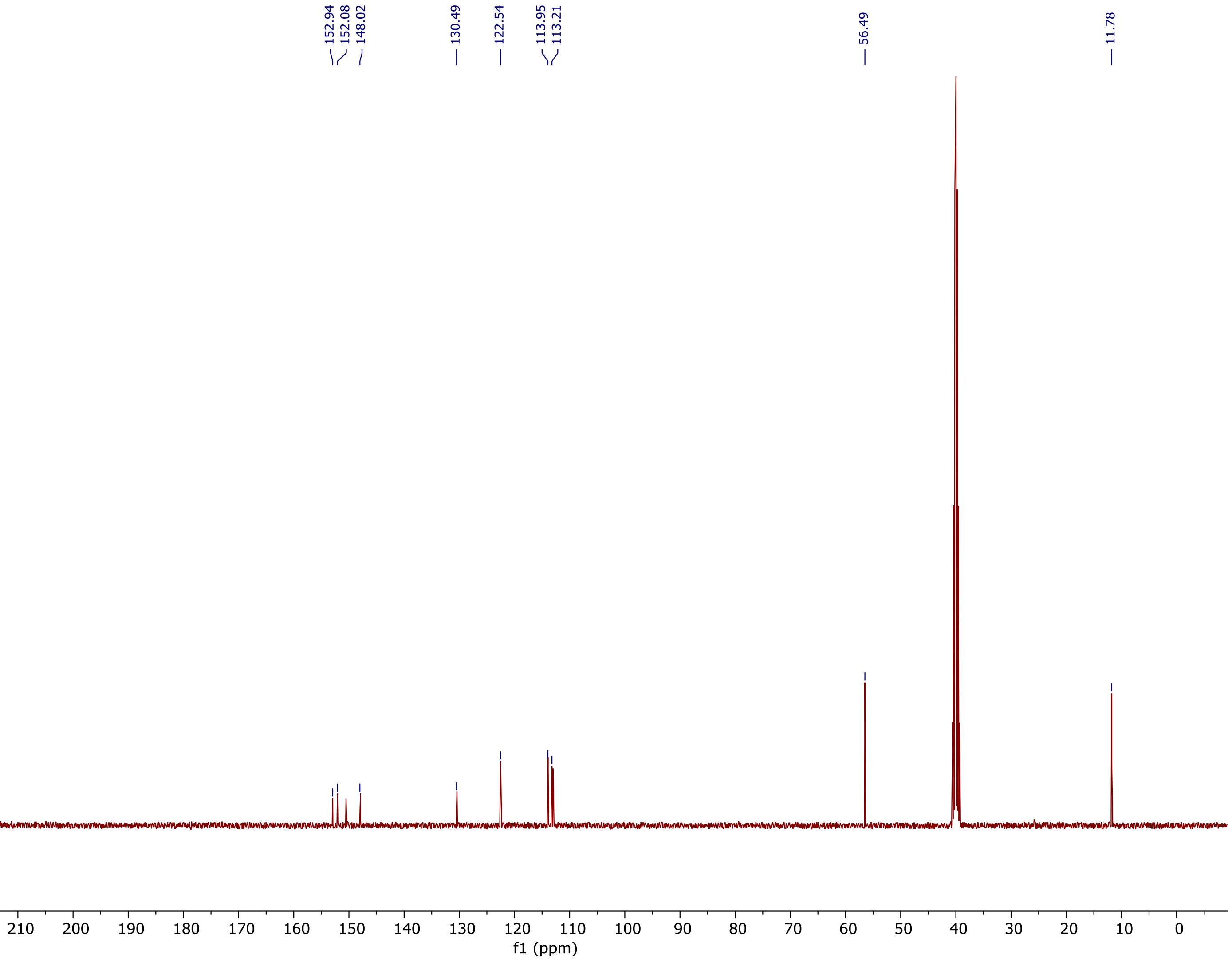

^{19}F NMR spectrum of **1j** (377 MHz, DMSO-d_6)

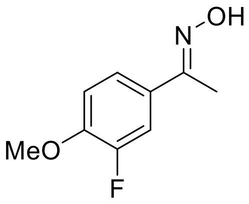

1j

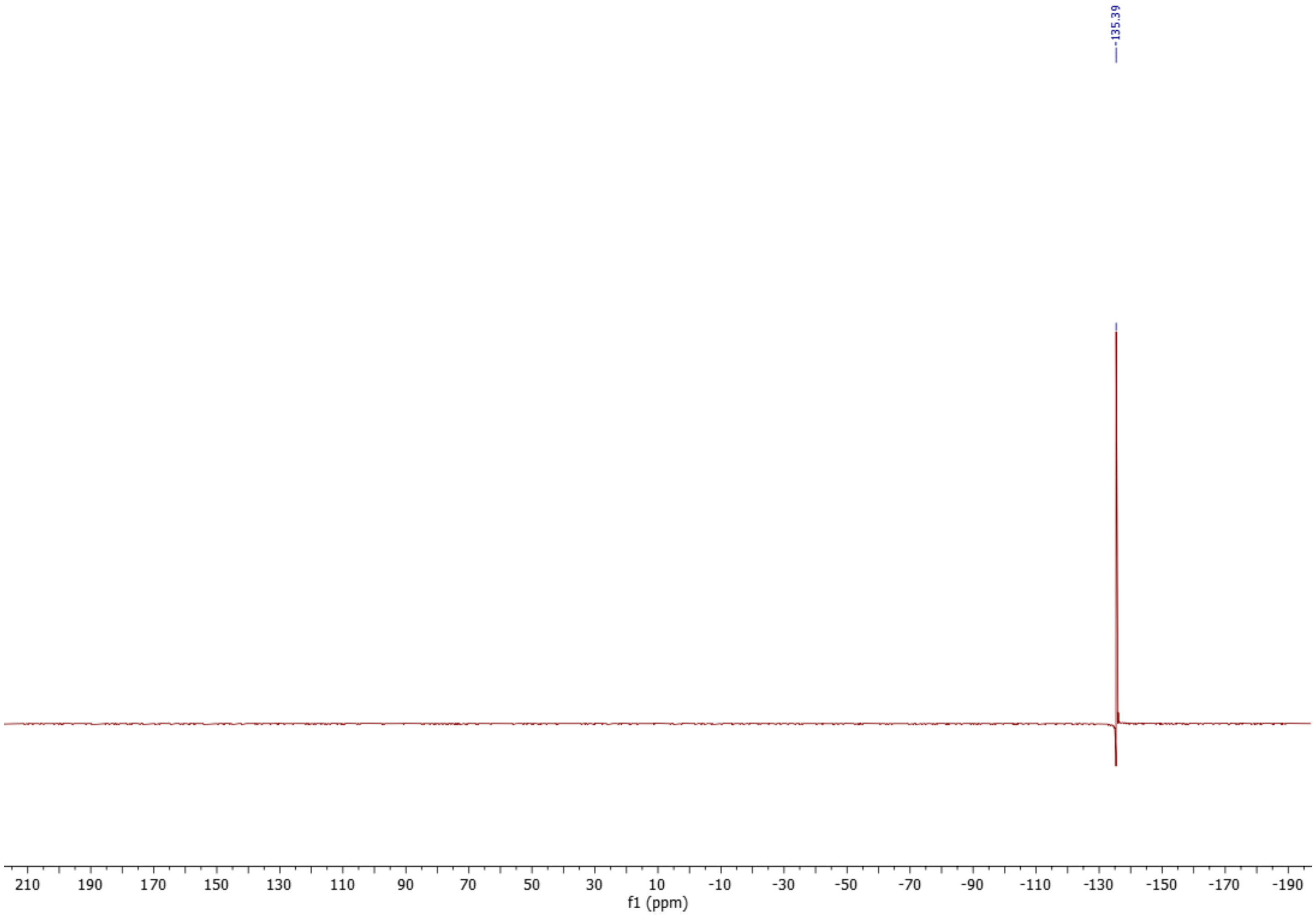

^{1}H NMR spectrum of **1k** (400 MHz, DMSO-d$_6$)

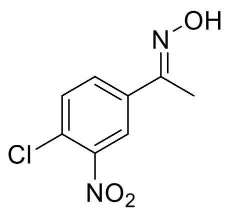

1k

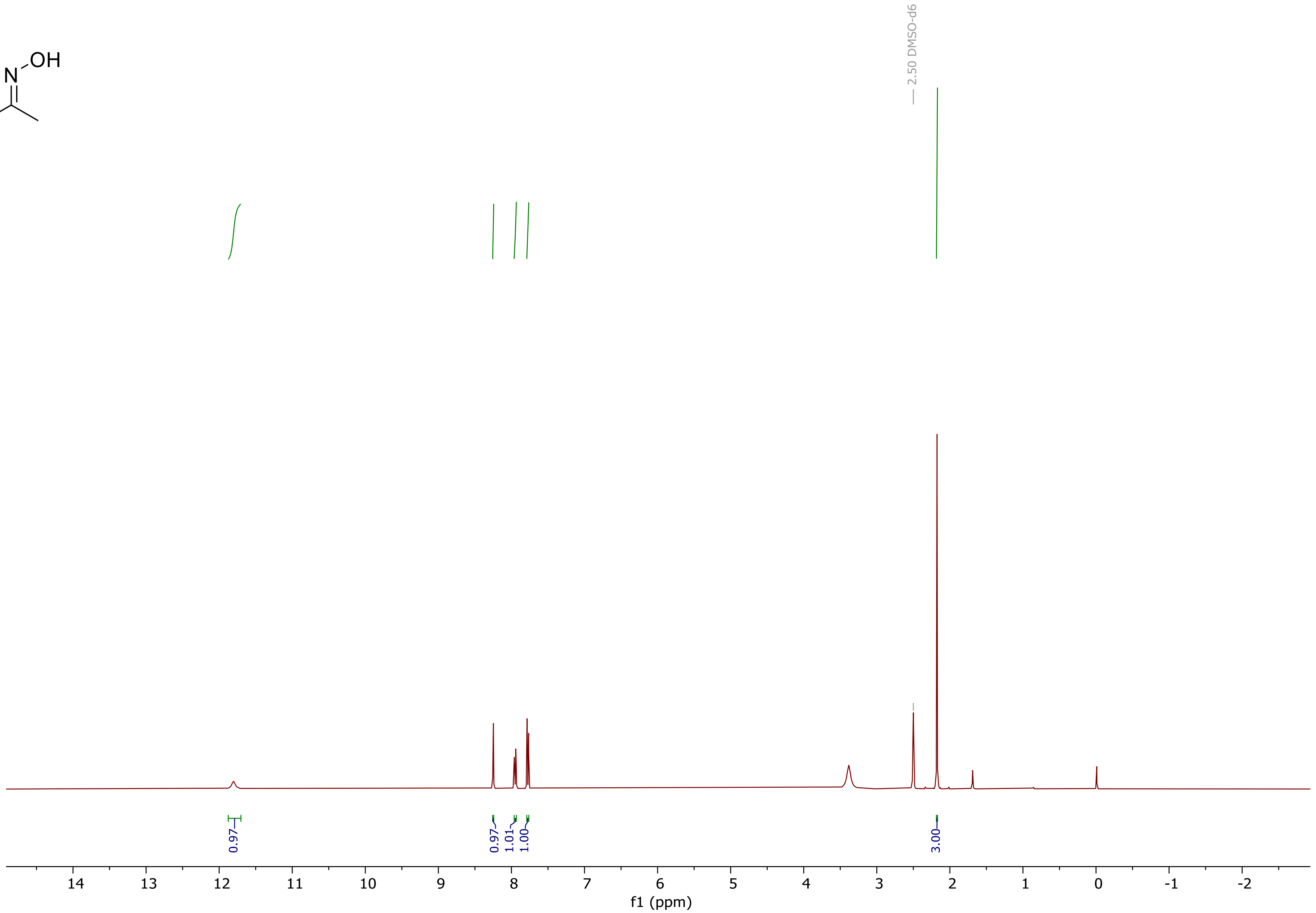

^{13}C NMR spectrum of **1k** (101 MHz, DMSO-d$_6$)

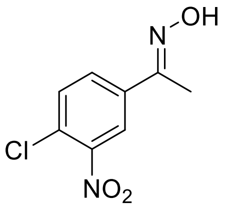

1k

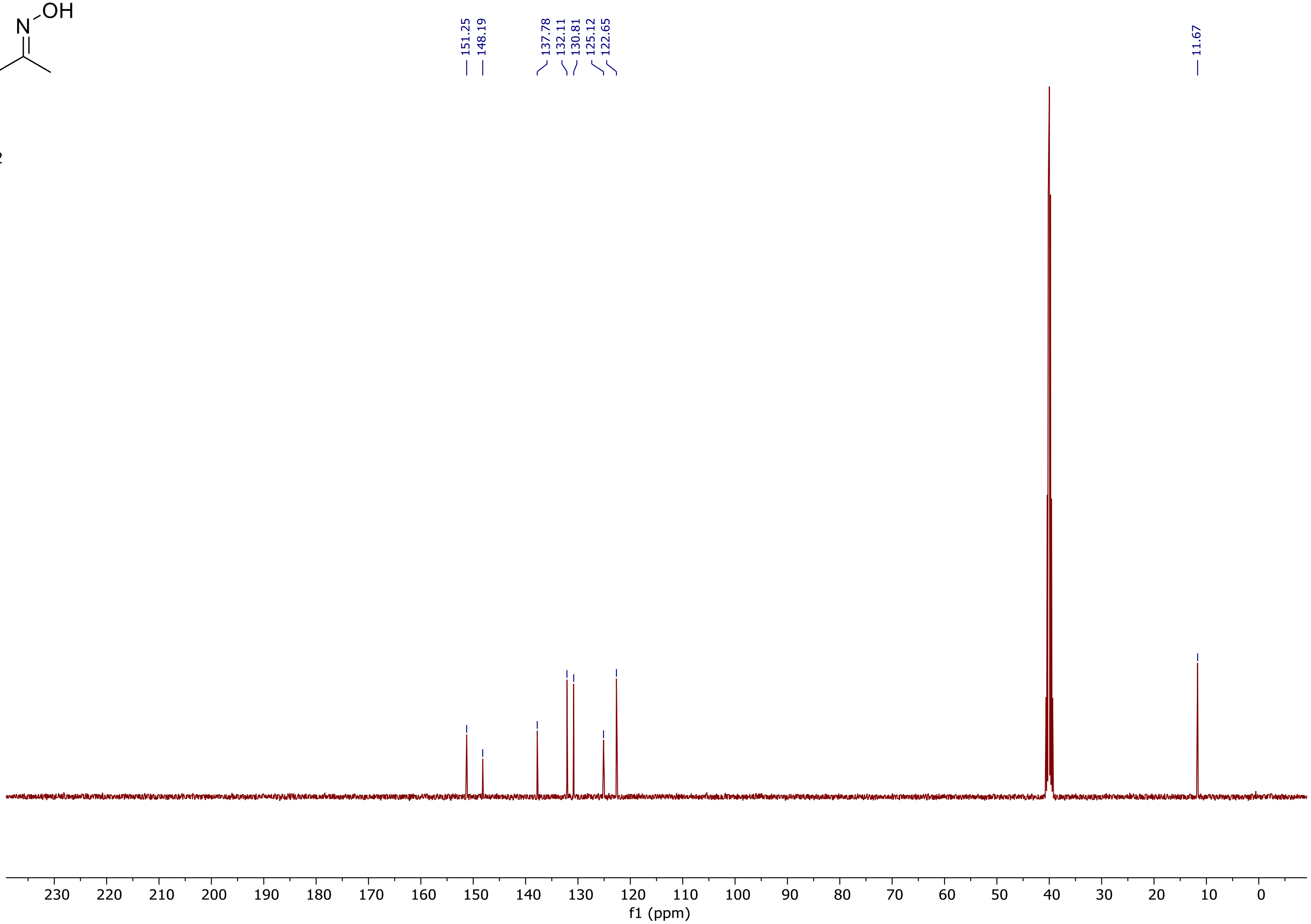

^1H NMR spectrum of **1l** (400 MHz, DMSO-d_6)

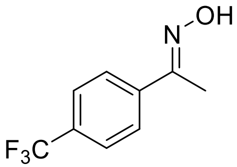

1l

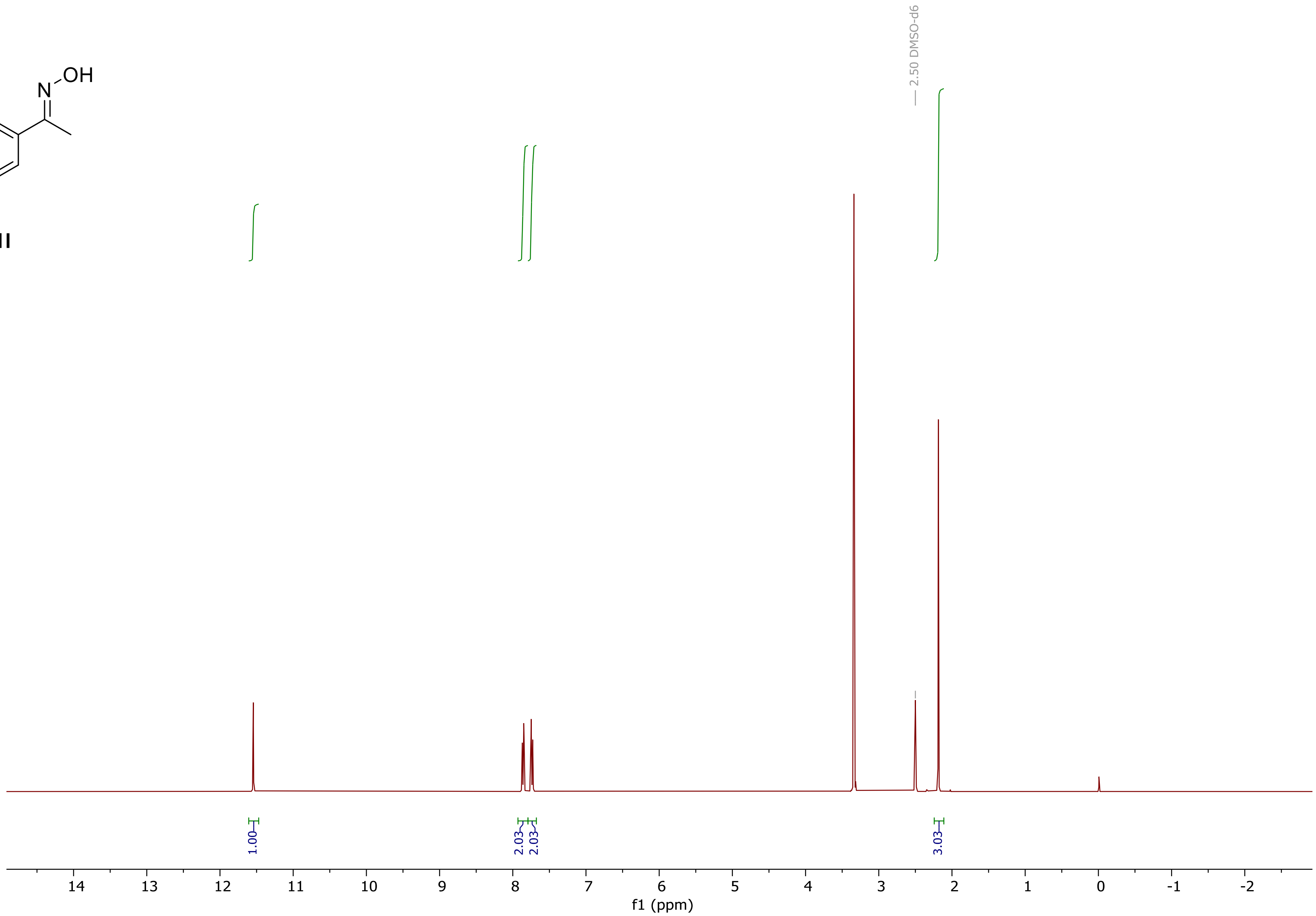

^{13}C NMR spectrum of **1l** (101 MHz, DMSO-d_6)

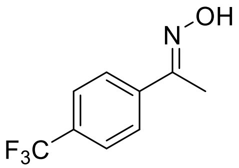

1l

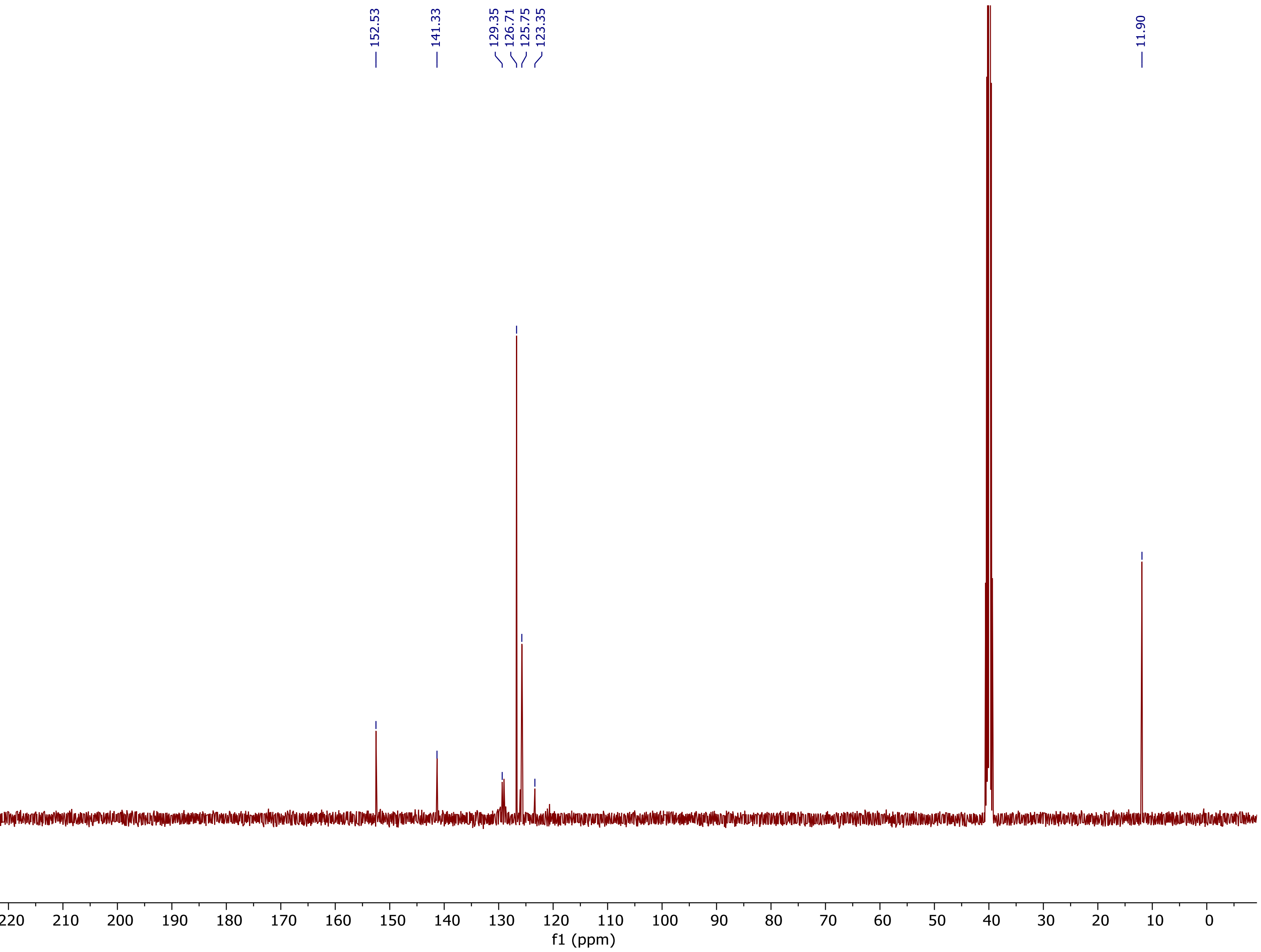

^{19}F NMR spectrum of **1l** (377 MHz, DMSO-d$_6$)

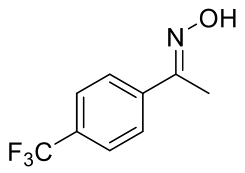

1l

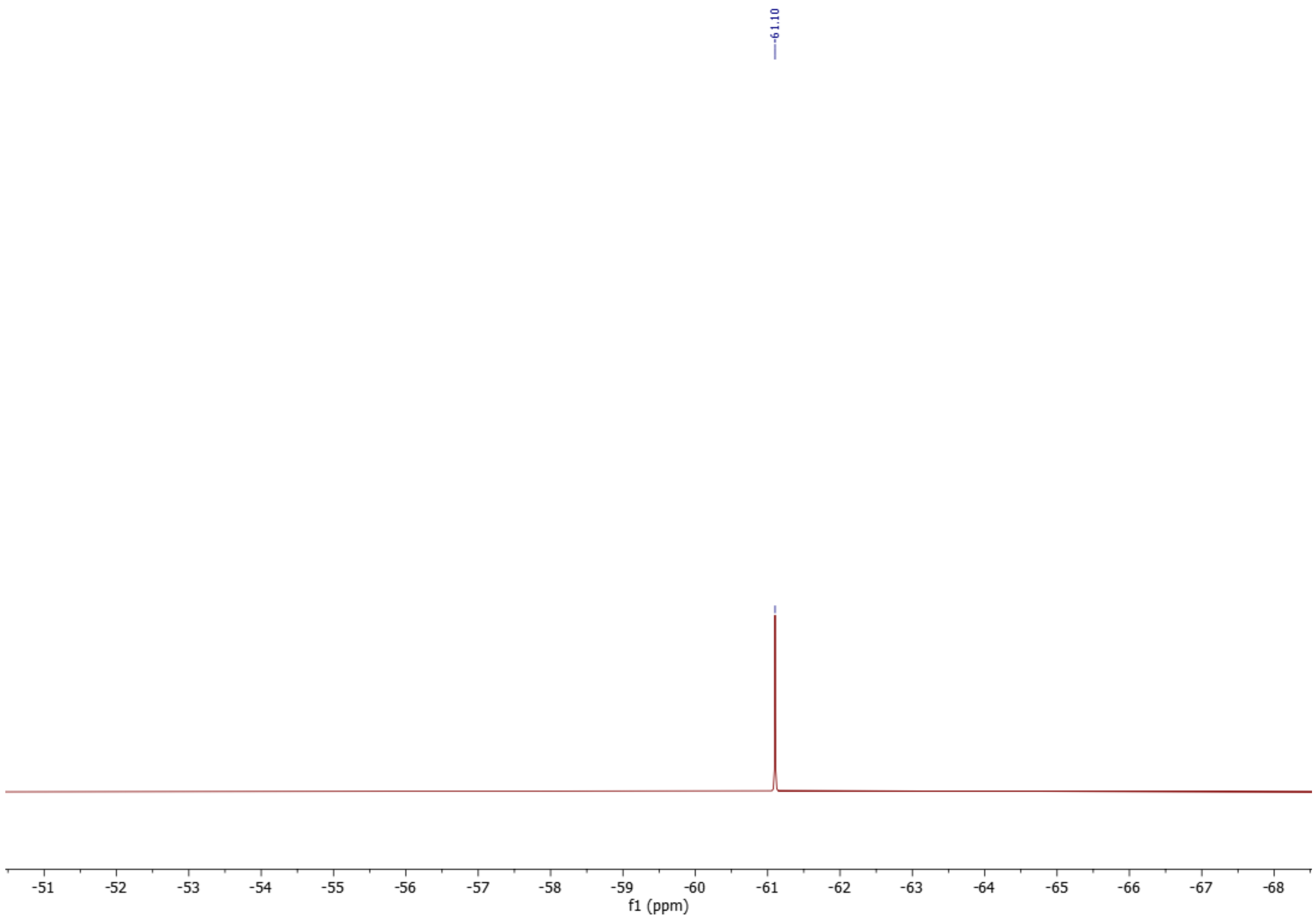

^{1}H NMR spectrum of **1m** (400 MHz, DMSO-d_6)

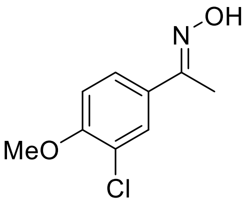

1m

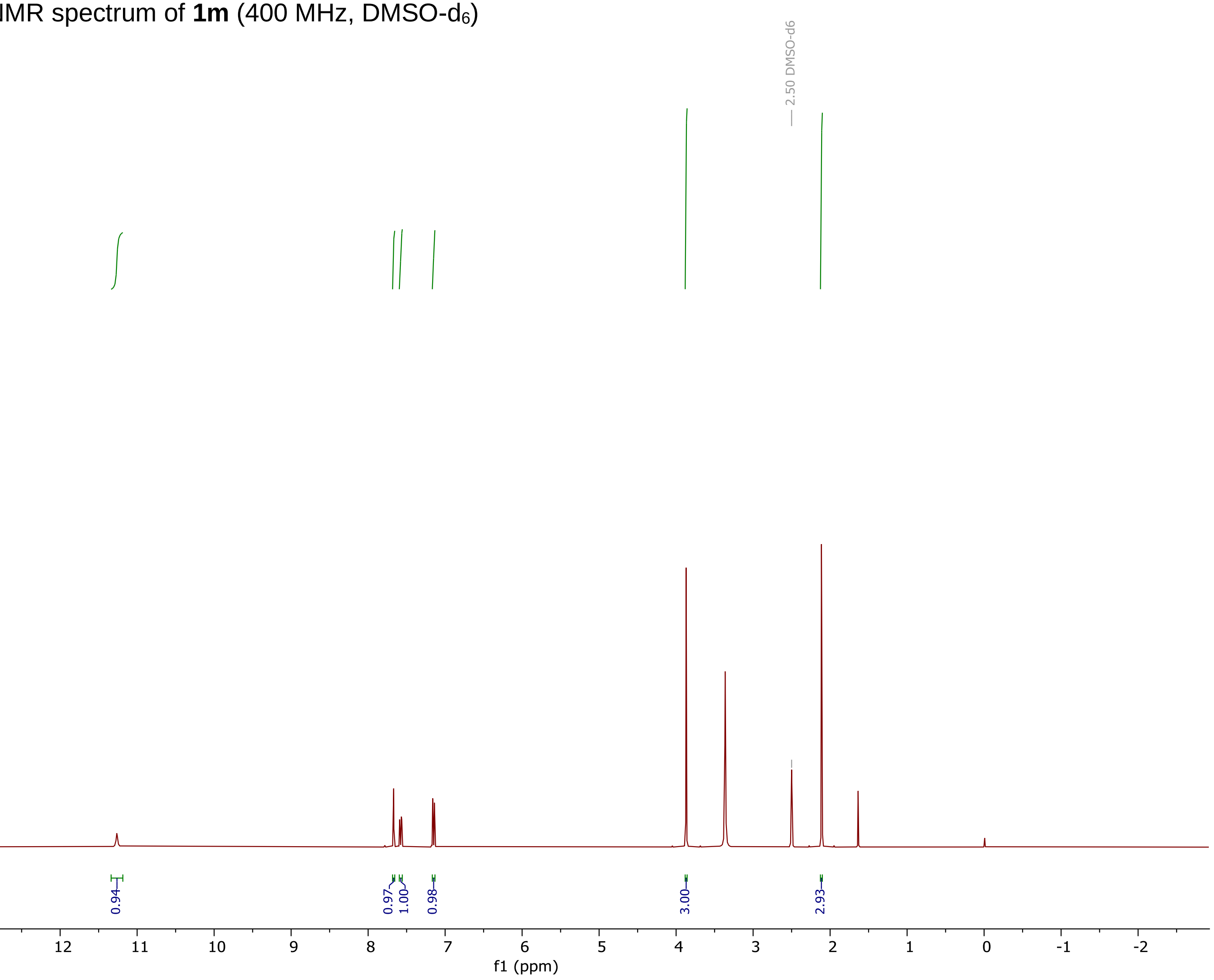

^{13}C NMR spectrum of **1m** (101 MHz, DMSO-d$_6$)

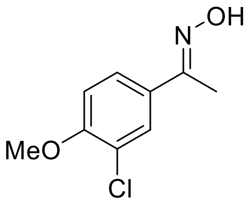

1m

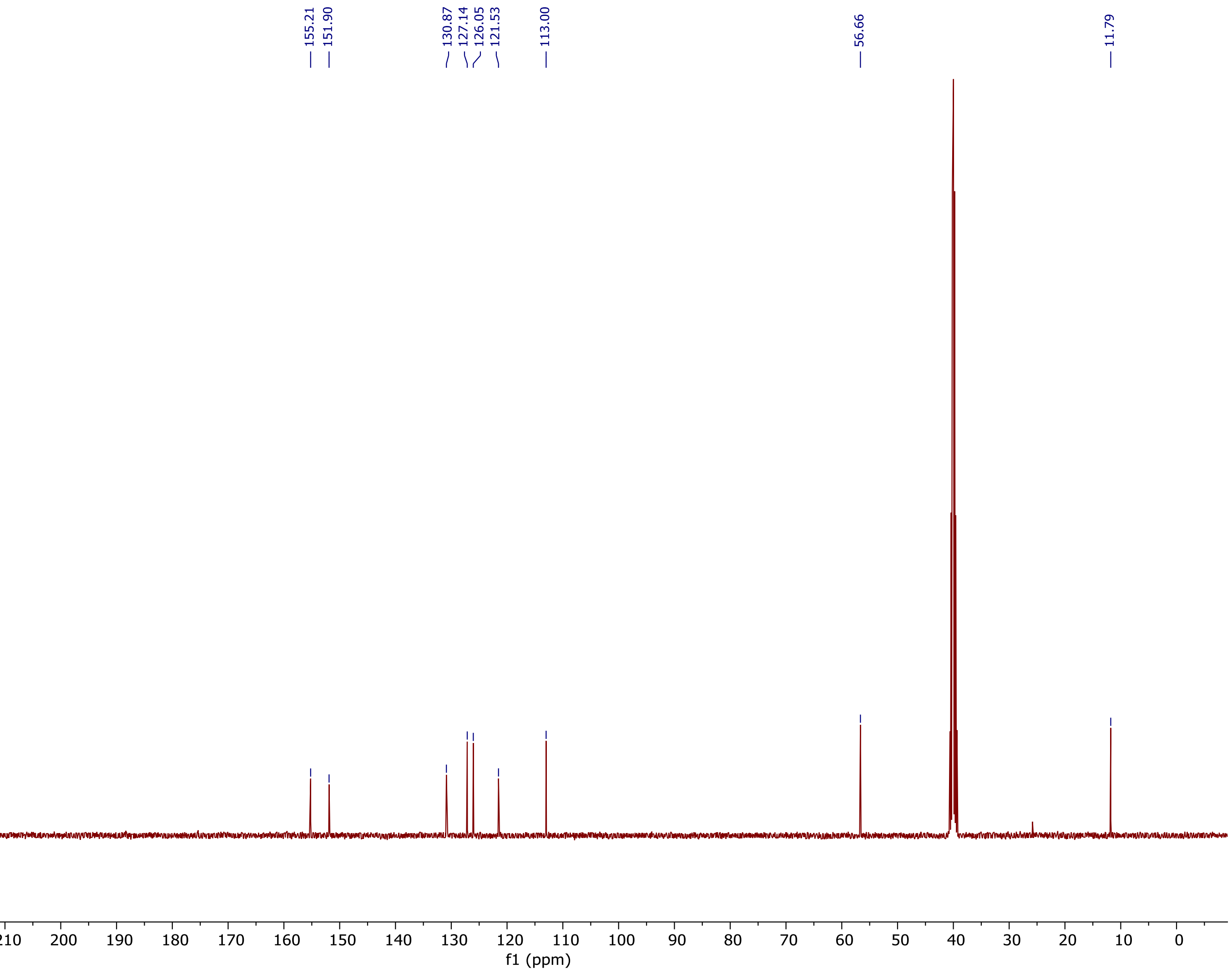

^{1}H NMR spectrum of **1n** (400 MHz, DMSO-d$_6$)

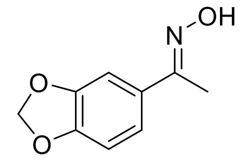

1n

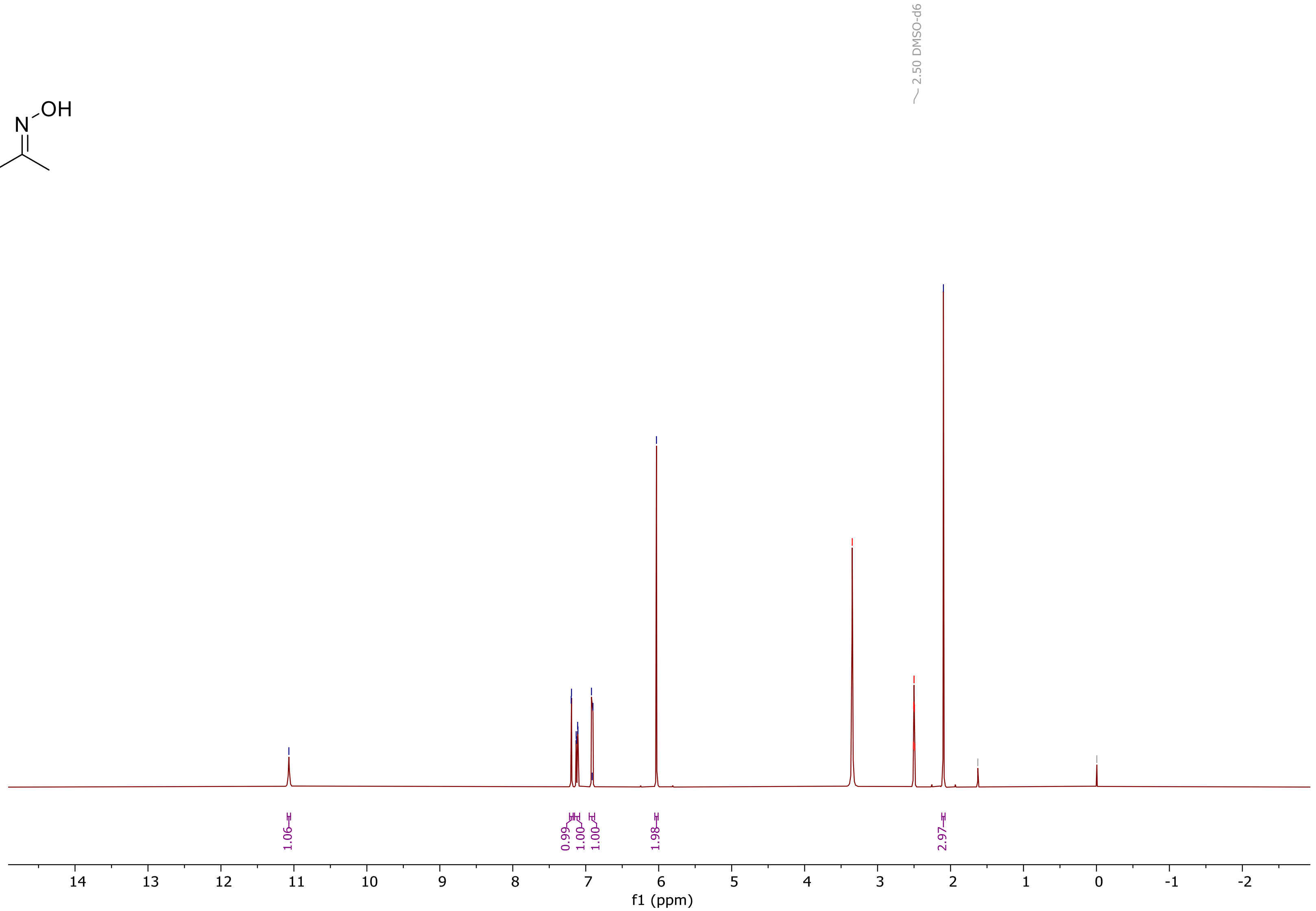

^{13}C NMR spectrum of **1n** (101 MHz, DMSO-d$_6$)

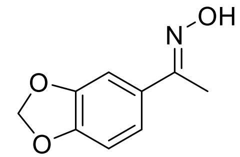

1n

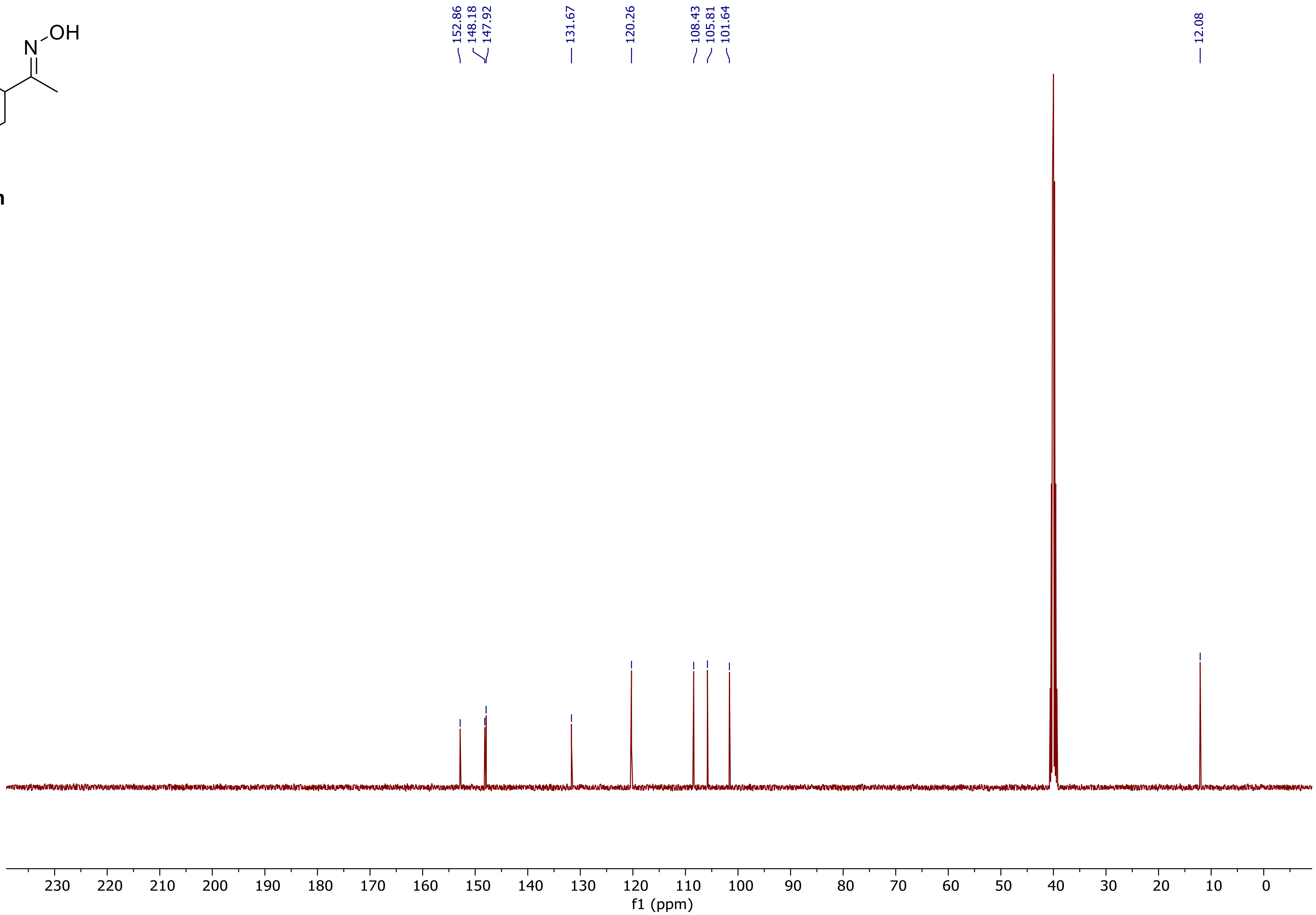

H NMR spectrum of **2a** (400 MHz, DMSO-d_6)

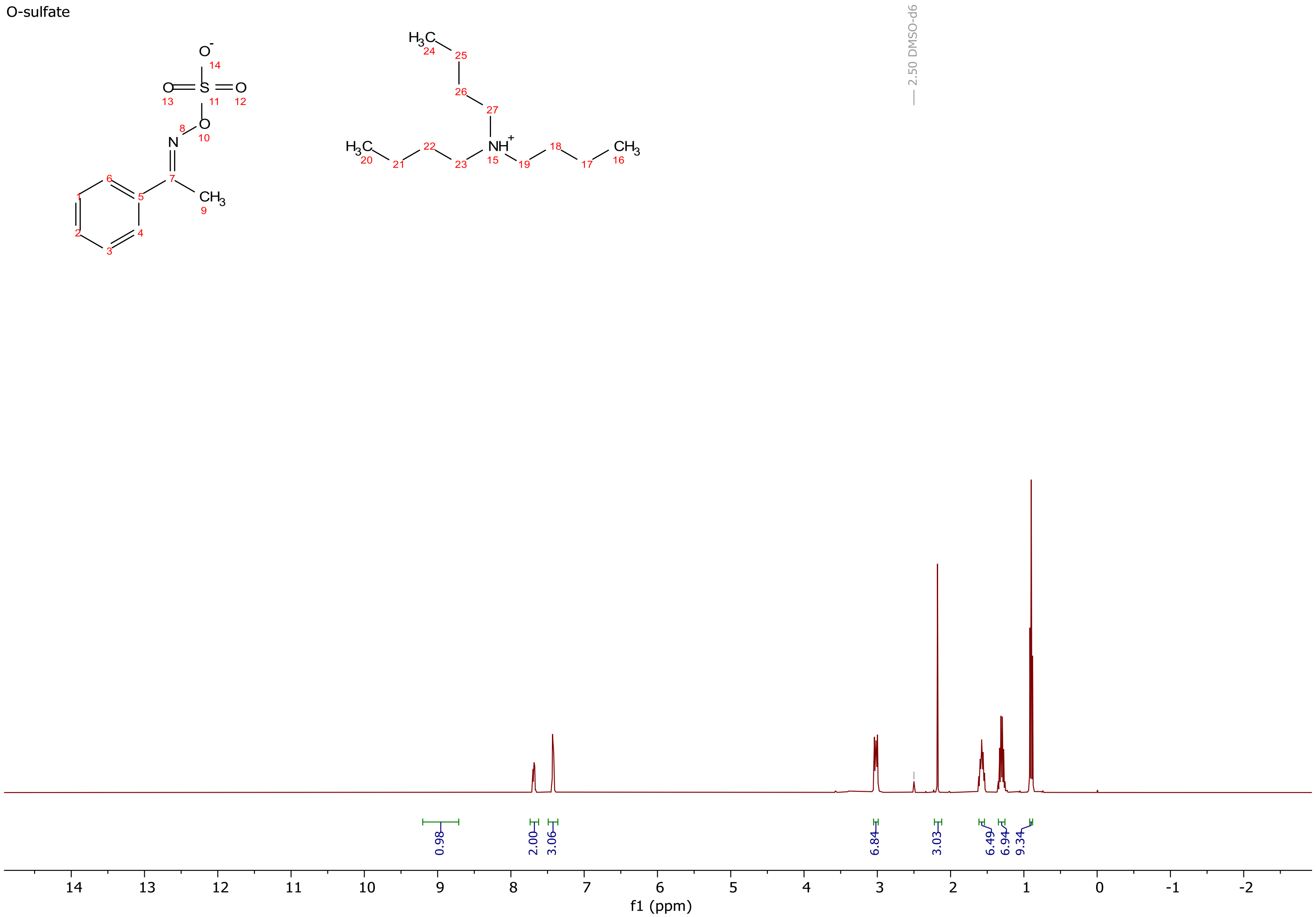

^{13}C NMR spectrum of **2a** (101 MHz, DMSO-d$_6$)

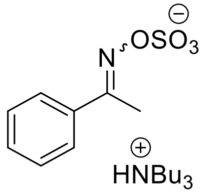

2a

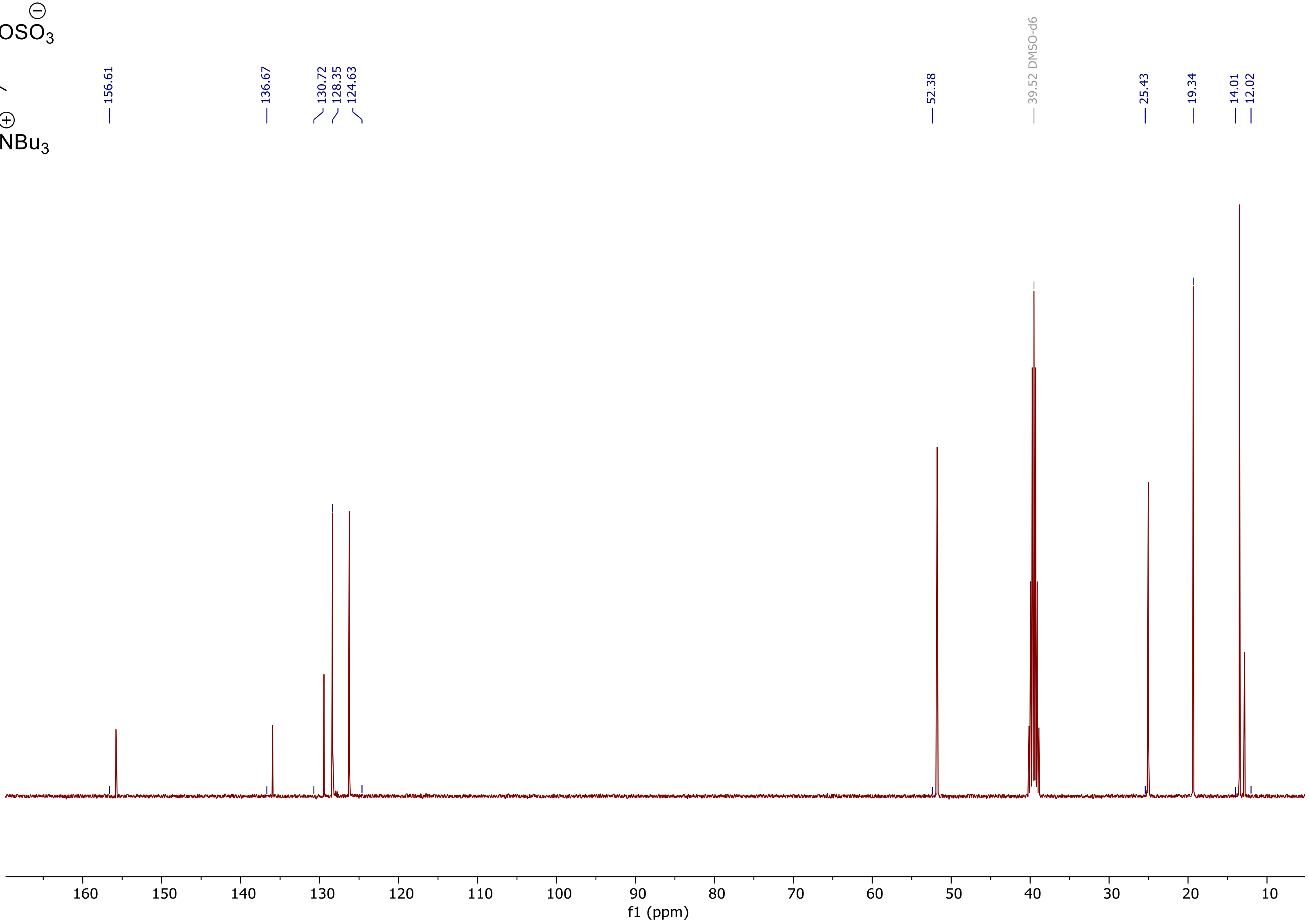

^{1}H NMR spectrum of **2a[Na]** (400 MHz, D_2O)

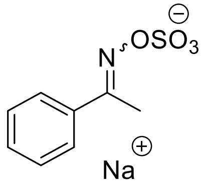

2a[Na]

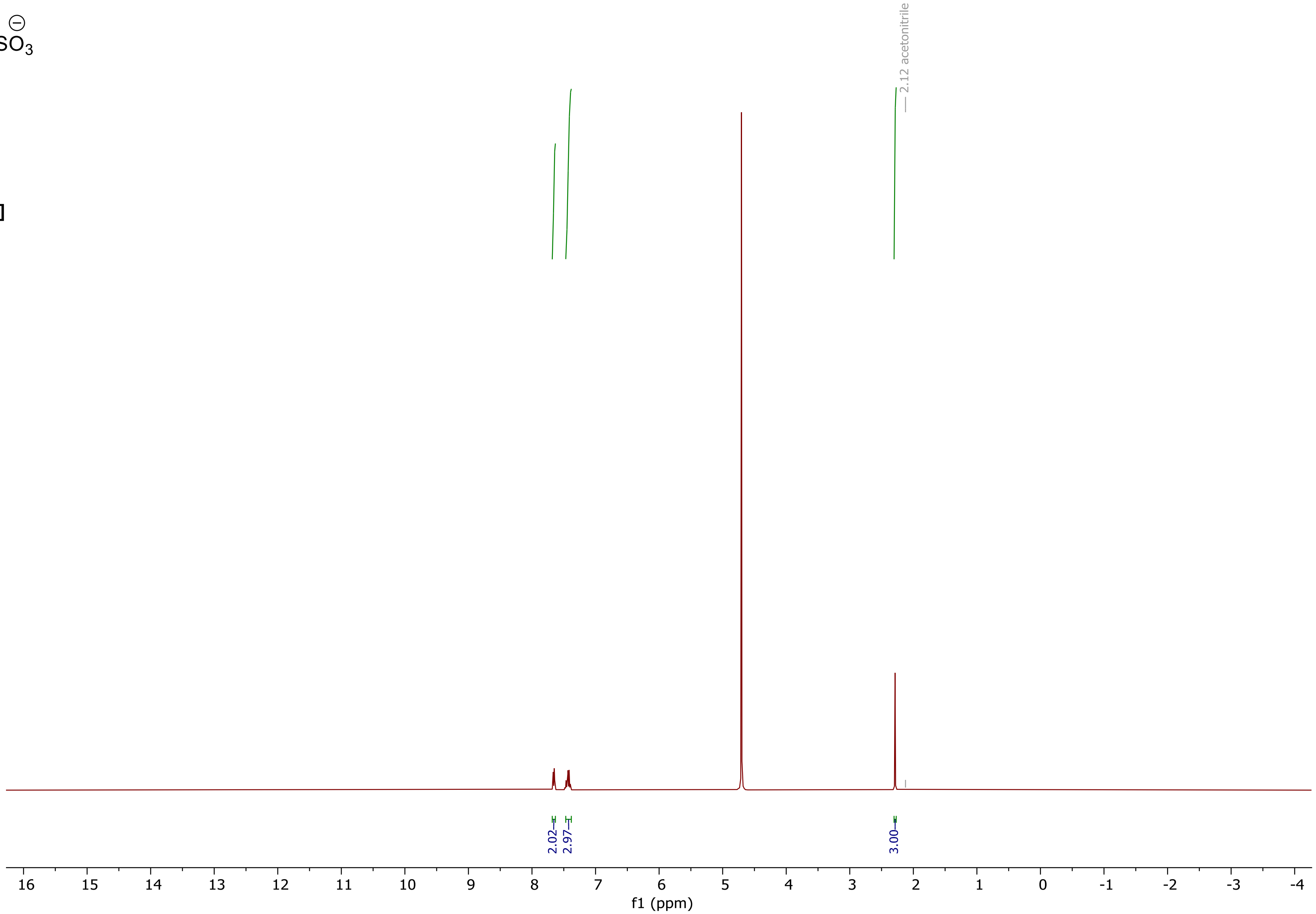

^{13}C NMR spectrum of **2a[Na]** (101 MHz, D_2O)

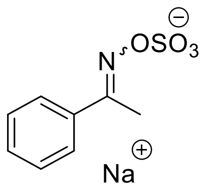

2a[Na]

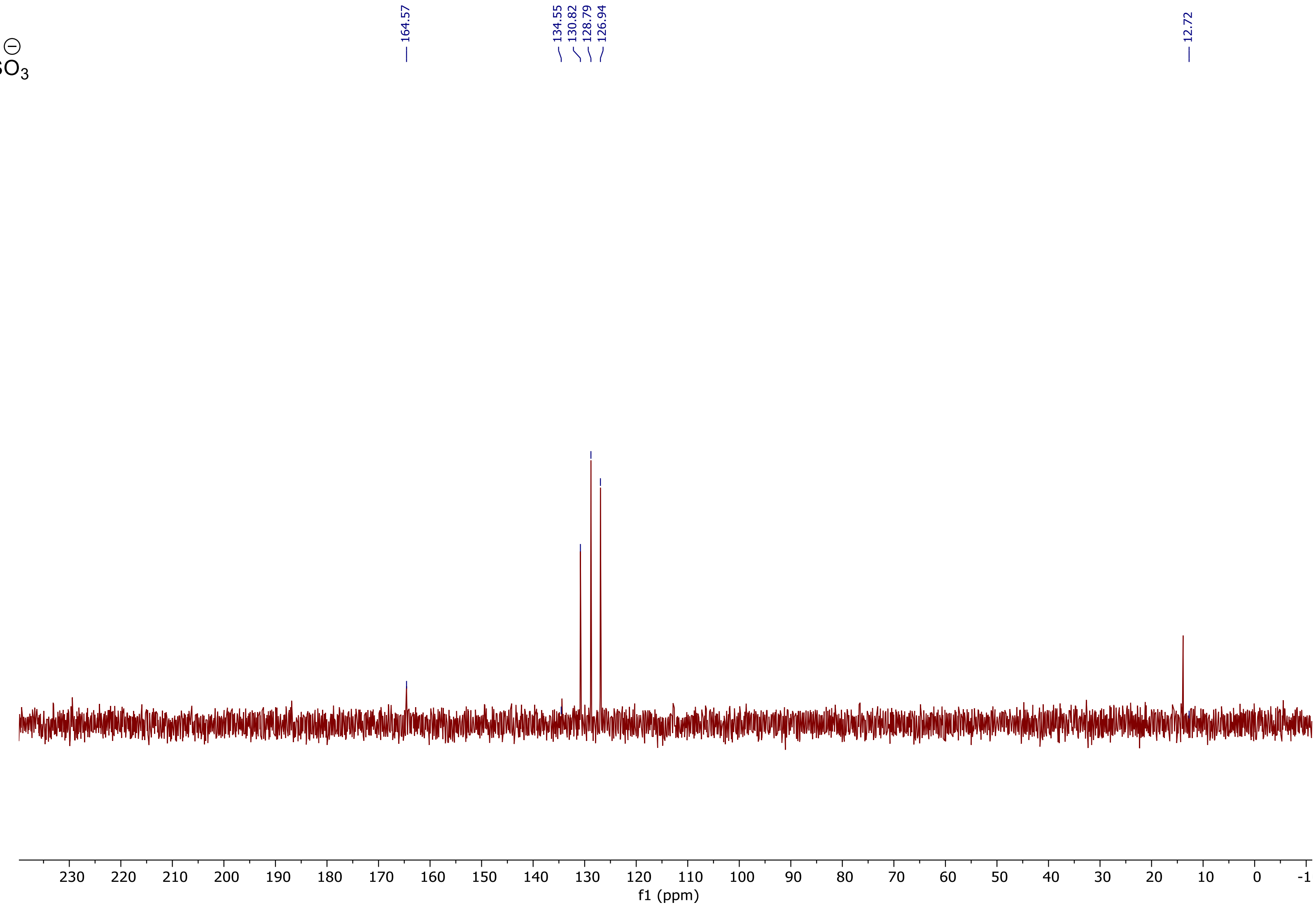

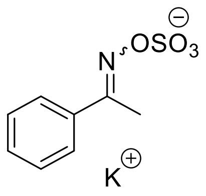
N
OSO3
K

2a[K]

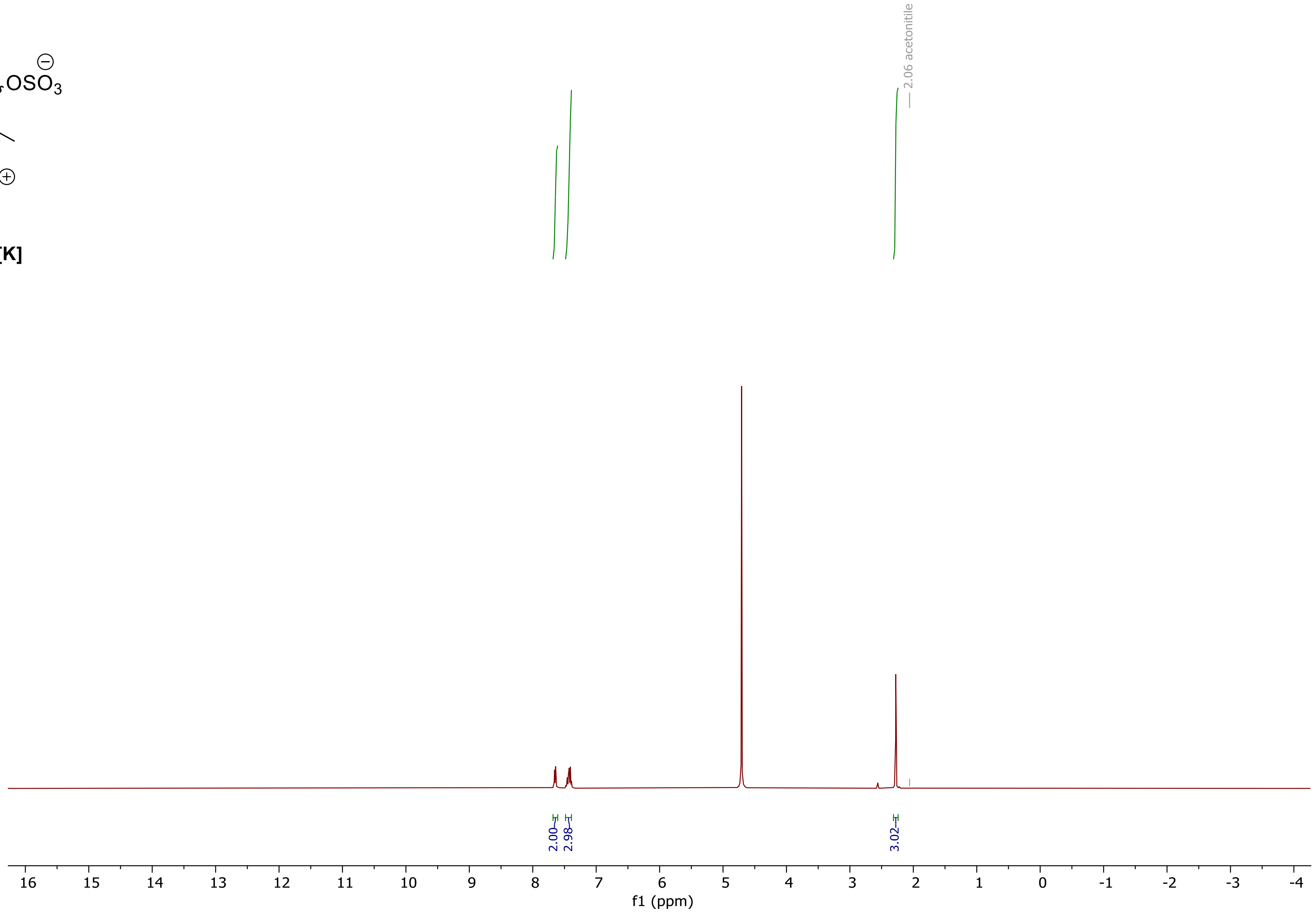
2.06 acetonitile
2.00
2.98
3.02
16
15
14
13
12
11
10
9
8
7
6
5
4
3
2
1
0
-1
-2
-3
-4
f1 (ppm)

^{13}C NMR spectrum of **2a[K]** (101 MHz, D_2O)

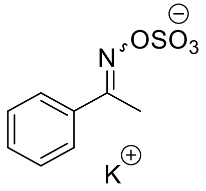

2a[K]

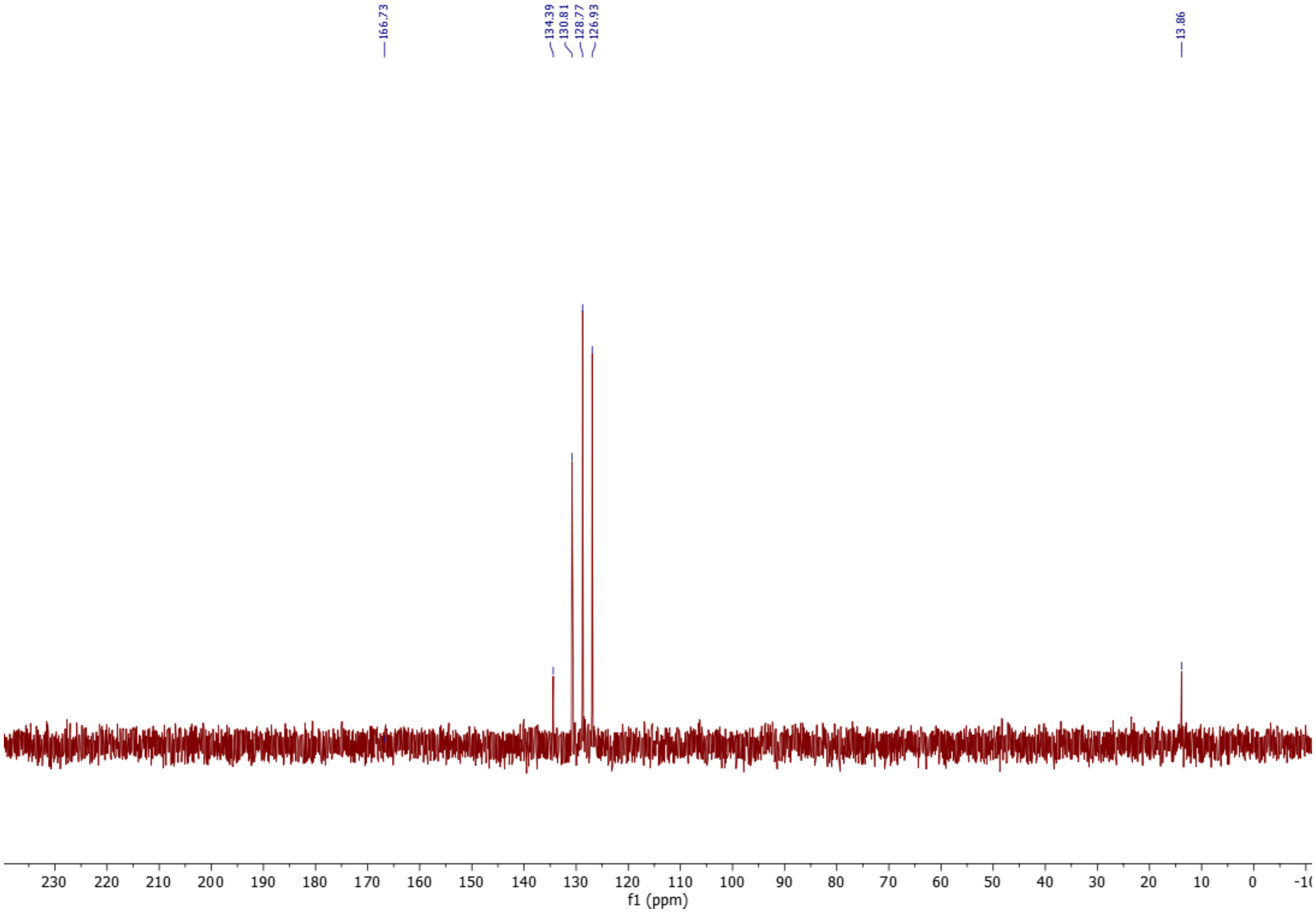

^{1}H NMR spectrum of **2b** (400 MHz, DMSO-d_6)

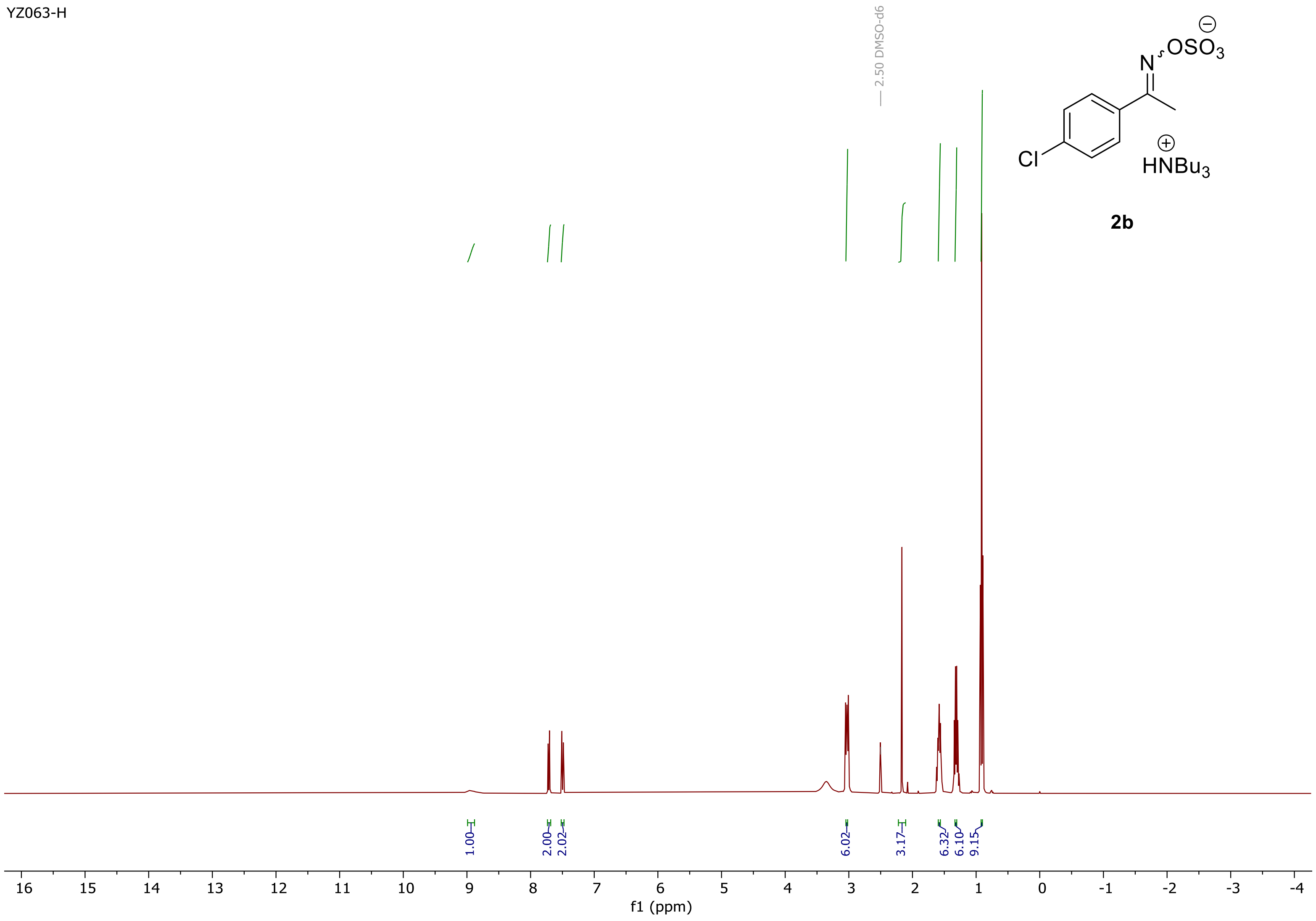

^{13}C NMR spectrum of **2b** (101 MHz, DMSO-d$_6$)

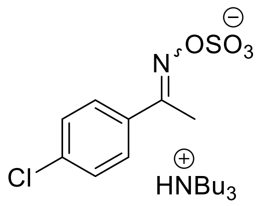

2b

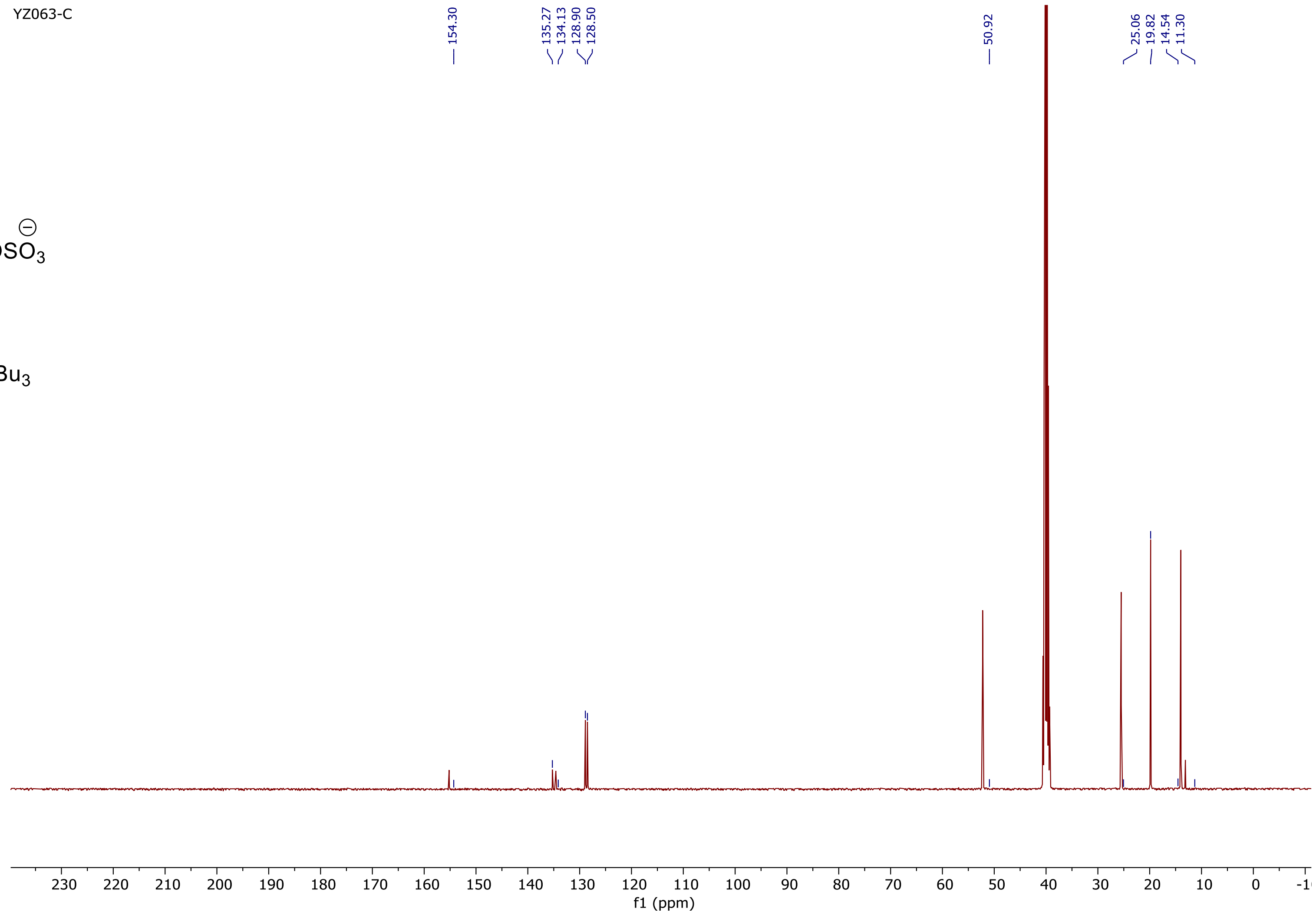

^{1}H NMR spectrum of **2c** (400 MHz, DMSO-d$_6$)

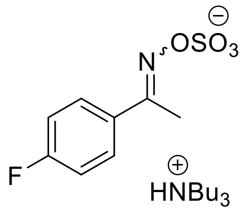

2c

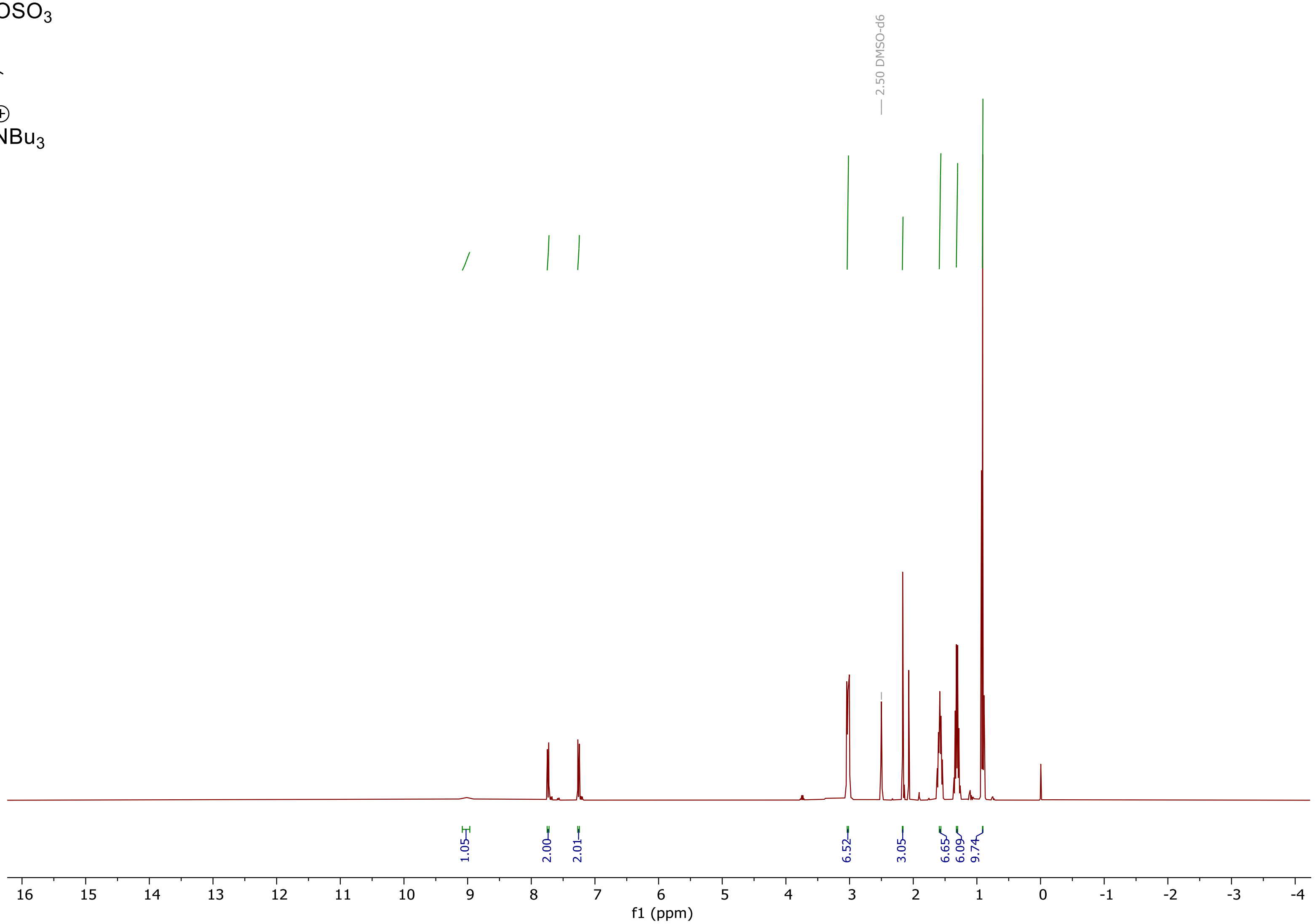

^{13}C NMR spectrum of **2c** (101 MHz, DMSO-d$_6$)

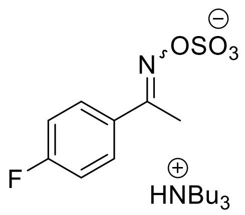

2c

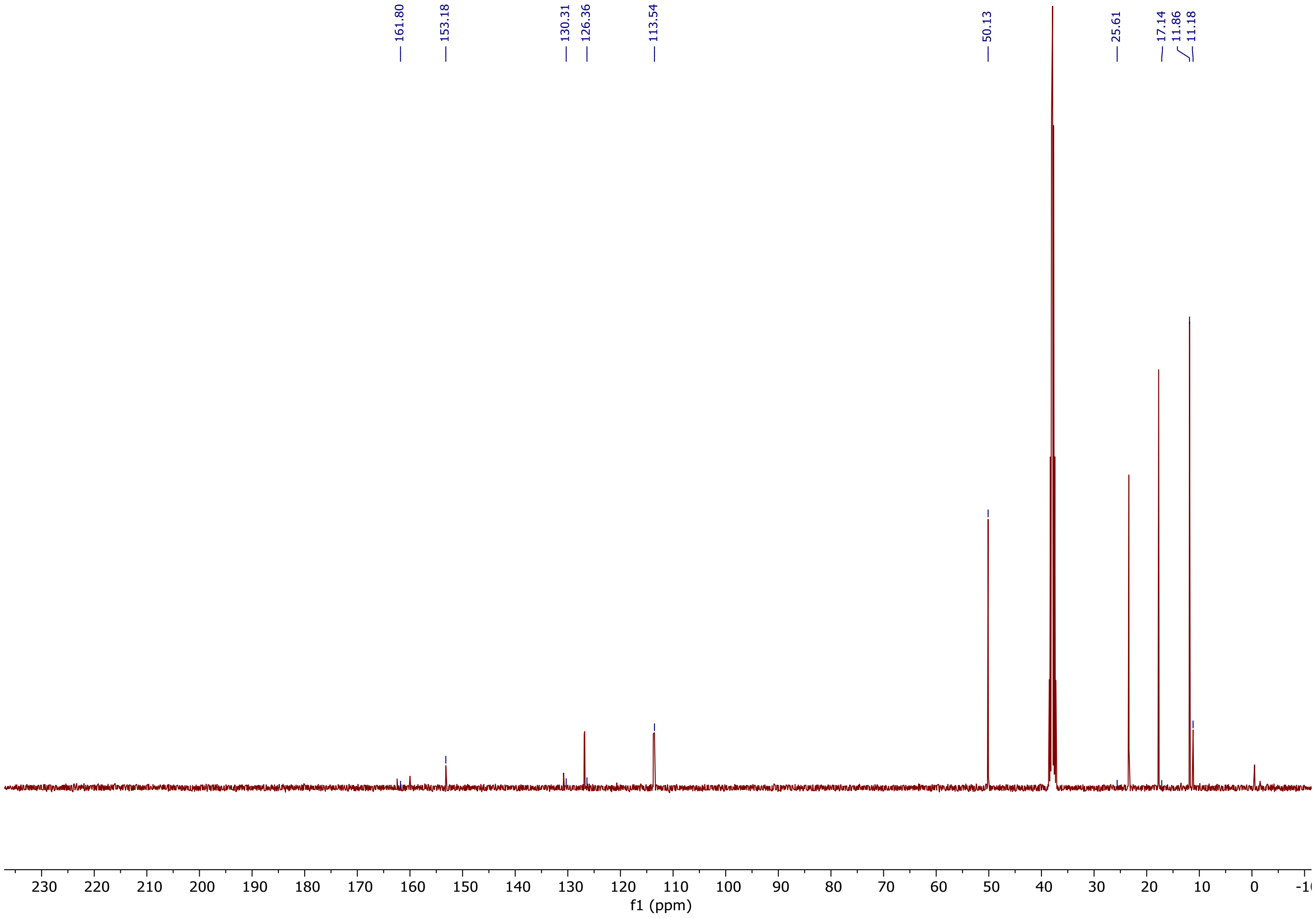

^{19}F NMR spectrum of **2c** (377 MHz, DMSO-d$_6$)

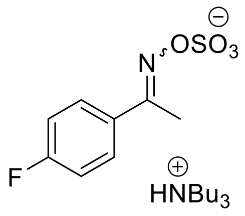

2c

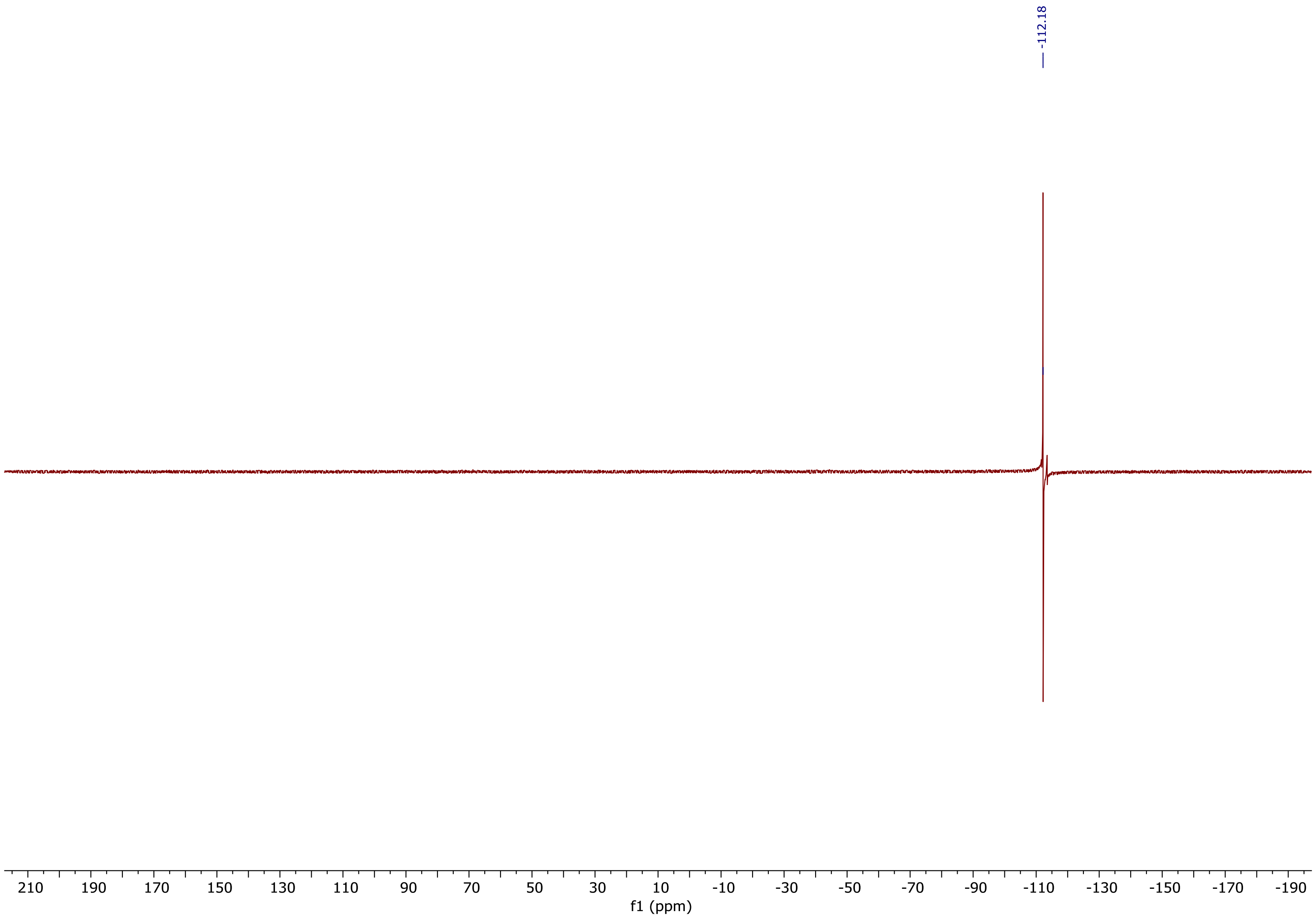

^{1}H NMR spectrum of **2d** (400 MHz, DMSO-d$_6$)

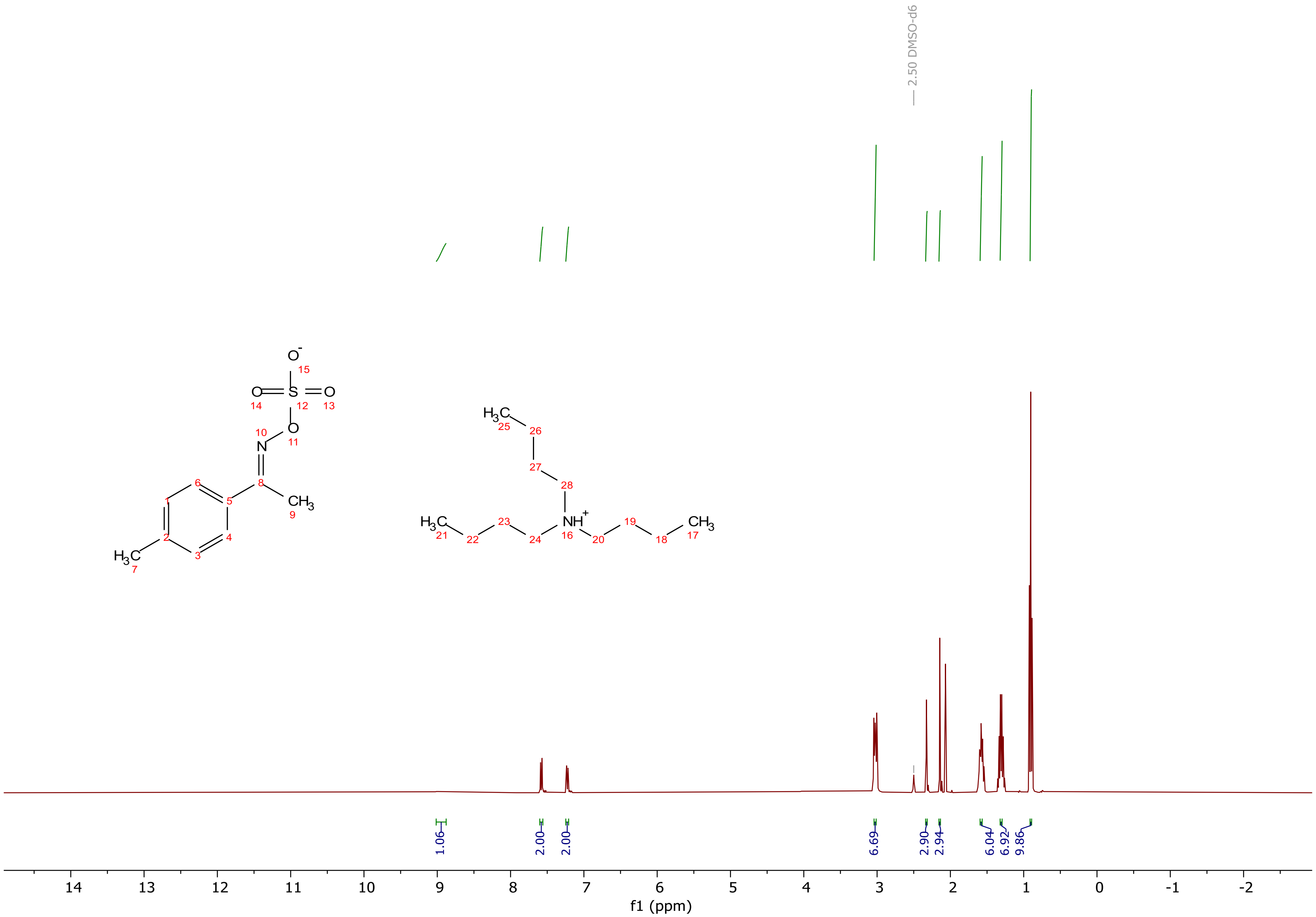

^{13}C NMR spectrum of **2d** (101 MHz, DMSO-d_6)

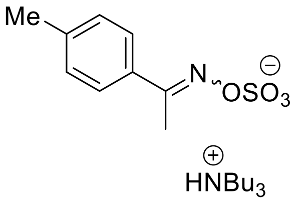

2d

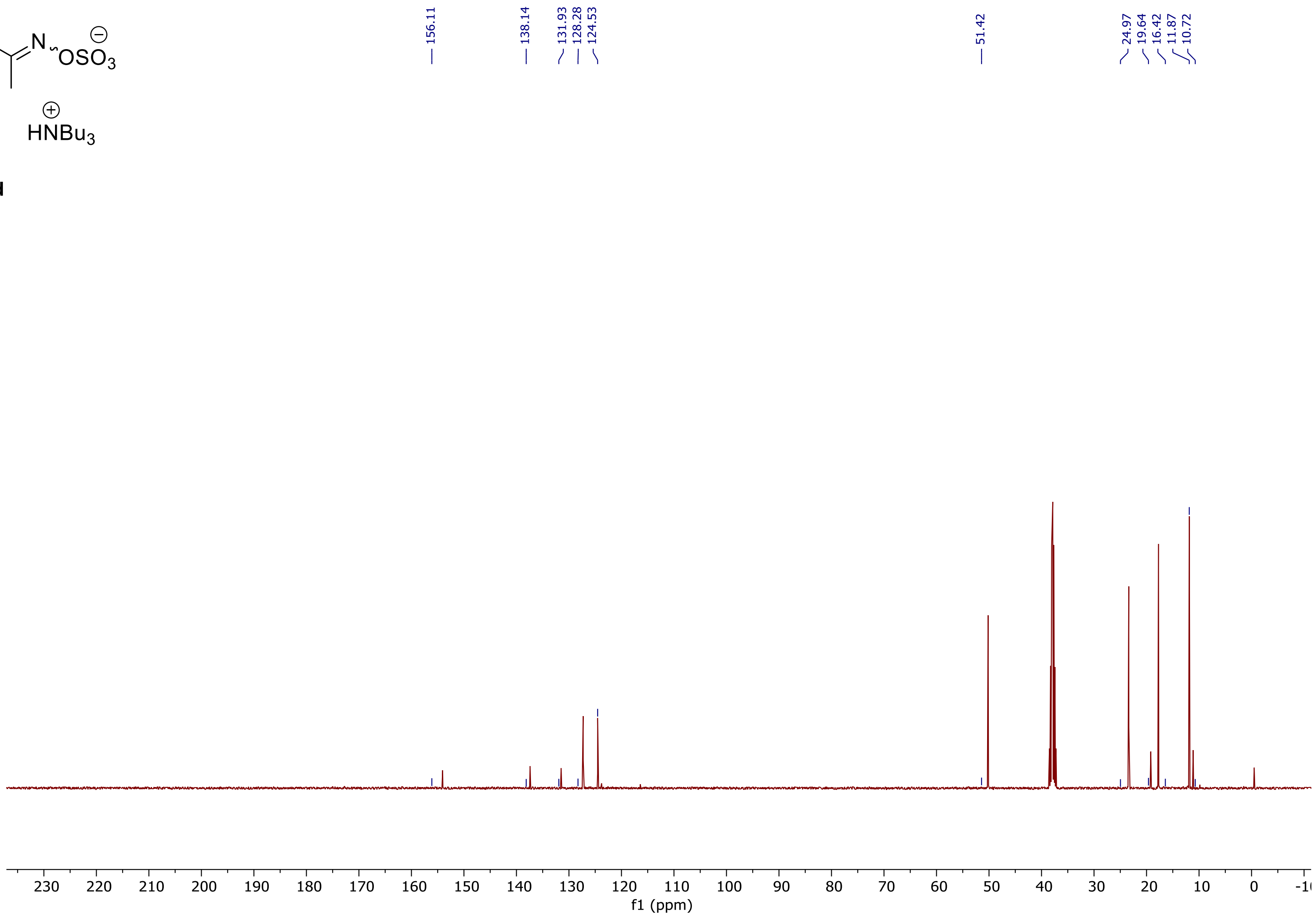

^1H NMR spectrum of **2e** (400 MHz, DMSO-d_6)

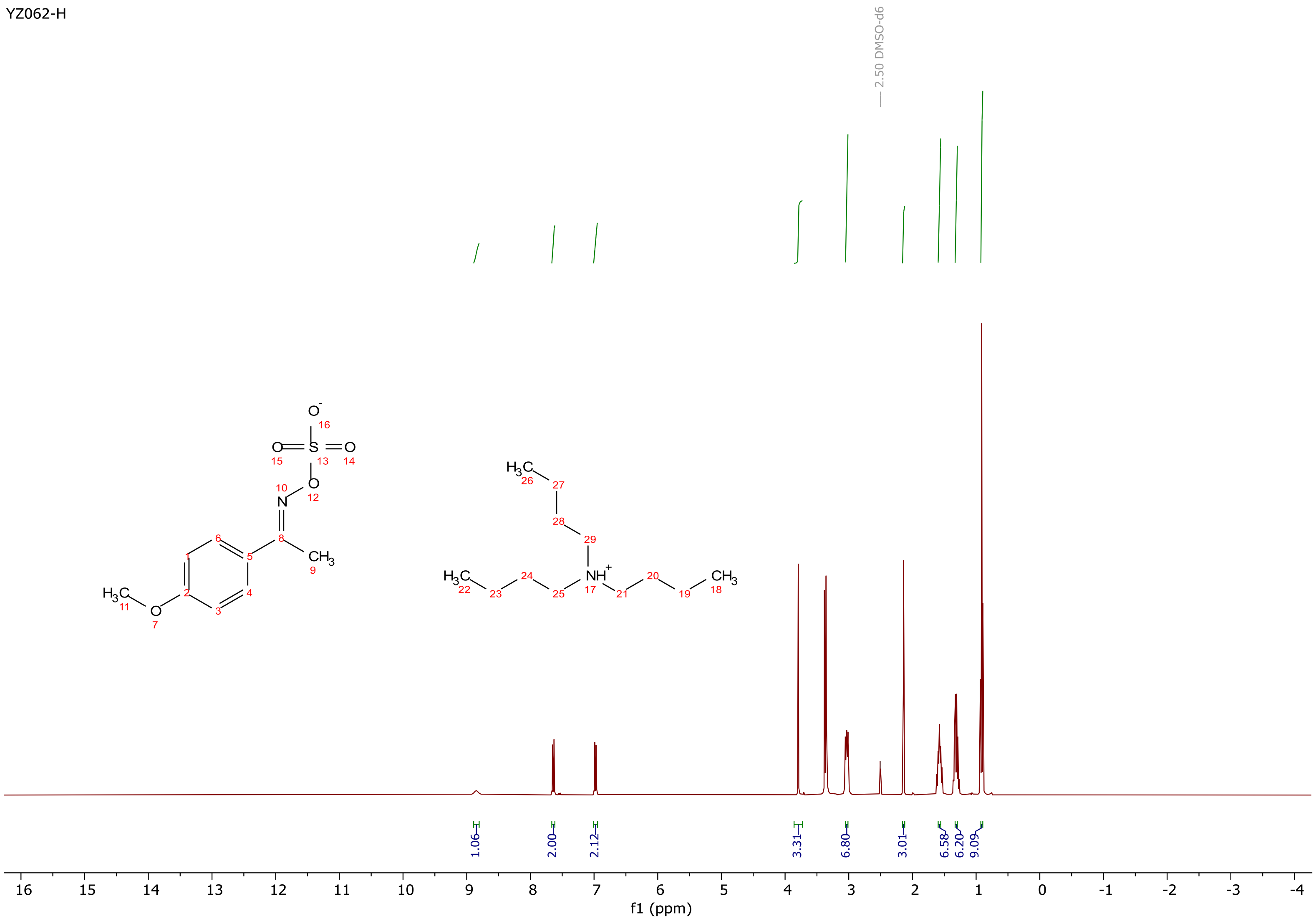

^{13}C NMR spectrum of **2e** (101 MHz, DMSO-d$_6$)

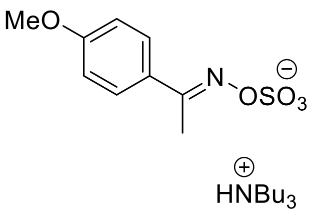

2e

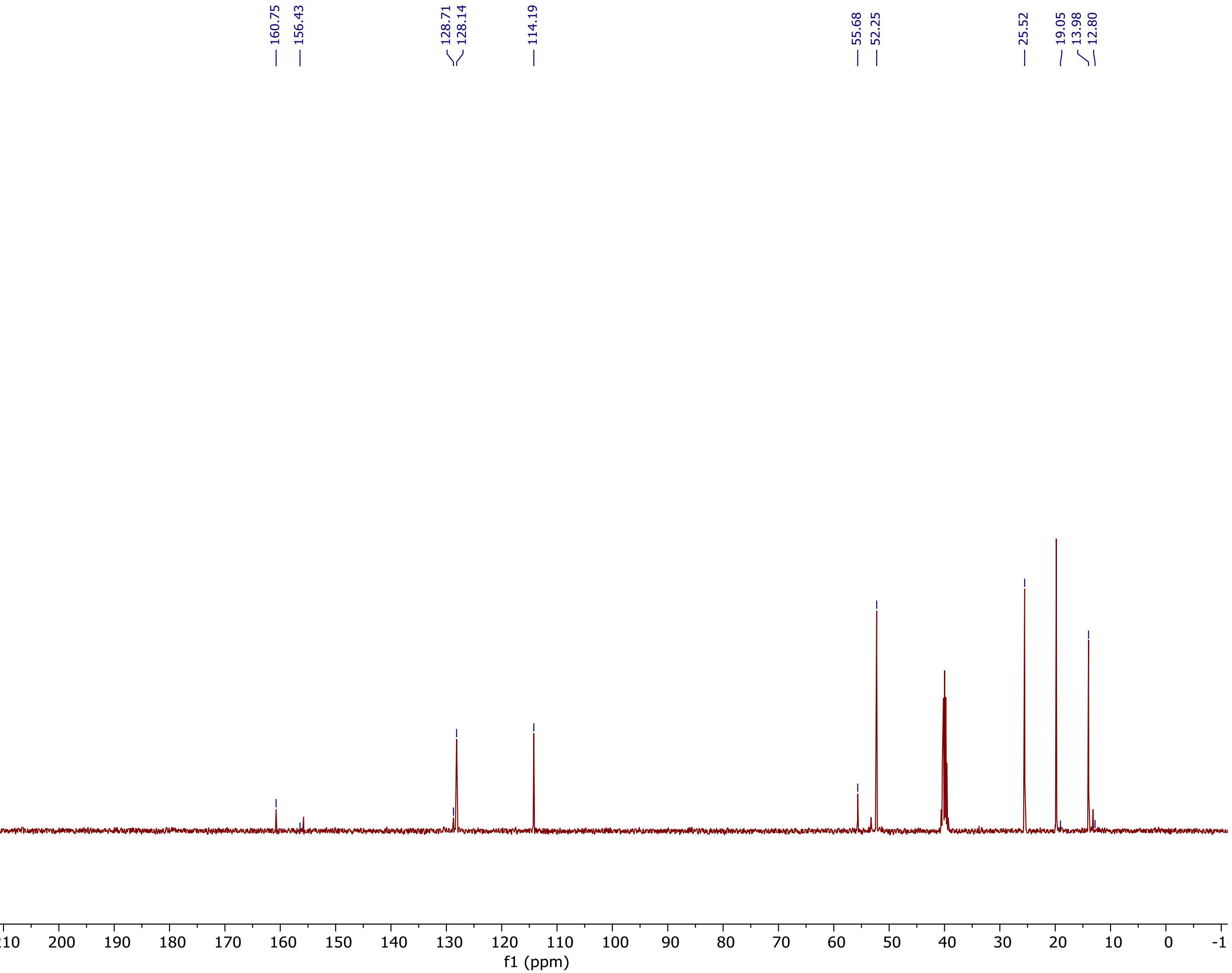

^{1}H NMR spectrum of **2f** (400 MHz, DMSO-d6)

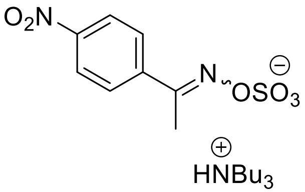

2f

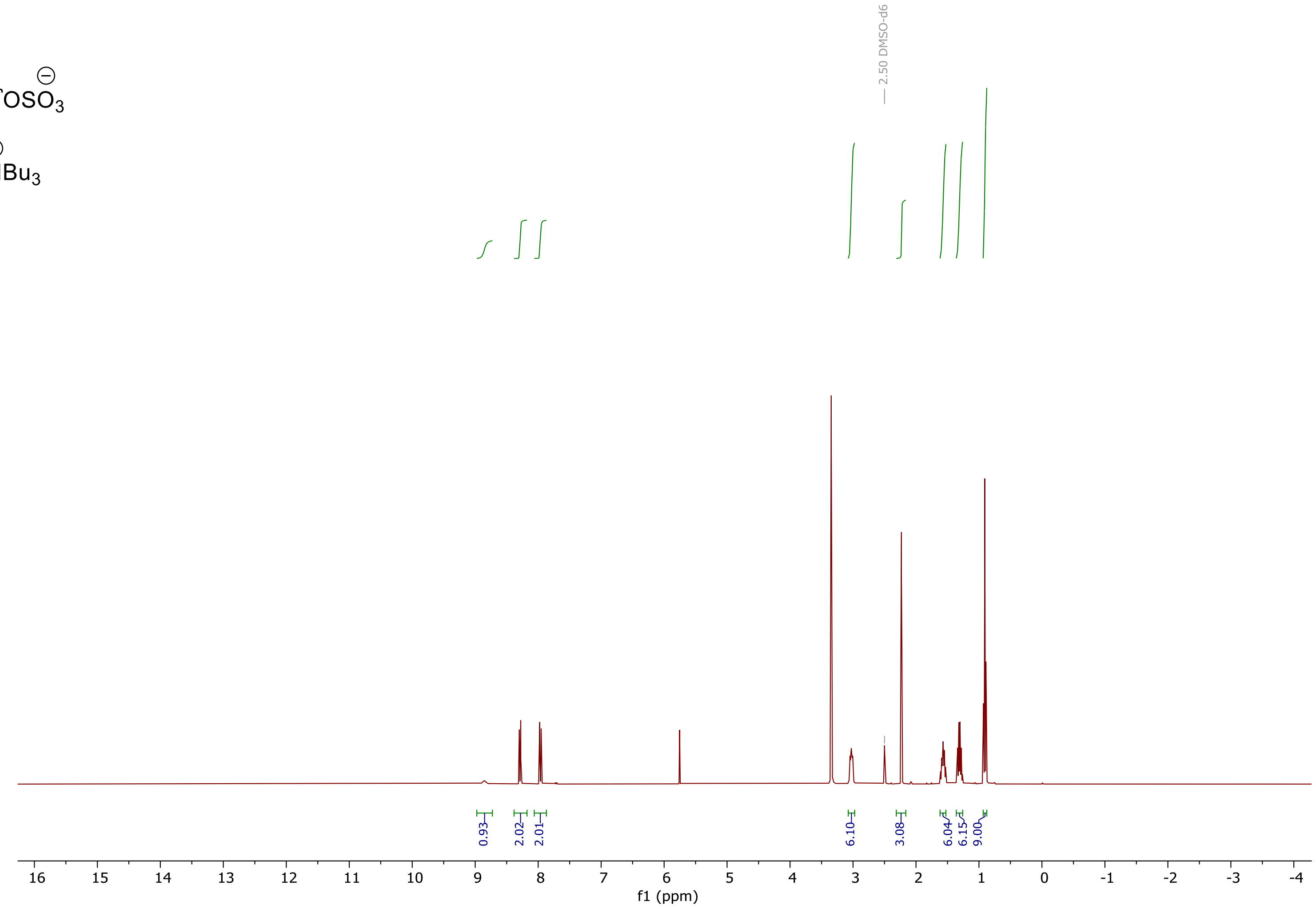

^{13}C NMR spectrum of **2f** (101 MHz, DMSO-d$_6$)

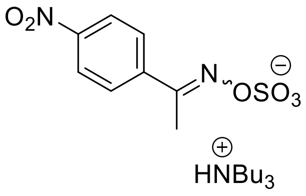

2f

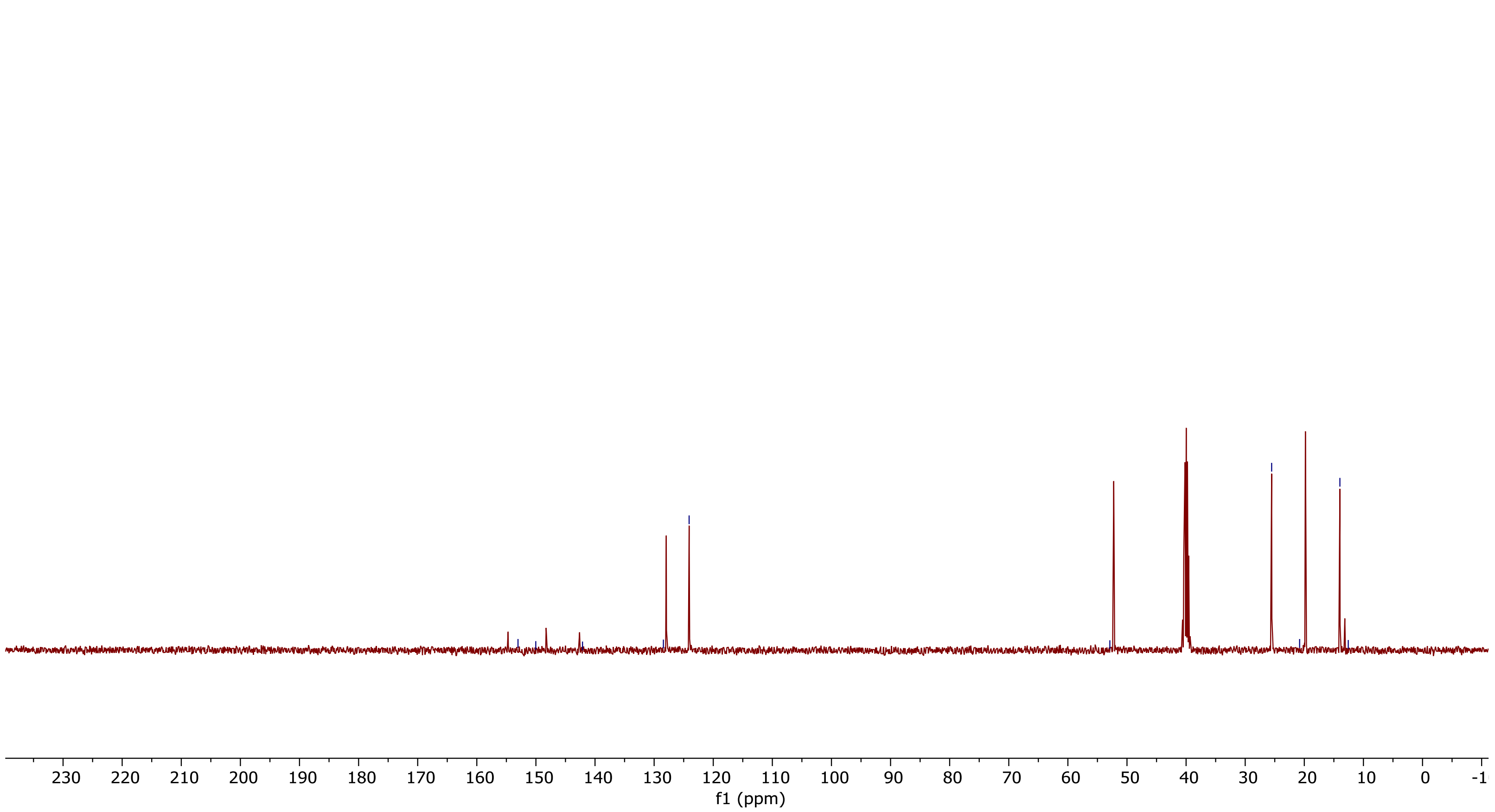

^1H NMR spectrum of **2g** (400 MHz, DMSO-d_6)

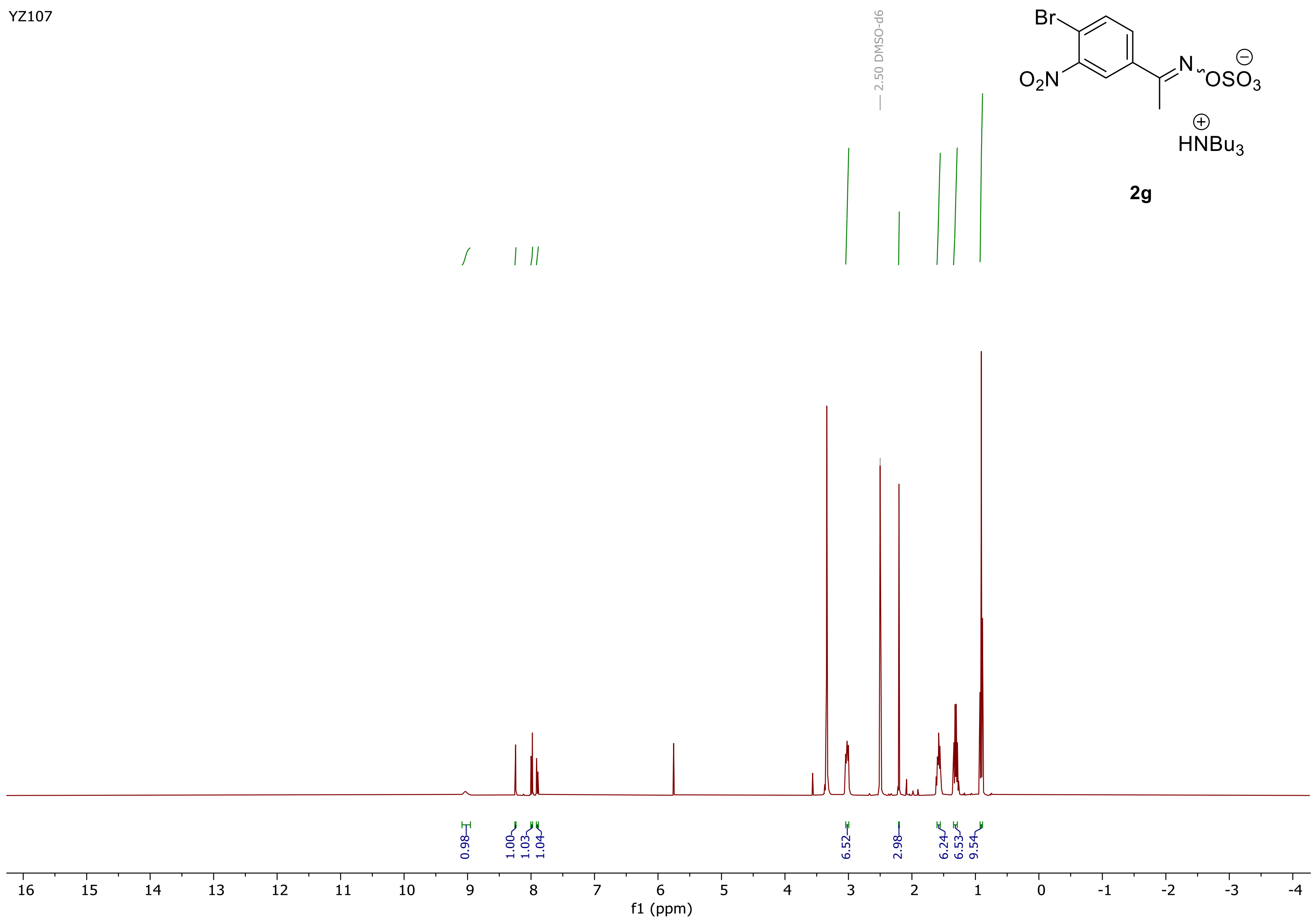

^{13}C NMR spectrum of **2g** (101 MHz, DMSO-d$_6$)

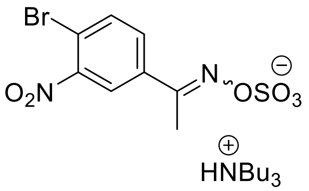

2g

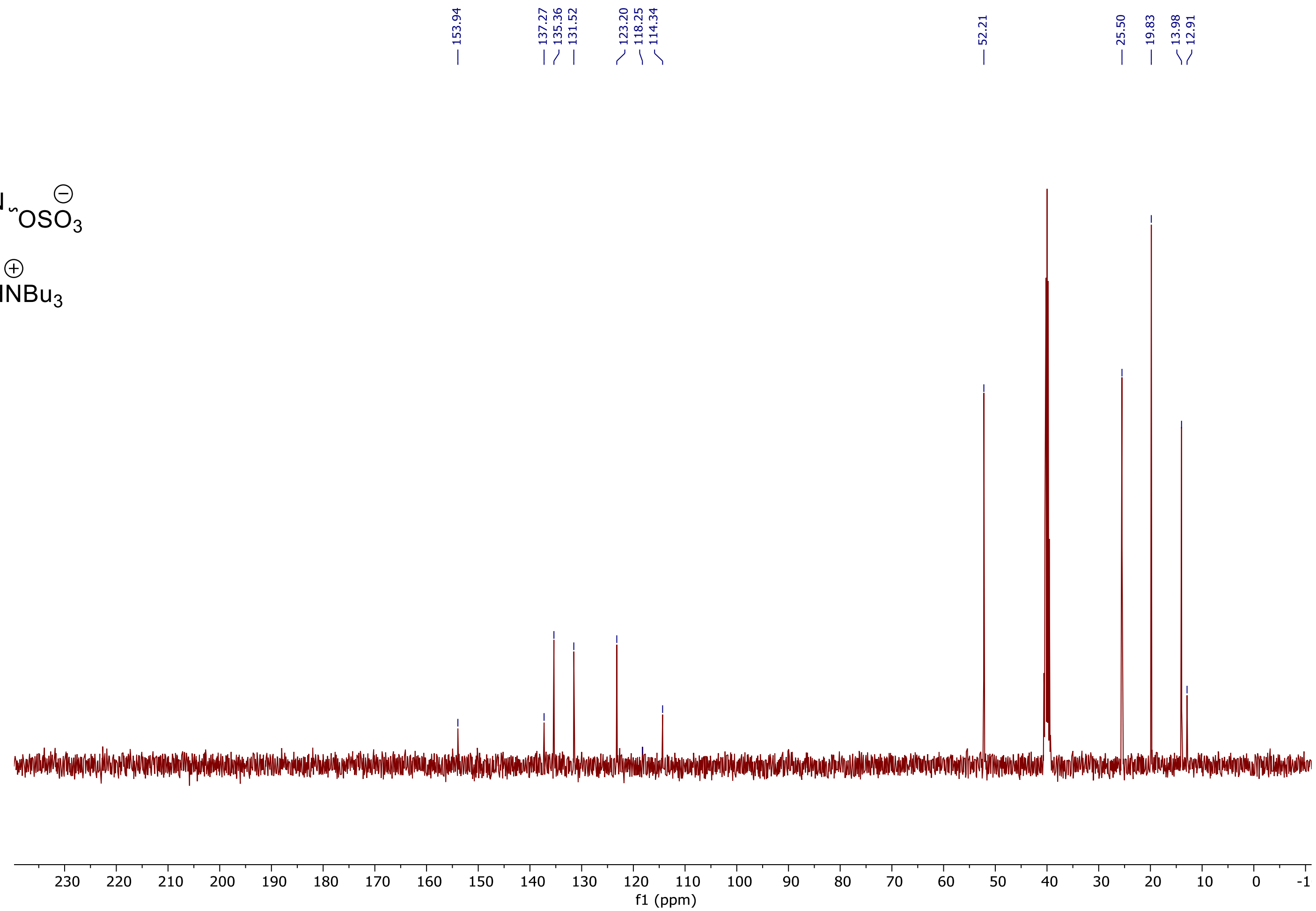

^1H NMR spectrum of **2h** (400 MHz, DMSO-d$_6$)

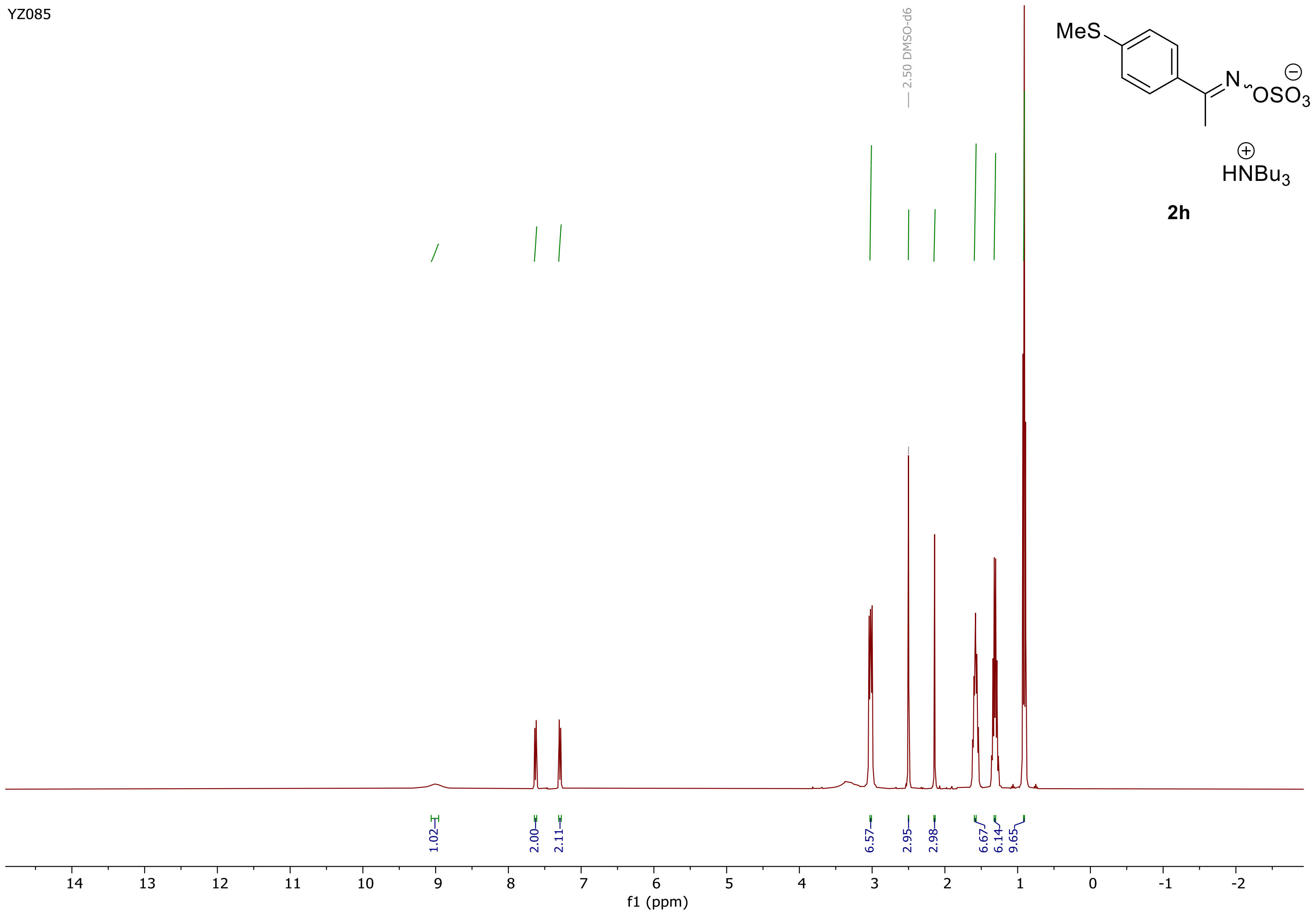

^{13}C NMR spectrum of **2h** (101 MHz, DMSO-d$_6$)

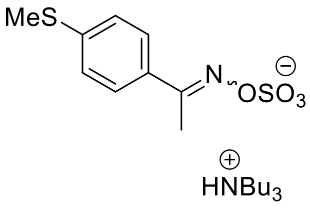

2h

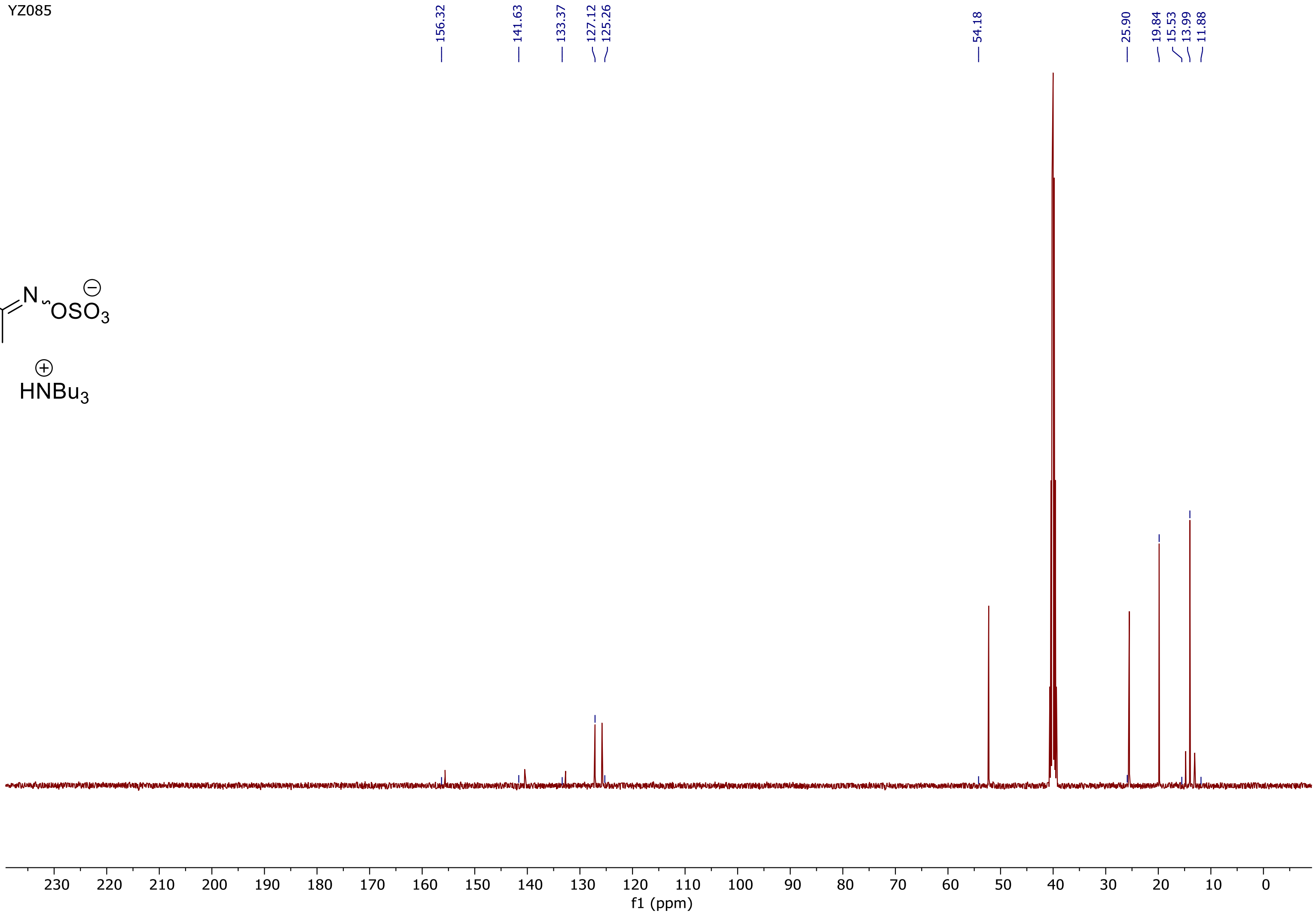

^{1}H NMR spectrum of **2i** (400 MHz, DMSO-d_6)

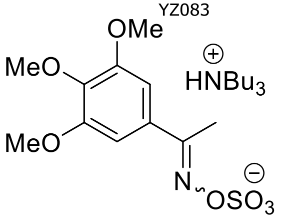

2i

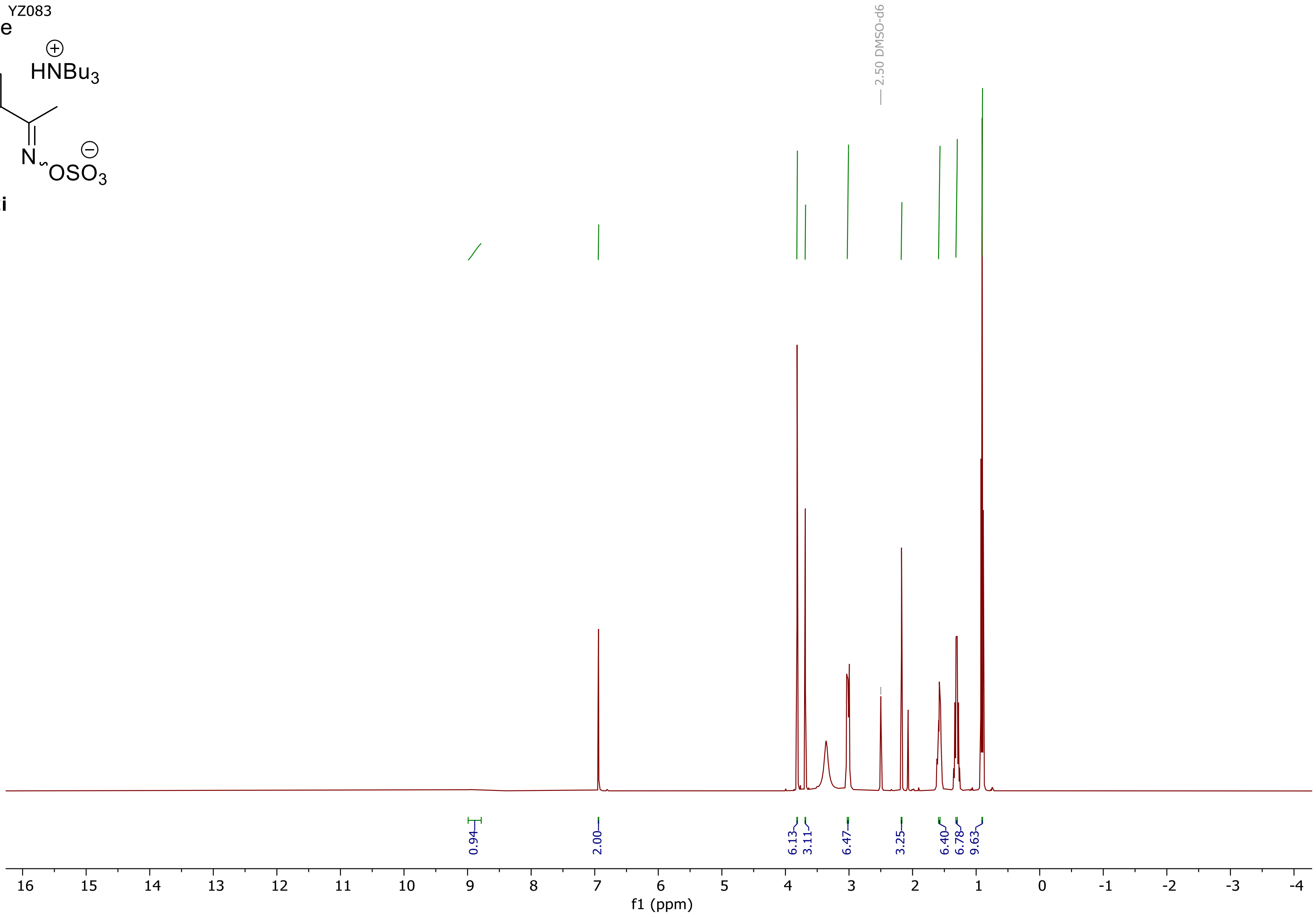

^{13}C NMR spectrum of **2i** (101 MHz, DMSO-d_6)

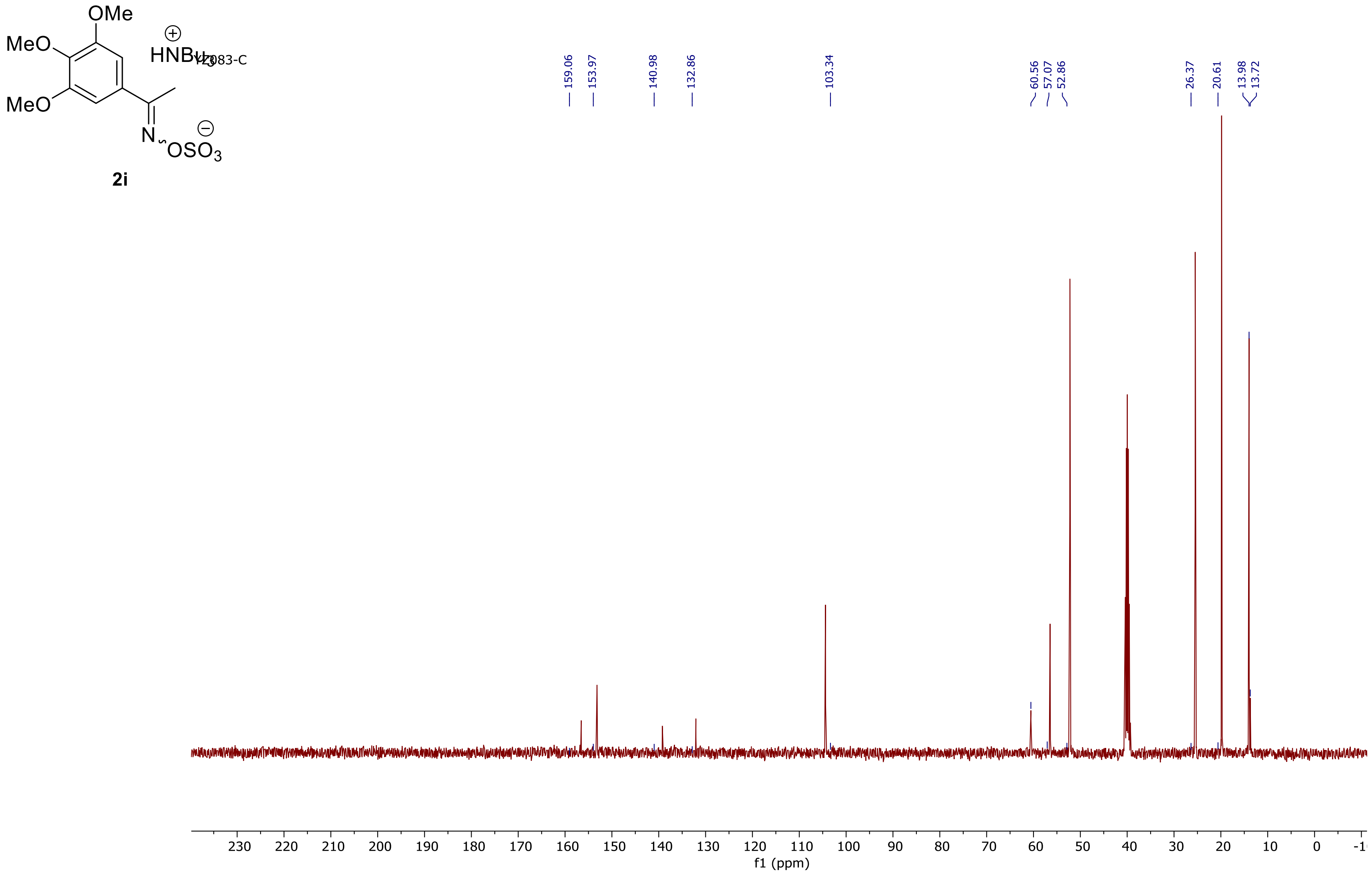

2i

^{1}H NMR spectrum of **2j** (400 MHz, DMSO-d$_6$)

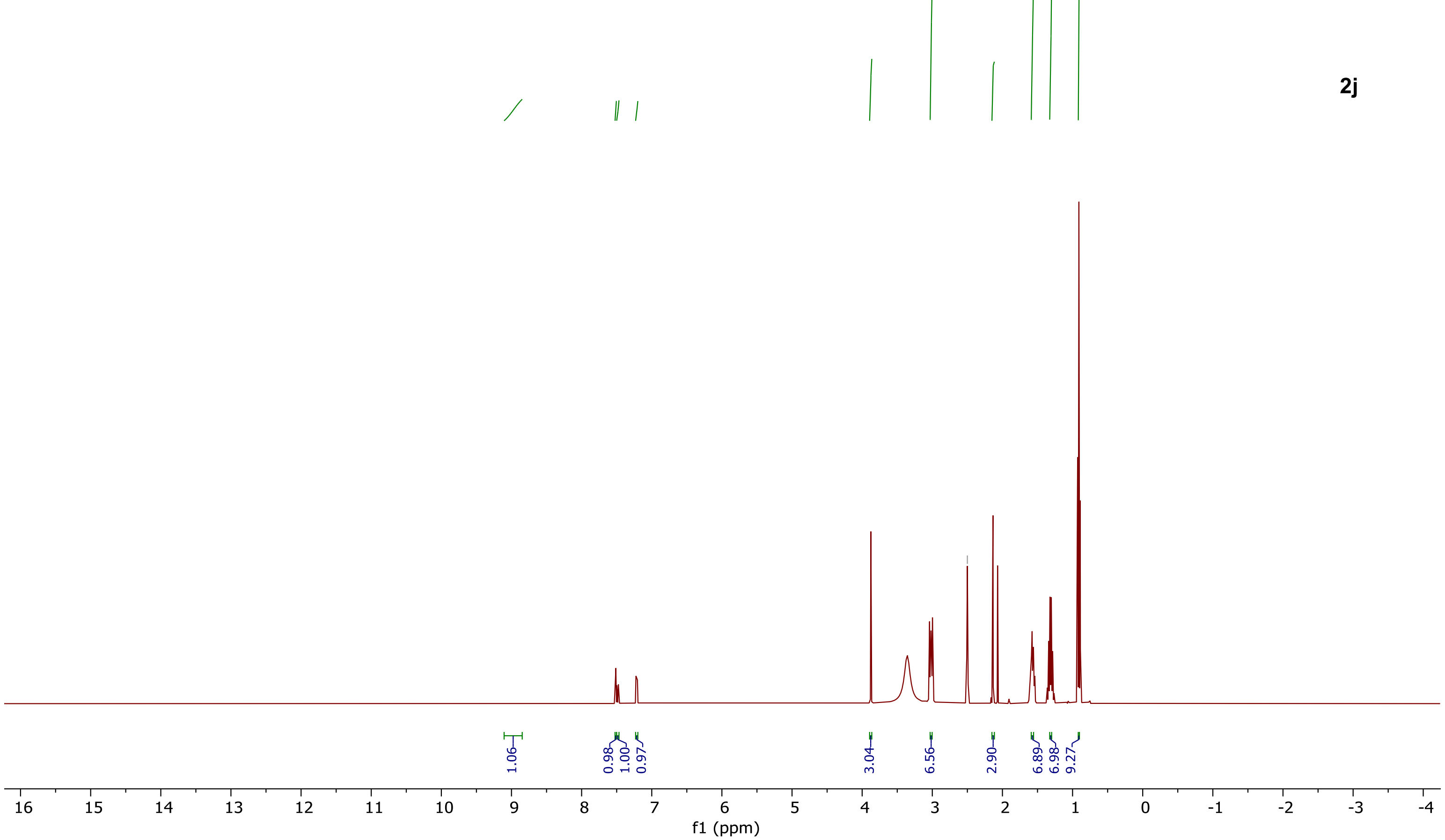

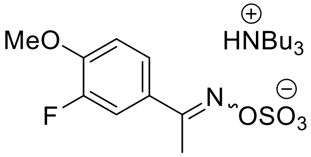

2j

^{13}C NMR spectrum of **2j** (101 MHz, DMSO-d_6)

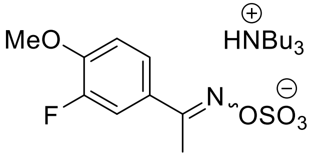

2j

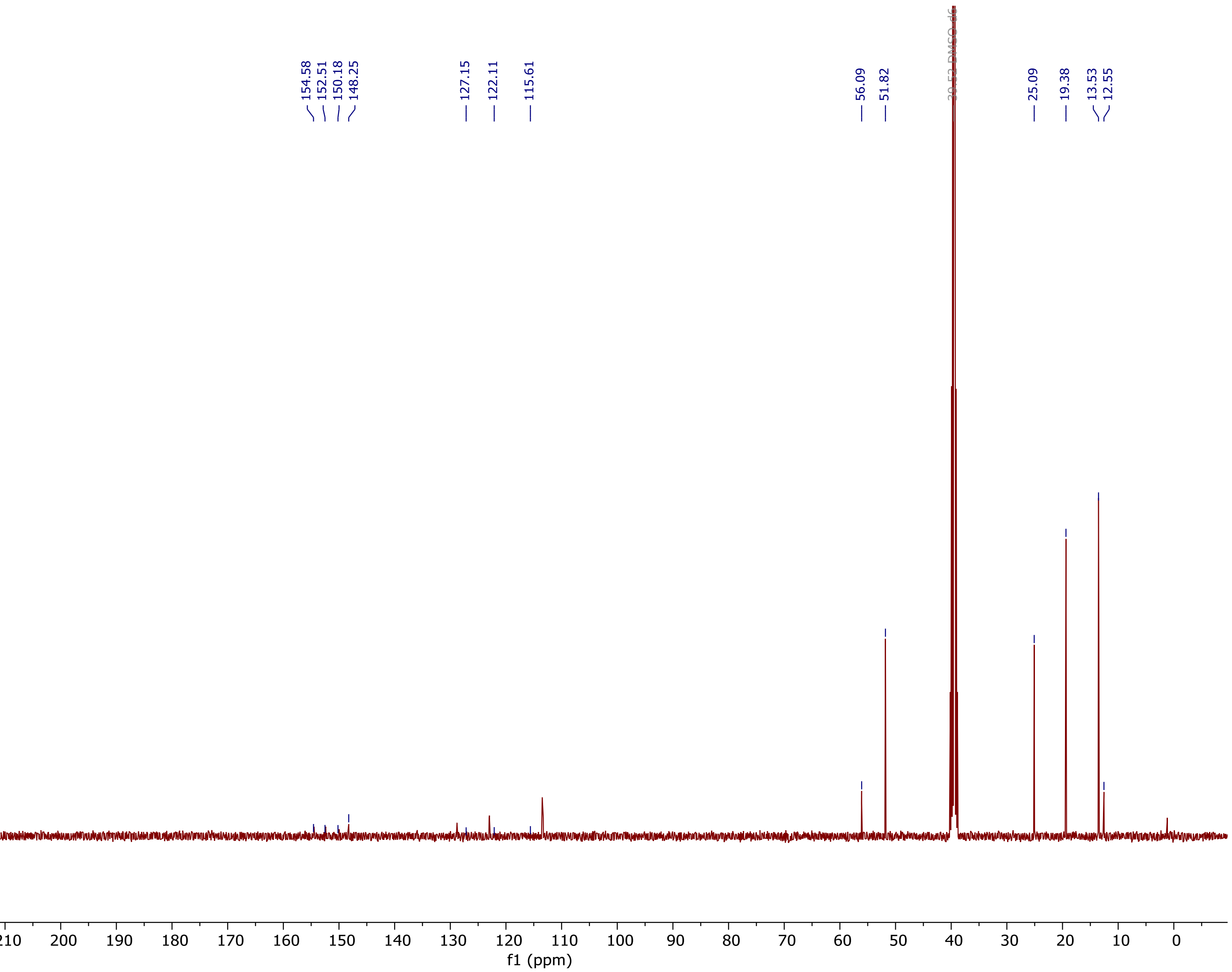

^{19}F NMR spectrum of **2j** (377 MHz, DMSO-d$_6$)

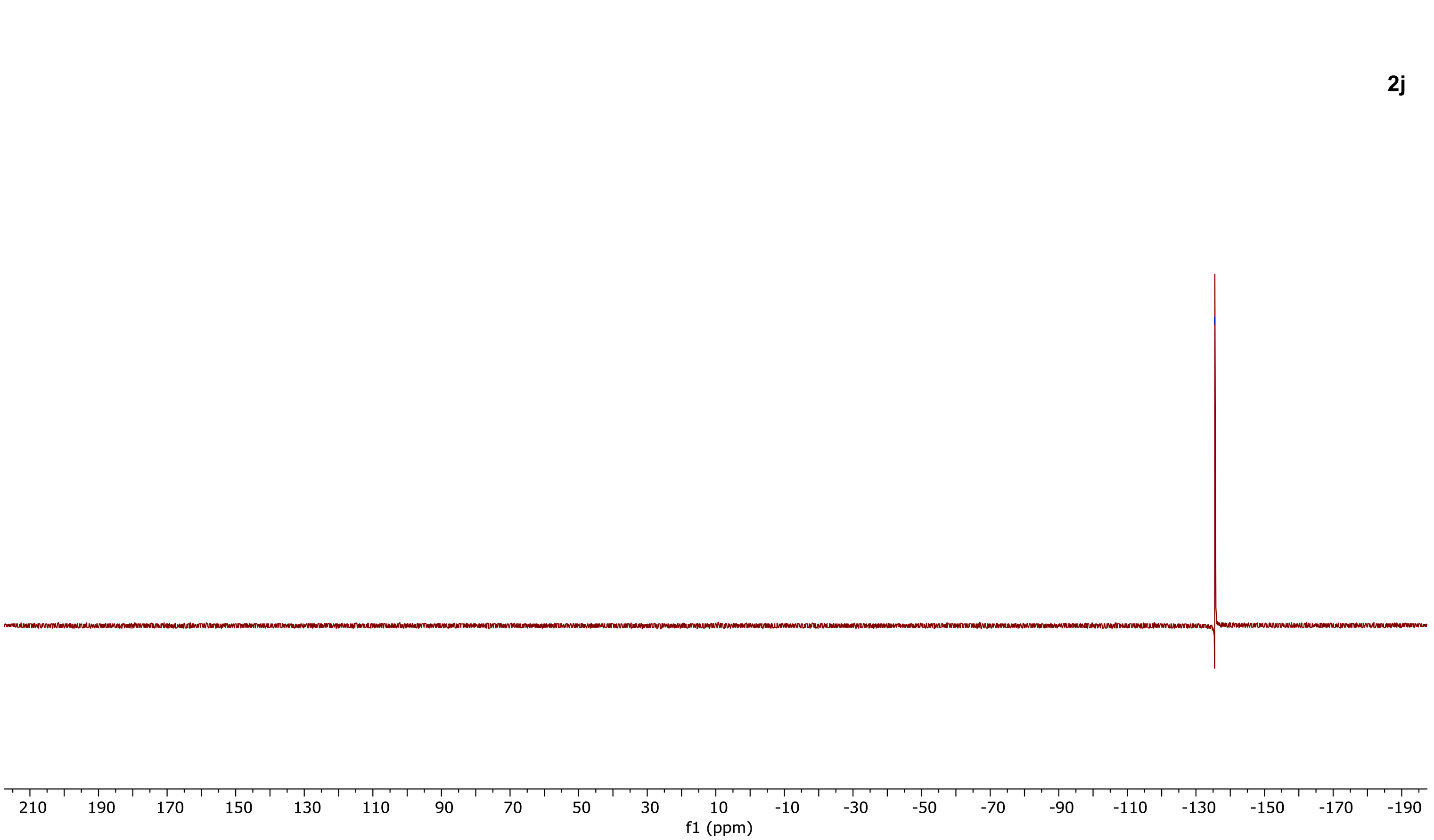

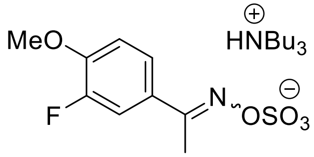

2j

^1H NMR spectrum of **2k** (400 MHz, DMSO-d_6)

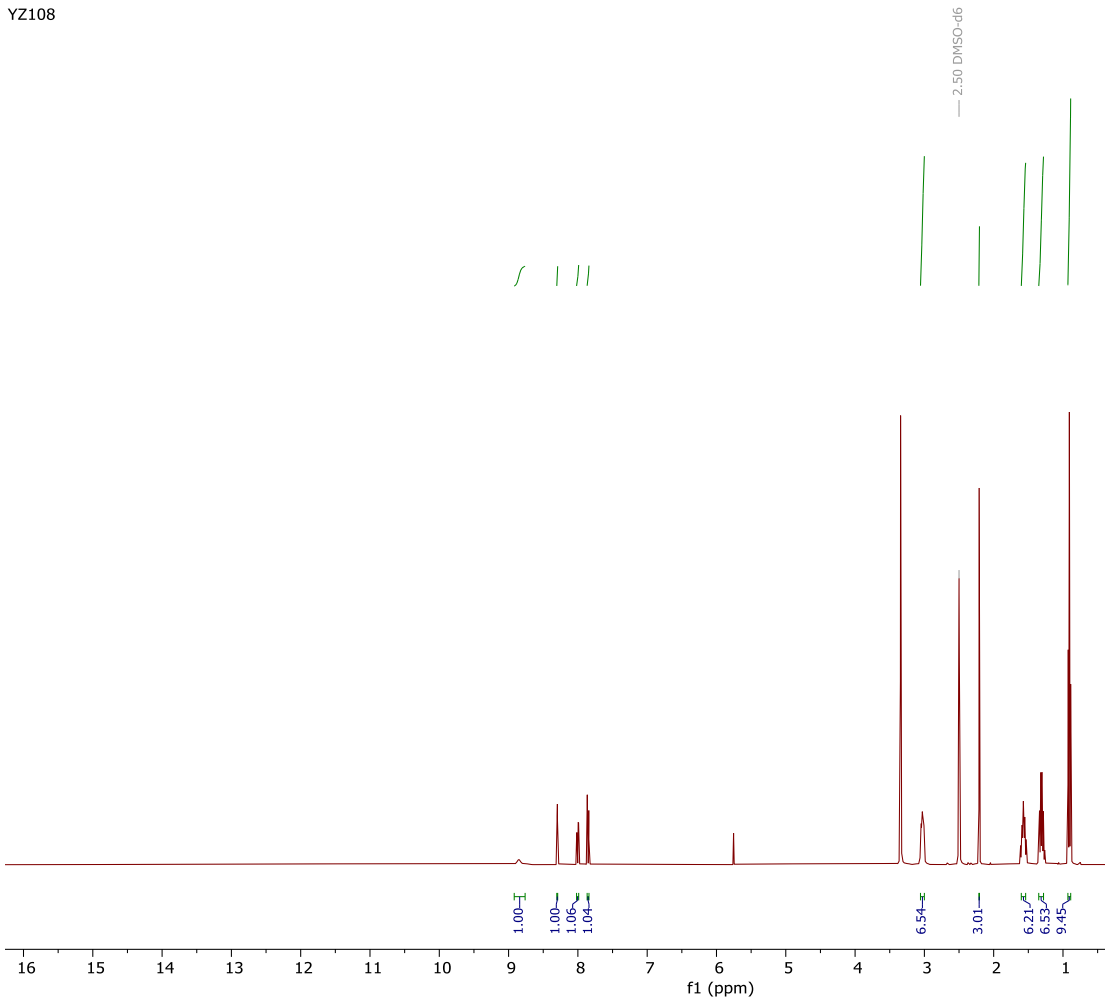

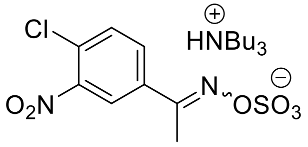

2k

^{13}C NMR spectrum of **2k** (101 MHz, DMSO-d$_6$)

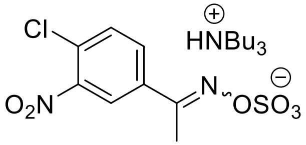

2k

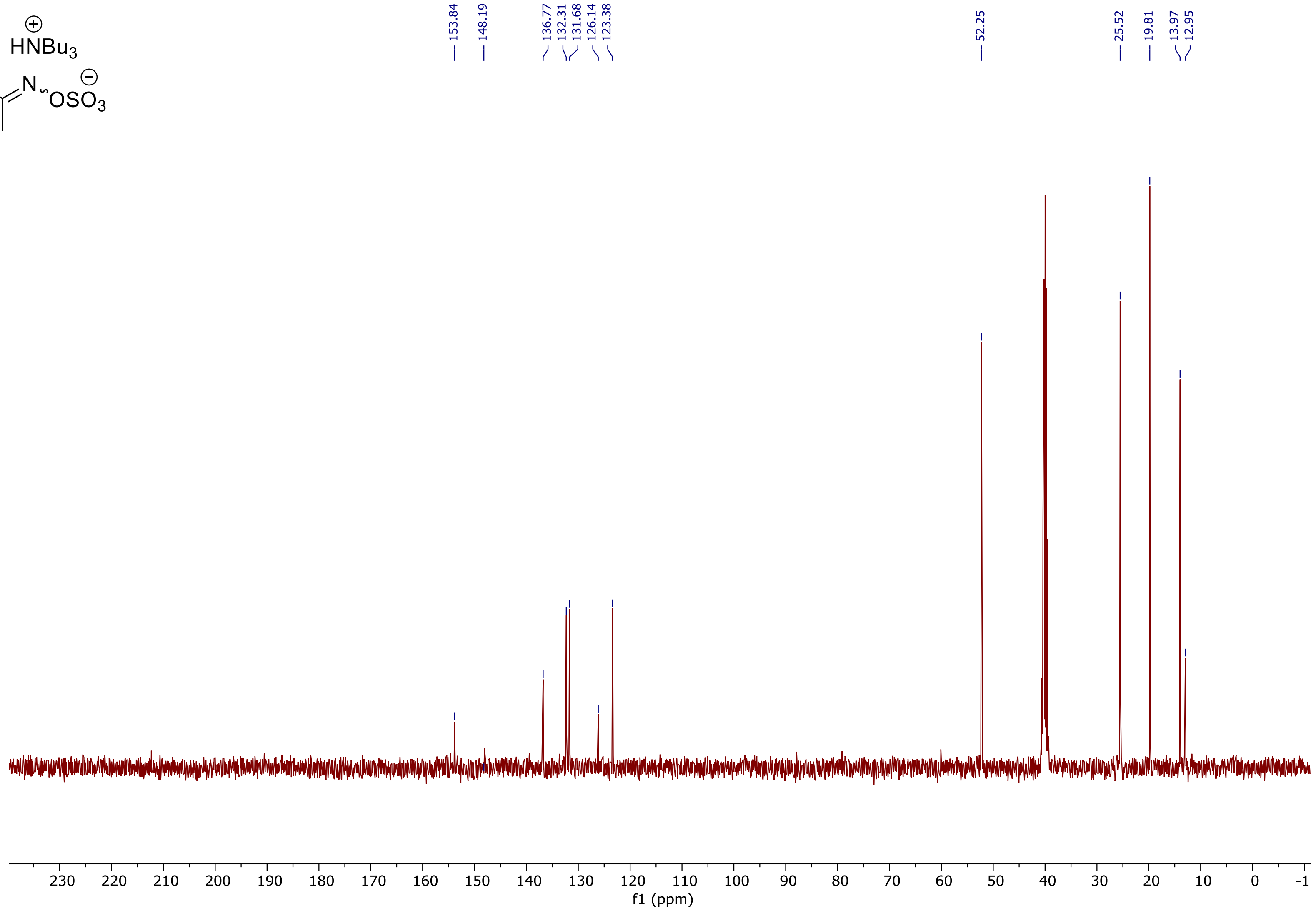

^{1}H NMR spectrum of **2l** (400 MHz, DMSO-d$_6$)

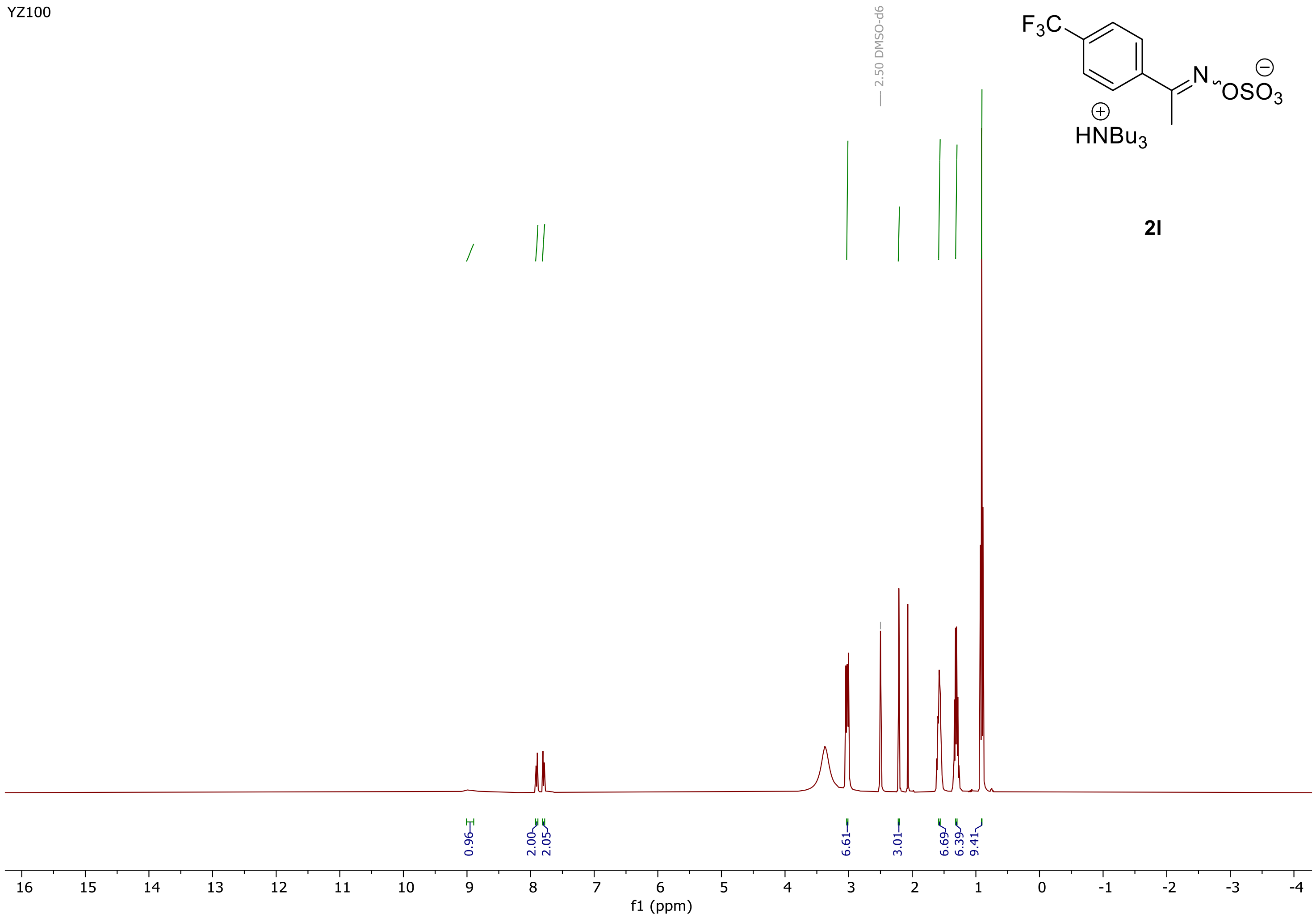

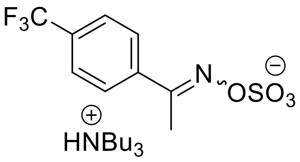
F3C
N
OSO3
HNBu3

2l

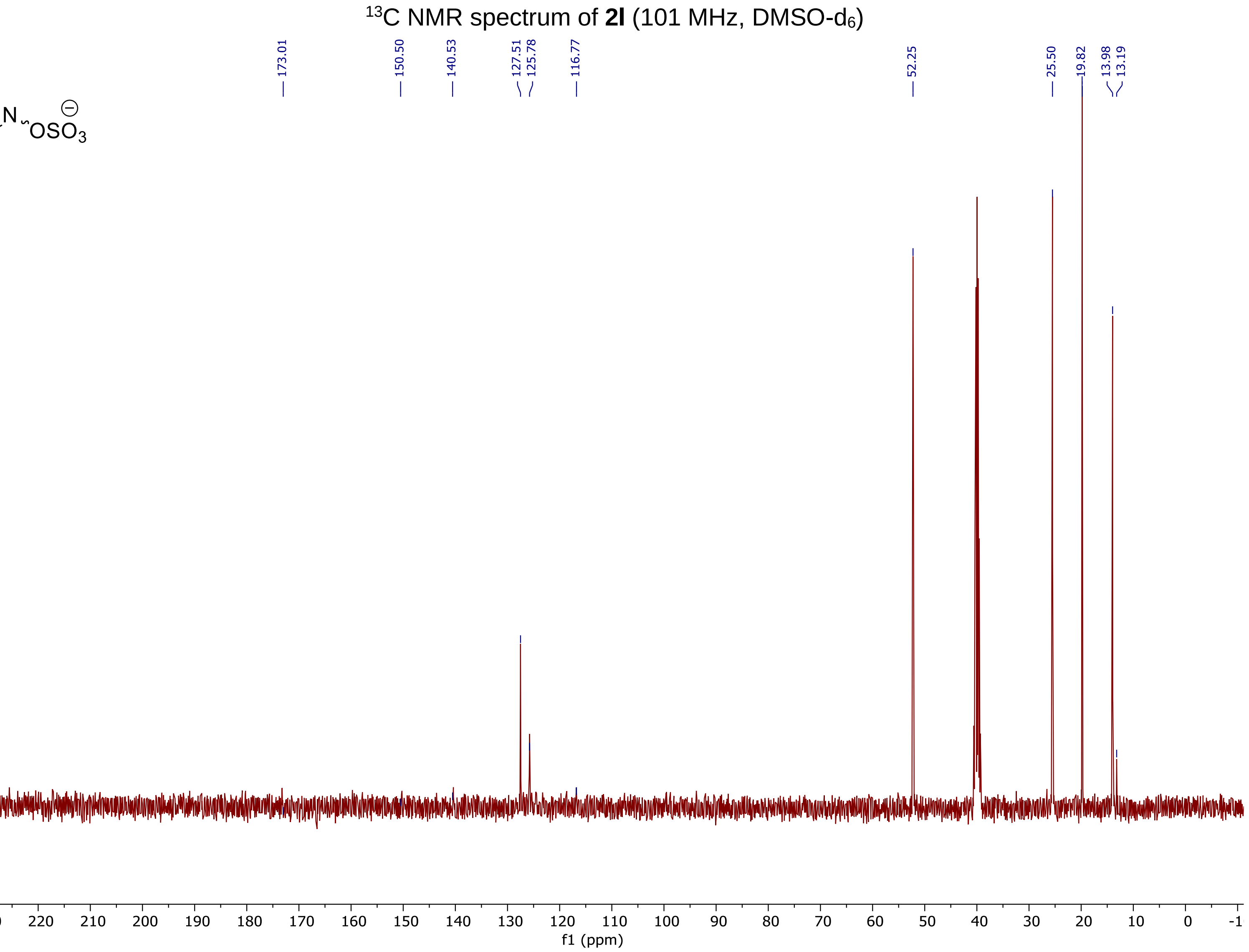
13C NMR spectrum of 2l (101 MHz, DMSO-d6)
173.01
150.50
140.53
127.51
125.78
116.77
52.25
25.50
19.82
13.98
13.19
230
220
210
200
190
180
170
160
150
140
130
120
110
100
90
80
70
60
50
40
30
20
10
0
-1
f1 (ppm)

^{19}F NMR spectrum of **2l** (377 MHz, DMSO-d$_6$)

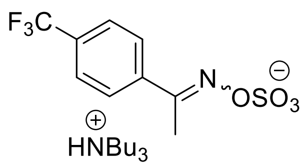

2l

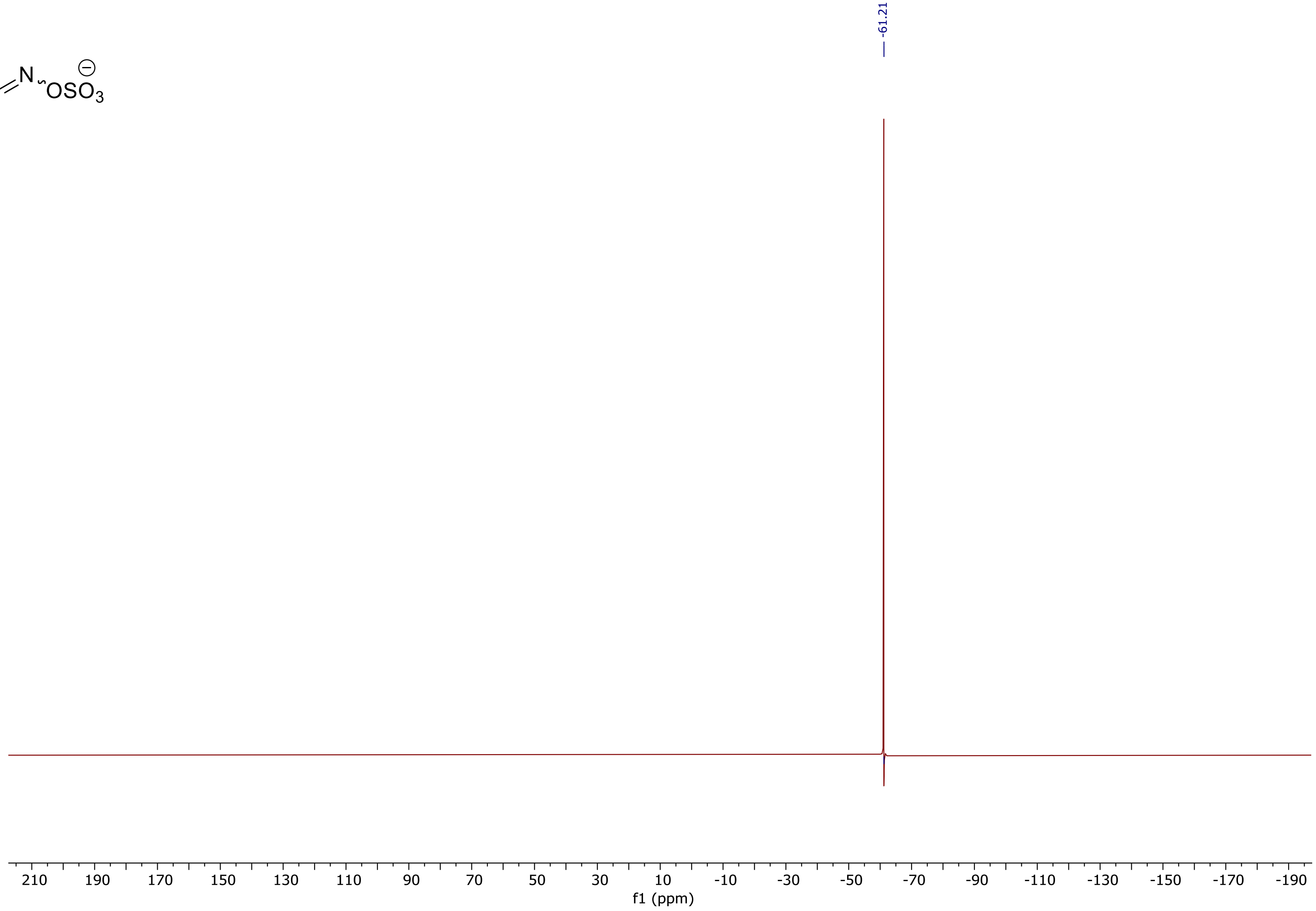

^{1}H NMR spectrum of **2m** (400 MHz, DMSO-d$_6$)

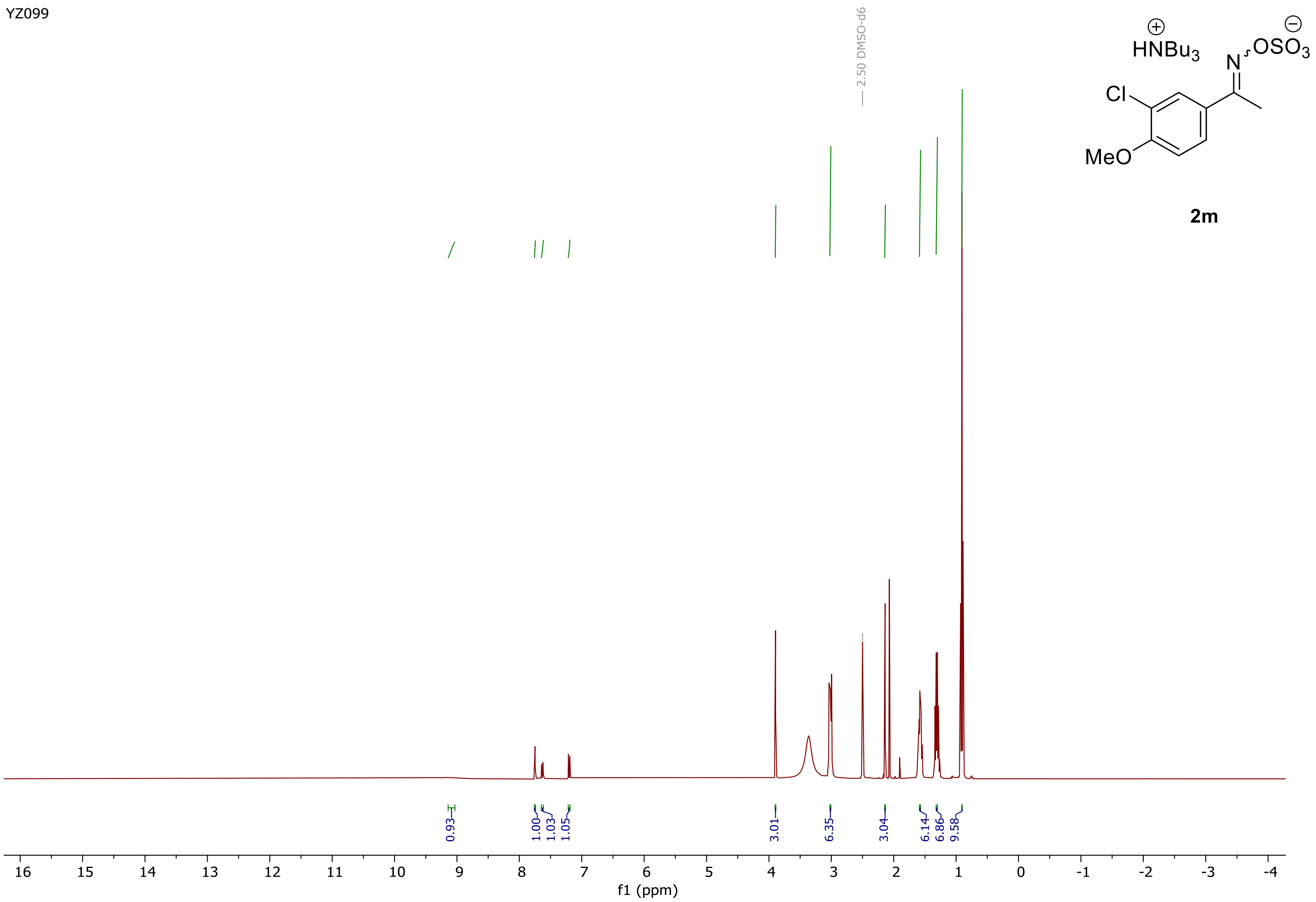

2m

^{13}C NMR spectrum of **2m** (101 MHz, DMSO-d$_6$)

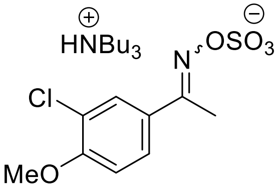

2m

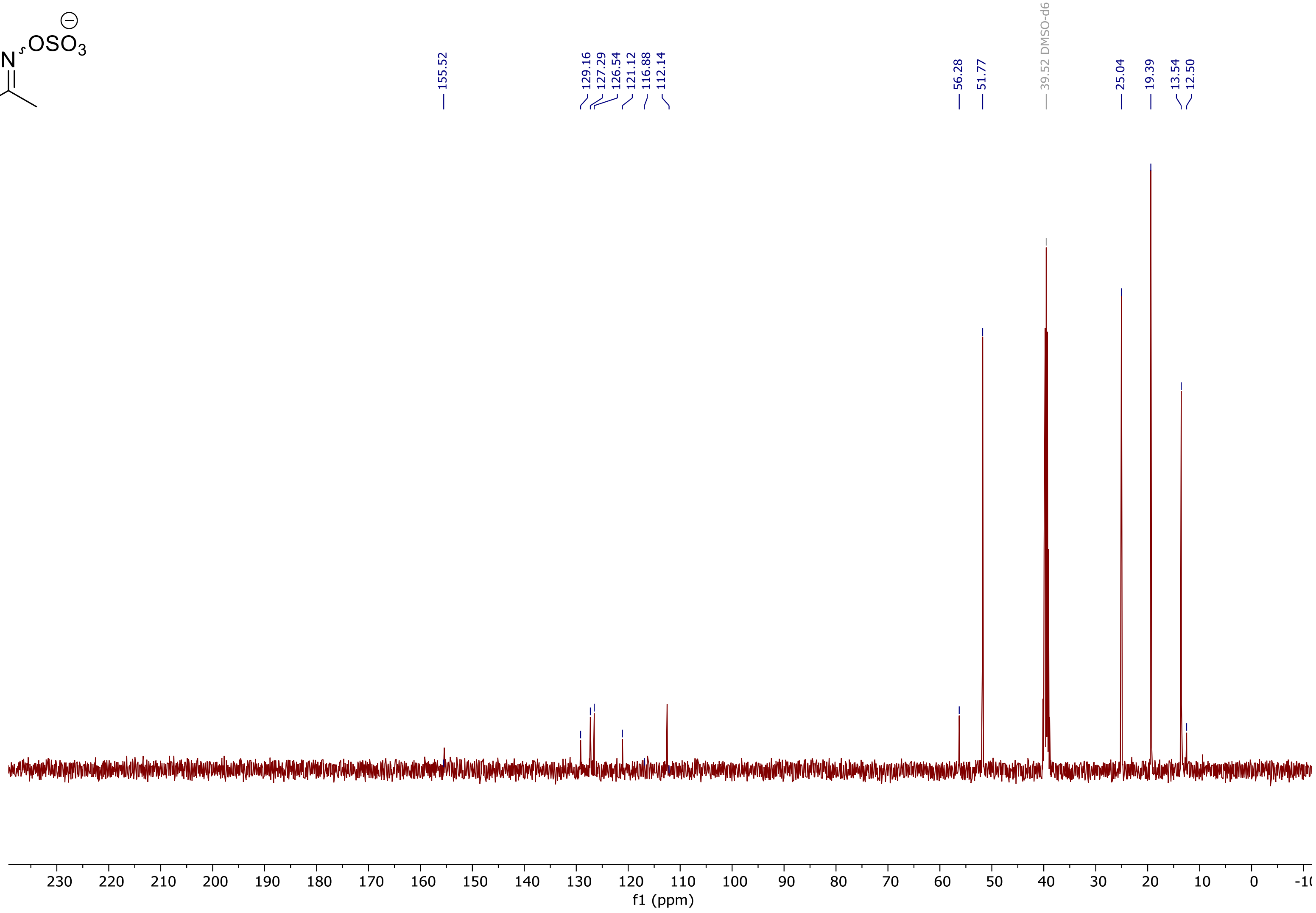

^{1}H NMR spectrum of **2n** (400 MHz, DMSO-d6)

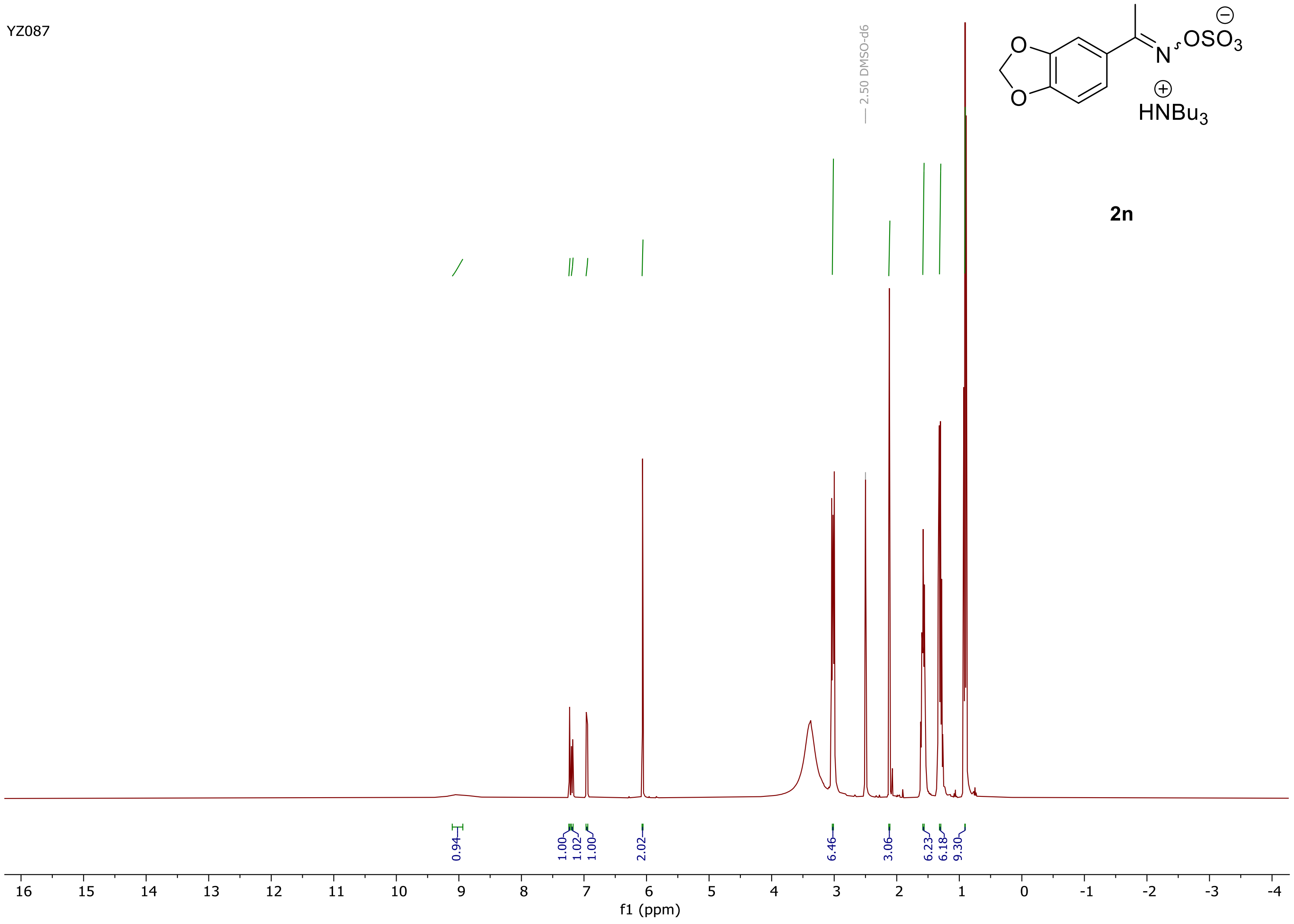

2n

^{13}C NMR spectrum of **2n** (101 MHz, DMSO-d$_6$)

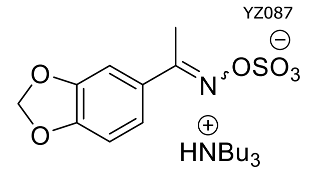

2n

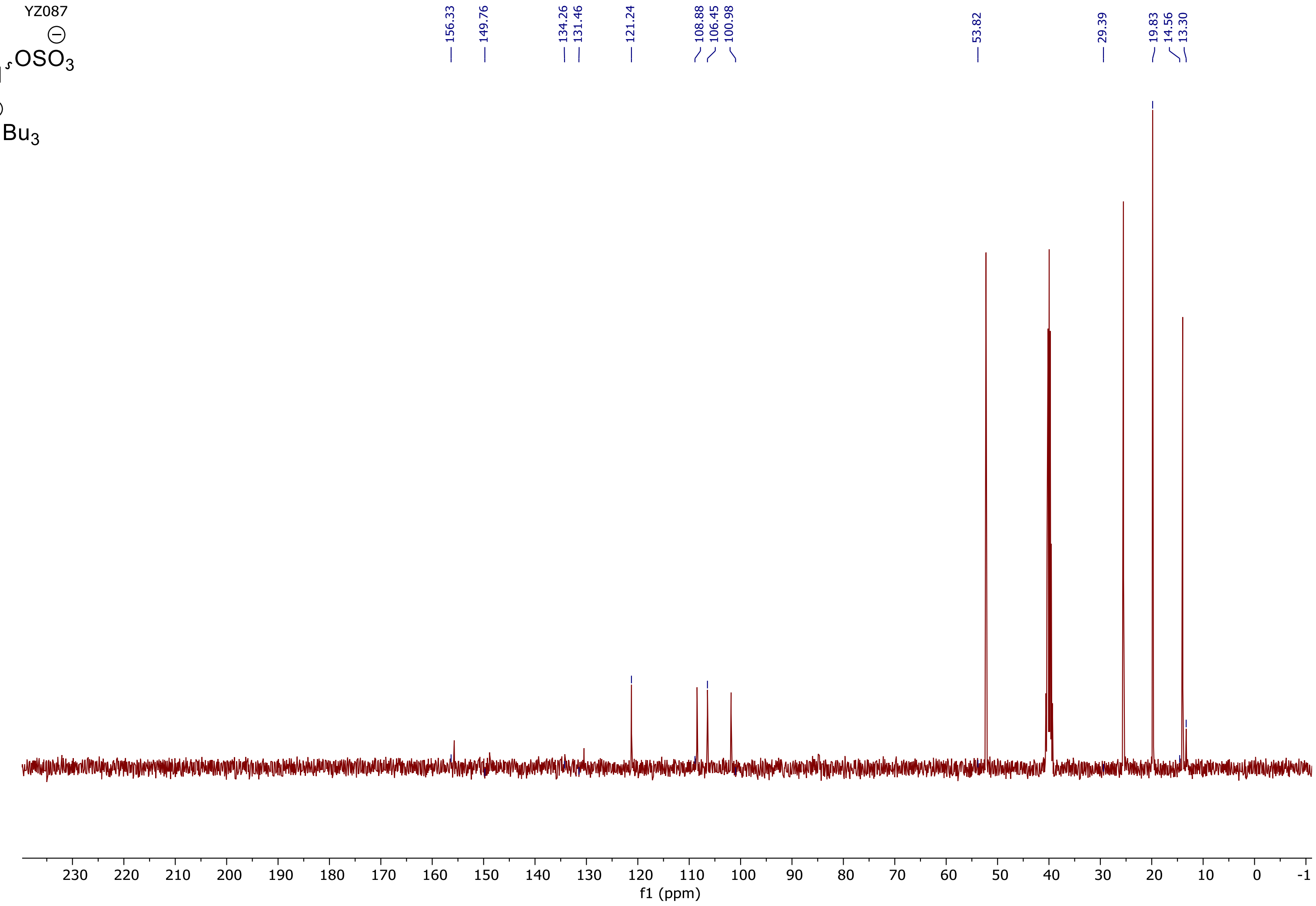

^{1}H NMR spectrum of **2o** (400 MHz, DMSO-d$_6$)

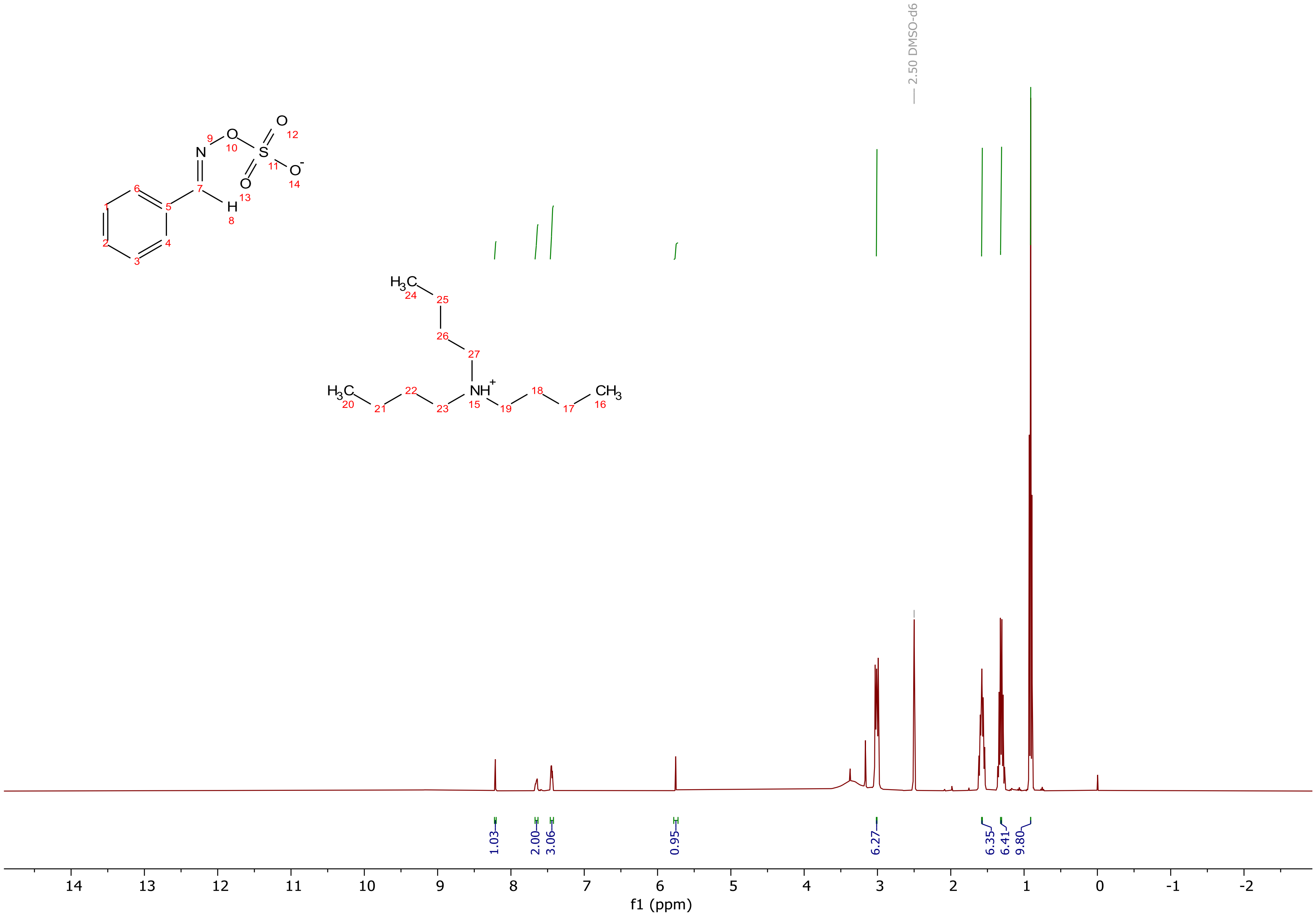

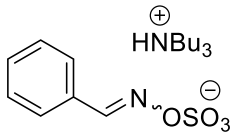

2o

^{13}C NMR spectrum of **2o** (101 MHz, DMSO-d$_6$)

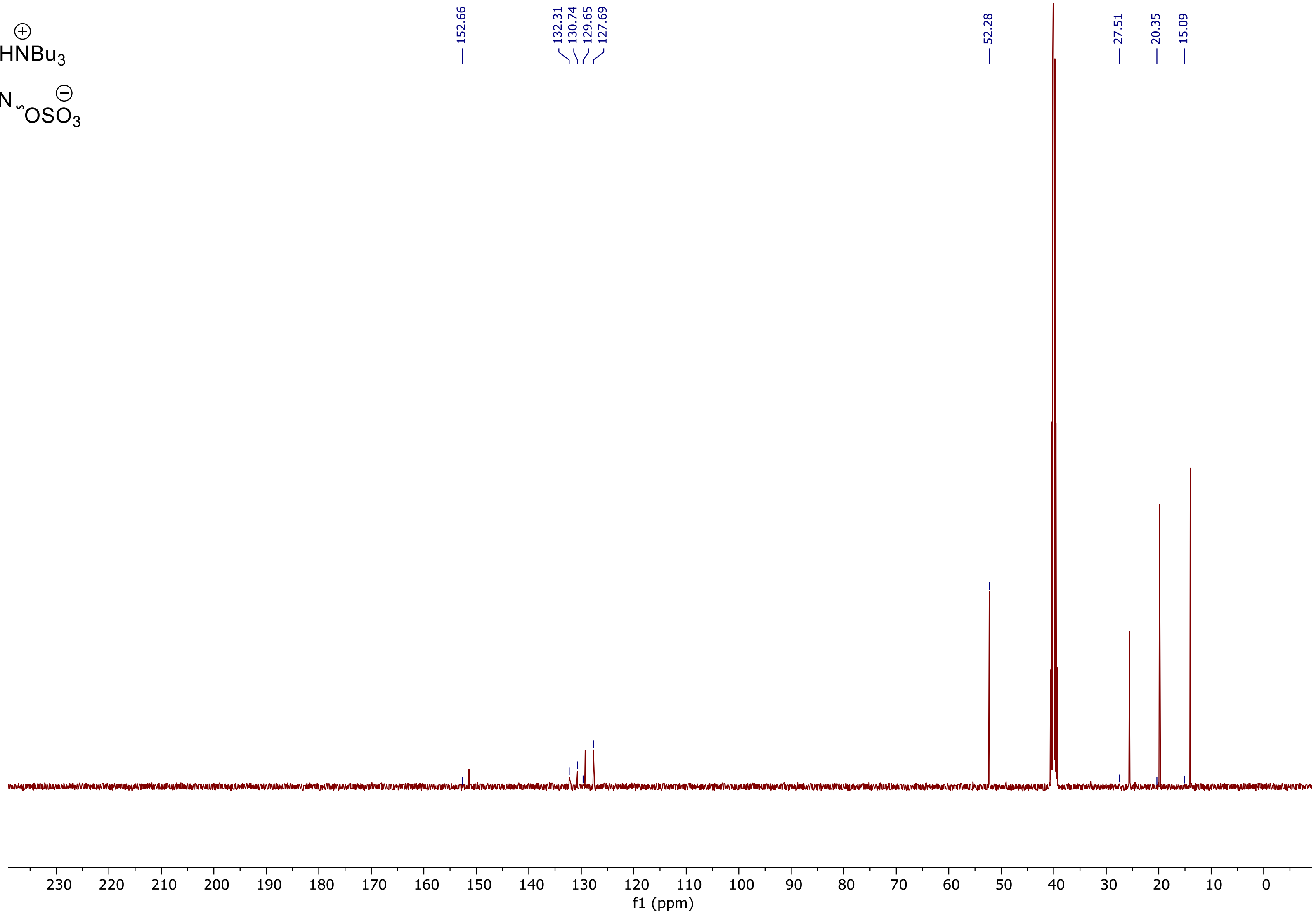

^{1}H NMR spectrum of **2p** (400 MHz, DMSO-d$_6$)

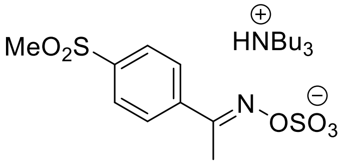

2p

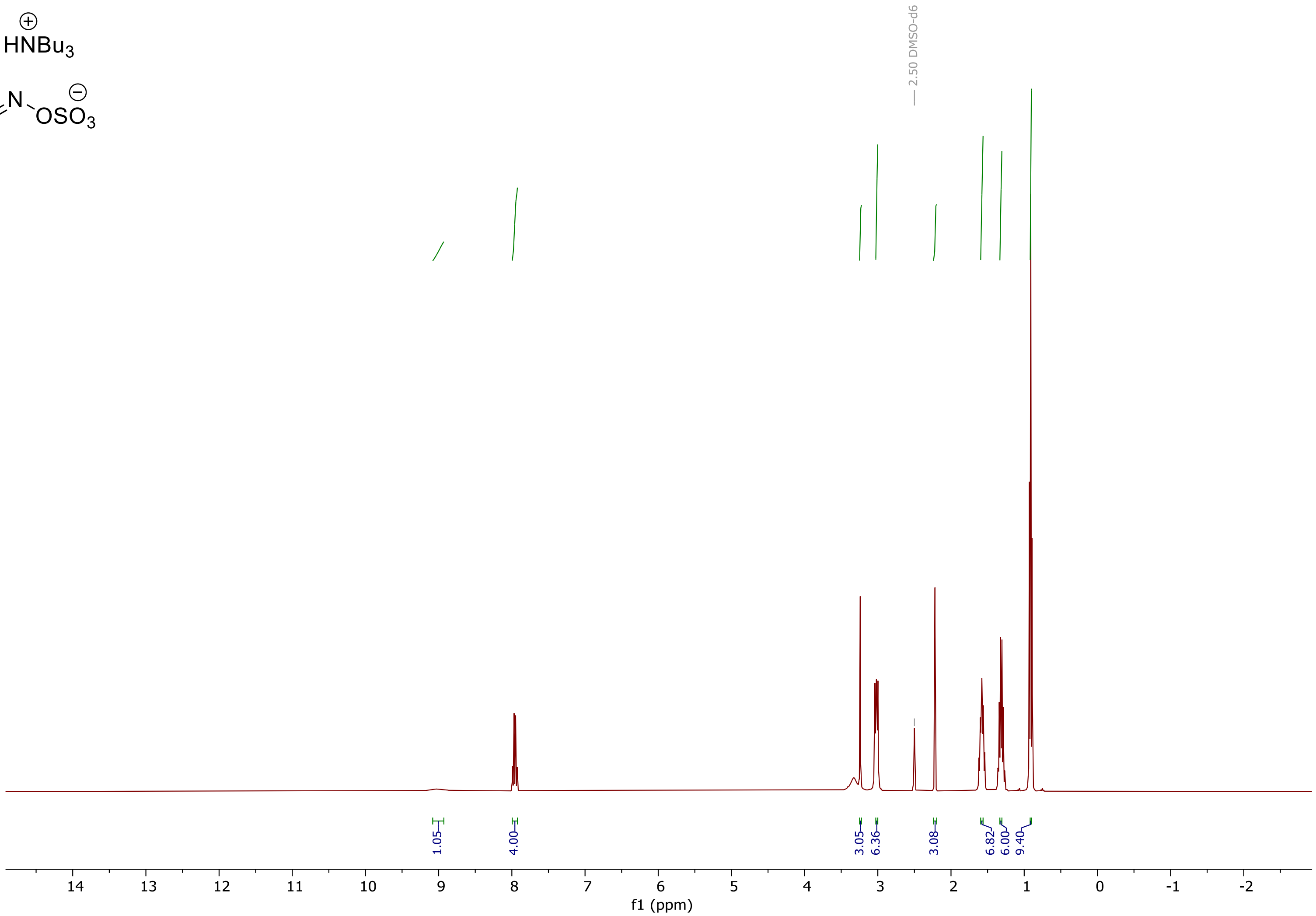

^{13}C NMR spectrum of **2p** (101 MHz, DMSO-d$_6$)

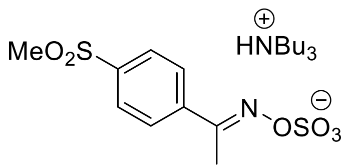

2p

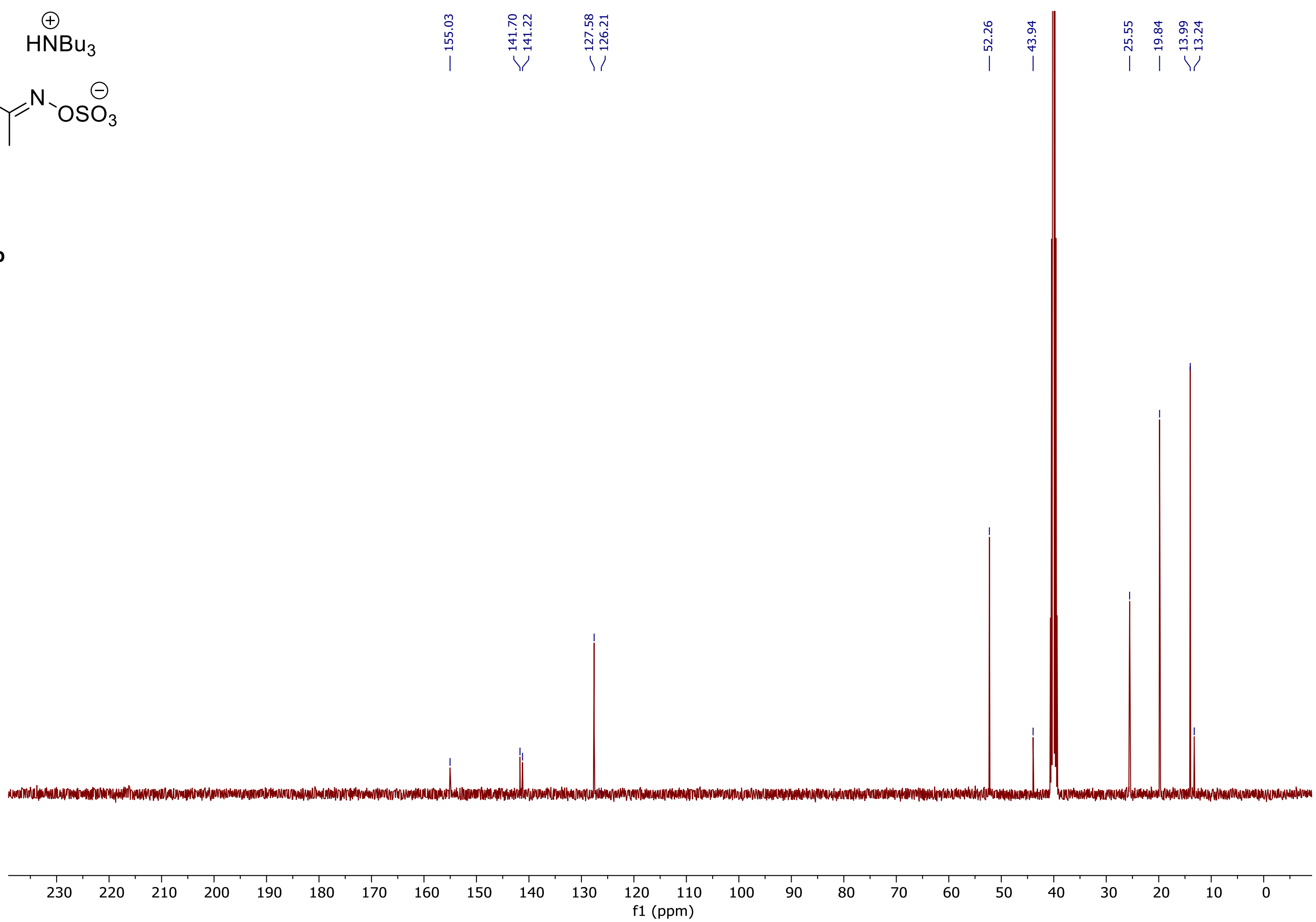

^{1}H NMR spectrum of **2q** (400 MHz, DMSO-d_6)

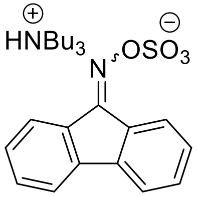

2q

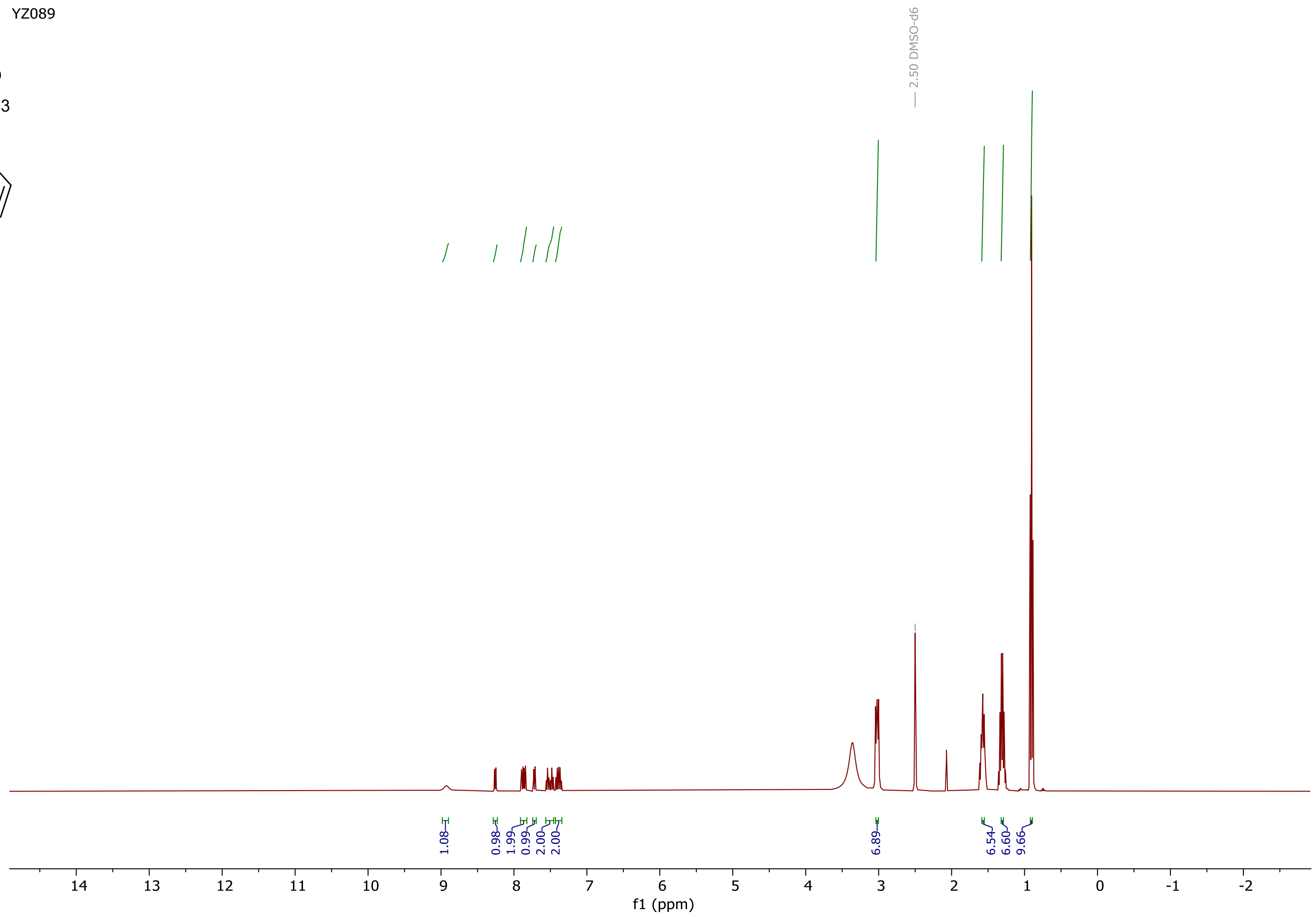

^{13}C NMR spectrum of **2q** (101 MHz, DMSO-d_6)

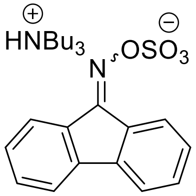

2q

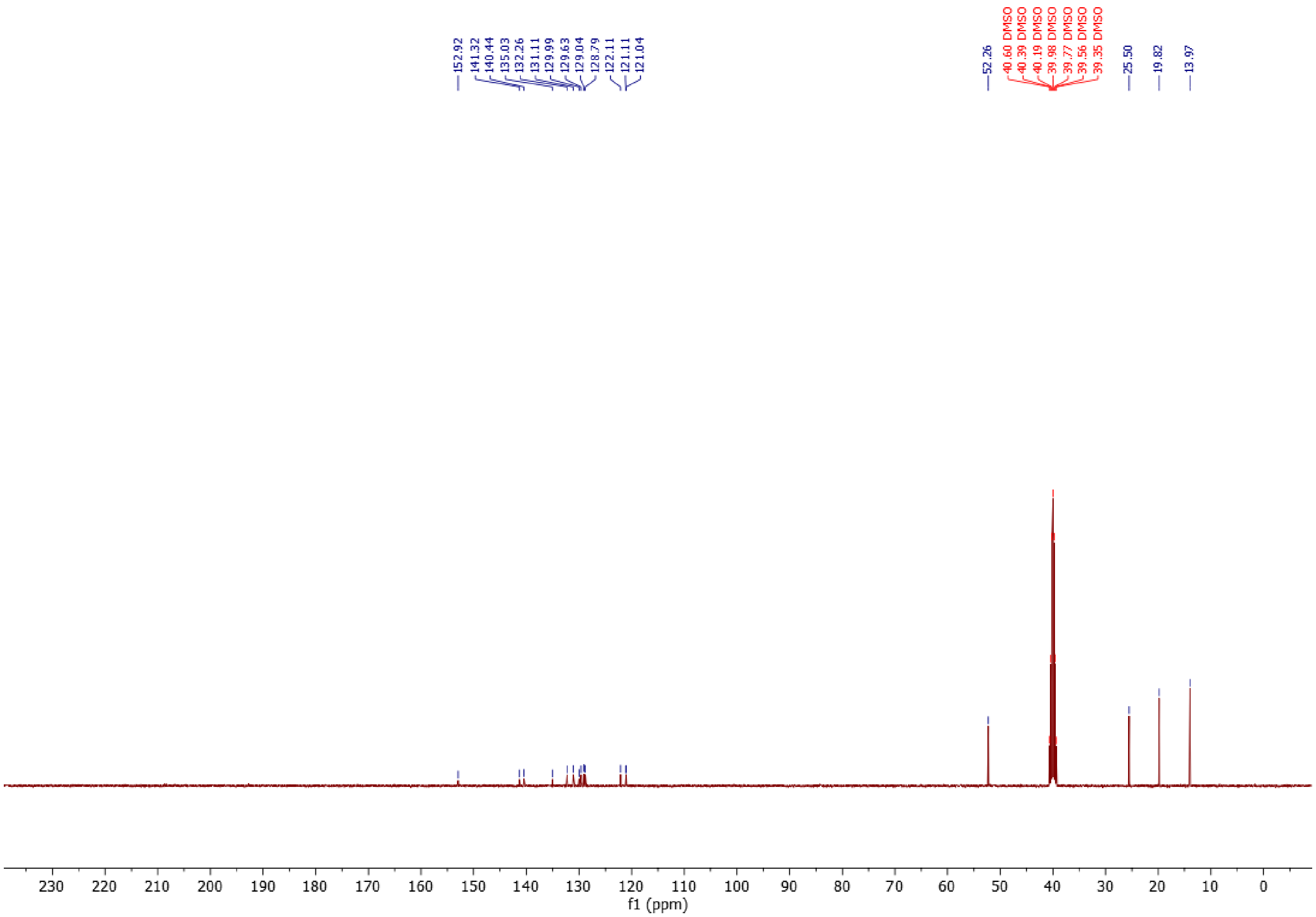

^{1}H NMR spectrum of **2r** (400 MHz, DMSO-d$_6$)

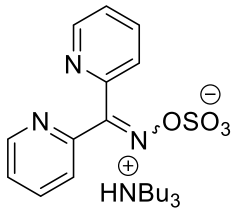

2r

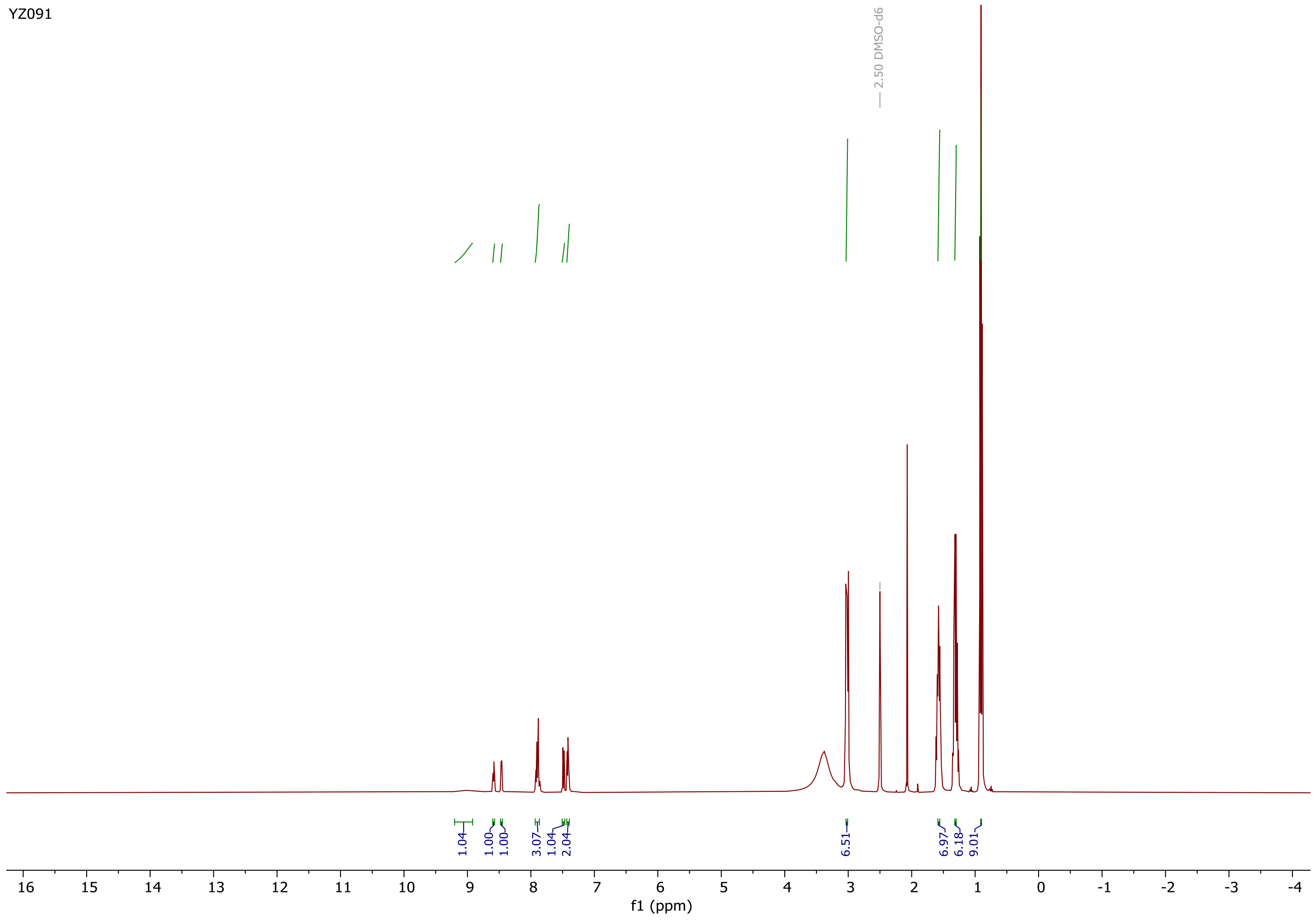

^{13}C NMR spectrum of **2r** (101 MHz, DMSO-d$_6$)

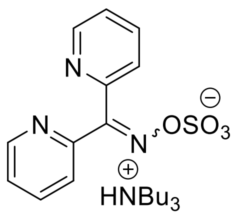

2r

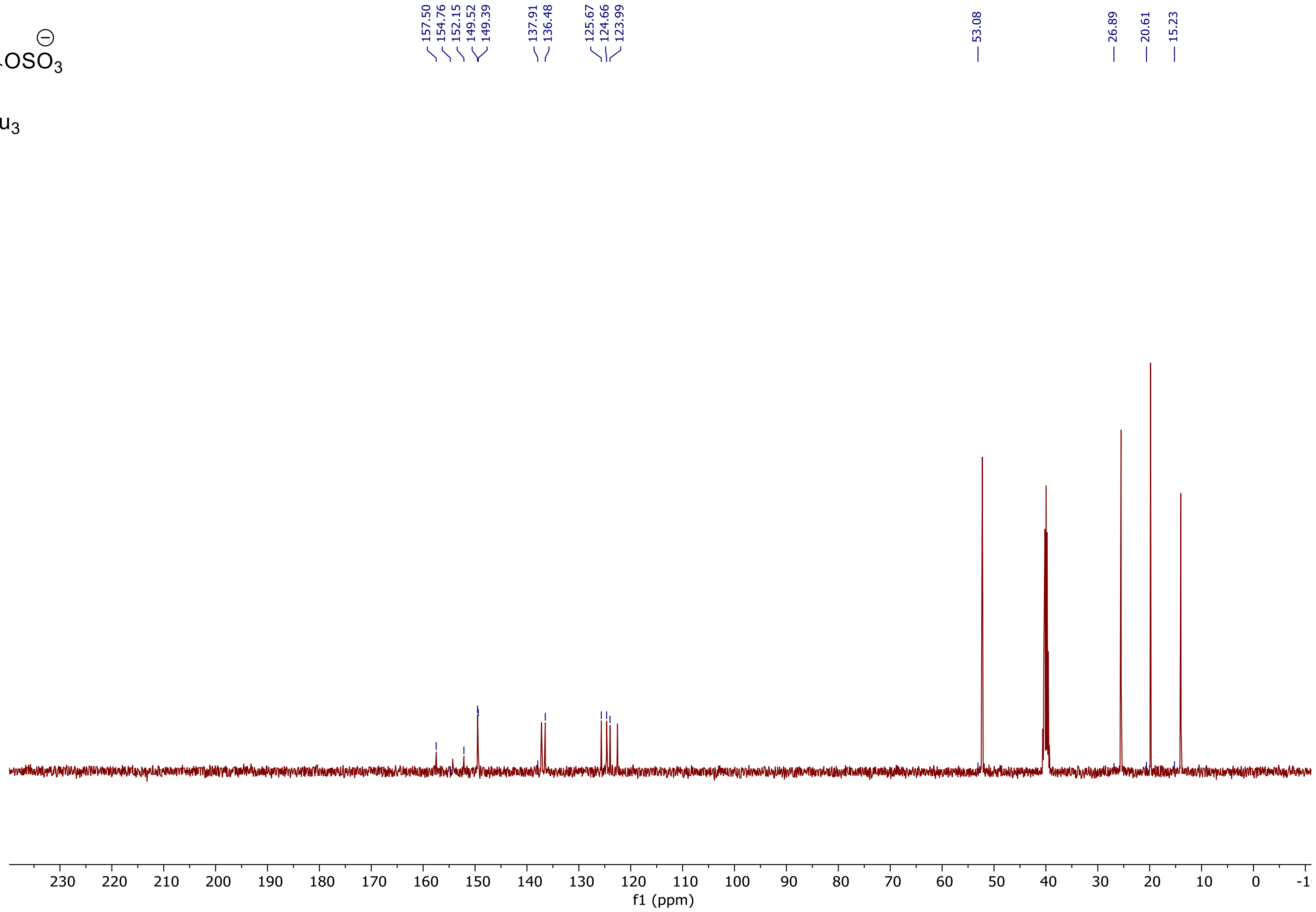

^{1}H NMR spectrum of **2s** (400 MHz, DMSO-d$_6$)

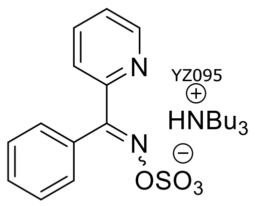

2s

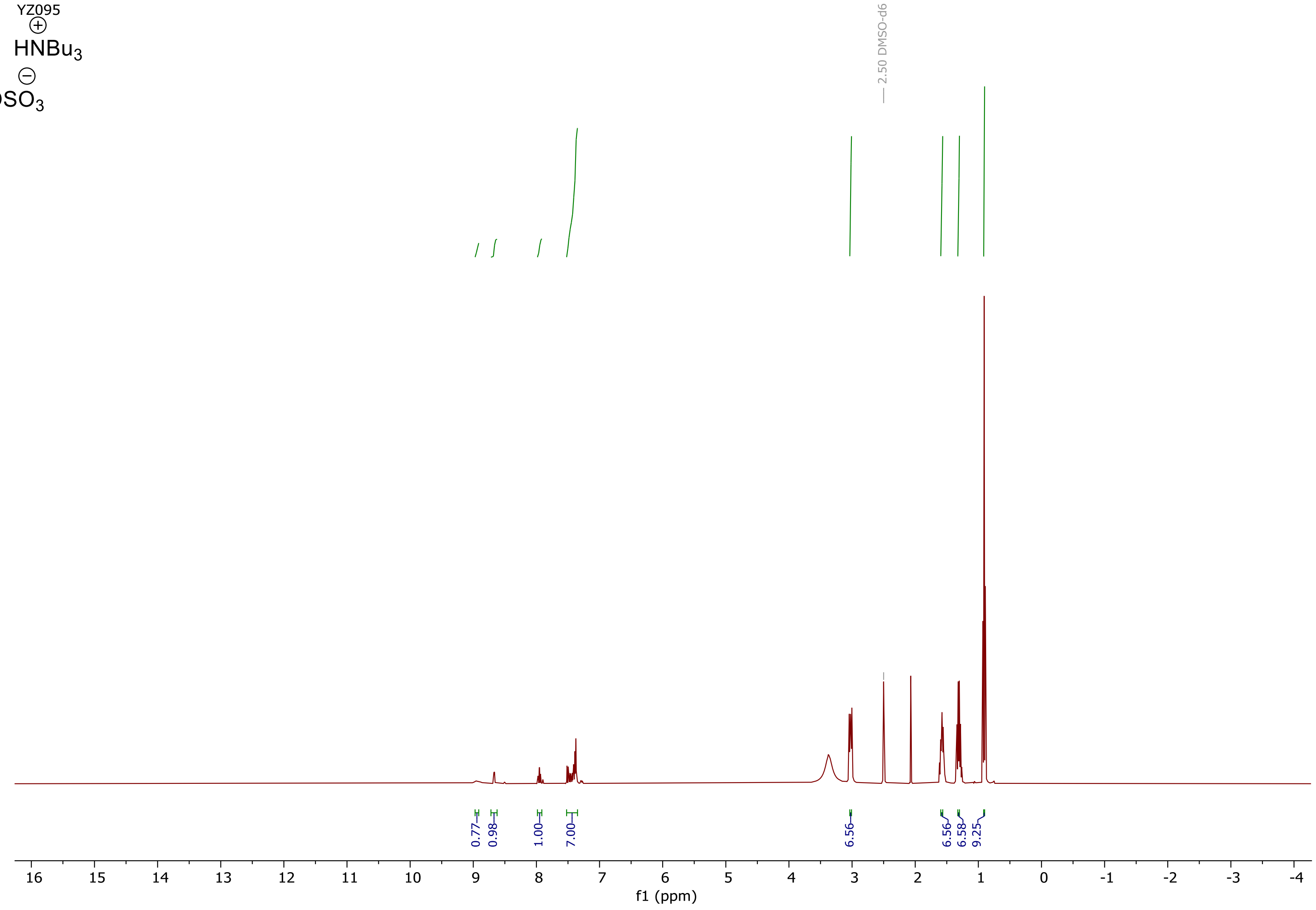

^{13}C NMR spectrum of **2s** (101 MHz, DMSO-d$_6$)

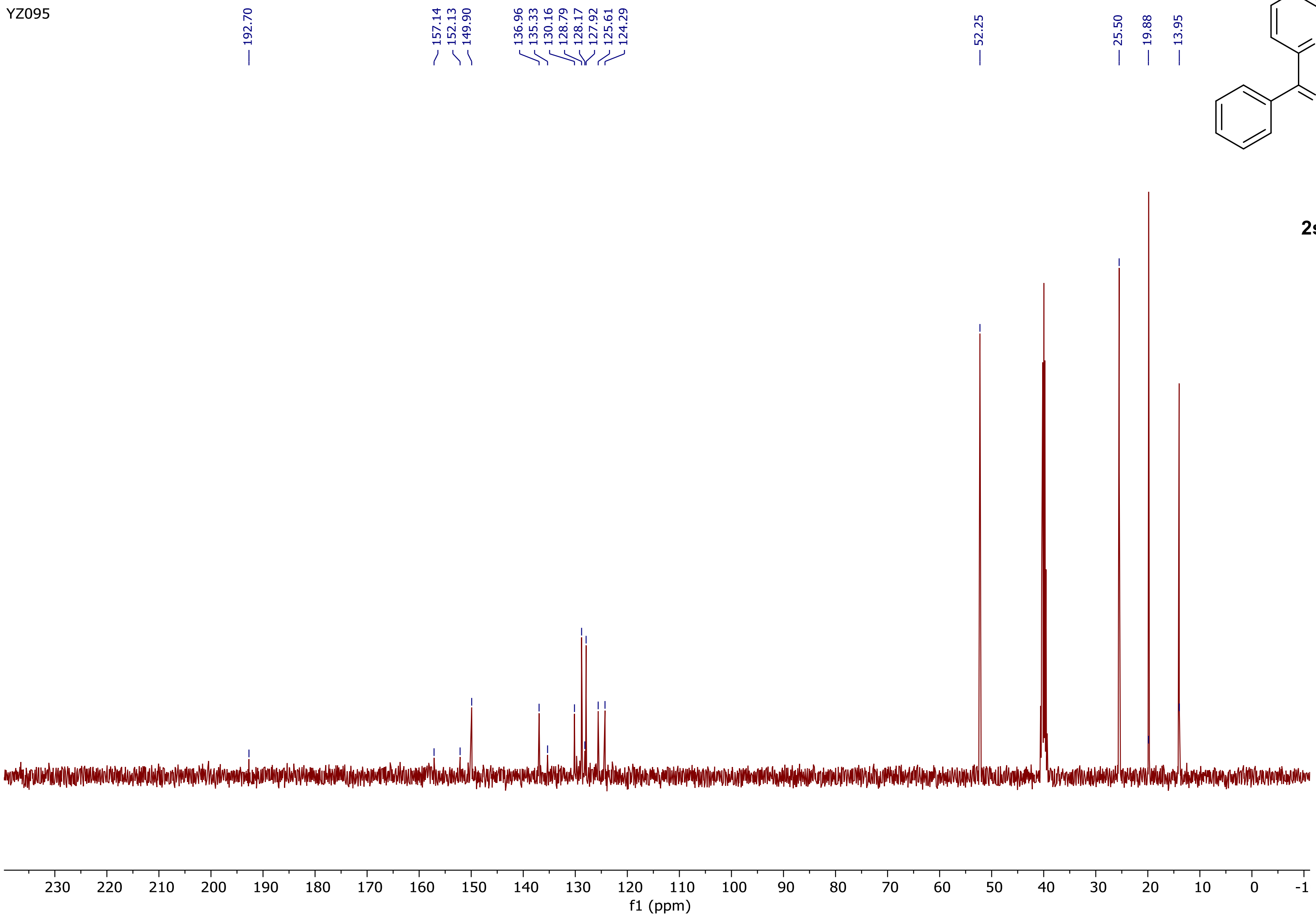

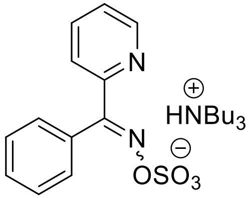

2s

^{1}H NMR spectrum of **3a** (400 MHz, DMSO-d$_6$)

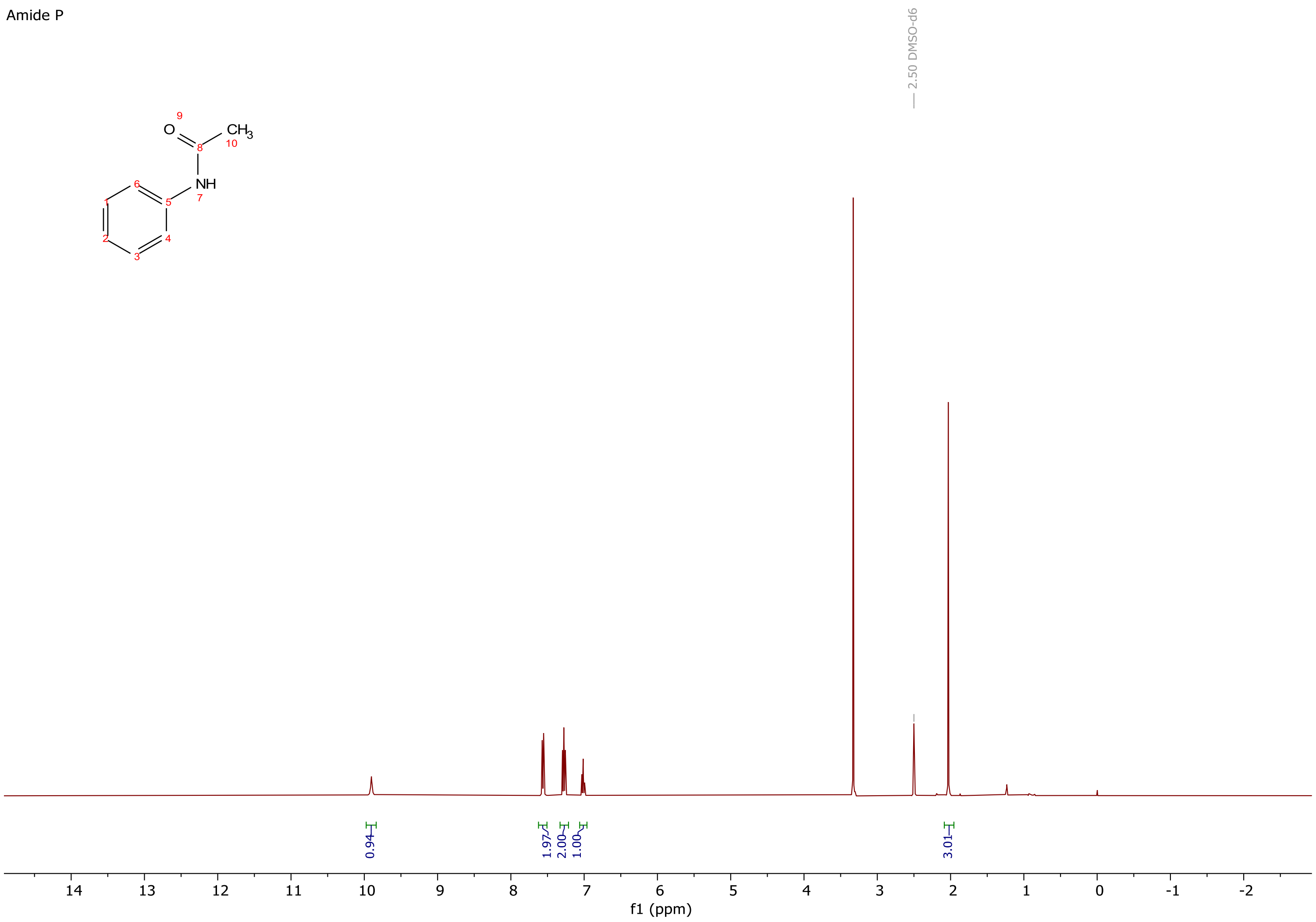

^{13}C NMR spectrum of **3a** (101 MHz, DMSO-d_6)

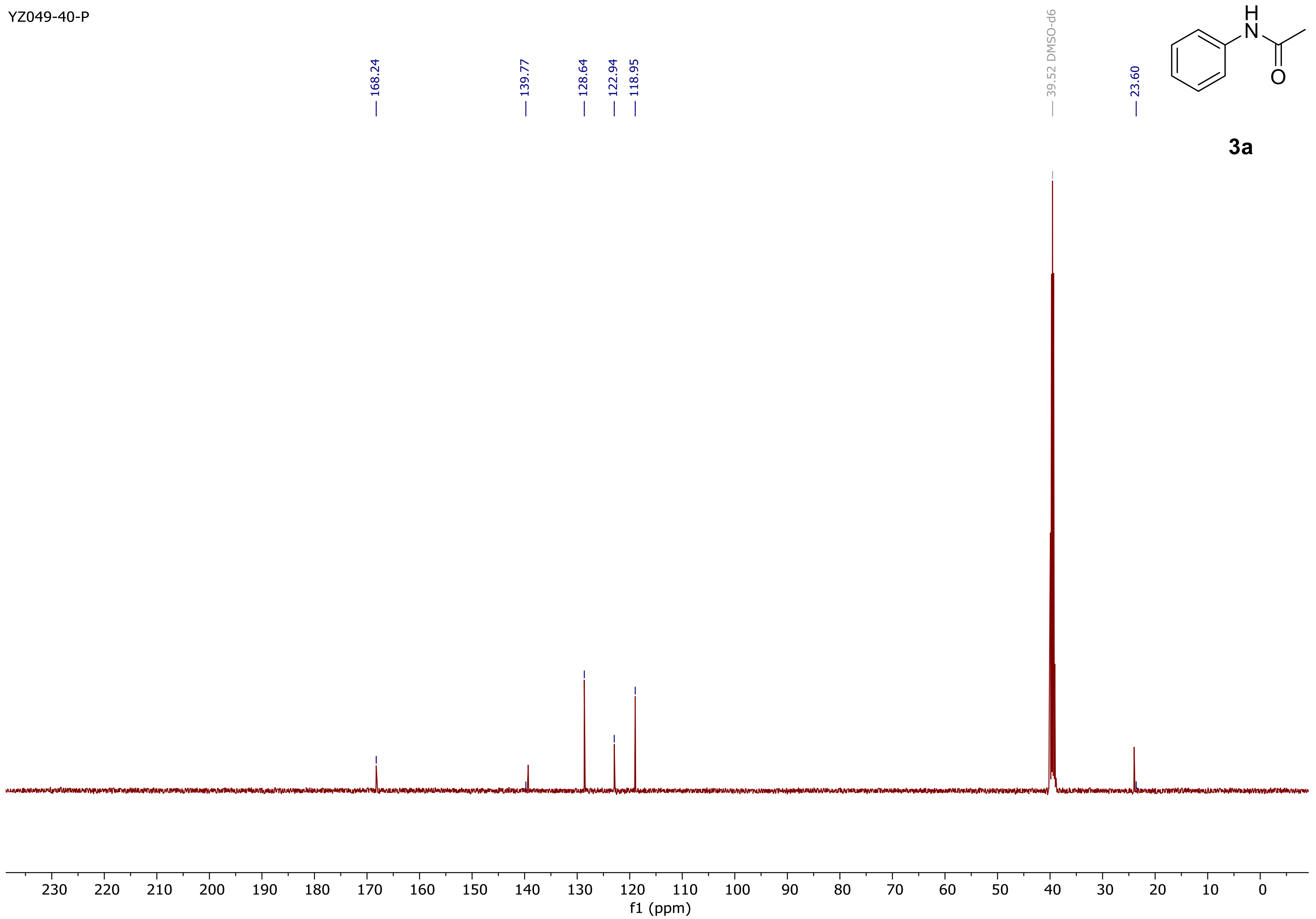

^{1}H NMR spectrum of **3c** (400 MHz, DMSO-d_6)

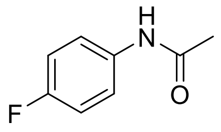

3c

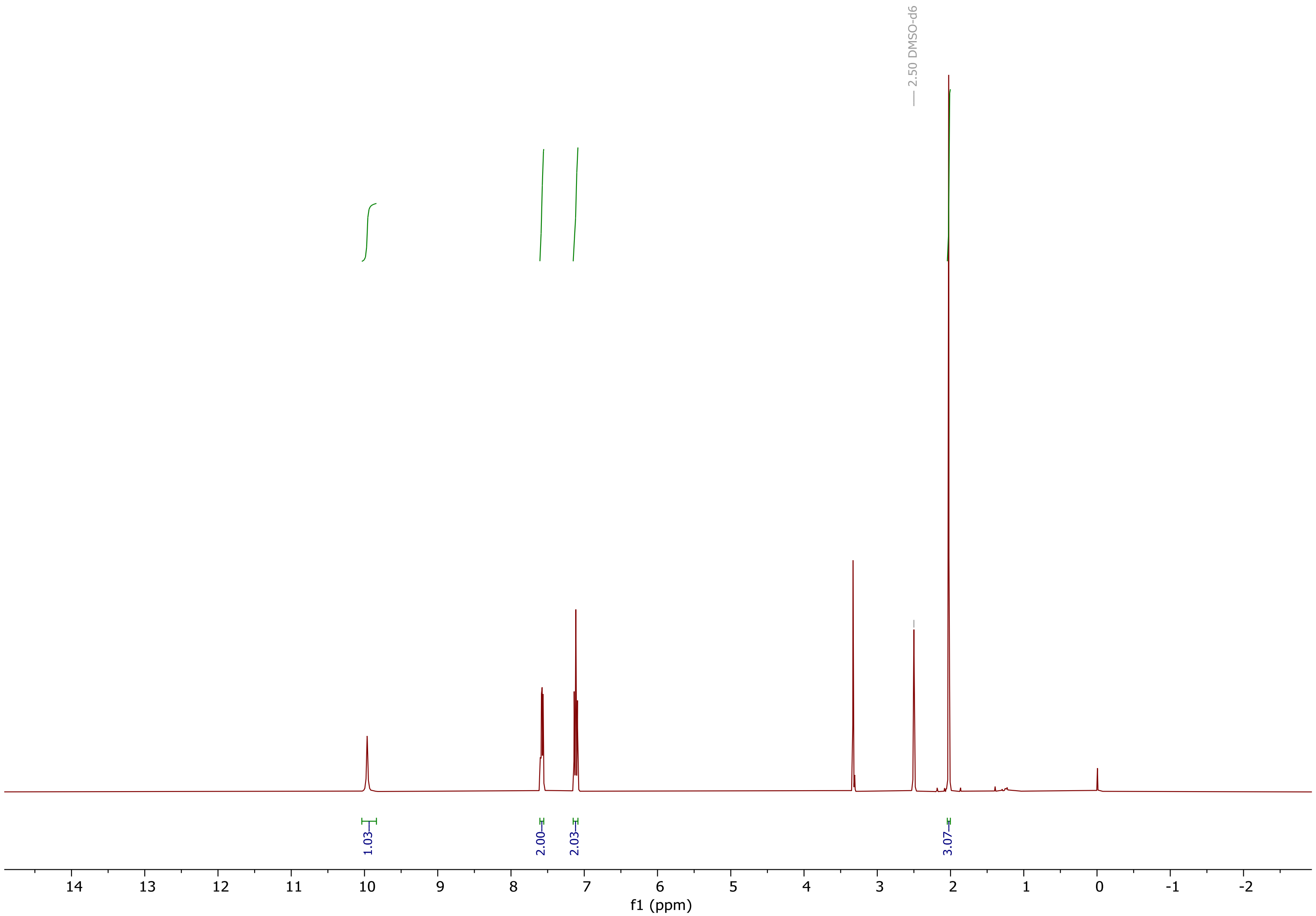

^{13}C NMR spectrum of **3c** (101 MHz, DMSO-d$_6$)

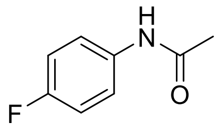

3c

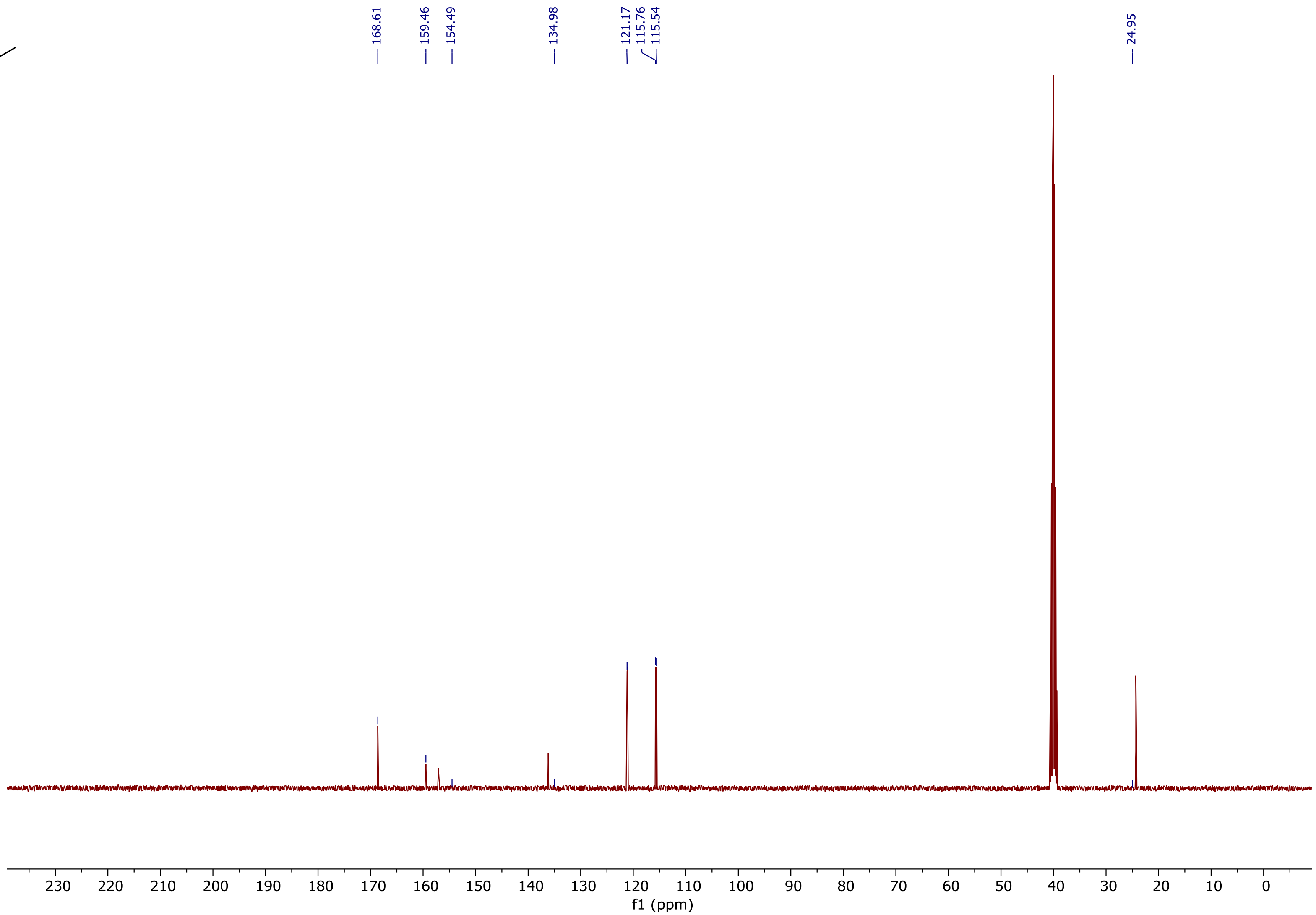

^{19}F NMR spectrum of **3c** (377 MHz, DMSO-d_6)

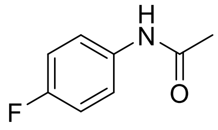

3c

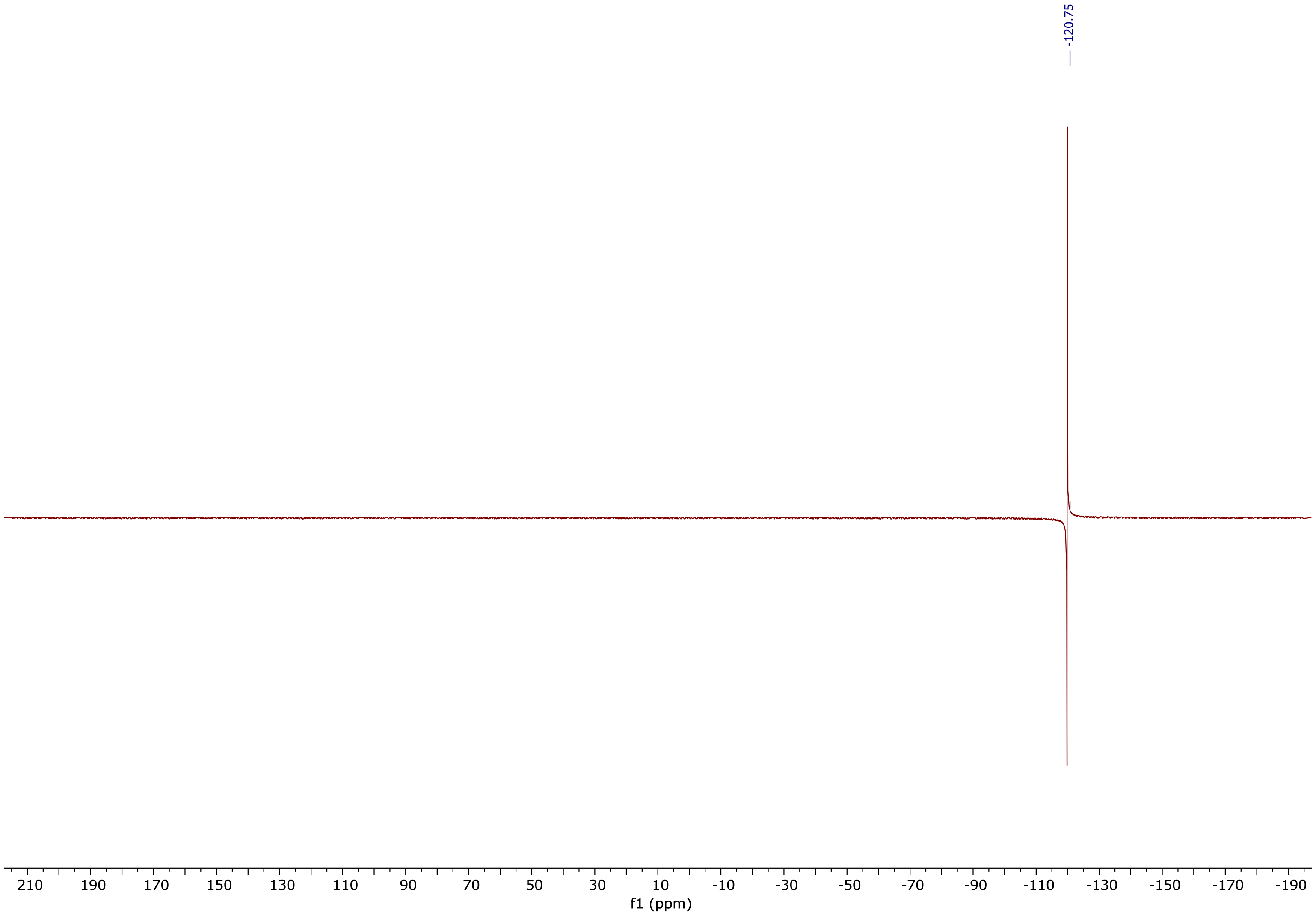

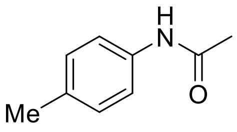

3d

^{1}H NMR spectrum of **3d** (400 MHz, DMSO-d_6)

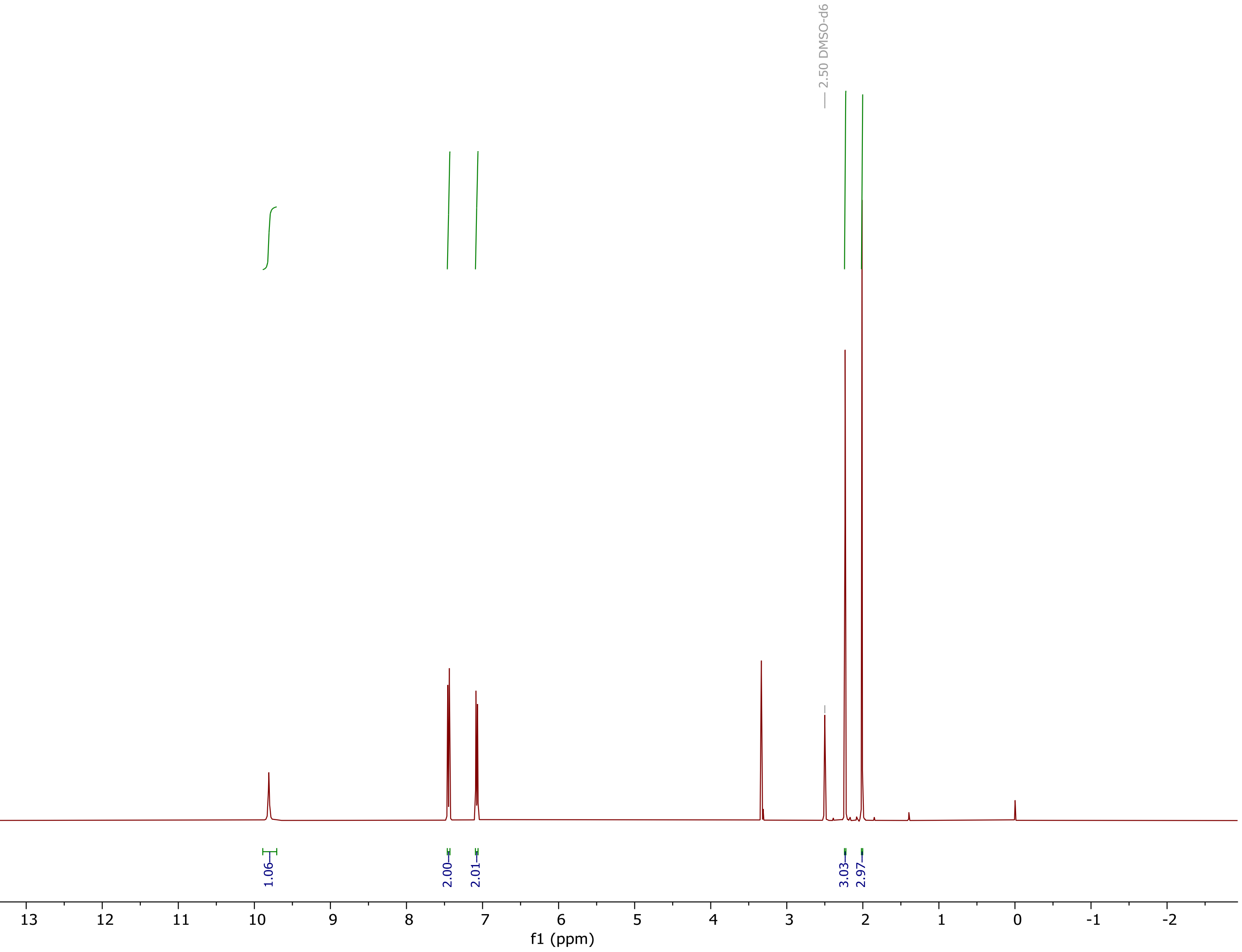

^{13}C NMR spectrum of **3d** (101 MHz, DMSO-d$_6$)

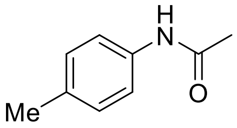

3d

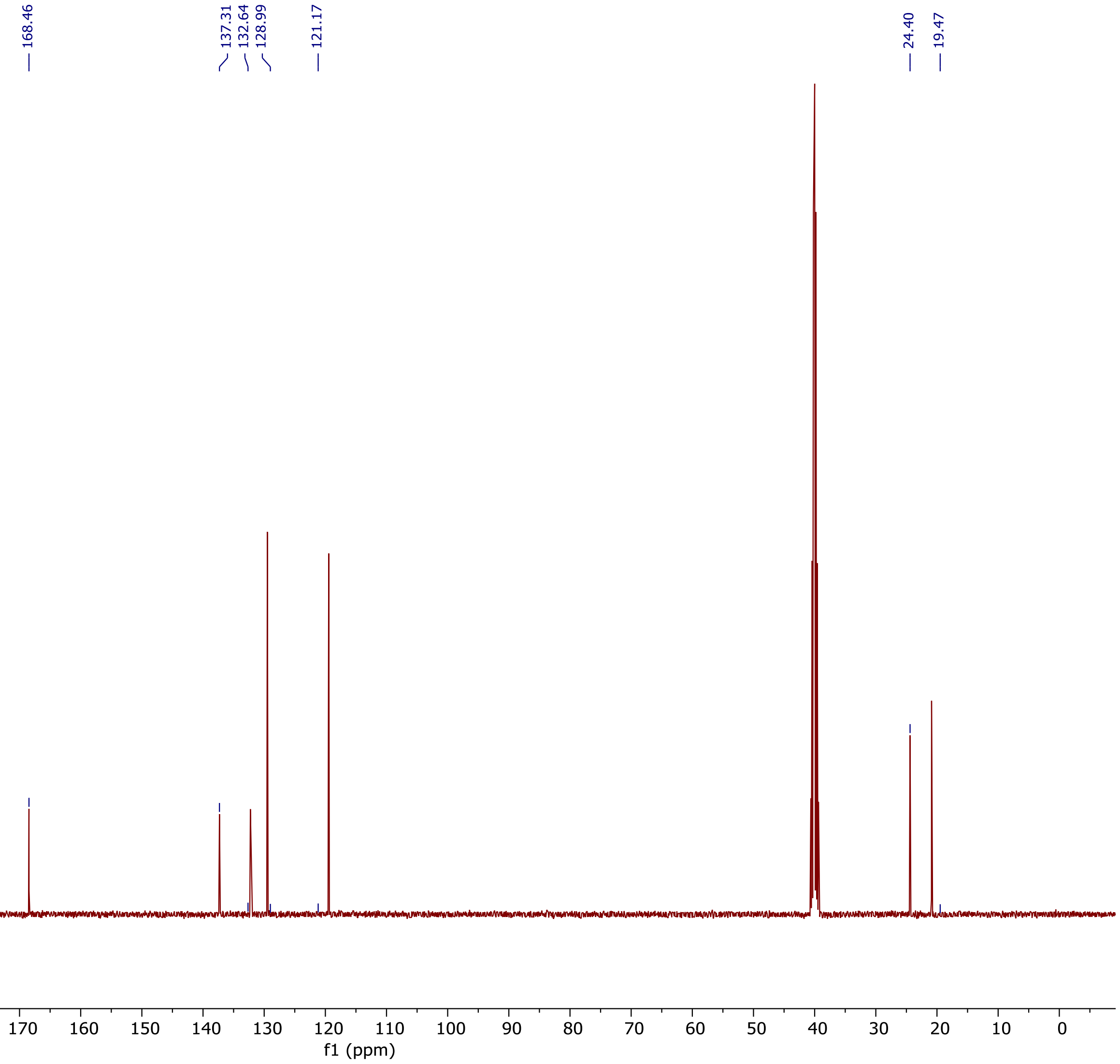

^{1}H NMR spectrum of **3e** (400 MHz, DMSO-d$_6$)

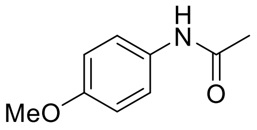

3e

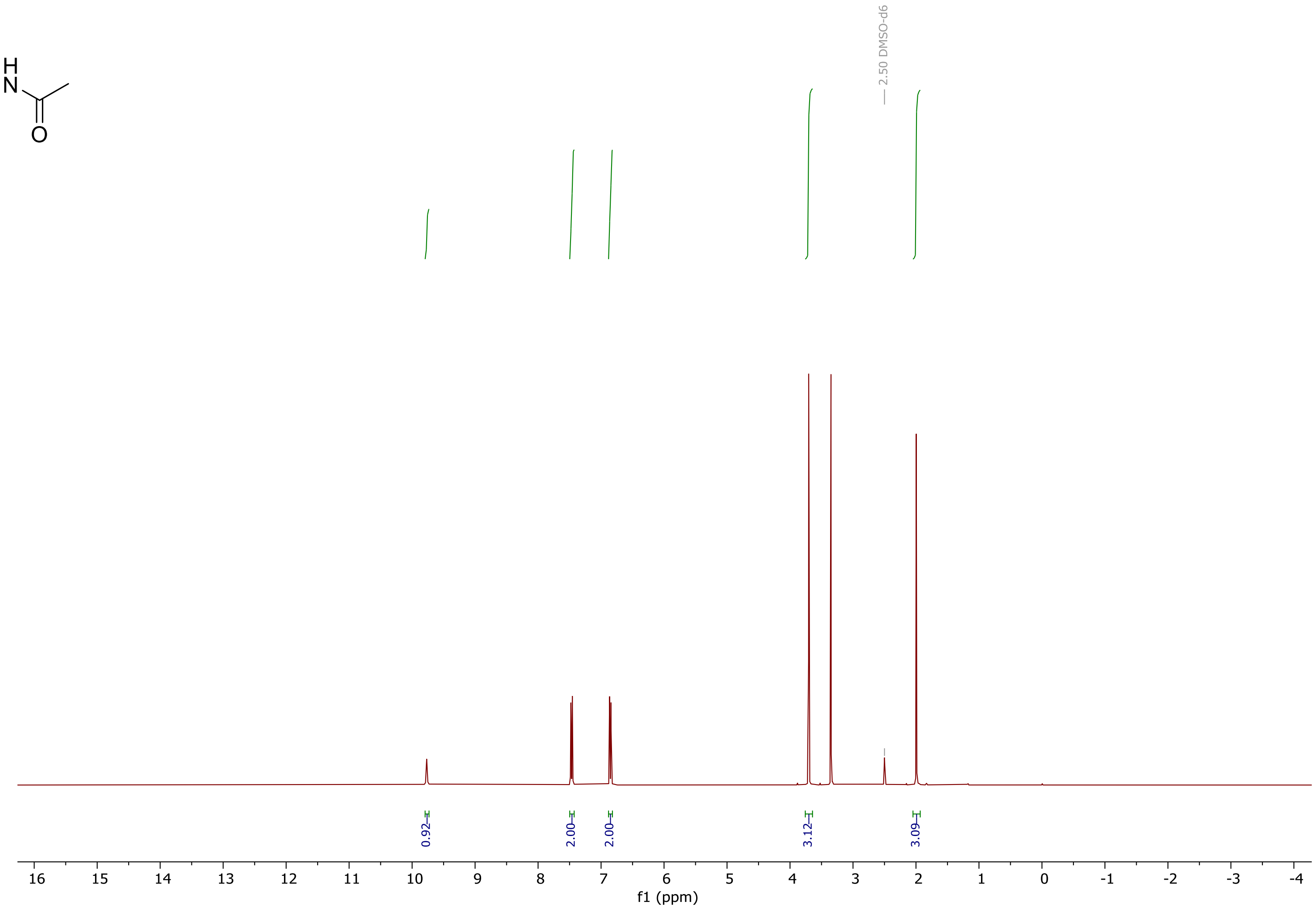

^{13}C NMR spectrum of **3e** (101 MHz, DMSO-d$_6$)

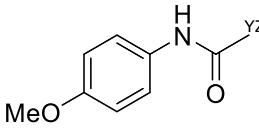

3e

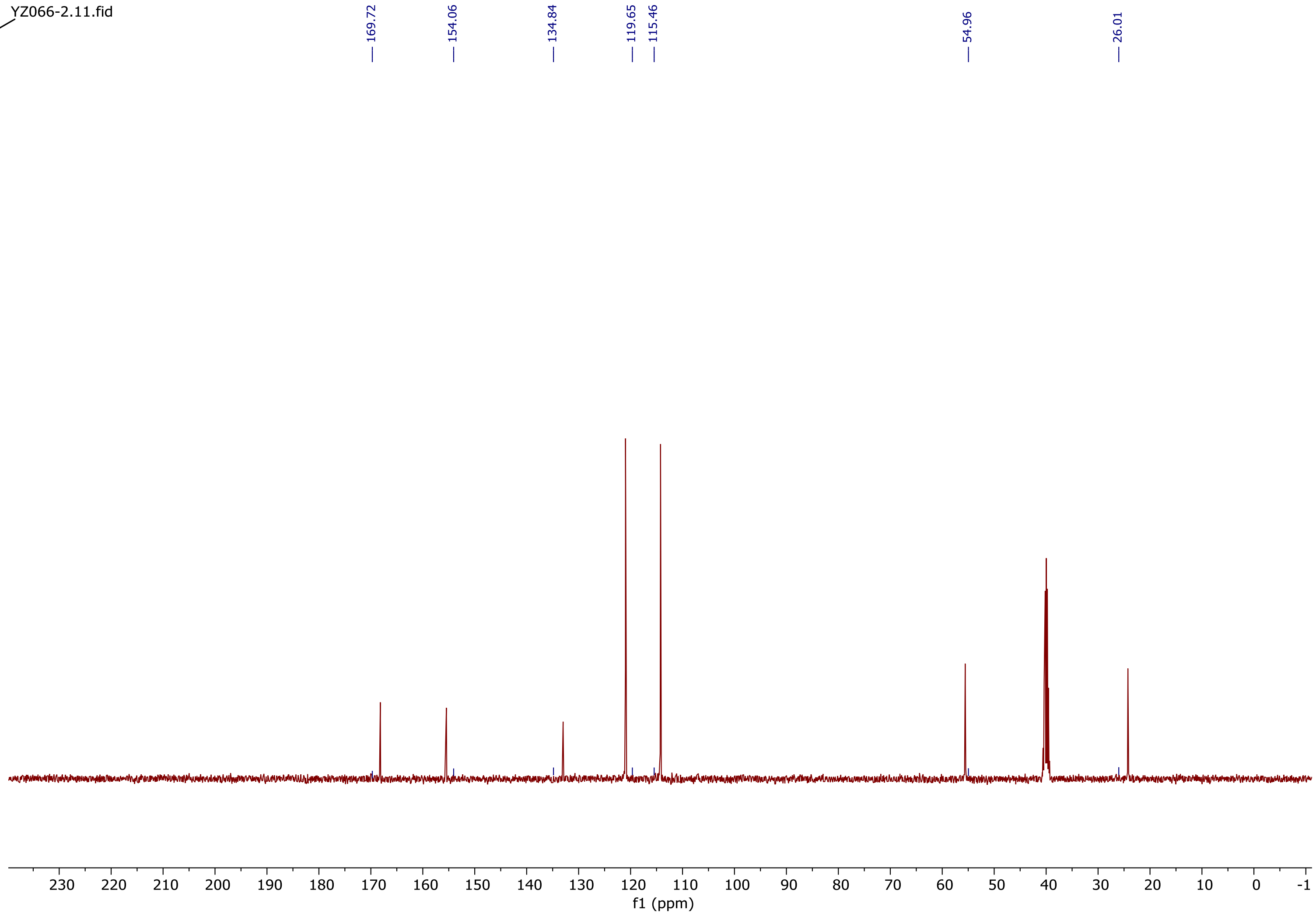

^{1}H NMR spectrum of **3h** (400 MHz, DMSO-d_6)

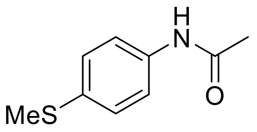

3h

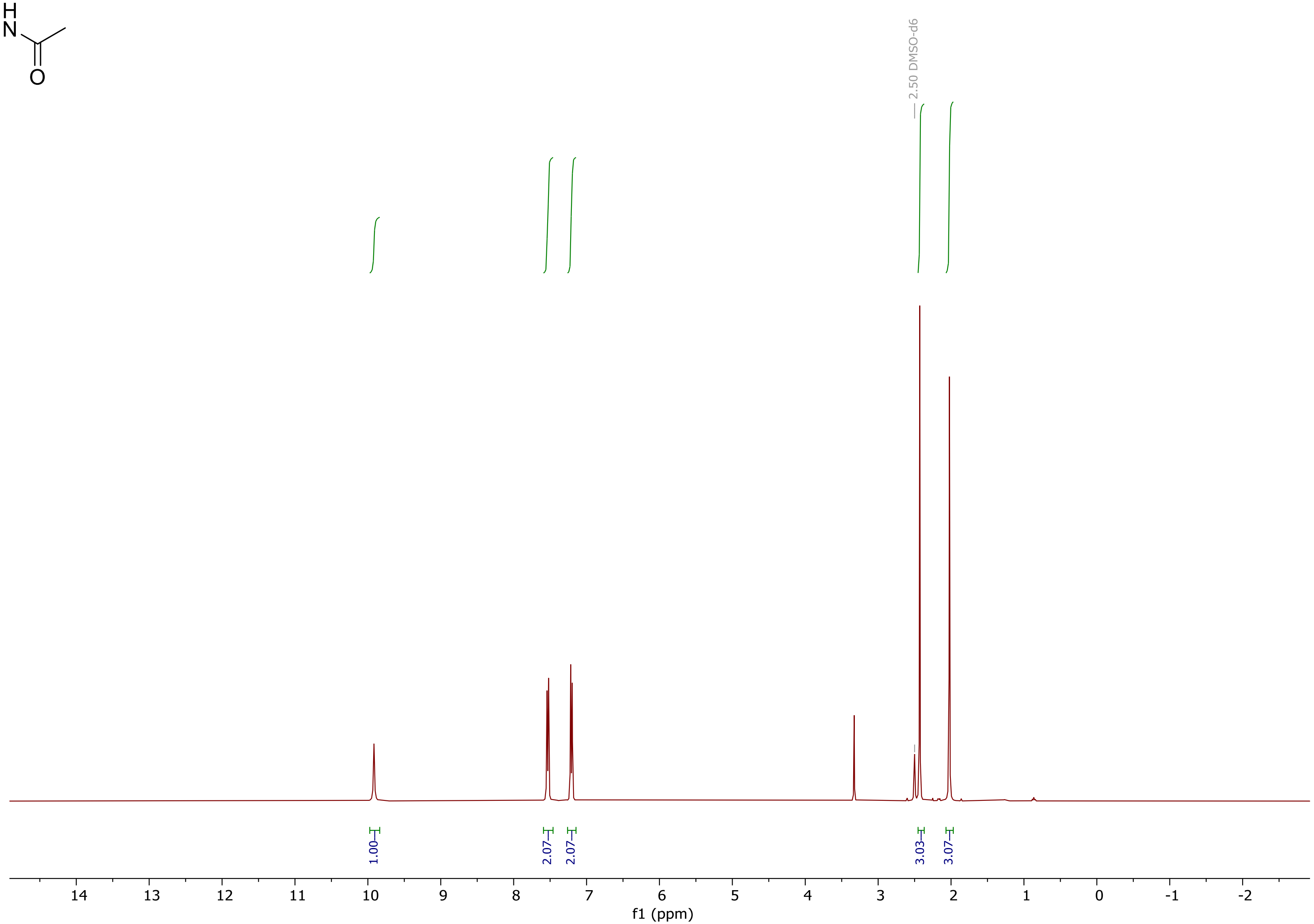

^{13}C NMR spectrum of **3h** (101 MHz, DMSO-d$_6$)

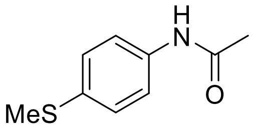

3h

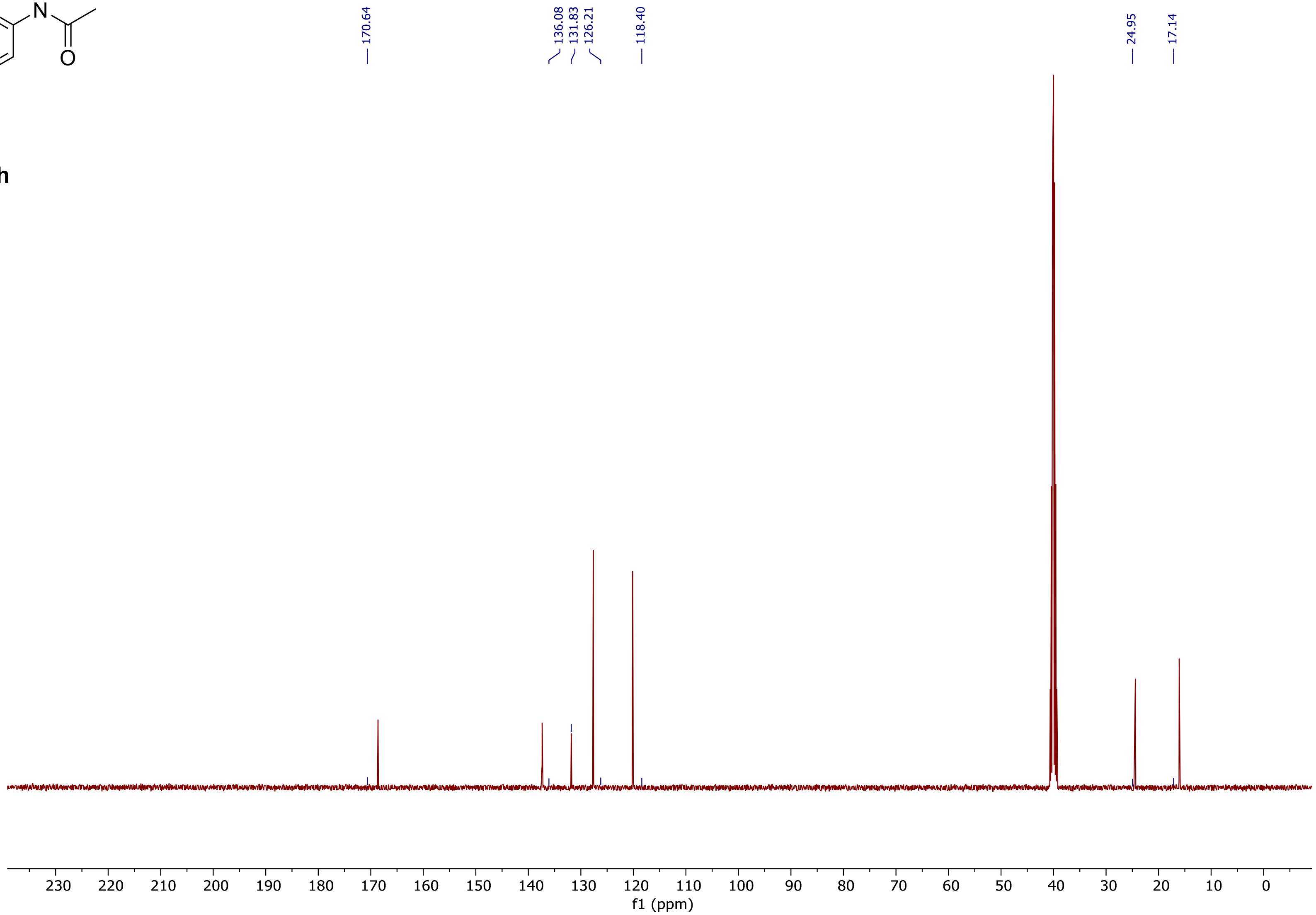

^{1}H NMR spectrum of **3i** (400 MHz, DMSO-d_6)

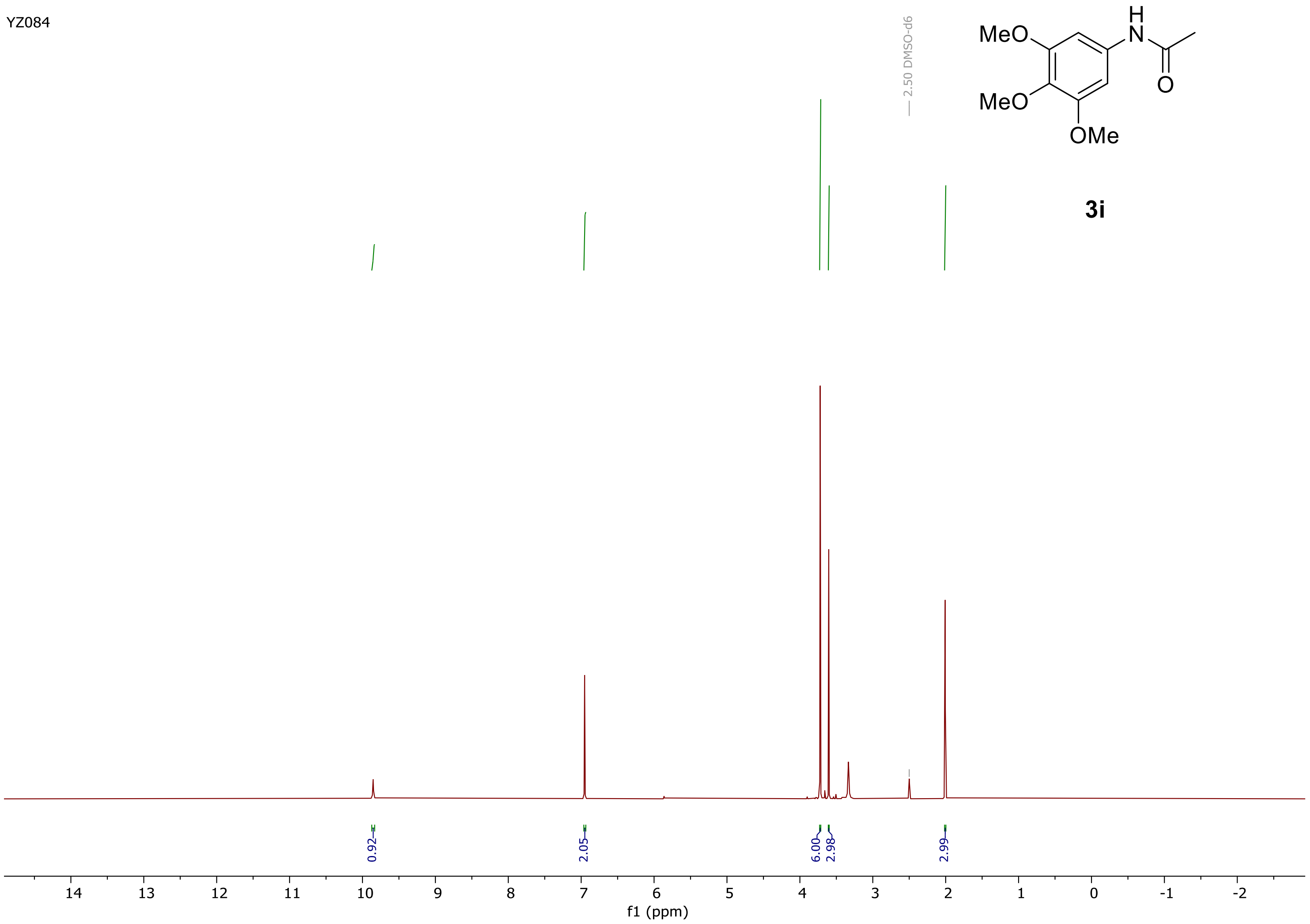

3i

^{13}C NMR spectrum of **3i** (101 MHz, DMSO-d_6)

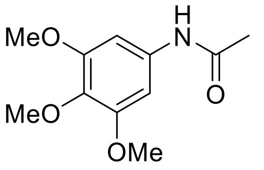

3i

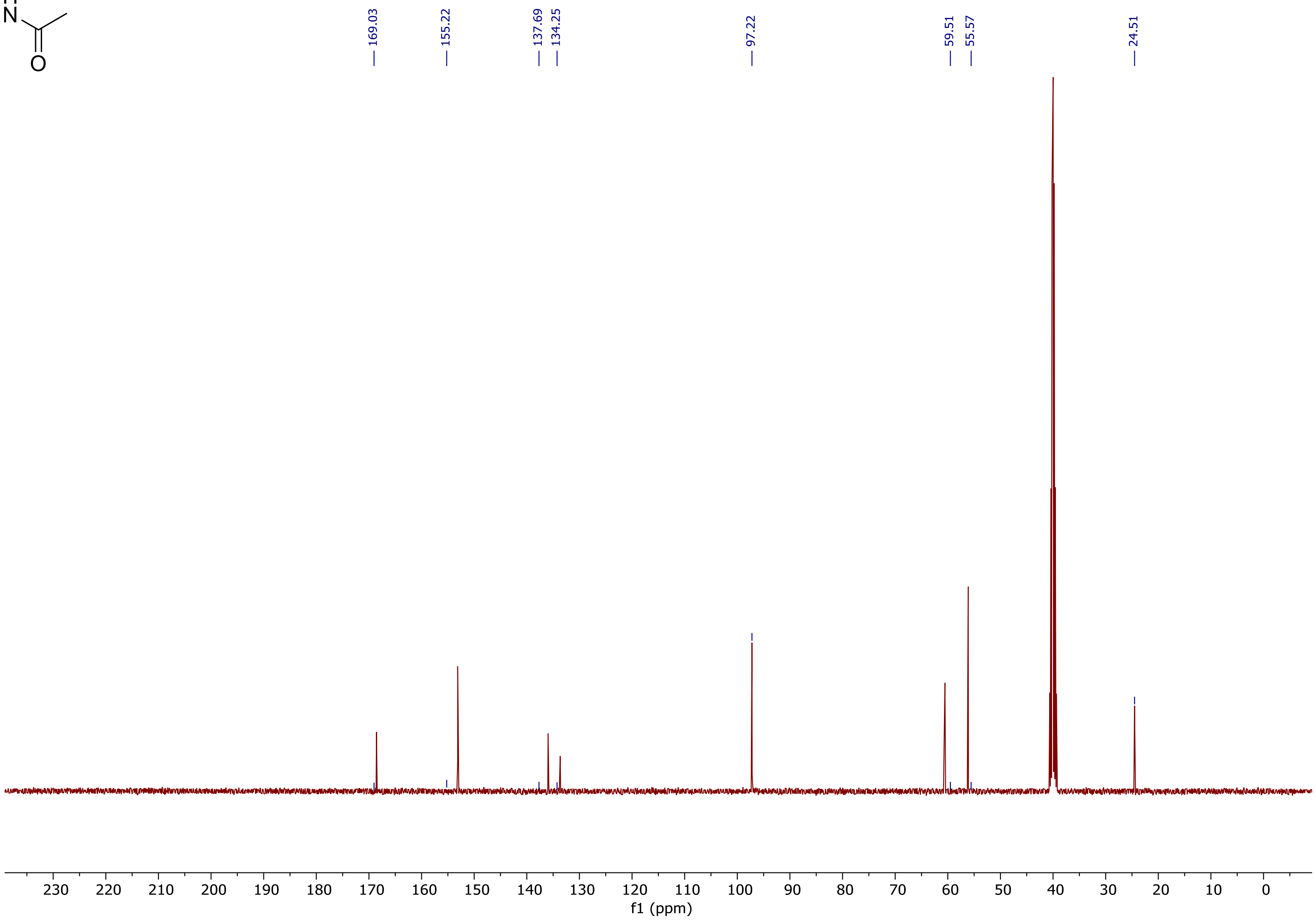

^{1}H NMR spectrum of **3j** (400 MHz, DMSO-d_6)

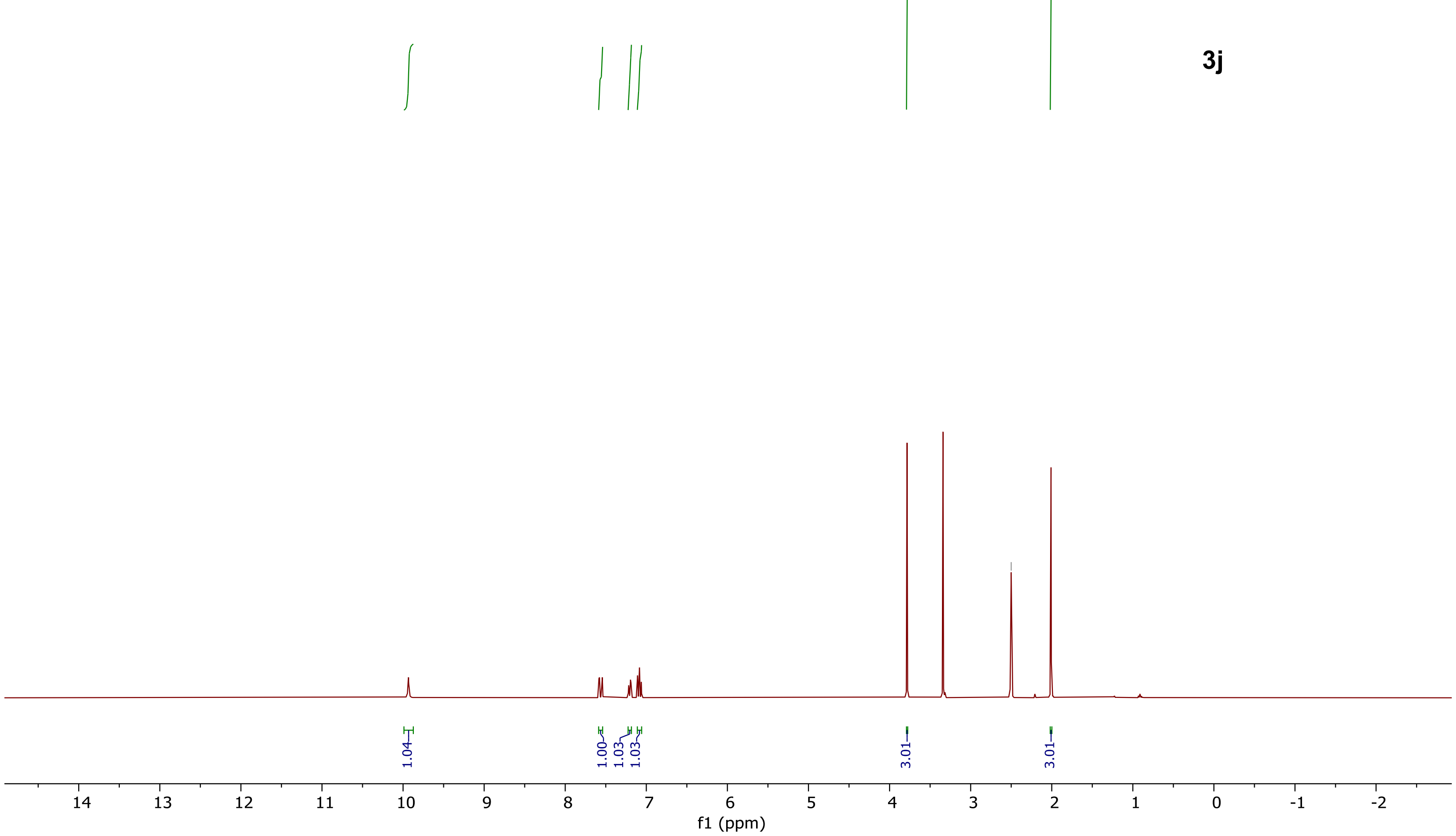

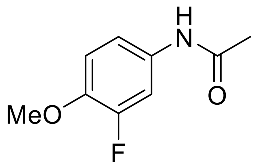

3j

^{13}C NMR spectrum of **3j** (101 MHz, DMSO-d_6)

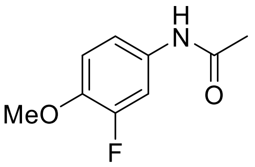

3j

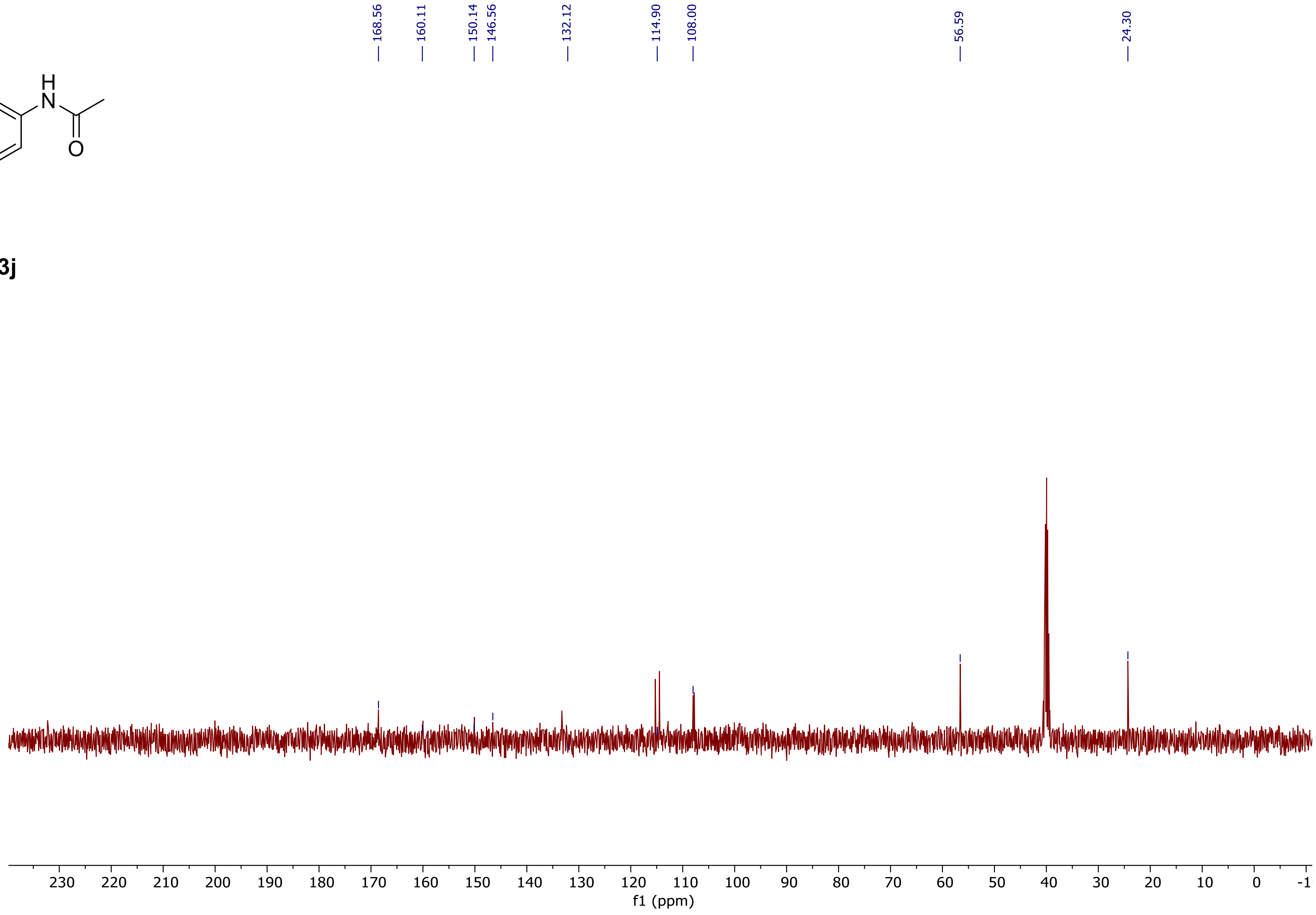

^{19}F NMR spectrum of **3j** (377 MHz, DMSO-d_6)

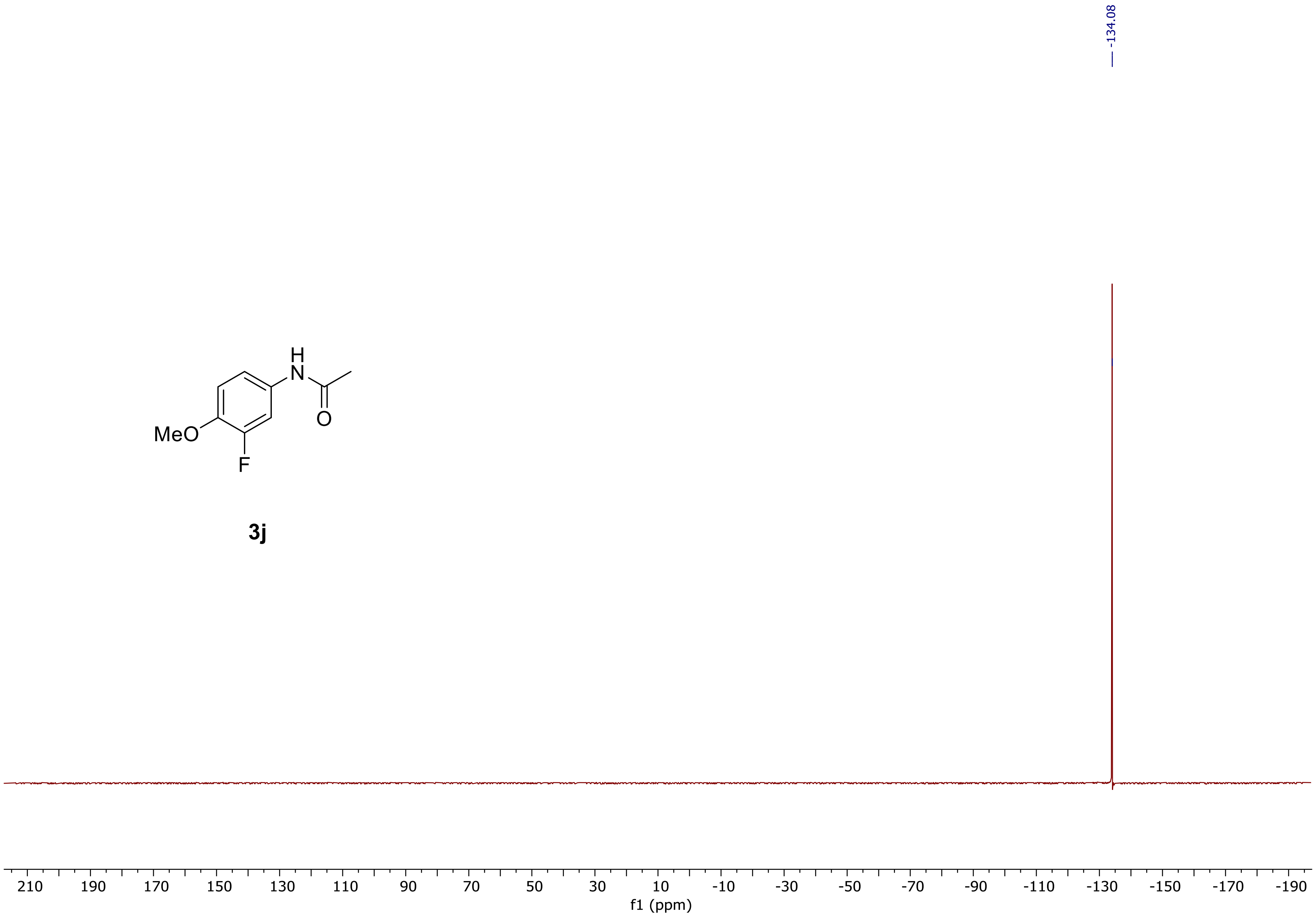

3j

^{1}H NMR spectrum of **3l** (400 MHz, DMSO-d_6)

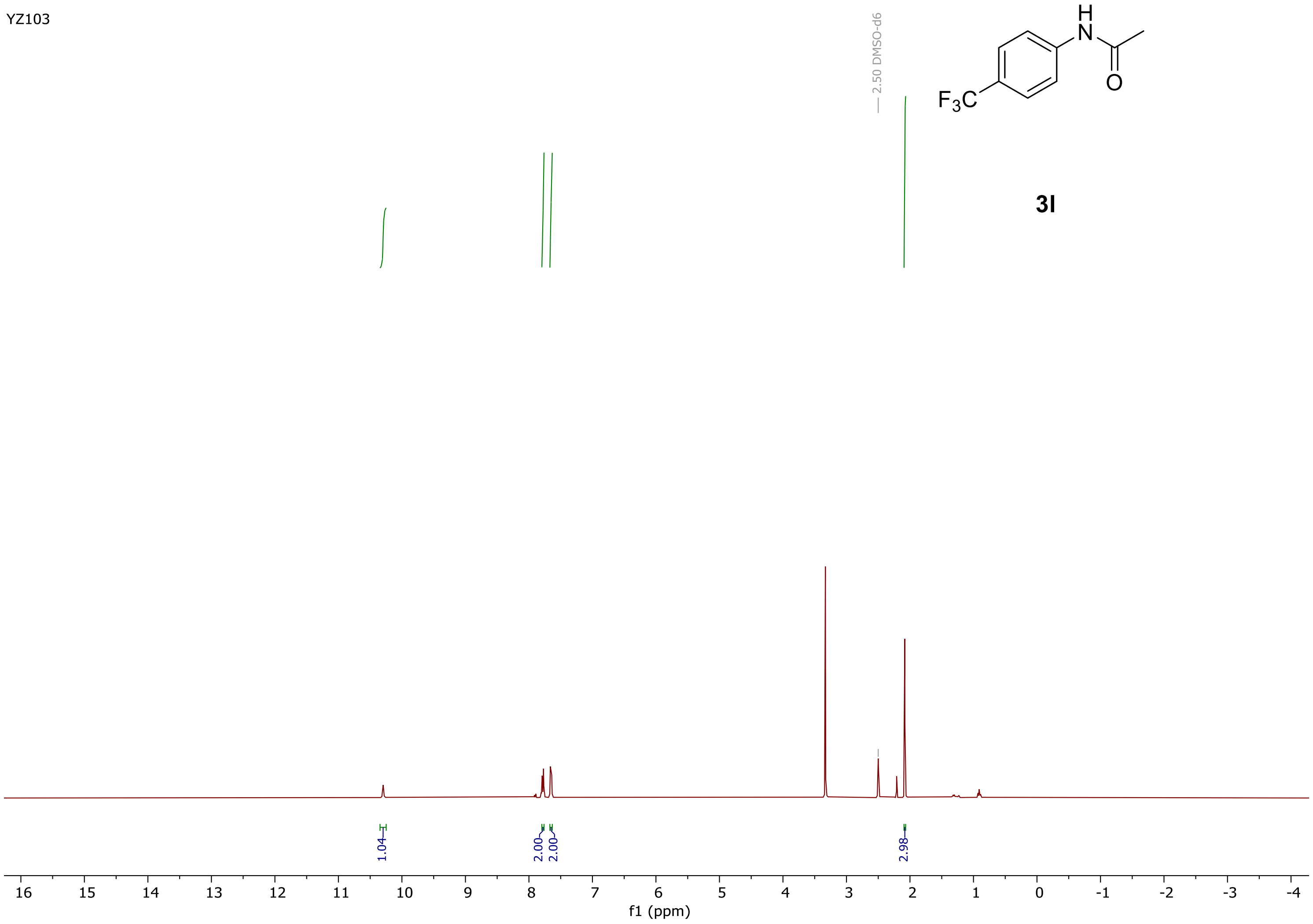

3l

^{13}C NMR spectrum of **3l** (101 MHz, DMSO-d$_6$)

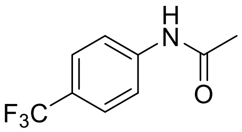

3l

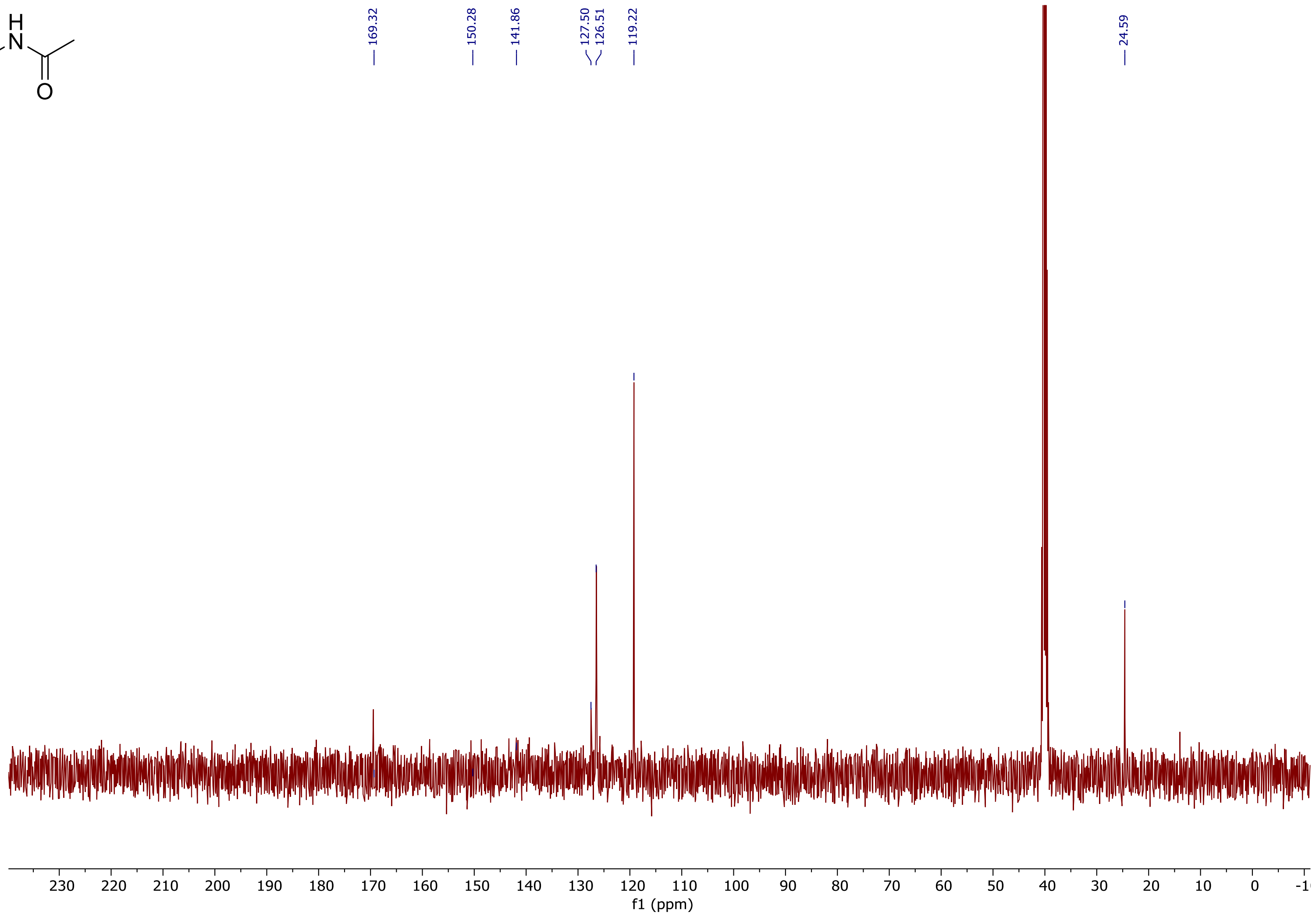

^{19}F NMR spectrum of **3l** (377 MHz, DMSO-d_6)

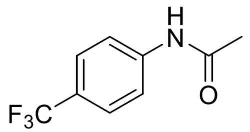

3l

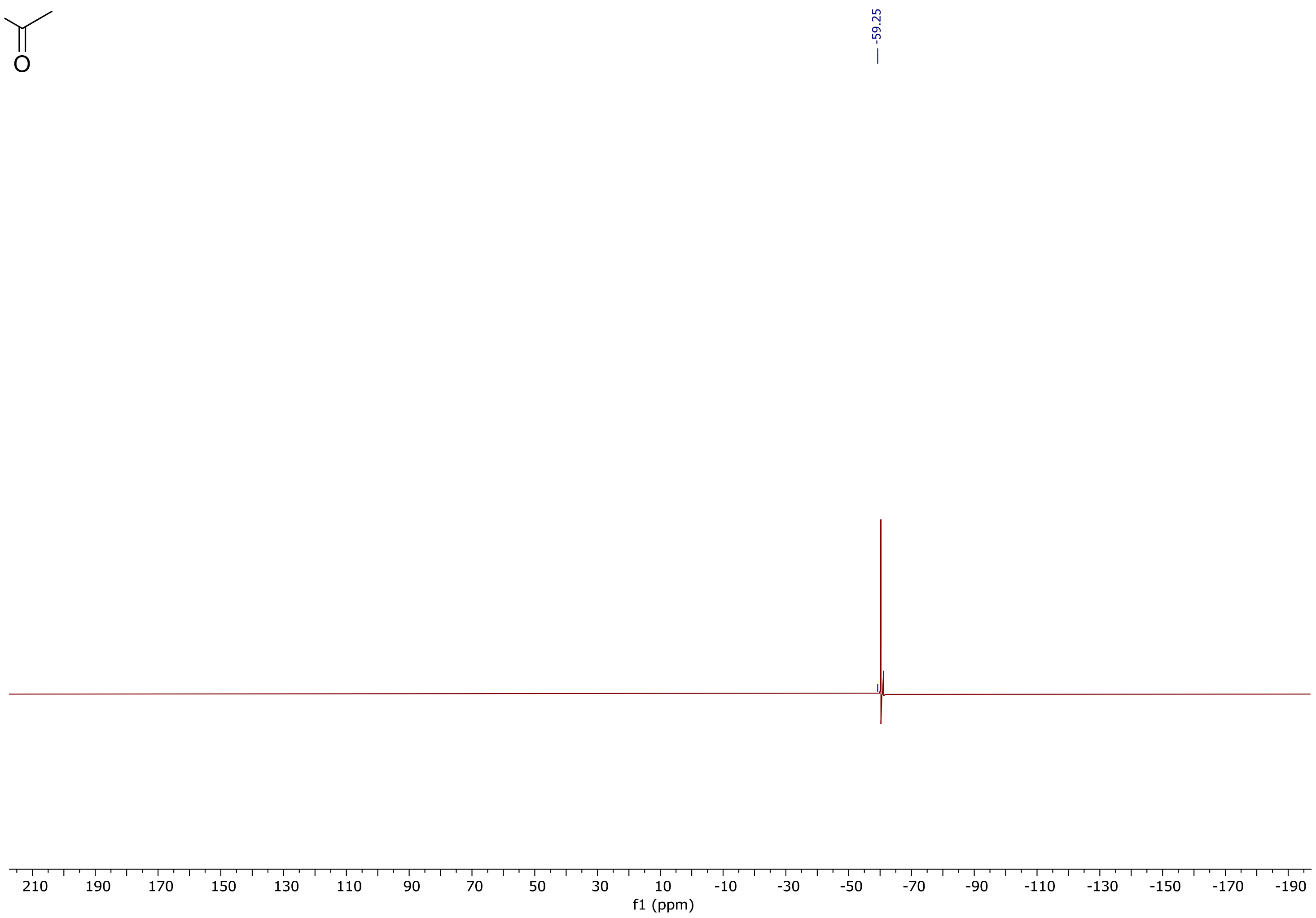

^{1}H NMR spectrum of **3m** (400 MHz, DMSO-d_6)

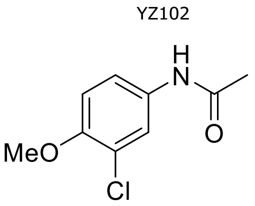

3m

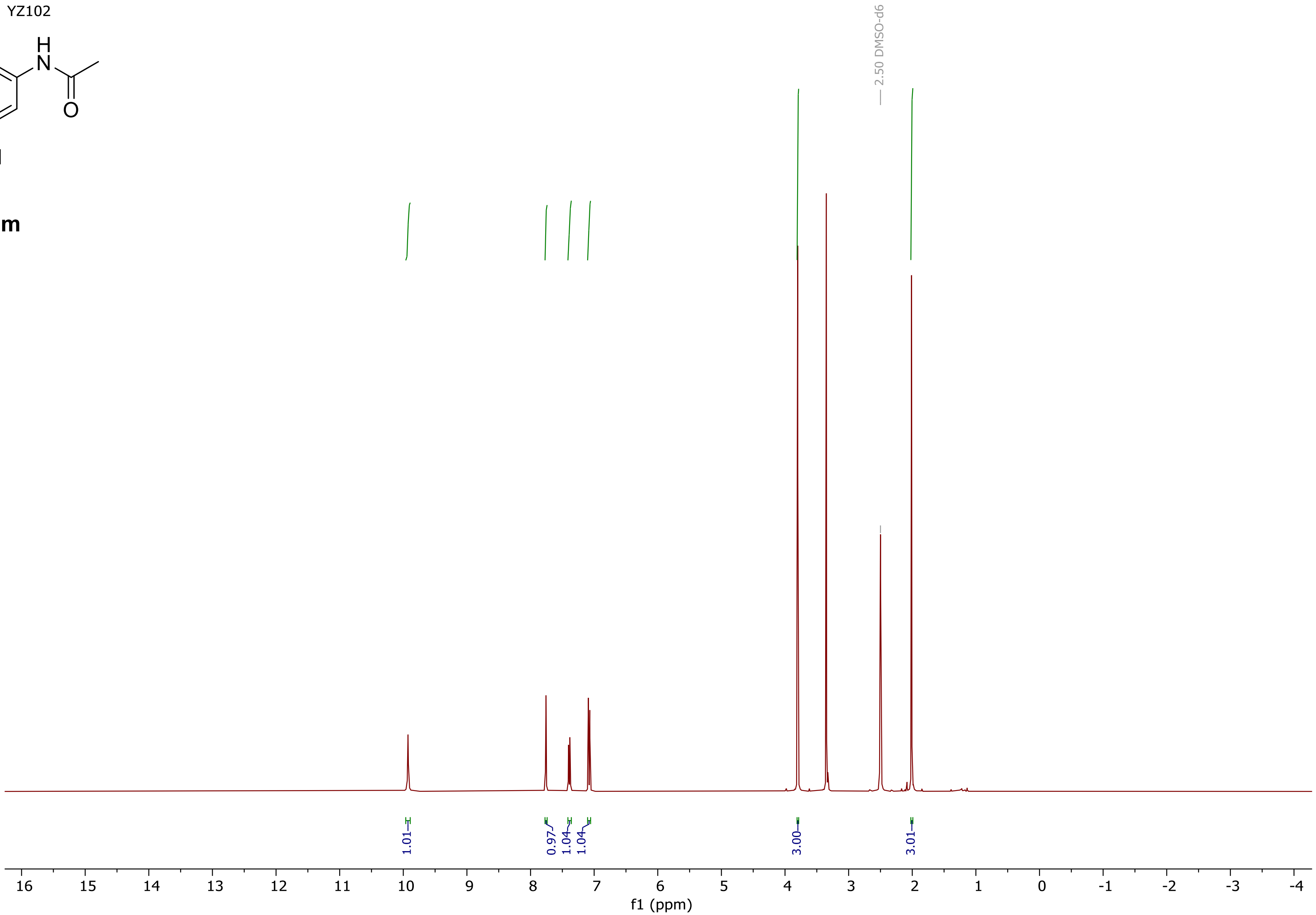

^{13}C NMR spectrum of **3m** (101 MHz, DMSO-d$_6$)

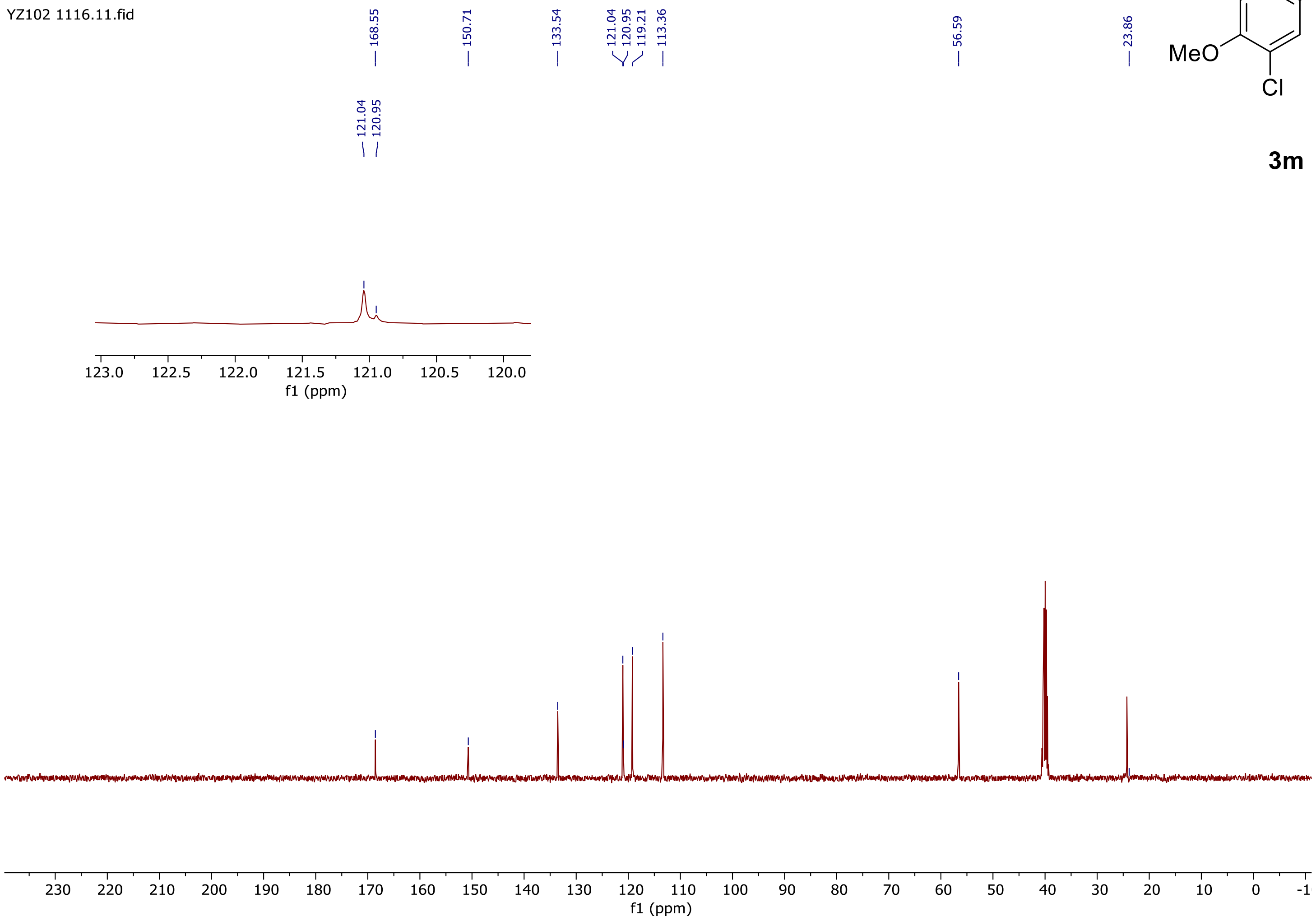

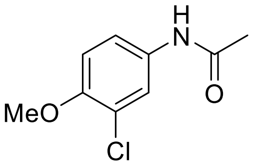

3m

^{1}H NMR spectrum of **3n** (400 MHz, DMSO-d$_6$)

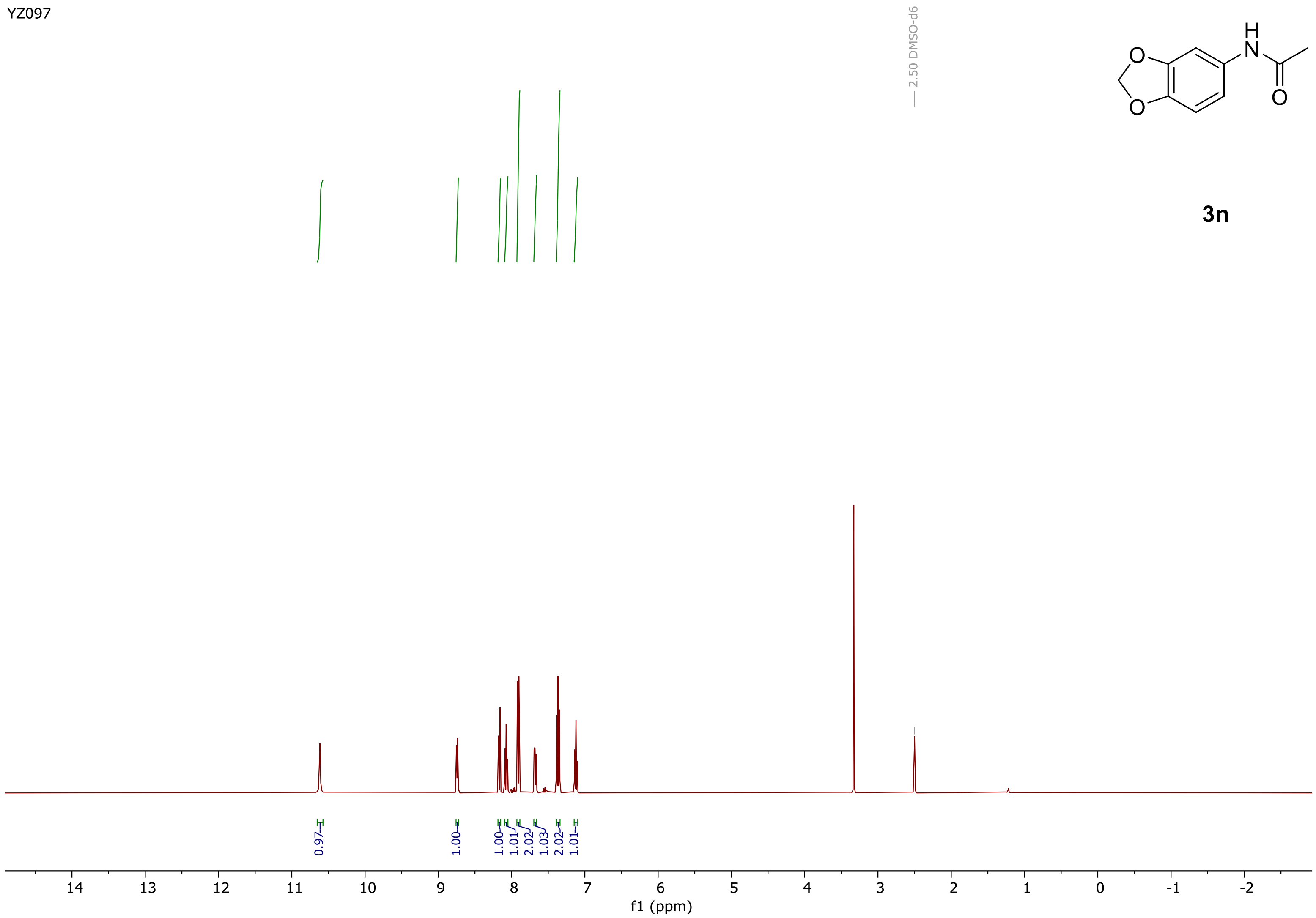

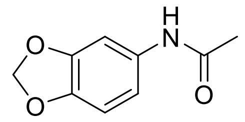

3n

^{13}C NMR spectrum of **3n** (101 MHz, DMSO-d$_6$)

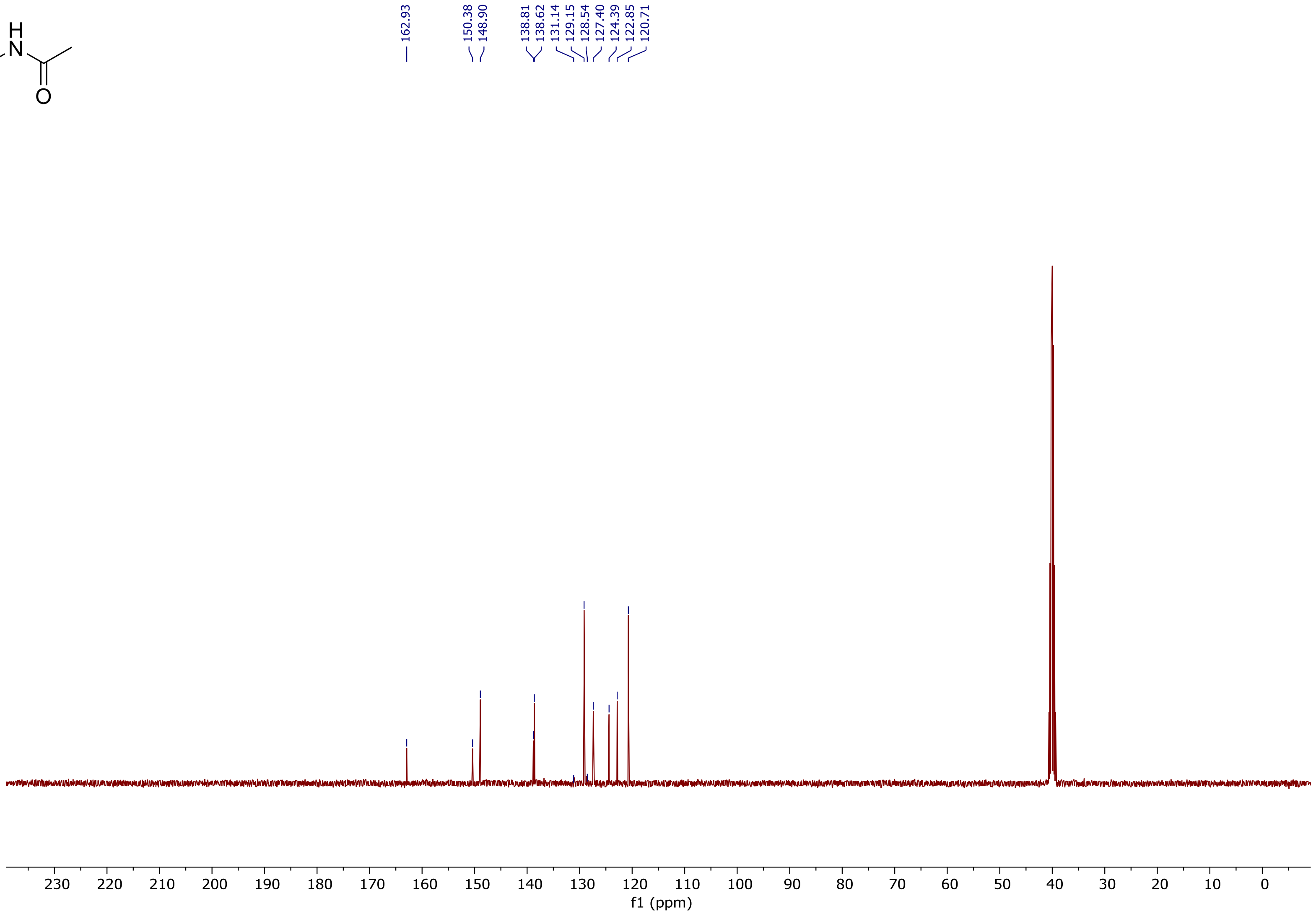

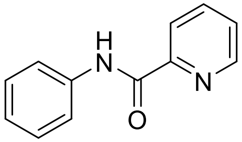

3s

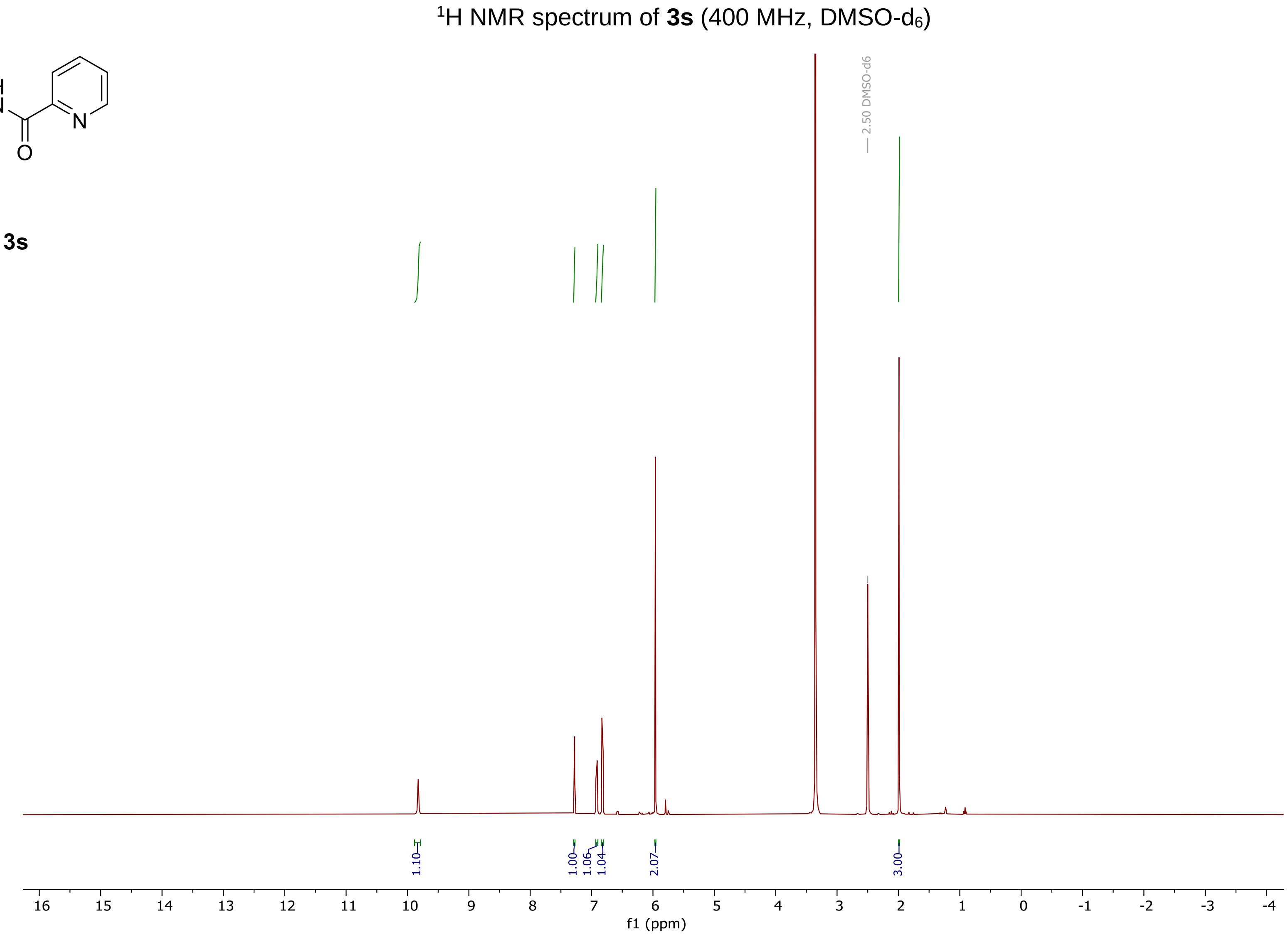
^{1}H NMR spectrum of **3s** (400 MHz, DMSO-d$_6$)
2.50 DMSO-d6
1.10
1.00
1.06
1.04
2.07
3.00
16
15
14
13
12
11
10
9
8
7
6
5
4
3
2
1
0
-1
-2
-3
-4
f1 (ppm)

^{13}C NMR spectrum of **3s** (101 MHz, DMSO-d_6)

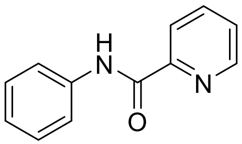

3s

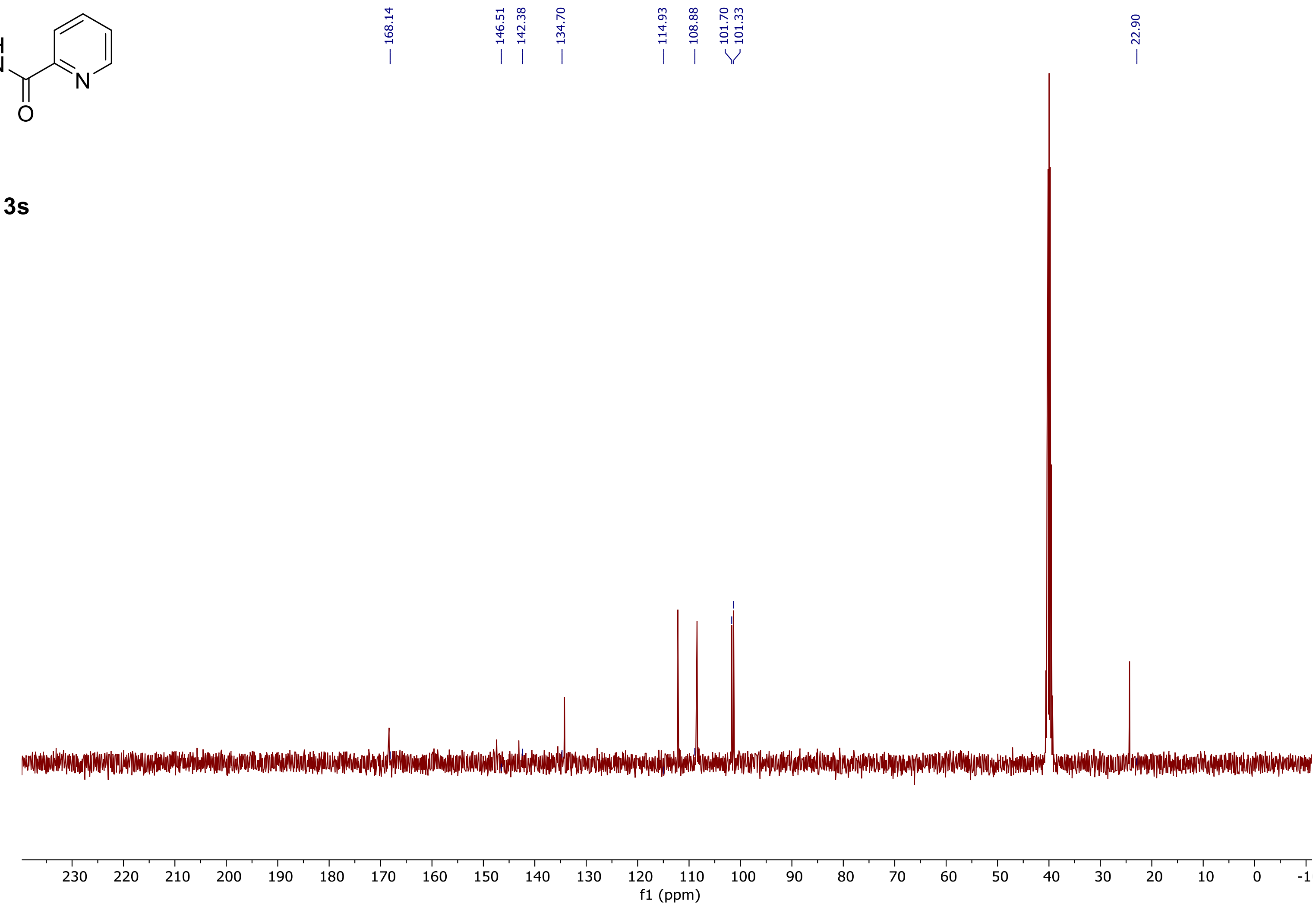

References

S1. Han, Z.; Lv, J.; Zhang, J. One-pot synthesis of 2-amino-3,4-dicyanopyridines from ketoximes and tetracyanoethylene via Cu(I)-catalyzed cyclization *Tetrahedron*, **2019**, 75, 2162-2168.

S2. Chen, Y.; Cantillo, D.; Kappe, C.O. Visible Light-Promoted Beckmann Rearrangements: Separating Sequential Photochemical and Thermal Phenomena in a Continuous Flow Reactor *Eur. J. Org. Chem.* 2019, *2019*, 2163-2171.

S3. Sonn, A. Über einige synthetische Versuche mit O-Trimethyl-gallusaldehyd. (Mitbearbeitet von Ernst Müller, Wolfgang Bülow und Walter Meyer). Ber. dtsch. Chem. Ges. A/B, **1925**, *58,* 1103-1110.

S4. Mahajan, P.S.; Mahajan, J.P.; Mhaske, S.B. Malonic ester amide synthesis. An efficient methodology for synthesis of amides *Synth. Commun*. **2013**, *43*, 2508-2516.

S5. Tang, L.; Wang, Z.-L.; Wan, H.-L.; He, Y.-H.; Guan, Z. Visible-Light-Induced Beckmann Rearrangement by Organic Photoredox Catalysis *Org. Lett.* **2020**, *22*, 6182-6186.

S6. Bhattacharya, S.; Ghosh, P.; Basu, B. Graphene oxide (GO) catalyzed transamidation of aliphatic amides: An efficient metal-free procedure *Tetrahedron Lett.* **2018**, *59*, 899-903.

S7. Jat, J.L.; Kumar, P.; Verma, S.; Chandra, D.; Singh, V.; Tiwari, B. Metal-free synthesis of secondary amides using N-Boc-O-tosylhydroxylamine as nitrogen source via Beckmann rearrangement *New J. Chem.* **2022**, *46*, 14782-14785.

S8. Ito, A.; Asami, Y.; Asato, M.; Fukuda, K.; Yamasaki, R.; Okamoto, I. Synthesis and conformational analysis of N-aryl-N-(3-thienyl)acetamides *Tetrahedron Lett.* **2018**, *59*, 2454-2458.

S9. Mahajan, P.S.; Humne, V.T.; Tanpure, S.D.; Mhaske, S.B. Radical Beckmann Rearrangement and Its Application in the Formal Total Synthesis of Antimalarial Natural Product Isocryptolepine via C–H Activation *Org. Lett.* **2016**, *18*, 3450-3453.

S10. Ran, M.; He, J.; Yan, B.; Liu, W.; Li, Y.; Fu, Y.; Li, S.-J.; Yao Q. Catalyst-free generation of acyl radicals induced by visible light in water to construct C–N bonds *Org. Biomol. Chem.* **2021**, *19*, 1970-1975.

S11.Chip, G.K.; Grossert, J.S. Aromatic Halogenation with Titanium (IV) Chloride in Presence of Peroxytrifluoroacetic Acid *Can. J. Chem.* **1972**, *50*, 1233 – 1240.

S12. Crisenza, G.E.M.; Sokolova, O.O.; Bower, J.F. Branch-Selective Alkene Hydroarylation by Cooperative Destabilization: Iridium-Catalyzed ortho-Alkylation of Acetanilides *Angew. Chem. Int. Ed.* **2015**, *54*, 14866-14870.

S13. Fu, Z.; Wang, X.; Tao, S.; Bu, Q.; Wei, D.; Liu, N. Manganese Catalyzed Direct Amidation of Esters with Amines *J. Org. Chem.* **2021**, *86*, 2339-2358.
